# Supplementary material for: Enantioselective formal (3 + 3) cycloaddition of bicyclobutanes with nitrones enabled by asymmetric Lewis acid catalysis
Source: Nat Commun. 2024 Sep 13;15:8005. doi: 10.1038/s41467-024-52419-x (PMC11393060; doi:10.1038/s41467-024-52419-x)
Supplement: Supplementary file 1 — Supplementary Information [file 41467_2024_52419_MOESM1_ESM.pdf]

Enantioselective formal (3+3) cycloaddition of bicyclobutanes with nitrones enabled by asymmetric Lewis acid catalysis

Wen-Biao Wu,[1,2,3,4] Bing Xu,[4,5] Xue-Chun Yang,[1,4] Feng Wu,[1] Heng-Xian He,[1] Xu Zhang,[2] and Jian-Jun Feng*[1]

[1]State Key Laboratory of Chemo/Biosensing and Chemometrics, Advanced Catalytic Engineering Research Center of the Ministry of Education, College of Chemistry and Chemical Engineering, Hunan University, Changsha, 410082, P. R. China.

[2]School of Chemistry & Chemical Engineering, Yangzhou University, Yangzhou, 225002, P. R. China.

[3]School of Physics and Chemistry, Hunan First Normal University, Changsha, 410205, P. R. China.

[4]These authors contributed equally to this work.

[5]Department of Chemistry, Fudan University, Shanghai, 200438, P.R. China.

* Corresponding Author (Jian-Jun Feng): jianjunfeng@hnu.edu.cn

Supplementary information

Table of Contents

1. Supplementary Methods S2
- 1.1 General Information S2
- 1.2 Optimization Study S3
- 1.3 General Procedure for the Enantioselective (3+3) Cycloadditions (GP1) S7
- 1.4 General Procedure for the Synthesis of New Bicyclobutanes (GP2) S7
- 1.5 Scale-Up Experiment S8
- 1.6 Synthetic Transformations S9
- 1.7 General Procedure for the Synthesis of Chiral Ligands S13

2. Supplementary Discussion S14
- 2.1 Unsuccessful BCB Substrates and Control Experiments S14
- 2.2 Non-Linear Effect Study S16
- 2.3 Proposed Catalytic Cycle and Computed Transition Structures S17
- 2.4 Characterization Data of the New BCBs and Products S18

3. X-Ray Crystallography S73

4. Supplementary NMR and HPLC Spectra S75

5. Supplementary Computational Details S201

6. Supplementary References S201

1. Supplementary Methods

1.1 General Information

All reactions were performed in flame-dried glassware using conventional Schlenk techniques under a static pressure of nitrogen unless otherwise stated. Liquids and solutions were transferred with syringes. Bicyclo[1.1.0]butanes (BCBs)[1-4] and nitrones [5-6] were prepared according to reported procedures. $Co(OTf)_2$ (98%, *Bide* Chemical Company) and other commercially available reagents were purchased from *Leyan*, *Energy Chemical* and *Bide* Chemical Company and used as received. The solvents (CH_2Cl_2, 1,2-dichloroethane, Et_2O, THF and toluene *etc*.) were dried and purified following standard procedures. PhCl and Ethyl acetate (EtOAc) were purchased from *Energy Chemical* (99%, Extra Dry) and used as received. Technical grade solvents for extraction or chromatography (Petroleum ether, CH_2Cl_2, and ethyl acetate) were distilled prior to use. Analytical thin layer chromatography (TLC) was performed on silica gel 60 F254 glass plates by *Merck*. Flash column chromatography was performed on silica gel 60 (40–63 μm, 230–400 mesh, ASTM) by *Grace* using the indicated solvents. ^{1}H, ^{13}C NMR spectra were recorded in $CDCl_3$ on Bruker AV400 or 600 instruments. Chemical shifts are reported in parts per million (ppm) and are referenced to the residual solvent resonance as the internal standard ($CDCl_3$: δ = 7.26 ppm for ^{1}H NMR and $CDCl_3$: δ = 77.0 ppm for ^{13}C NMR). Data are reported as follows: chemical shift, multiplicity (s = singlet, d = doublet, t = triplett, q = quartet, m = multiplet), coupling constants (Hz), and integration. The full-scan mass spectra were taken on a hybrid quadrupole-orbitrap mass spectrometer equipped with a heated electrospray ionization source (ThermoFischer Scientific, Bremen, Germany). Chiral HPLC analysis was performed on a Shimadzu LC-20AD instrument using Daicel chiral columns at 35 ^{o}C and a mixture of HPLC-grade hexanes and isopropanol as eluent. Acknowledgement: the ^{1}H, ^{13}C NMR spectra, single crystal X-ray diffraction and HRMS (ESI) were performed at Analytical Instrumentation Center of Hunan University. The absolute configuration was determined by single crystal X-ray diffraction analysis on Rigaku XtaLAB PRO MM003-DS dual system with a Cu micro-focus source. Diffraction data was collected at 173 K on a Rigaku XtaLAB PRO MM003-DS dual System with a Cu micro-focus source.

1.2 Optimization Study

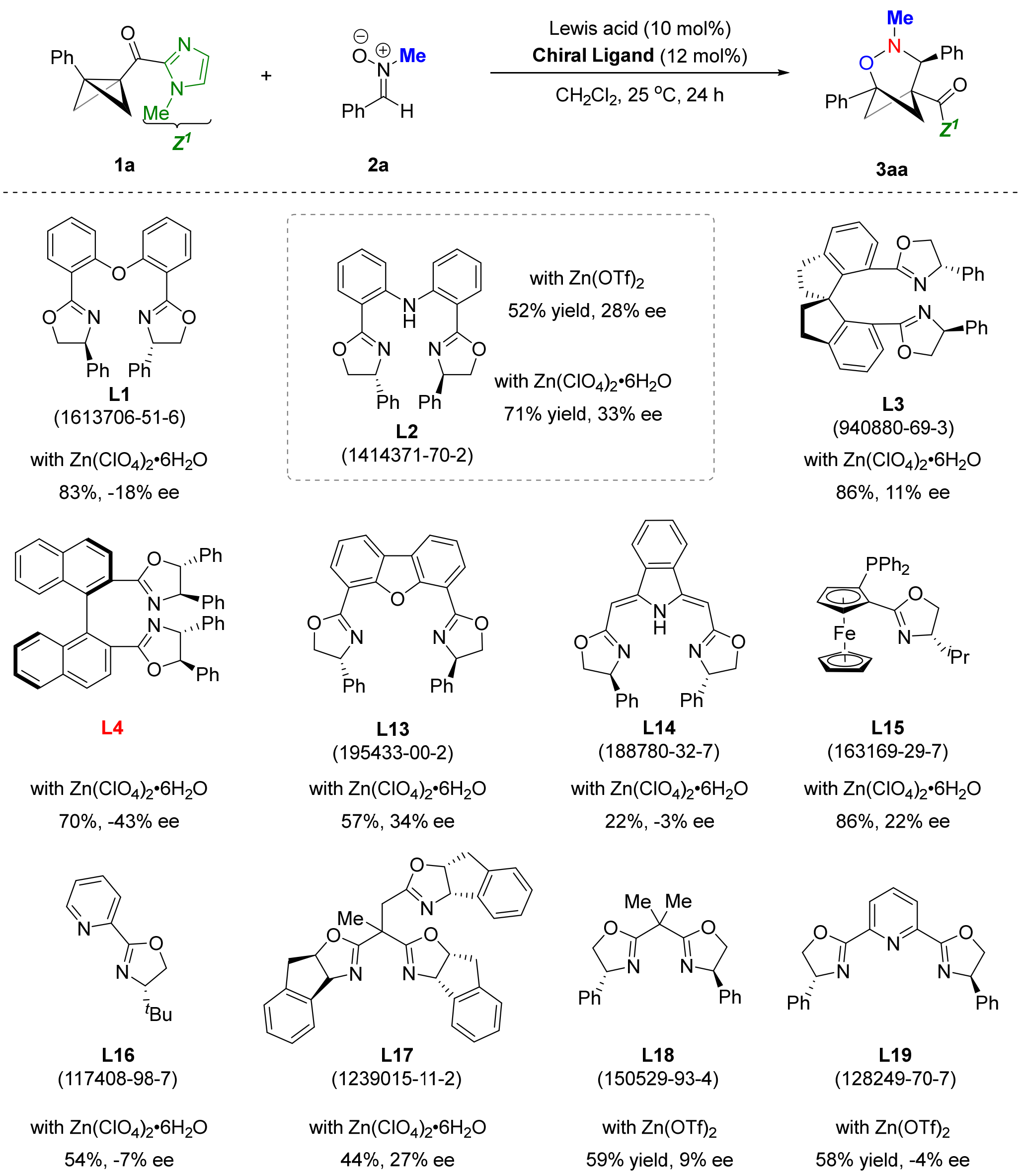

Supplementary Figure 1. Screening of chiral ligands for the enantioselective (3+3) cycloaddition of BCB **1a** and nitrone **2a**[a]. [a] Reaction conditions: **1a** (0.10 mmol, 1.0 equiv), **2a** (0.12 mmol, 1.2 equiv), Lewis acid (10 mol%) and Ligand (12 mol%), CH_2Cl_2 (2 mL), 25 °C, under N_2 for 24 h. The yields of **3aa** was determined by ^{1}H NMR with CH_2Br_2 as an internal standard. The *ee* value was determined by chiral HPLC with hexane/2-propanol. The CAS number of the ligand is in parentheses. [b] The ligands **L1-4** and **L13-19** were purchased from *Bide* Chemical Company and used as received.

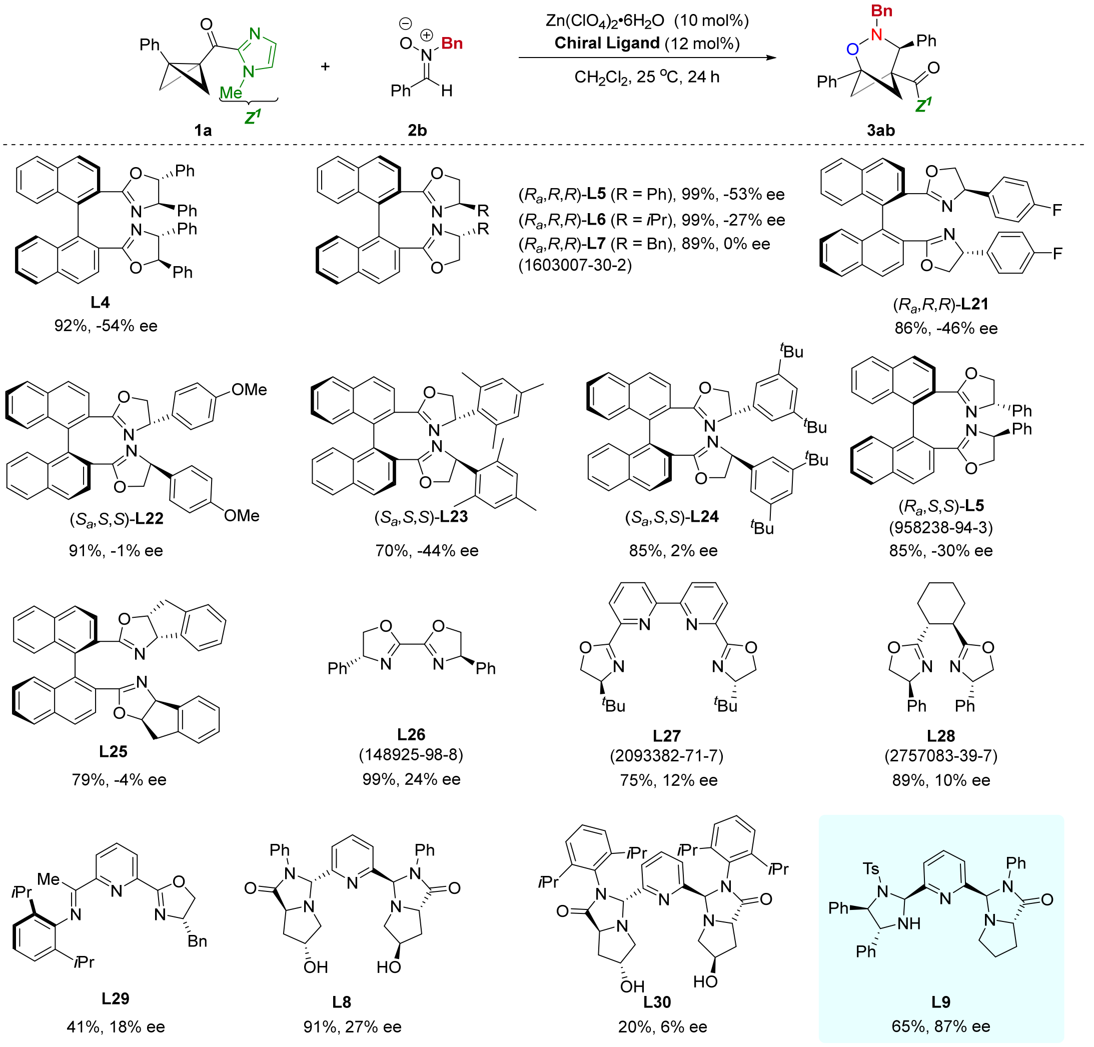

Supplementary Figure 2. Screening of chiral ligands for the enantioselective (3+3) cycloaddition of BCB **1a** and nitrone **2b**[a]. [a] Reaction conditions: **1a** (0.10 mmol, 1.0 equiv), **2b** (0.12 mmol, 1.2 equiv), $Zn(ClO_4)_2{\cdot}H_2O$ (10 mol%) and Ligand (12 mol%), CH_2Cl_2 (2 mL), 25 °C, under N_2 for 24 h. The yields of **3ab** was determined by ^{1}H NMR with CH_2Br_2 as an internal standard. The ee value was determined by chiral HPLC with hexane/2-propanol. The CAS number of the ligand is in parentheses. [b] The ligands **L4-9** and **L22-28** were purchased from *Bide* Chemical Company and used as received. The ligands **L29** was synthesized according to the literature[7]. The ligands **L8** and **L30** were synthesized according to the literature[8]. The ligands **L9-12** were synthesized according to the literature[9-10]

Supplementary Table 1. Screening of Lewis acid and solvent for the enantioselective (3+3) cycloaddition of BCB **1a** and nitrone **2b**[a]

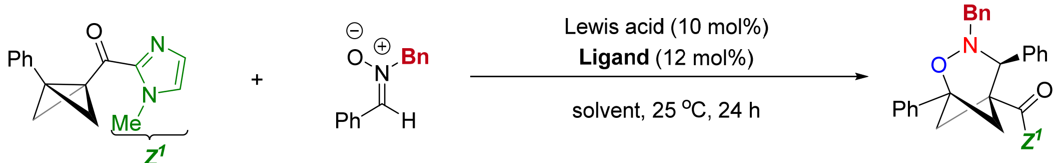

Entry	Lewis acid	Ligand	solvent	Yield (%)[b]	*ee* (%)[c]
1	$Eu(OTf)_3$	**L9**	CH_2Cl_2	90	2
2	$Ga(OTf)_3$	**L9**	CH_2Cl_2	99	0
3	$Sc(OTf)_3$	**L9**	CH_2Cl_2	66	17
4	$Zn(OTf)_2$	**L9**	CH_2Cl_2	51	90
5	$Ni(OTf)_2$	**L9**	CH_2Cl_2	95	80
6	$Cu(OTf)_2$	**L9**	CH_2Cl_2	89	65
7	$Fe(OTf)_3$	**L9**	CH_2Cl_2	84	14
8	$Mg(OTf)_2$	**L9**	CH_2Cl_2	45	23
9	$Co(OTf)_2$	**L9**	CH_2Cl_2	95	93
10	$Co(OTf)_2$	**L9**	EtOAc	96	92
11	$Co(OTf)_2$	**L9**	toluene	87	81
12	$Co(OTf)_2$	**L9**	THF	82	91
13	$Co(OTf)_2$	**L9**	1,4-dioxane	92	84
14	$Co(OTf)_2$	**L9**	DCE	93	91
15	$Co(OTf)_2$	**L9**	CH_3CN	60	59
16	$Co(OTf)_2$	**L9**	PhCl	85	94
17	$Co(OTf)_2$	**L10**	CH_2Cl_2	98	99
18[d]	$Co(OTf)_2$	**L10**	CH_2Cl_2	88	99
19[e]	$Co(OTf)_2$	**L10**	CH_2Cl_2	99	99
20	$Co(OTf)_2$	**L10**	$CHCl_3$	90	99
21	$Co(OTf)_2$	**L10**	CH_2Cl_2:HFIP (3:1, v/v)	15	96
22[f]	$Co(OTf)_2$	**L10**	CH_2Cl_2	99	97
23[g]	$Co(OTf)_2$	**L10**	CH_2Cl_2	75	97
24	$Co(OTf)_2$	-	CH_2Cl_2	99	-
25	-	**L10**	CH_2Cl_2	No reaction	
26	-	-	CH_2Cl_2	No reaction	
27	$Co(OTf)_2$	*ent*-**L10**	CH_2Cl_2	98	-99

[a] Reaction conditions: **1a** (0.10 mmol, 1.0 equiv), **2b** (0.12 mmol, 1.2 equiv), Lewis acid (10 mol%) and ligand (12 mol%), solvent (2 mL), 25 °C, under N_2 for 24 h. [b] Determined by ^1H NMR with CH_2Br_2 as an internal standard. [c] Determined by chiral HPLC with hexane/2-propanol. [d] At 0 °C. [e] At 40 °C. [f] 4 ÅMS (50 mg) was added. [g] H_2O (5 μL) was added.

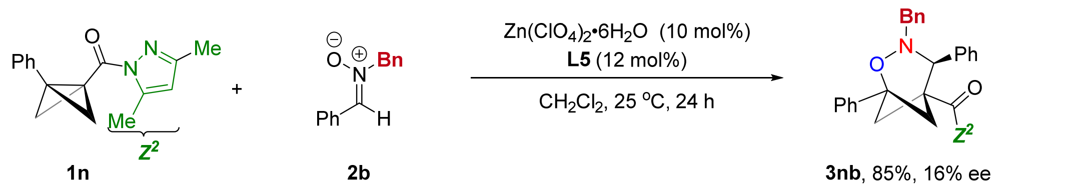

Supplementary Figure 3. Enantioselective cycloaddition of BCB **1n** and nitrone **2b**[a]. [a] Reaction conditions: **1n** (0.10 mmol, 1.0 equiv), **2b** (0.12 mmol, 1.2 equiv), $Zn(ClO_4)_2{\cdot}H_2O$ (10 mol%) and **L5** (12 mol%), CH_2Cl_2 (2 mL), 25 °C, under N_2 for 24 h. The yield of **3nb** was determined by ^{1}H NMR with CH_2Br_2 as an internal standard. The *ee* value was determined by chiral HPLC with hexane/2-propanol.

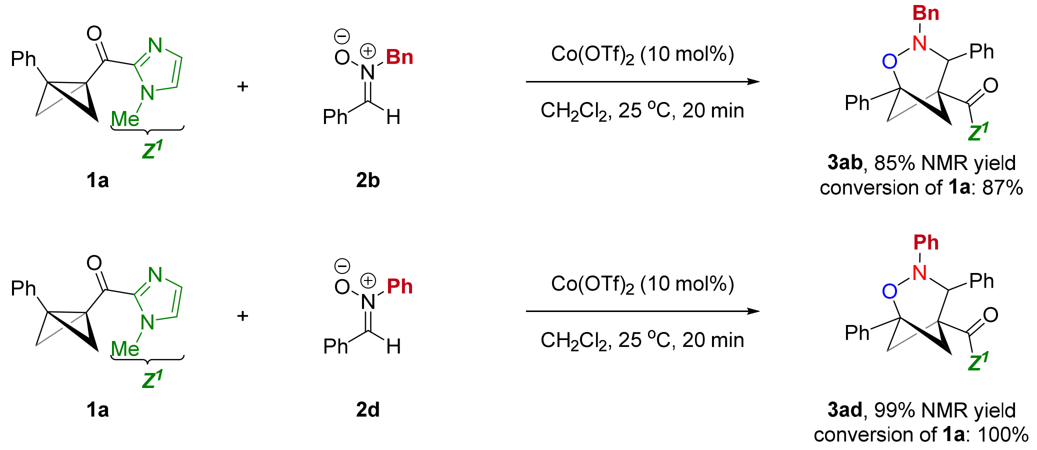

Supplementary Figure 4. Influence of R^1 substituents in nitrones on background reaction [a]. [a] Reaction conditions: **1a** (0.10 mmol, 1.0 equiv), **2b** or **2d** (0.12 mmol, 1.2 equiv), $Co(OTf)_2$ (10 mol%), CH_2Cl_2 (2 mL), 25 °C, under N_2 for 20 min. The yields of **3ab** or **3ad** were determined by ^{1}H NMR with CH_2Br_2 as an internal standard.

1.3 General Procedure for the Enantioselective (3+3) Cycloadditions (GP1)

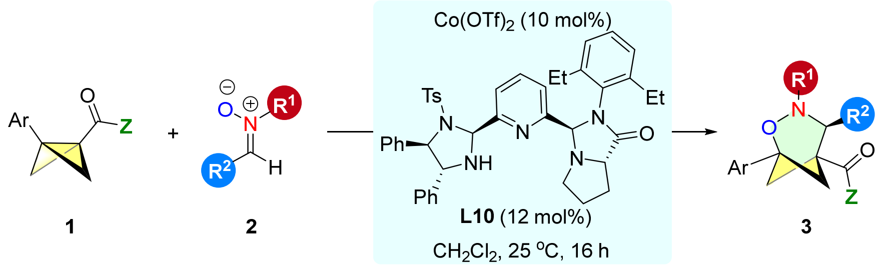

Under an atmosphere of N_2, to a 25 mL oven-dried Schlenk tube were added $Co(OTf)_2$ (7.1 mg, 0.020 mmol) and **L10** (17.1 mg, 0.024 mmol), followed by 2.0 mL of anhydrous CH_2Cl_2. The solution was stirred at 25 °C for 0.5 h, and then the BCBs **1** (0.20 mmol, 1.0 equiv) and nitrones **2** (0.24 mmol, 1.2 equiv) were added. Then the resulting mixture was stirred at room temperature for 16 h till full conversion of **1** by TLC analysis. After the solvent was removed under reduced pressure, the residue was directly subjected to a column chromatography purification using PE/EtOAc (4:1, v/v) as the eluent, to afford the desired product **3**.

1.4 General Procedure for the Synthesis of New Bicyclobutanes (GP2)

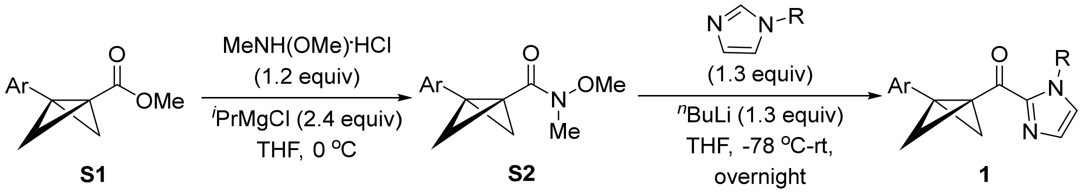

BCB esters **S1** were prepared according to literature procedures.[1-4] Weinreb amide derived BCBs **S2** were synthesized as following: An oven-dried 100 mL round bottom flask equipped with a stir bar was cooled under vacuum. After backfilled with N_2 (x 3) and capped with a septum, BCB esters **S1** (5.00 mmol, 1.0 equiv) and THF (25.0 mL) were added. The reaction was cooled to 0 °C. MeNH(OMe)·HCl (585.0 mg, 6.00 mmol, 1.2 equiv) and ^{i}PrMgCl (6.0 mL, 2.0 M in THF, 12.00 mmol, 2.4 equiv) were sequentially added to the solution. After stirred at the same temperature for 12 h, the reaction was quenched by saturated NH_4Cl solution (20 mL). The aqueous layer was extracted with EtOAc (3 x 10 mL). The combined organic layers were washed with brine (25 mL),

dried over anhydrous $MgSO_4$, filtered and concentrated by rotary evaporation. The crude Weinreb amide derived BCBs **S2** was directly used in next reaction.

An oven-dried 100 mL round bottom flask equipped with a stir bar was cooled under vacuum. After backfilled with N_2 (x 3) and capped with a septum, *N*-substituted imidazole derivatives (6.50 mmol, 1.3 equiv) and THF (20.0 mL) were added. The reaction was cooled to -78 °C. ^{n}BuLi (2.6 mL, 2.5 M in THF, 6.50 mmol, 1.3 equiv) was added to the solution. After stirred at the same temperature for 20 min, **S2** dissolved in THF (5.0 mL) was added. Next, the reaction was warm to room temperature and stirred overnight. Then, the reaction was quenched by saturated NH_4Cl solution (20 mL). The aqueous layer was extracted with EtOAc (3 x 10 mL). The combined organic layers were washed with brine (25 mL), dried over anhydrous $MgSO_4$, filtered and concentrated by rotary evaporation. The residue was purified by flash chromatography on silica gel (petroleum ether/EtOAc = 5/1) to afford a new kind of BCB **1**.

NOTE: the bicyclo[1.1.0]butanes (**1n-p**) equipped with an acyl pyrazole group were synthesized according to the literature[11-12].

1.5 Scale-Up Experiment

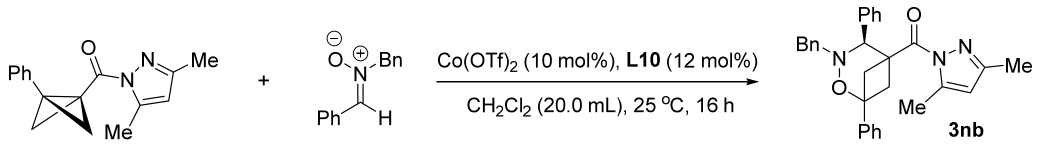

1n **2b** 99% yield, 99% ee

Under an atmosphere of N_2, to a 100 mL oven-dried Schlenk tube were added $Co(OTf)_2$ (35.7 mg, 0.10 mmol, 10 mol%) and **L10** (85.0 mg, 0.12 mmol, 12 mol%), followed by 20.0 mL of anhydrous CH_2Cl_2. The solution was stirred at 25 °C for 0.5 h. Then the BCB **1n** (252.0 mg, 1.00 mmol, 1.0 equiv) and nitrone **2b** (253.2 mg, 1.20 mmol, 1.2 equiv) were added. The resulting mixture was stirred at room temperature for 16 h till full conversion of **1n** by TLC analysis. After the solvent was removed under reduced pressure, the residue was directly subjected to a column chromatography

purification using PE/EtOAc (20:1, v/v) as the eluent, to afford the desired product **3nb** (458.4 mg, 99% yield, 99% ee).

1.6 Synthetic Transformations

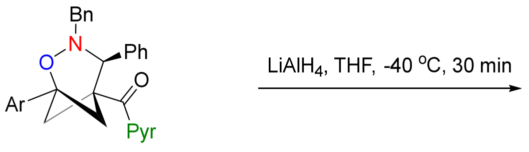

3 **4**

General Procedure for the conversion of 3nb or 3qb to aldehyde (GP3): The solution of $LiAlH_4$ (5.7 mg, 0.15 mmol, 1.5 equiv) in THF (2.0 mL) was cooled to -40 °C, and then a solution of **3** (0.10 mmol, 1.0 equiv) in THF (1.0 mL) was charged into the solution at -40 °C. After TLC analysis showed the starting material was consumed (30 min), HCl (aq. 1.0 M) was added at -40 °C and stirred for 15 min. The reaction mixture was directly filtered through celite, washed and extracted with EtOAc. The mixture was extracted with EtOAc (3 × 5 mL). Filtered and concentrated on rotavapor under reduced pressure. The residue was purification by flash chromatography on silica gel using petroleum ether/ethyl acetate (10/1) to afford **4** as a colorless oil.

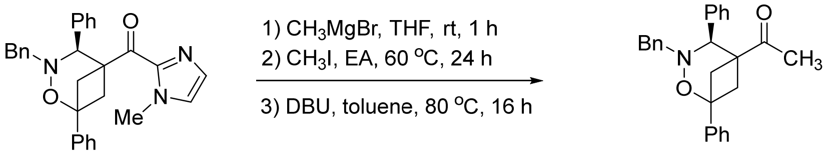

3ab **5,** 75% yield, 99% ee

Synthesis of (5): In a flame dried Schlenk flask, MeMgBr (3.0 M in Et_2O, 0.1 mL, 0.30 mmol, 3.0 equiv) was added portionwise to a solution of the **3ab** (44.9 mg, 0.10 mmol, 1.0 equiv) in dry THF (1.0 mL) at room temperature. The reaction was stirred for 1 hours, and monitored by TLC. Then aqueous saturated NH_4Cl solution (5 mL) was added to quench the reaction. The solution was extracted with ethyl acetate, washed with brine, dried over Na_2SO_4, filtered and concentrated on rotavapor under reduced pressure. The intermediate was dissolved with dry ethyl acetate (2.0 mL) and MeI (141.9 mg, 1.00 mmol, 10.0 equiv) was added. The reaction was heated at 60°C for 24 hours. And then, it was cooled down to room temperature and concentrated to

dryness. The residue was taken up in dry toluene (1.0 mL) and DBU (106.4 mg, 0.70 mmol, 7.0 equiv) was added. The mixture was heated at 80°C for 16 hours. The crude residue was purified by silica gel column chromatography (PE/EtOAc = 5:1) to afford **5** (28.7 mg, 75%, 99% ee) as a colorless oil.

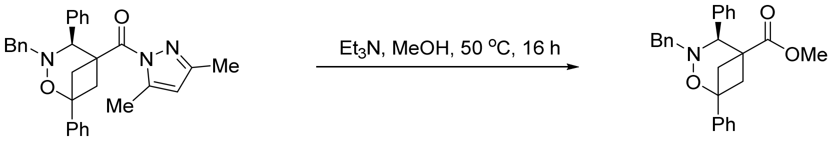

3nb **6**, 99% yield, 99% ee

Synthesis of (6): To a solution of **3nb** (46.3 mg, 0.10 mmol, 1.0 equiv) in MeOH (2.0 mL) was added Et_3N (30 μL, 0.40 mmol, 2.0 equiv). The mixture was then stirred at 50 °C for 16 hour, and monitored by TLC. After the solvent was removed under reduced pressure, the residue was directly subjected to a column chromatography purification (PE/EtOAc = 20:1) as the eluent to afford **6** (41.0 mg, 99% yield, 99% ee) as a colorless oil.

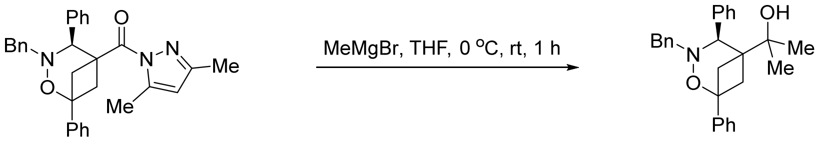

3nb **7,** 80% yield, 98% ee

Synthesis of (7): In a flame dried Schlenk flask, **3nb** (46.3 mg, 0.10 mmol, 1.0 equiv) was stirred in anhydrous THF (2.0 mL). The solution was cooled to 0 °C and MeMgBr (3.0 M in Et_2O, 0.1 mL, 0.30 mmol, 3.0 equiv) was added slowly. And then the reaction was stirred at room temperature for 1 hour. Finally, aqueous saturated NH_4Cl solution (2.0 mL) was added to quench the reaction. The mixture was extracted with EtOAc (3 × 5 mL). Filtered and concentrated on rotavapor under reduced pressure. The residue was purification by flash chromatography on silica gel using petroleum ether/ ethyl acetate (10/1) to afford **7** as a colorless oil (32.0 mg, 80% yield, 98% ee).

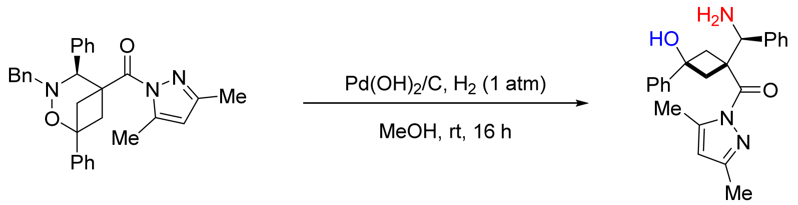

3nb **8**, 80% yield

Synthesis of (8): To a solution of **3nb** (23.2 mg, 0.05 mmol, 1.0 equiv) in MeOH (5.0 mL) was added $Pd(OH)_2/C$ (10 wt%) (5.0 mg). This flask was in a vacuum and back-filled with H_2 (1 atm). After being stirred at room temperature for 16 hour, the reaction solution was filtered, and the filtered-cake was washed with EtOAc (5.0 mL). The filtrate was evaporated under vacuo, and purified by silica gel column chromatography using petroleum ether/ethyl acetate (2/1) as the eluent to give the product **8** in 80% yield (15.0 mg). $[\alpha]_D^{20}$ = +32.7 (*c* = 0.30, $CHCl_3$). The enantiomeric excess could not be determined using HPLC analysis with chiral stationary phases in our laboratory.

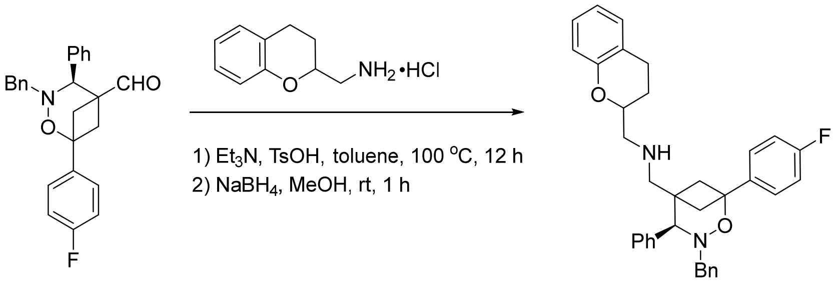

8 (+)-**9**, 93% yield

Synthesis of (9): To the solution of chroman-2-ylmethanamine hydrochloride (15.0 mg, 0.075 mmol) in toluene (1.0 mL) was added Et_3N (7.6 mg, 0.075 mmol), and the mixture was stirred at 35 °C for 30 min. **8** (16.0 mg, 0.04 mmol), 4 Å MS (50 mg) and $TsOH·H_2O$ (11.9 mg, 0.06 mmol) was added to the solution, and the mixture was stirred at 100 °C for 16 h. Then, the mixture was cooled to 0 °C. MeOH (1.0 mL) and $NaBH_4$ (2.3 mg, 0.06 mmol) was added to the solution, and the mixture was stirred for 1 h at room temperature. The resulting solution was quenched with H_2O and extracted with EtOAc (3 × 5 mL). Filtered and concentrated on rotavapor under reduced pressure. The residue was purification by flash chromatography on silica gel using petroleum ether/ethyl acetate (3/1) to afford **9** as a colorless oil (20.0 mg, 93% yield).

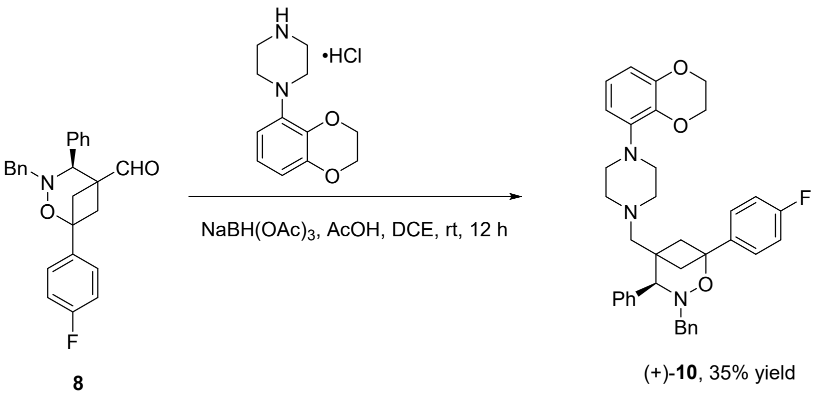

Synthesis of (10): **8** (19.4 mg, 0.05 mmol) and 1-(2,3-dihydrobenzo[b][1,4]dioxin-5-yl)piperazine hydrochloride (12.9 mg, 0.05 mmol) were mixed in 1,2-dichloroethane (2 mL) and treated with $NaBH(OAc)_3$ (15.9 mg, 0.075 mmol) and HOAc (20 μL) under an atmosphere of argon. The reaction mixture was stirred at room temperature for 12 h. The resulting solution was quenched with 2 N NaOH (2.0 mL) and extracted with EtOAc (3 × 5 mL). Filtered and concentrated on rotavapor under reduced pressure. The residue was purification by flash chromatography on silica gel using petroleum ether/ethyl acetate (10/1) to afford **10** as a colorless oil (10.4 mg, 35% yield).

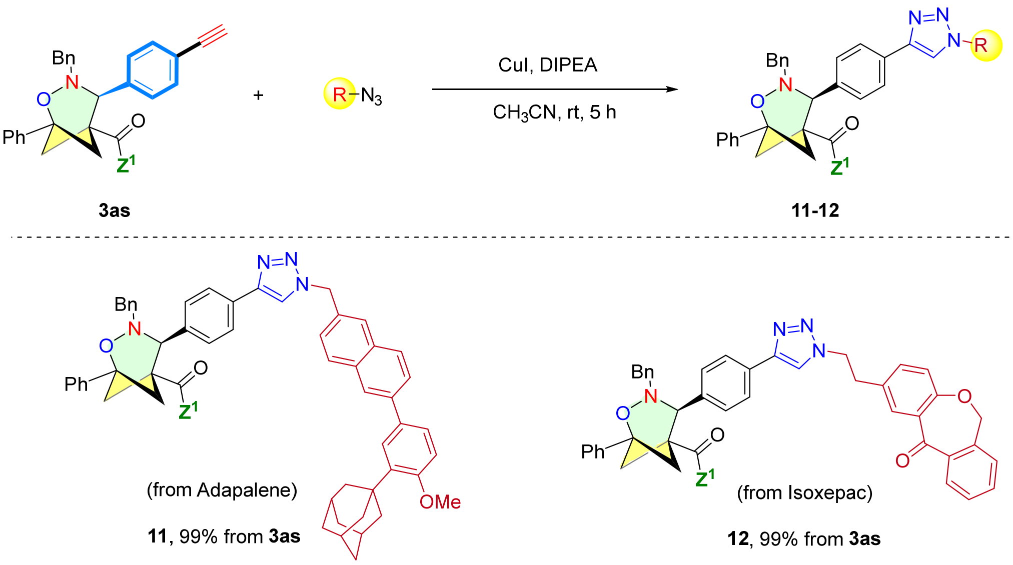

General Procedure for Copper-Catalyzed Alkyne–Azide Cycloaddition (GP4): A Schlenk tube is charged with **3as** (20.0 mg, 0.04 mmol, 1.0 equiv), CuI (0.8 mg, 0.004 mmol, 10 mol%), DIPEA (5.4 mg, 0.04 mmol, 1.0 equiv) and CH_3CN (1.0 mL). Then azide compounds (0.06 mmol, 1.5 equiv) was added. The reaction mixture is stirred at

room temperature for 5 h. The mixture is then concentrated under reduced pressure, and purified by silica gel column chromatography to give the product **11-12**.

1.7 General Procedure for the Synthesis of Chiral Ligands

1.7.1 The synthesis of L9-12 (GP5)

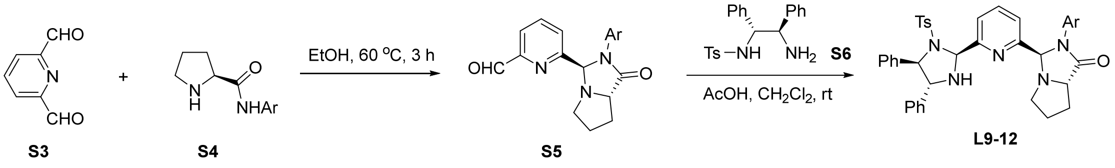

To the solution of pyridine-2,6-dicarbaldehyde (2.70 g, 20 mmol, 2.0 equiv) in anhydrous ethanol (40 mL) was added *L*-prolinamides (10 mmol, 1.0 equiv), and the reaction was heated and stirred at 60 °C for 3 h. After that, the reaction mixture was concentrated in vacuo to remove ethanol. The residue was purified by flash column chromatography (PE/EtOAc, 10/1-1/1, v/v) to give compounds **S5**.

In a round-bottomed flask containing a stir bar, compound **S5** (6.9 mmol, 1,0 equiv), **S6** (6.9 mmol, 1.0 equiv) AcOH (0.6 mL, 10.5 mmol, 1.5 equiv) and CH_2Cl_2 (70 mL) were added. Then, the reaction was stirred at room temperature for 6-8 h. After that, the reaction mixture was quenched by saturated $NaHCO_3$ solution (20 mL). The organic layer was extracted with CH_2Cl_2 (3 × 20 mL). Filtered and concentrated on rotavapor under reduced pressure. The resulting residue was purified by silica gel column chromatography to give ligands **L9-12**.

1.7.2 The synthesis of L21

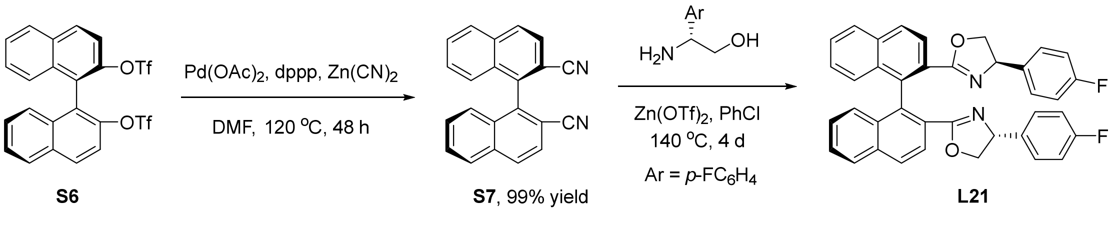

To the solution of BINOL-bistriflate **S6** (2.75 g, 5.0 mmol, 1.0 equiv) in DMF (20 mL) was added $Pd(OAc)_2$ (56.5 mg, 0.25 mmol, 5 mol%), dppp (162.0 mg, 0.5 mmol, 10 mol%) and $Zn(CN)_2$ (292.5 mg. 2.5 mmol, 5.0 equiv). Then the reaction was heated and stirred at 120 ^{o}C for 48 h. After the reaction was cooled to room temperature, the reaction mixture was diluted with ethyl acetate, and washed with H_2O and brine. The mixture was then dried over anhydrous Na_2SO_4, filtered, and concentrated under reduced pressure. The residue was further purified by flash chromatography (PE/EtOAc, 10/1, v/v) to give compounds **S7** (1.50 g, 99% yield).

To a 25 mL sealed tube were added $ZnCl_2$ (204.0 mg, 1.5 mmol, 5.0 equiv), **S7** (100.0 mg, 0.3 mmol, 1.0 equiv) and PhCl (3 mL). After the reaction mixture was stirred at room temperature for 10 min, (*R*)-2-amino-2-(4-fluorophenyl)ethan-1-ol (232.5 mg, 1.5 mmol, 5.0 equiv) was added to the tube. This tube was sealed and the reaction mixture was stirred at 120 ^{o}C for 4 days. The mixture was cooled to room temperature. Et_2NH (1 mL) was added to the tube, and the mixture was stirred at room temperature for 1.0 h. The residue was further purified by flash chromatography (PE/EtOAc, 4/1, v/v) to give ligand **L21** as a colorless oil (69.6 mg, 40% yield).

2. Supplementary Discussion

2.1 Unsuccessful BCB Substrates and Control Experiments

*The (3+3) reaction of **2n** with N-phenyl nitrone **2d** yields the desired **3nd** with 28% ee. In contrast, the cycloaddition of **1a** and **2d** produces **3ad** with 94% ee under identical reaction conditions. Unfortunately, the current reaction did not apply to methyl-substituted BCB substrate **1r** and mono-substituted BCB substrate **1t.** The ester-substituted BCB **1u** was used in the (3+3) cycloaddition but did not produce any cycloadduct, indicating that the chelation of bidentate group to Lewis acid catalyst is essential. In the absence of nitrone, the reaction of BCB 1k resulted in a 70% recovery of the starting material with no detectable cyclobutene, potentially excluding the presence of carbocation species.*

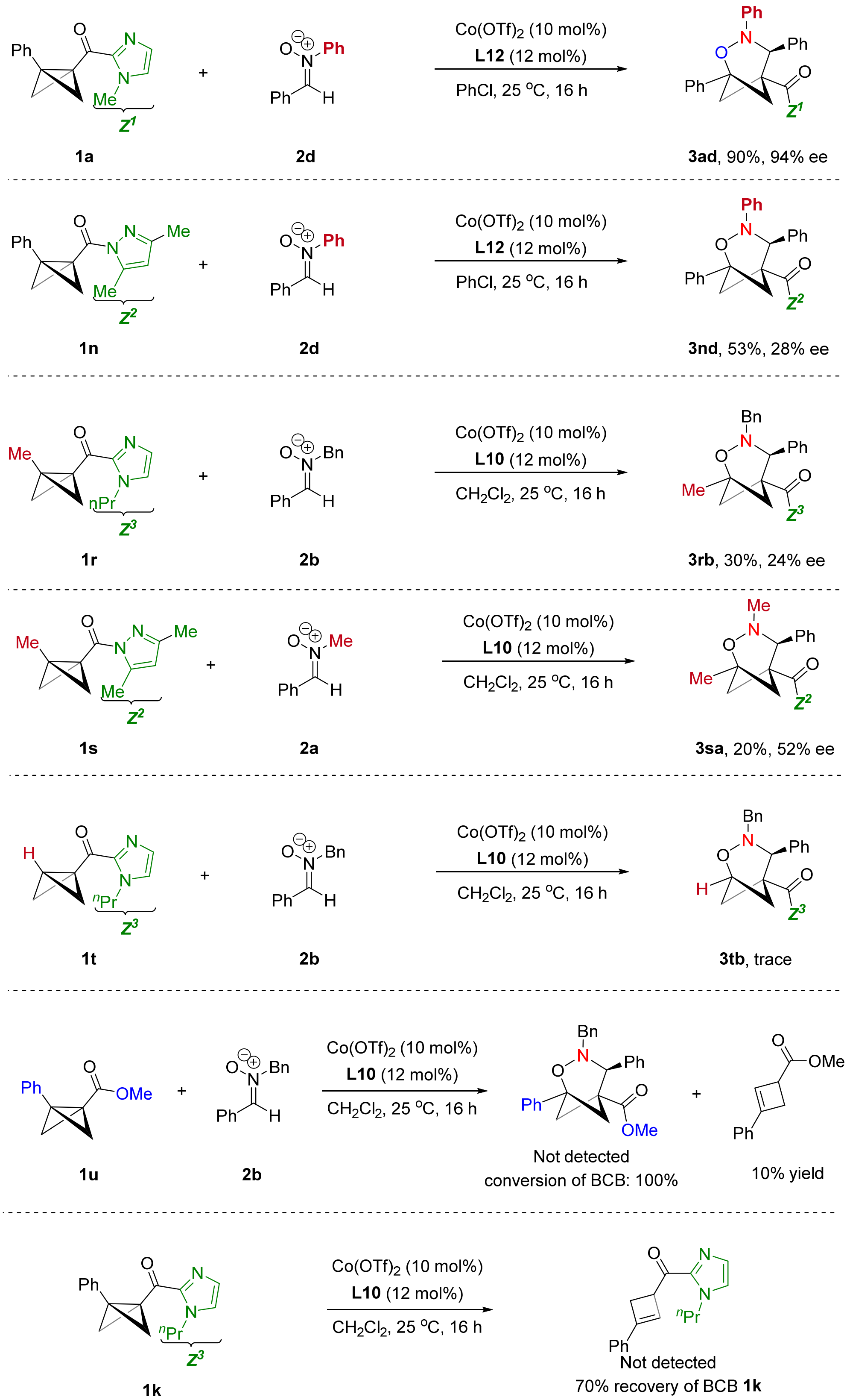

Supplementary Figure 5. Comparison of BCB with an acyl imidazole group and BCB with an acyl pyrazole moiety in stereocontrol and unsuccessful BCB substrates.

2.2 Non-Linear Effect Study

Supplementary Table 2. Tabulation of non-linear effects[a]

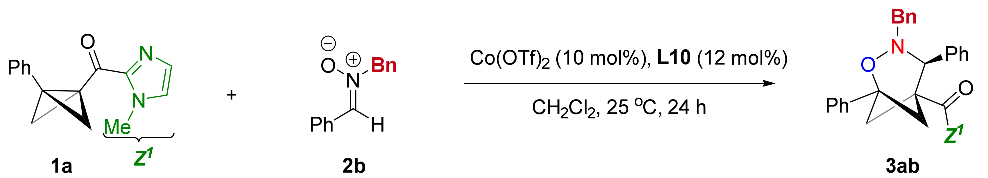

Entry	**L10** ee (%)[b]	Product **3ab** ee (%) [b]			
		1	2	3	average value
1	20	19.0	19.1	19.1	19.1
2	40	40.4	40.1	40.2	40.2
3	60	60.2	60.4	60.0	60.2
4	80	79.2	79.4	79.2	79.3
5	100	98.5	98.6	98.5	98.5

[a] Reaction conditions: **1a** (0.10 mmol, 1.0 equiv), **2b** (0.12 mmol, 1.2 equiv), $Co(OTf)_2$ (10 mol%) and **L10** (12 mol%), CH_2Cl_2 (2 mL), 25 °C, under N_2 for 24 h. [b] Determined by chiral HPLC with hexane/2-propanol.

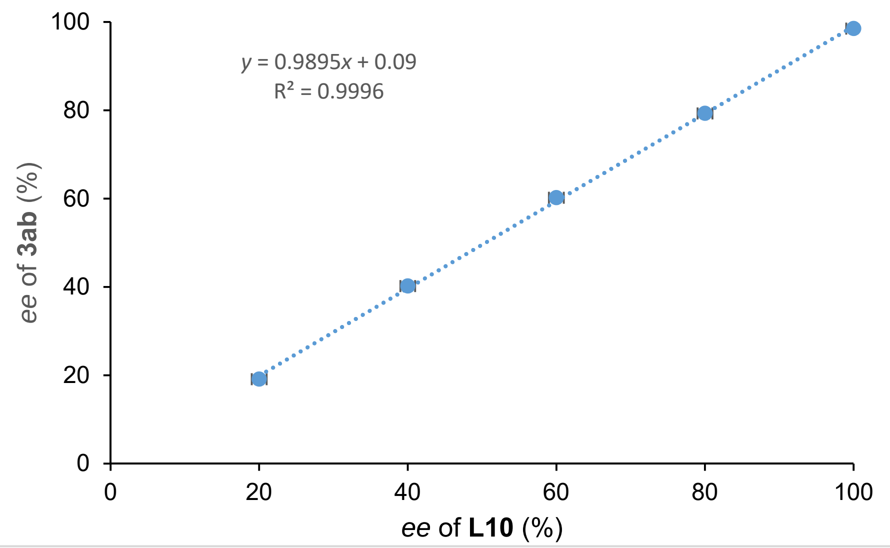

Supplementary Figure 6. Non-linear effect study.

2.3 Proposed Catalytic Cycle and Computed Transition Structures

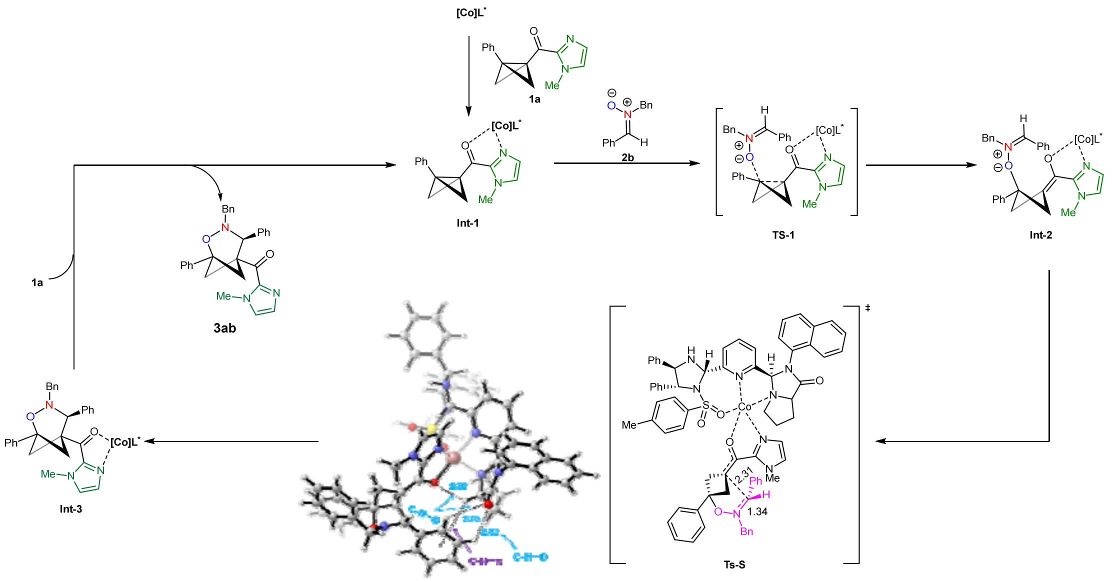

Supplementary Figure 7. Proposed catalytic cycle

To unravel the origin of enantiocontrol, density functional theory (DFT) calculations were performed at PBE0/6-31G(d)-SDD level of theory, using **1a** and **2b** as the model substrates along with the Co(II)−**L12** chiral system. As shown in Fig. 6 in the main text, transition states **Ts-S** and **Ts-R** leading to both products were located, in which divalent Co coordinated with two nitrogen and one oxygen atoms of **L12** as well as one oxygen and nitrogen atoms of **1a**, generating a square pyramidal geometry. The difference between the two activation barriers of the intramolecular nucleophilic cyclization for the two enantiomers was 2.2 kcal/mol, consistent with the excellent enantioselectivity experimentally observed. Moreover, noncovalent interactions between reactant fragment and **L12** in **Ts-S** and **Ts-R** were explored using independent gradient model based on Hirshfeld partition (IGMH) analysis. Three pairs of C–H•••O interaction existed in both **Ts-S** and **Ts-R**. Notably, in the favored transition state **Ts-S**, imidazolidone of **L12** engaged in C–H…π interaction with phenyl group of nitrone **2a**, likely dominating the preference for the nucleophilic attack of the enolate on the *Re*-face of nitrone **2a** to furnish the (*S*)-configuration of product **3ab**.

Based on the work of Deng[6], Xie, and Guo[5], as well as our DFT calculations, we have proposed a plausible catalytic cycle for the current (3+3) reaction. Initially, the

chiral Co(II)/**L12** catalyst coordinates with the acyl imidazole moiety, activating BCB **1a**. Subsequently, the concerted nucleophilic ring-opening pathway of **1a** with **2b** generates the key intermediate **int-2**. The carbanion is then generated in situ to attack the *Re* face of the C=N double bond in the nitrone via **Ts-S**, leading to the formation of **int-2**. Finally, ligand exchange releases the desired (*S*)-**3ab** and generates **int-1**.

2.4 Characterization Data of the New BCBs and Products

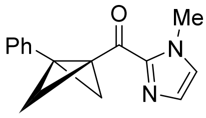

1a
$C_{15}H_{14}N_2O$
M = 238.29 g/mol

(1-methyl-1*H*-imidazol-2-yl)(3-phenylbicyclo[1.1.0]butan-1-yl)methanone: (**1a**) Prepared from methyl-3-phenylbicyclo[1.1.0]butane-1-carboxylate (1.88 g, 10.00 mmol, 1.0 equiv) according to the **GP2**. Purification by flash chromatography on silica gel afforded **1a** as a white solid (1.53 g, 64% yield over 2 steps).

R*f* = 0.35 (petroleum ether/EtOAc = 5/1). **^{1}H NMR** (400 MHz, $CDCl_3$): δ 7.28-7.26 (m, 2H), 7.24-7.16 (m, 3H), 7.07 (s, 1H), 6.85 (s, 1H), 3.63 (s, 2H), 3.60 (s, 3H), 1.90 (s, 2H) ppm. **^{13}C NMR** (100 MHz, $CDCl_3$): δ 185.7, 143.4, 133.3, 128.7, 128.2, 127.0, 126.0, 125.7, 40.4, 38.0, 35.7, 32.9 ppm. **HRMS** (ESI) *m/z*: $[M+H]^+$ calcd. for $C_{15}H_{15}N_2O$: 239.1179; found: 239.1179.

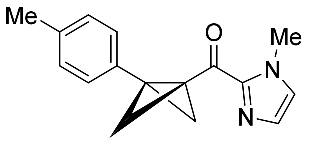

1b
$C_{16}H_{16}N_2O$
M = 252.32 g/mol

(1-methyl-1*H*-imidazol-2-yl)(3-(*p*-tolyl)bicyclo[1.1.0]butan-1-yl)methanone: (**1b**) Prepared from methyl 3-(*p*-tolyl)bicyclo[1.1.0]butane-1-carboxylate (0.94 g, 5.00 mmol, 1.0 equiv)

according to the **GP2**. Purification by flash chromatography on silica gel afforded **1b** as a white solid (0.76 g, 60% yield over 2 steps).

R*f* = 0.35 (petroleum ether/EtOAc = 5/1). **^{1}H NMR** (400 MHz, $CDCl_3$): δ 7.16 (d, *J* = 7.6 Hz, 2H), 7.07 (s, 1H), 7.03 (d, *J* = 7.6 Hz, 2H), 6.85 (s, 1H), 3.61 (s, 5H), 2.27 (s, 3H), 1.88 (s, 2H) ppm. **^{13}C NMR** (100 MHz, $CDCl_3$): δ 185.8, 143.4, 136.8, 130.0, 129.0, 128.6, 125.9, 125.7, 41.0, 38.0, 35.7, 32.8, 21.1 ppm. **HRMS** (ESI) *m/z*: $[M+H]^+$ calcd. for $C_{16}H_{17}N_2O$: 253.1335; found: 253.1334.

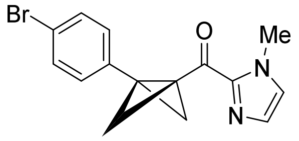

1c
$C_{15}H_{13}BrN_2O$
M = 317.19 g/mol

(3-(4-bromophenyl)bicyclo[1.1.0]butan-1-yl)(1-methyl-1*H*-imidazol-2-yl)methanone: (**1c**) Prepared from methyl 3-(4-bromophenyl)bicyclo[1.1.0]butane-1-carboxylate (1.34 g, 5.00 mmol, 1.0 equiv) according to the **GP2**. Purification by flash chromatography on silica gel afforded **1c** as a white solid (0.79 g, 50% yield over 2 steps).

R*f* = 0.30 (petroleum ether/EtOAc = 5/1). **^{1}H NMR** (400 MHz, $CDCl_3$): δ 7.33 (d, *J* = 8.4 Hz, 2H), 7.15 (d, *J* = 8.4 Hz, 2H), 7.07 (s, 1H), 6.89 (s, 1H), 3.69 (s, 3H), 3.62 (s, 2H), 1.88 (s, 2H) ppm. **^{13}C NMR** (100 MHz, $CDCl_3$): δ 185.1, 143.2, 132.7, 131.4, 128.8, 127.6, 126.0, 120.9, 39.4, 38.0, 36.0, 33.0 ppm. **HRMS** (ESI) *m/z*: $[M+H]^+$ calcd. for $C_{15}H_{14}BrN_2O$: 317.0284; found: 317.0282.

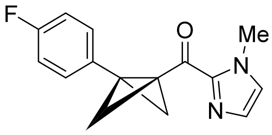

1d
$C_{15}H_{13}FN_2O$
M = 256.28 g/mol

(3-(4-fluorophenyl)bicyclo[1.1.0]butan-1-yl)(1-methyl-1H-imidazol-2-yl)methanone: (**1d**) Prepared from methyl 3-(4- fluorophenyl)bicyclo[1.1.0]butane-1-carboxylate (1.03 g,

5.00 mmol, 1.0 equiv) according to the **GP2**. Purification by flash chromatography on silica gel afforded **1d** as a white solid (0.64 g, 50% yield over 2 steps).

R*f* = 0.30 (petroleum ether/EtOAc = 5/1). **¹H NMR** (400 MHz, $CDCl_3$): δ 7.27-7.23 (m, 2H), 7.07 (s, 1H), 6.94-6.88 (m, 3H), 3.68 (s, 3H), 3.61 (s, 2H), 1.89 (s, 2H) ppm. **¹³C NMR** (100 MHz, $CDCl_3$): δ 185.4, 162.1 (d, *J* = 245 Hz), 143.4, 129.2 (d, *J* = 3 Hz), 128.9, 127.6 (d, *J* = 8 Hz), 125.9, 115.3 (d, *J* = 22 Hz), 39.7, 38.1, 35.9, 32.5 ppm. **¹⁹F NMR** (376 MHz, $CDCl_3$) δ -114.956 ppm. **HRMS** (ESI) *m/z*: $[M+NH_4]^+$ calcd. for $C_{15}H_{17}N_3O$: 274.1350; found: 274.1358.

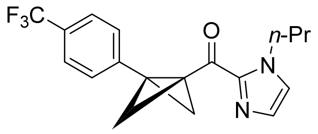

1e
$C_{18}H_{17}F_3N_2O$
M = 334.13 g/mol

(1-propyl-1H-imidazol-2-yl)(3-(4-(trifluoromethyl)phenyl)bicyclo[1.1.0]butan-1-yl) methanone: (**1e**) Prepared from methyl 3-(4-(trifluoromethyl)phenyl) bicycle [1.1.0] butane-1-carboxylate (1.28 g, 5.00 mmol, 1.0 equiv) according to the **GP2**. Purification by flash chromatography on silica gel afforded **1e** as a white solid (0.71 g, 52% yield over 2 steps).

R*f* = 0.36 (petroleum ether/EtOAc = 5/1). **¹H NMR** (400 MHz, $CDCl_3$): δ 7.45 (d, *J* = 8.0 Hz, 2H), 7.35 (d, *J* = 8.0 Hz, 2H), 7.09 (s, 1H), 6.92 (s, 1H), 4.07 (t, *J* = 7.2 Hz, 2H), 3.68 (s, 2H), 1.95 (s, 2H), 1.19 (q, *J* = 7.2 Hz, 2H), 0.53 (t, *J* = 7.6 Hz, 3H) ppm. **¹³C NMR** (100 MHz, $CDCl_3$): δ 184.8, 142.4, 138.1, 128.8 (q, *J* = 30 Hz), 125.7, 125.4, 125.2 (q, *J* = 4 Hz), 122.7, 123.4 (q, *J* = 70 Hz), 50.0, 38.2, 38.1, 33.8, 24.1, 10.5 ppm. **¹⁹F NMR** (376 MHz, $CDCl_3$) δ -62.539 ppm. **HRMS** (ESI) *m/z*: $[M+H]^+$ calcd. for $C_{18}H_{18}F_3N_2O$: 335.1366; found: 335.1376.

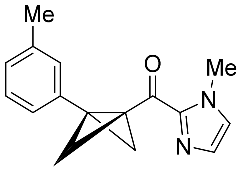

1f
$C_{16}H_{16}N_2O$
M = 252.32 g/mol

(1-methyl-1*H*-imidazol-2-yl)(3-(*m*-tolyl)bicyclo[1.1.0]butan-1-yl)methanone: (**1f**) Prepared from methyl 3-(*m*-tolyl)bicyclo[1.1.0]butane-1-carboxylate (1.01 g, 5.00 mmol, 1.0 equiv) according to the **GP2**. Purification by flash chromatography on silica gel afforded **1f** as a white solid (0.50 g, 40% yield over 2 steps).

R*f* = 0.25 (petroleum ether/EtOAc = 5/1). **^{1}H NMR** (400 MHz, $CDCl_3$): δ 7.11-7.04 (m, 4H), 6.98 (d, *J* = 6.8 Hz, 1H), 6.84 (s, 1H), 3.61 (s, 3H), 3.60 (s, 2H), 2.25 (s, 3H), 1.87 (s, 2H) ppm. **^{13}C NMR** (100 MHz, $CDCl_3$): δ 185.8, 143.4, 137.8, 133.1, 128.6, 128.1, 127.8, 126.8, 125.6, 123.0, 40.6, 38.0, 35.7, 32.8, 21.3 ppm. **HRMS** (ESI) *m/z*: $[M+H]^+$ calcd. for $C_{16}H_{17}N_2O$: 253.1335; found: 253.1334.

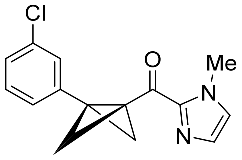

1g
$C_{15}H_{13}ClN_2O$
M = 272.73 g/mol

(3-(3-chlorophenyl)bicyclo[1.1.0]butan-1-yl)(1-methyl-1*H*-imidazol-2-yl)methanone: (**1g**) Prepared from methyl 3-(3-chlorophenyl)bicyclo[1.1.0]butane-1-carboxylate (1.11 g, 5.00 mmol, 1.0 equiv) according to the **GP2**. Purification by flash chromatography on silica gel afforded **1g** as a white solid (0.59 g, 43% yield over 2 steps).

R*f* = 0.35 (petroleum ether/EtOAc = 5/1). **^{1}H NMR** (400 MHz, $CDCl_3$): δ 7.25 (s, 1H), 7.16-7.13 (m, 3H), 7.09 (s, 1H), 6.89 (s, 1H), 3.69 (s, 3H), 3.61 (s, 2H), 1.88 (s, 2H) ppm. **^{13}C NMR** (100 MHz, $CDCl_3$): δ 185.1, 143.2, 135.8, 134.2, 129.4, 128.8, 127.0, 126.2, 126.0, 124.2, 38.9, 38.0, 35.9, 33.0 ppm. **HRMS** (ESI) *m/z*: $[M+H]^+$ calcd. for $C_{15}H_{14}ClN_2O$: 273.0789; found: 273.0787.

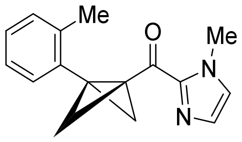

1h
$C_{16}H_{16}N_2O$
M = 252.32 g/mol

(1-methyl-1*H*-imidazol-2-yl)(3-(*o*-tolyl)bicyclo[1.1.0]butan-1-yl)methanone: (**1h**) Prepared from methyl methyl 3-(*o*-tolyl)bicyclo[1.1.0]butane-1-carboxylate (0.61 g, 3.00 mmol, 1.0 equiv) according to the **GP2**. Purification by flash chromatography on silica gel afforded **1h** as a white solid (0.45 g, 59% yield over 2 steps).

R*f* = 0.35 (petroleum ether/EtOAc = 5/1). **^{1}H NMR** (400 MHz, $CDCl_3$): δ 7.12-7.05 (m, 3H), 7.01-6.96 (m, 2H), 6.93 (s, 1H), 3.85 (s, 3H), 3.35 (s, 2H), 2.44 (s, 3H), 1.94 (s, 2H) ppm. **^{13}C NMR** (100 MHz, $CDCl_3$): δ 186.8, 143.7, 138.7, 132.3, 130.7, 128.8, 127.1, 126.0, 125.8, 125.6, 41.3, 38.6, 36.1, 30.3, 20.4 ppm. **HRMS** (ESI) *m/z*: $[M+H]^+$ calcd. for $C_{16}H_{17}N_2O$: 253.1335; found: 253.1335.

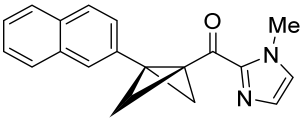

1i
$C_{19}H_{16}N_2O$
M = 288.35 g/mol

(1-methyl-1*H*-imidazol-2-yl)(3-(naphthalen-2-yl)bicyclo[1.1.0]butan-1-yl)methanone: (**1i**) Prepared from methyl 3-(naphthalen-2-yl)bicyclo[1.1.0]butane-1-carboxylate (1.19 g, 5.00 mmol, 1.0 equiv) according to the **GP2**. Purification by flash chromatography on silica gel afforded **1i** as a white solid (0.72 g, 50% yield over 2 steps).

R*f* = 0.30 (petroleum ether/EtOAc = 5/1). **^{1}H NMR** (400 MHz, $CDCl_3$): δ 7.77 (s, 1H), 7.73 (d, *J* = 8.0 Hz, 2H), 7.65 (d, *J* = 8.4 Hz, 1H), 7.44-7.36 (m, 2H), 7.33 (d, *J* = 8.4 Hz, 1H), 7.07 (s, 1H), 6.79 (s, 1H), 3.77 (s, 2H), 3.53 (s, 3H), 1.96 (s, 2H) ppm. **^{13}C NMR** (100 MHz, $CDCl_3$): δ 185.4, 143.4, 133.2, 132.4, 131.1, 128.7, 127.8, 127.6, 127.5, 126.2, 125.8, 125.7, 125.6, 123.5, 40.8, 38.2, 35.8, 33.2 ppm. **HRMS** (ESI) *m/z*: $[M+H]^+$ calcd. for $C_{19}H_{17}N_2O$: 289.1335; found: 289.1331.

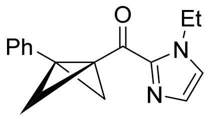

1j
$C_{16}H_{16}N_2O$
M = 252.13 g/mol

(1-ethyl-1H-imidazol-2-yl)(3-phenylbicyclo[1.1.0]butan-1-yl)methanone: (**1j**) Prepared from methyl-3-phenylbicyclo[1.1.0]butane-1-carboxylate (1.88 g, 10.00 mmol, 1.0 equiv) according to the **GP2**. Purification by flash chromatography on silica gel afforded **1j** as a white solid (1.35 g, 62% yield over 2 steps).

R_f = 0.35 (petroleum ether/EtOAc = 5/1). **^{1}H NMR** (400 MHz, $CDCl_3$): δ 7.28-7.24 (m, 2H), 7.20 (t, J = 7.2 Hz, 2H), 7.16-7.12 (m, 1H), 7.08 (s, 1H), 6.91 (s, 1H), 4.06 (q, J = 7.2 Hz, 2H), 3.63 (s, 2H), 1.91 (s, 2H), 0.94 (t, J = 7.2 Hz, 3H) ppm. **^{13}C NMR** (100 MHz, $CDCl_3$): δ 185.5, 142.5, 133.3, 128.8, 128.2, 126.9, 125.8, 124.1, 43.3, 40.1, 37.9, 33.0, 16.1 ppm. **HRMS** (ESI) *m/z*: $[M+H]^+$ calcd. for $C_{16}H_{17}N_2O$: 253.1335; found: 253.1332.

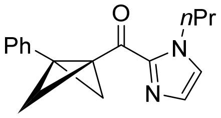

1k
$C_{17}H_{18}N_2O$
M = 266.34 g/mol

(3-phenylbicyclo[1.1.0]butan-1-yl)(1-propyl-1H-imidazol-2-yl)methanone: (**1k**) Prepared from methyl-3-phenylbicyclo[1.1.0]butane-1-carboxylate (1.88 g, 10.00 mmol, 1.0 equiv) according to the **GP2**. Purification by flash chromatography on silica gel afforded **1k** as a white solid (1.47 g, 55% yield over 2 steps).

R_f = 0.30 (petroleum ether/EtOAc = 5/1). **^{1}H NMR** (400 MHz, $CDCl_3$): δ 7.27-7.15 (m, 5H), 7.09 (s, 1H), 6.90 (s, 1H), 4.02 (t, J = 7.2 Hz, 2H), 3.62 (s, 2H), 1.93 (s, 2H), 1.22-1.17 (m, 2H), 0.54 (t, J = 7.2 Hz, 3H) ppm. **^{13}C NMR** (100 MHz, $CDCl_3$): δ 185.6, 142.6, 133.2, 128.6, 128.3, 126.9, 125.8, 125.0, 49.9, 40.2, 37.9, 33.1, 24.1, 10.7 ppm. **HRMS** (ESI) *m/z*: $[M+H]^+$ calcd. for $C_{17}H_{19}N_2O$: 267.1492; found: 267.1487.

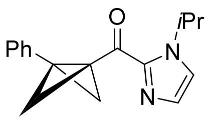

1l
$C_{17}H_{18}N_2O$
M = 266.34 g/mol

(1-isopropyl-1H-imidazol-2-yl)(3-phenylbicyclo[1.1.0]butan-1-yl)methanone: (**1l**) Prepared from methyl-3-phenylbicyclo[1.1.0]butane-1-carboxylate (1.88 g, 10.00 mmol, 1.0 equiv) according to the **GP2**. Purification by flash chromatography on silica gel afforded **1l** as a white solid (1.36 g, 51% yield over 2 steps).

R*f* = 0.35 (petroleum ether/EtOAc = 5/1). **^{1}H NMR** (400 MHz, $CDCl_3$): δ 7.27-7.15 (m, 5H), 7.11 (s, 1H), 7.08 (s, 1H), 5.03-4.97 (m, 1H), 3.59 (s, 2H), 1.93 (s, 2H), 1.07 (d, *J* = 6.8 Hz, 6H) ppm. **^{13}C NMR** (100 MHz, $CDCl_3$): δ 186.1, 142.5, 133.3, 129.1, 128.3, 126.9, 125.8, 119.8, 48.6, 40.1, 38.0, 33.4, 23.3 ppm. **HRMS** (ESI) *m/z*: $[M+H]^+$ calcd. for $C_{17}H_{19}N_2O$: 267.1492; found: 267.1488.

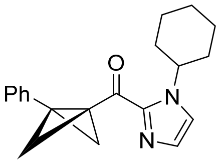

1m
$C_{20}H_{22}N_2O$
M = 306.17 g/mol

(1-cyclohexyl-1H-imidazol-2-yl)(3-phenylbicyclo[1.1.0]butan-1-yl)methanone: (**1m**) Prepared from methyl-3-phenylbicyclo[1.1.0]butane-1-carboxylate (1.88 g, 10.00 mmol, 1.0 equiv) according to the **GP2**. Purification by flash chromatography on silica gel afforded **1m** as a white solid (1.10 g, 52% yield over 2 steps).

R*f* = 0.35 (petroleum ether/EtOAc = 5/1). **^{1}H NMR** (400 MHz, $CDCl_3$): δ 7.27-7.14 (m, 5H), 7.09 (s, 1H), 7.06 (s, 1H), 4.55-4.49 (m, 1H), 3.57 (s, 2H), 1.93 (s, 2H), 1.67-1.63 (m, 3H), 1.51-1.49 (m, 2H), 1.26-1.15 (m, 4H), 1.09-1.02 (m, 1H) ppm. **^{13}C NMR** (100 MHz, $CDCl_3$): δ 186.2, 142.6, 133.3, 129.0, 128.2, 126.8, 125.8, 122.2, 56.0, 40.1, 37.9, 34.1, 33.6, 25.5, 25.2 ppm. **HRMS** (ESI) *m/z*: $[M+H]^+$ calcd. for $C_{20}H_{23}N_2O$: 307.1805; found: 307.1801.

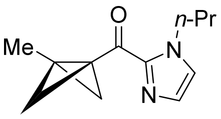

1q
$C_{12}H_{16}N_2O$
M = 204.13 g/mol

(3-methylbicyclo[1.1.0]butan-1-yl)(1-propyl-1H-imidazol-2-yl)methanone: (1q) Prepared from benzyl 3-methylbicyclo[1.1.0]butane-1-carboxylate (2.02 g, 10.00 mmol, 1.0 equiv) according to the **GP2**. Purification by flash chromatography on silica gel afforded **1q** as a colorless oil (1.02 g, 50% yield over 2 steps).

R*f* = 0.40 (petroleum ether/EtOAc = 5/1). **^{1}H NMR** (400 MHz, $CDCl_3$): δ 7.10 (s, 1H), 7.02 (s, 1H), 4.35 (t, *J* = 7.2 Hz, 2H), 2.89 (s, 2H), 1.84-1.74 (m, 2H), 1.57 (s, 3H), 1.53 (s, 2H), 0.91 (t, *J* = 7.2 Hz, 3H) ppm. **^{13}C NMR** (100 MHz, $CDCl_3$): δ 188.2, 143.2, 128.7, 124.8, 50.3, 41.1, 36.0, 24.7, 23.1, 12.9, 11.0 ppm. **HRMS** (ESI) *m/z*: $[M+H]^+$ calcd. for $C_{12}H_{17}N_2O$: 205.1335; found: 205.1333.

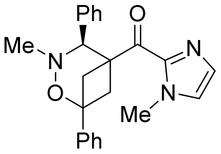

3aa
$C_{23}H_{23}N_3O_2$
M = 373.46 g/mol

(*S*)-(3-methyl-1,4-diphenyl-2-oxa-3-azabicyclo[3.1.1]heptan-5-yl)(1-methyl-1H-imidazol-2-yl)methanone: (**3aa**) Prepared from ((1-methyl-1H-imidazol-2-yl)(3-phenylbicyclo[1.1.0]butan-1-yl)methanone (**1a**, 47.6 mg, 0.20 mmol) and (*Z*)-*N*-methyl-1-phenylmethanimine oxide (**2a**, 32.4 mg, 0.24 mmol) according to the **GP1** at rt for 16 h. Purification by flash chromatography on silica gel using petroleum ether/EtOAc (5/1) afforded **3aa** as a white solid (64.2 mg, 86% yield).

3aa: **R*f*** = 0.4 (petroleum ether/EtOAc = 3/1). Mp: 107-109 °C. HPLC analysis (Chiralpak AD-H, ^{i}PrOH/hexane = 10/90, 1.0 mL/min, 254 nm; tr (major) = 10.89 min, tr (minor) = 15.61 min) gave the isomeric composition of the product: 99% ee. $[\alpha]_D^{20}$ = +66.8 (*c* = 1.50, $CHCl_3$). **^{1}H NMR** (400 MHz, $CDCl_3$): δ 7.44 (d, *J* = 6.8 Hz, 2H), 7.39-

7.36 (m, 2H), 7.32-7.28 (m, 1H), 7.20 (s, 1H), 7.15-7.11 (m, 3H), 7.09-7.07 (m, 2H), 6.87 (s, 1H), 4.58 (s, 1H), 3.61 (t, *J* = 8.6 Hz, 1H), 3.40 (s, 3H), 2.90-2.84 (m, 2H), 2.62 (s, 3H), 2.47 (d, *J* = 10.4 Hz, 1H) ppm. **^{13}C NMR** (100 MHz, $CDCl_3$): δ 191.55, 142.6, 141.1, 138.1, 129.1, 128.3, 128.1, 127.9, 127.8, 125.9, 125.9, 81.9, 77.3, 53.8, 49.9, 43.3, 35.1, 33.5 ppm. **HRMS** (ESI) *m/z*: $[M+H]^+$ calcd. for $C_{23}H_{24}N_3O_2$: 374.1863; found: 374.1856.

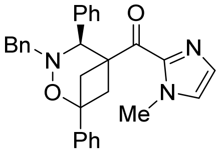

3ab
$C_{29}H_{27}N_3O_2$
M = 449.55 g/mol

(*S*)-(3-benzyl-1,4-diphenyl-2-oxa-3-azabicyclo[3.1.1]heptan-5-yl)(1-methyl-1H-imidazol-2-yl)methanone: (**3ab**) Prepared from ((1-methyl-1H-imidazol-2-yl)(3-phenylbicyclo[1.1.0]butan-1-yl)methanone (**1a**, 47.6 mg, 0.20 mmol) and (*Z*)-*N*-benzyl-1-phenylmethanimine oxide (**2b**, 50.6 mg, 0.24 mmol) according to the **GP1** at rt for 16 h. Purification by flash chromatography on silica gel using petroleum ether/EtOAc (5/1) afforded **3ab** as a white solid (72.4 mg, 85% yield).

3ab: **R*f*** = 0.4 (petroleum ether/EtOAc = 3/1). Mp: 113-115 °C. HPLC analysis (Chiralpak AD-H, ^{i}PrOH/hexane = 10/90, 1.0 mL/min, 254 nm; tr (minor) = 15.00 min, tr (major) = 19.23 min) gave the isomeric composition of the product: 99% ee. $[\alpha]_D^{20}$ = +79.3 (*c* = 0.85, $CHCl_3$). **^{1}H NMR** (400 MHz, $CDCl_3$): δ 7.37 (d, *J* = 7.2 Hz, 2H), 7.30-7.24 (m, 9H), 7.14 (s, 5H), 6.87 (s, 1H), 4.85 (s, 1H), 3.99 (AB, *J* = 15.2 Hz, 1H), 3.86 (AB, *J* = 14.8 Hz, 1H), 3.65 (t, *J* = 9.4 Hz, 1H), 3.41 (s, 3H), 2.93 (d, *J* = 9.6 Hz, 1H), 2.81 (t, *J* = 9.2 Hz, 1H), 2.42 (d, *J* = 10.0 Hz, 1H) ppm. **^{13}C NMR** (100 MHz, $CDCl_3$): δ 191.6, 142.6, 141.3, 138.2, 138.1, 129.1, 128.6, 128.2, 128.11, 128.07, 127.9, 127.8, 127.5, 126.6, 125.9, 125.4, 81.7, 74.4, 58.5, 54.1, 49.7, 35.1, 35.0 ppm. **HRMS** (ESI) *m/z*: $[M+H]^+$ calcd. for $C_{29}H_{28}N_3O_2$: 450.2166; found: 450.2166.

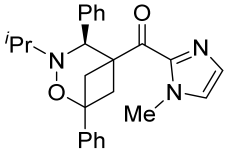

3ac
$C_{25}H_{27}N_3O_2$
M = 401.51 g/mol

(*S*)-(3-isopropyl-1,4-diphenyl-2-oxa-3-azabicyclo[3.1.1]heptan-5-yl)(1-methyl-1H-imidazol-2-yl)methanone: (**3ac**) Prepared from ((1-methyl-1H-imidazol-2-yl)(3-phenylbicyclo[1.1.0]butan-1-yl)methanone (**1a**, 47.6 mg, 0.20 mmol) and (*Z*)-*N*-isopropyl-1-phenylmethanimine oxide (**2c**, 39.2 mg, 0.24 mmol) according to the **GP1** at rt for 16 h. Purification by flash chromatography on silica gel using petroleum ether/EtOAc (5/1) afforded **3ac** as a white solid (64.2 mg, 80% yield).

3ac: **R*f*** = 0.4 (petroleum ether/EtOAc = 3/1). Mp: 143-145 °C. HPLC analysis (Chiralpak AD-H, ^{i}PrOH/hexane = 5/95, 1.0 mL/min, 254 nm; tr (minor) = 6.79 min, tr (major) = 8.15 min) gave the isomeric composition of the product: 99% ee. $[\alpha]_D^{20}$ = +37.2 (*c* = 2.00, $CHCl_3$). **^{1}H NMR** (400 MHz, $CDCl_3$): δ 7.44 (d, *J* = 8.0 Hz, 2H), 7.36 (t, *J* = 7.4 Hz, 2H), 7.30-7.26 (m, 1H), 7.20 (s, 1H), 7.14-7.05 (m, 5H), 6.86 (s, 1H), 4.98 (s, 1H), 3.57 (t, *J* = 9.4 Hz, 1H), 3.38 (s, 3H), 2.94-2.90 (m, 1H), 2.87 (d, *J* = 9.6 Hz, 1H), 2.74 (t, *J* = 9.0 Hz, 1H), 2.37 (d, *J* = 10.0 Hz, 1H), 1.21 (d, *J* = 6.8 Hz, 3H), 1.14 (d, *J* = 6.4 Hz, 3H) ppm. **^{13}C NMR** (100 MHz, $CDCl_3$): δ 192.1, 142.78, 142.0, 138.8, 129.0, 128.2, 127.9, 127.74, 127.71, 127.5, 125.7, 125.4, 80.8, 70.1, 53.9, 51.5, 49.3, 35.1, 34.4, 21.2, 14.0 ppm. **HRMS** (ESI) *m/z*: $[M+H]^+$ calcd. for $C_{25}H_{28}N_3O_2$: 402.2176; found: 402.2176.

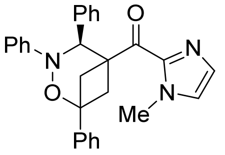

3ad
$C_{28}H_{25}N_3O_2$
M = 435.53 g/mol

(*S*)-(1-methyl-1H-imidazol-2-yl)(1,3,4-triphenyl-2-oxa-3-azabicyclo[3.1.1]heptan-5-yl)methanone: (**3ad**) Prepared from ((1-methyl-1H-imidazol-2-yl)(3-phenylbicyclo[1.1.0]butan-1-

yl)methanone (**1a**, 47.6 mg, 0.20 mmol) and (*Z*)-*N*,1-diphenylmethanimine oxide (**2d**, 47.3 mg, 0.24 mmol) according to the **GP1** at rt for 16 h. Purification by flash chromatography on silica gel using petroleum ether/EtOAc (5/1) afforded **3ad** as colorless oil (78.4 mg, 90% yield).

3ad: **R*f*** = 0.4 (petroleum ether/EtOAc = 3/1). HPLC analysis (Chiralpak AD-H, ^{i}PrOH/hexane = 10/90, 1.0 mL/min, 254 nm; tr (minor) = 13.10 min, tr (major) = 20.54 min) gave the isomeric composition of the product: 94% ee. $[\alpha]_D^{20}$ = +66.8 (*c* = 1.38, $CHCl_3$). **^{1}H NMR** (400 MHz, $CDCl_3$): δ 7.49 (d, *J* = 7.6 Hz, 2H), 7.41 (t, *J* = 7.6 Hz, 2H), 7.36-7.31 (m, 1H), 7.18-7.14 (m, 8H), 7.06 (d, *J* = 8.0 Hz, 2H), 6.97 (s, 1H), 6.86 (t, *J* = 7.4 Hz, 1H), 6.02 (s, 1H), 3.71 (s, 3H), 3.24-3.16 (m, 2H), 2.73 (d, *J* = 9.6 Hz, 1H), 2.65 (d, *J* = 9.2 Hz, 1H) ppm. **^{13}C NMR** (100 MHz, $CDCl_3$): δ 190.8, 149.8, 142.1, 140.5, 138.6, 129.4, 128.5, 128.4, 128.1, 127.6, 127.5, 126.4, 125.9, 121.6, 117.0, 83.6, 71.1, 54.1, 45.4, 35.7, 35.7 ppm. **HRMS** (ESI) *m/z*: $[M+H]^+$ calcd. for $C_{28}H_{26}N_3O_2$: 436.2020; found: 436.2011.

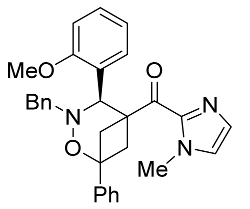

3ae

$C_{30}H_{29}N_3O_3$

M = 479.58 g/mol

(*S*)-(3-benzyl-4-(2-methoxyphenyl)-1-phenyl-2-oxa-3-azabicyclo[3.1.1]heptan-5-yl)(1-methyl-1H-imidazol-2-yl)methanone: (**3ae**) Prepared from ((1-methyl-1H-imidazol-2-yl)(3-phenylbicyclo[1.1.0]butan-1-yl)methanone (**1a**, 47.6 mg, 0.20 mmol) and (*Z*)-*N*-benzyl-1-(2-methoxyphenyl)methanimine oxide (**2e**, 57.9 mg, 0.24 mmol) according to the **GP1** at rt for 16 h. Purification by flash chromatography on silica gel using petroleum ether/EtOAc (5/1) afforded **3ae** as a white solid (89.2 mg, 93% yield).

3ae: **R*f*** = 0.4 (petroleum ether/EtOAc = 3/1). Mp: 161-163 ^{o}C. HPLC analysis (Chiralpak AD-H, ^{i}PrOH/hexane = 15/85, 1.0 mL/min, 254 nm; tr (minor) = 8.97 min, tr (major) = 11.54 min) gave the isomeric composition of the product: 99% ee. $[\alpha]_D^{20}$ =

+96.0 (c = 2.00, $CHCl_3$). **^{1}H NMR** (400 MHz, $CDCl_3$): δ 7.65 (d, J = 7.6 Hz, 1H), 7.36 (d, J = 7.6 Hz, 2H), 7.32-7.21 (m, 7H), 7.19-7.09 (m, 3H), 6.88 (t, J = 7.6 Hz, 1H), 6.81 (s, 1H), 6.60 (d, J = 8.4 Hz, 1H), 5.34 (s, 1H), 3.94 (AB, J = 14.8 Hz, 1H), 3.90 (AB, J = 14.8 Hz, 1H), 3.72 (t, J = 9.2 Hz, 1H), 3.47 (s, 3H), 3.40 (s, 3H), 2.99 (d, J = 9.6 Hz, 1H), 2.90 (t, J = 9.2 Hz, 1H), 2.46 (d, J = 10.0 Hz, 1H) ppm. **^{13}C NMR** (100 MHz, $CDCl_3$): δ 192.3, 156.8, 142.7, 141.5, 138.7, 129.2, 128.9, 128.8, 128.4, 128.1, 127.7, 127.4, 126.4, 126.1, 125.43, 125.35, 120.6, 109.9, 81.7, 65.8, 58.4, 55.2, 53.9, 50.0, 35.8, 35.0 ppm. **HRMS** (ESI) m/z: $[M+H]^+$ calcd. for $C_{30}H_{30}N_3O_3$: 480.2282; found: 480.2270.

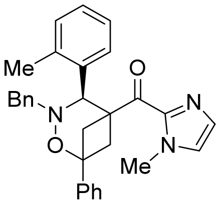

3af
$C_{30}H_{29}N_3O_2$
M = 463.58 g/mol

(*S*)-(3-benzyl-1-phenyl-4-(*o*-tolyl)-2-oxa-3-azabicyclo[3.1.1]heptan-5-yl)(1-methyl-1H-imidazol-2-yl)methanone: (**3af**) Prepared from ((1-methyl-1H-imidazol-2-yl)(3-phenylbicyclo[1.1.0]butan-1-yl)methanone (**1a**, 47.6 mg, 0.20 mmol) and (*Z*)-*N*-benzyl-1-(*o*-tolyl)methanimine oxide (**2f**, 54.0 mg, 0.24 mmol) according to the **GP1** at rt for 16 h. Purification by flash chromatography on silica gel using petroleum ether/EtOAc (5/1) afforded **3af** as a white solid (66.8 mg, 72% yield).

3af: **R_f** = 0.4 (petroleum ether/EtOAc = 3/1). Mp: 175-177 °C. HPLC analysis (Chiralpak IC, ^{i}PrOH/hexane = 15/85, 0.8 mL/min, 254 nm; tr (major) = 8.86 min, tr (minor) = 9.20 min) gave the isomeric composition of the product: 99% ee. $[\alpha]_D^{20}$ = +116.9 (c = 1.50, $CHCl_3$). **^{1}H NMR** (400 MHz, $CDCl_3$): δ 7.66 (d, J = 8.0 Hz, 1H), 7.34 (d, J = 7.2 Hz, 2H), 7.28-7.20 (m, 8H), 7.15-7.10 (m, 2H), 7.03 (t, J = 7.4 Hz, 1H), 6.90 (d, J = 7.6 Hz, 1H), 6.82 (s, 1H), 5.06 (s, 1H), 3.90 (s, 1H), 3.92 (AB, J = 14.4 Hz, 1H), 3.85 (AB, J = 14.8 Hz, 1H), 3.79 (t, J = 9.2 Hz, 1H), 3.37 (s, 3H), 3.02 (d, J = 9.6 Hz, 1H), 2.86 (t, J = 9.2 Hz, 1H), 2.50 (d, J = 10.0 Hz, 1H), 2.05 (s, 3H) ppm. **^{13}C NMR** (100 MHz, $CDCl_3$): δ 192.1, 142.7, 141.3, 138.5, 136.8, 135.9, 130.0, 128.9, 128.4, 128.2, 128.1, 127.8,

127.7, 127.5, 126.5, 126.2, 126.0, 125.4, 81.8, 69.3, 57.8, 54.2, 50.5, 36.1, 34.9, 19.4 ppm. **HRMS** (ESI) *m/z*: $[M+H]^+$ calcd. for $C_{30}H_{30}N_3O_2$: 464.2333; found: 464.2324.

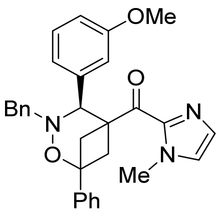

3ag
$C_{30}H_{29}N_3O_3$
M = 479.58 g/mol

(*S*)-(3-benzyl-4-(3-methoxyphenyl)-1-phenyl-2-oxa-3-azabicyclo[3.1.1]heptan-5-yl)(1-methyl-1H-imidazol-2-yl)methanone: (**3ag**) Prepared from ((1-methyl-1H-imidazol-2-yl)(3-phenylbicyclo[1.1.0]butan-1-yl)methanone (**1a**, 47.6 mg, 0.20 mmol) and (*Z*)-*N*-benzyl-1-(3-methoxyphenyl)methanimine oxide (**2g**, 57.9 mg, 0.24 mmol) according to the **GP1** at rt for 16 h. Purification by flash chromatography on silica gel using petroleum ether/EtOAc (5/1) afforded **3ag** as colorless oil (57.5 mg, 60% yield).

3ag: $\mathbf{R}_f$ = 0.4 (petroleum ether/EtOAc = 3/1). HPLC analysis (Chiralpak AD-H, ^{i}PrOH/hexane = 15/85, 0.8 mL/min, 254 nm; tr (minor) = 16.40 min, tr (major) = 17.12 min) gave the isomeric composition of the product: 98% ee. $[\alpha]_D^{20}$ = +63.7 (*c* = 1.38, $CHCl_3$). **^{1}H NMR** (400 MHz, $CDCl_3$): δ 7.38 (d, *J* = 8.4 Hz, 2H), 7.30-7.27 (m, 6H), 7.23-7.20 (m, 3H), 7.06 (t, *J* = 8.0 Hz, 1H), 6.91 (s, 1H), 6.76-6.67 (m, 3H), 4.83 (s, 1H), 4.01 (AB, *J* = 14.8 Hz, 1H), 3.86 (AB, *J* = 14.8 Hz, 1H), 3.65 (s, 3H), 3.62 (t, *J* = 9.6 Hz, 1H), 3.49 (s, 3H), 2.92 (d, *J* = 10.0 Hz, 1H), 2.79 (t, *J* = 9.2 Hz, 1H), 2.41 (d, *J* = 10.0 Hz, 1H) ppm. **^{13}C NMR** (100 MHz, $CDCl_3$): δ 191.5, 159.3, 142.8, 141.3, 139.6, 138.2, 129.2, 129.1, 128.7, 128.2, 127.9, 127.5, 126.6, 125.9, 125.4, 120.0, 114.2, 112.8, 81.7, 74.3, 58.7, 55.1, 54.1, 49.7, 35.2, 35.0 ppm. **HRMS** (ESI) *m/z*: $[M+H]^+$ calcd. for $C_{30}H_{30}N_3O_3$: 480.2282; found: 480.2271.

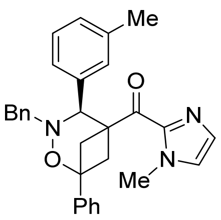

3ah
$C_{30}H_{29}N_3O_2$
M = 463.58 g/mol

(*S*)-(3-benzyl-1-phenyl-4-(*m*-tolyl)-2-oxa-3-azabicyclo[3.1.1]heptan-5-yl)(1-methyl-1H-imidazol-2-yl)methanone: (**3ah**) Prepared from ((1-methyl-1H-imidazol-2-yl)(3-phenylbicyclo[1.1.0]butan-1-yl)methanone (**1a**, 47.6 mg, 0.20 mmol) and (Z)-N-benzyl-1-(m-tolyl)methanimine oxide (**2h**, 54.0 mg, 0.24 mmol) according to the **GP1** at rt for 16 h. Purification by flash chromatography on silica gel using petroleum ether/EtOAc (5/1) afforded **3ah** as a white solid (76.0 mg, 82% yield).

3ah: $\mathbf{R}_f$ = 0.4 (petroleum ether/EtOAc = 3/1). Mp: 111-113 °C. HPLC analysis (Chiralcel IC, ^{i}PrOH/hexane = 10/90, 0.8 mL/min, 254 nm; tr (minor) = 8.18 min, tr (major) = 8.61 min) gave the isomeric composition of the product: 99% ee. $[\alpha]_D^{20}$ = +97.5 (*c* = 1.88, $CHCl_3$). **^{1}H NMR** (400 MHz, $CDCl_3$): δ 7.37 (d, *J* = 7.6 Hz, 2H), 7.30-7.26 (m, 6H), 7.24-7.19 (m, 3H), 7.04-7.01 (m, 1H), 6.97-6.93 (m, 2H), 6.87 (s, 2H), 4.78 (s, 1H), 4.00 (AB, *J* = 14.8 Hz, 1H), 3.84 (AB, *J* = 14.8 Hz, 1H), 3.64 (t, *J* = 9.4 Hz, 1H), 3.40 (s, 3H), 2.93 (d, *J* = 9.6 Hz, 1H), 2.80 (t, *J* = 9.2 Hz, 1H), 2.41 (d, *J* = 10.0 Hz, 1H), 2.19 (s, 3H) ppm. **^{13}C NMR** (100 MHz, $CDCl_3$): δ 191.7, 142.8, 141.4, 138.2, 138.0, 137.6, 129.0, 128.8, 128.6, 128.5, 128.1, 128.0, 127.8, 127.5, 126.6, 125.7, 125.4, 124.8, 81.7, 74.3, 58.6, 54.0, 49.7, 35.0, 34.9, 21.23 ppm. **HRMS** (ESI) *m/z*: $[M+H]^+$ calcd. for $C_{30}H_{30}N_3O_2$: 464.2333; found: 464.2323.

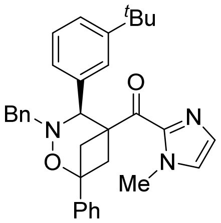

3ai
$C_{33}H_{35}N_3O_2$
M = 505.66 g/mol

(*S*)-(3-benzyl-4-(3-(tert-butyl)phenyl)-1-phenyl-2-oxa-3-azabicyclo[3.1.1]heptan-5-yl)(1-methyl-1H-imidazol-2-yl)methanone: (**3ai**) Prepared from ((1-methyl-1H-imidazol-2-yl)(3-phenylbicyclo[1.1.0]butan-1-yl)methanone (**1a**, 47.6 mg, 0.20 mmol) and (*Z*)-*N*-benzyl-1-(3-(tert-butyl)phenyl)methanimine oxide (**2i**, 64.2 mg, 0.24 mmol) according to the **GP1** at rt for 16 h. Purification by flash chromatography on silica gel using petroleum ether/EtOAc (5/1) afforded **3ai** as colorless oil (87.0 mg, 86% yield).

3ai: $\mathbf{R}_f$ = 0.4 (petroleum ether/EtOAc = 3/1). HPLC analysis (Chiralpak AD-H, ^{i}PrOH/hexane = 15/85, 1.0 mL/min, 254 nm; tr (minor) = 11.76 min, tr (major) = 18.65 min) gave the isomeric composition of the product: 99% ee. $[\alpha]_D^{20}$ = +30.9 (*c* = 2.13, $CHCl_3$). **^{1}H NMR** (400 MHz, $CDCl_3$): δ 7.37 (d, *J* = 7.2 Hz, 2H), 7.31-7.25 (m, 6H), 7.23-7.18 (m, 4H), 7.16-7.11 (m, 2H), 6.98 (s, 1H), 6.84 (s, 1H), 4.85 (s, 1H), 4.02 (AB, *J* = 14.8 Hz, 1H), 3.86 (AB, *J* = 14.8 Hz, 1H), 3.66 (t, *J* = 9.4 Hz, 1H), 3.41 (s, 3H), 2.91 (d, *J* = 9.6 Hz, 1H), 2.81 (t, *J* = 9.2 Hz, 1H), 2.41 (d, *J* = 10.4 Hz, 1H), 1.13 (s, 9H) ppm. **^{13}C NMR** (100 MHz, $CDCl_3$): δ 191.5, 150.7, 142.7, 141.4, 138.3, 137.6, 129.0, 128.6, 128.12, 128.07, 127.8, 127.5, 126.6, 125.9, 125.5, 125.4, 124.9, 124.5, 81.7, 74.7, 58.6, 54.1, 49.8, 35.2, 35.0, 34.5, 31.2 ppm. **HRMS** (ESI) *m/z*: $[M+H]^+$ calcd. for $C_{33}H_{36}N_3O_2$: 506.2802; found: 506.2792.

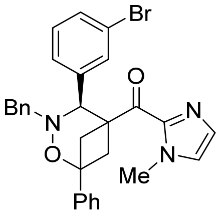

3aj
$C_{29}H_{26}BrN_3O_2$
M = 528.45 g/mol

(*S*)-(3-benzyl-4-(3-bromophenyl)-1-phenyl-2-oxa-3-azabicyclo[3.1.1]heptan-5-yl)(1-methyl-1H-imidazol-2-yl)methanone: (**3aj**) Prepared from ((1-methyl-1H-imidazol-2-yl)(3-phenylbicyclo[1.1.0]butan-1-yl)methanone (**1a**, 47.6 mg, 0.20 mmol) and (*Z*)-*N*-benzyl-1-(3-bromophenyl)methanimine oxide (**2j**, 69.6 mg, 0.24 mmol) according to the **GP1** at rt for 16 h. Purification by flash chromatography on silica gel using petroleum ether/EtOAc (5/1) afforded **3ai** as a white solid (81.4 mg, 77% yield).

3aj: **R*f*** = 0.4 (petroleum ether/EtOAc = 3/1). Mp: 70-72 °C. HPLC analysis (Chiralpak AD-H, *i*PrOH/hexane = 15/85, 1.0 mL/min, 254 nm; tr (major) = 9.82 min, tr (minor) = 13.10 min) gave the isomeric composition of the product: 99.6% ee. $[\alpha]_D^{20}$ = +47.1 (*c* = 2.00, $CHCl_3$). **^{1}H NMR** (400 MHz, $CDCl_3$): δ 7.36 (d, *J* = 7.6 Hz, 2H), 7.30-7.23 (m, 11H), 7.07-7.03 (m, 2H), 6.95 (s, 1H), 4.83 (s, 1H), 3.98 (AB, *J* = 14.8 Hz, 1H), 3.87 (AB, *J* = 14.8 Hz, 1H), 3.56 (d, *J* = 9.4 Hz, 1H), 3.52 (s, 3H), 2.91 (d, *J* = 9.6 Hz, 1H), 2.78 (t, *J* = 9.4 Hz, 1H), 2.41 (d, *J* = 10.0 Hz, 1H) ppm. **^{13}C NMR** (100 MHz, $CDCl_3$): δ 191.1, 142.4, 141.1, 140.5, 137.7, 131.2, 131.1, 129.9, 129.3, 128.7, 128.2, 127.9, 127.6, 126.8, 126.4, 126.3, 125.4, 122.0, 81.7, 73.7, 58.9, 54.1, 49.5, 35.2, 34.7 ppm. **HRMS** (ESI) *m/z*: $[M+H]^+$ calcd. for $C_{29}H_{27}BrN_3O_2$: 528.1281; found: 528.1271.

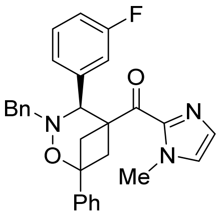

3ak
$C_{29}H_{26}FN_3O_2$
M = 467.54 g/mol

(*S*)-(3-benzyl-4-(3-fluorophenyl)-1-phenyl-2-oxa-3-azabicyclo[3.1.1]heptan-5-yl)(1-methyl-1H-imidazol-2-yl)methanone: (**3ak**) Prepared from ((1-methyl-1H-imidazol-2-yl)(3-phenylbicyclo[1.1.0]butan-1-yl)methanone (**1a**, 47.6 mg, 0.20 mmol) and (*Z*)-*N*-benzyl-1-(3-fluorophenyl)methanimine oxide (**2k**, 55.0 mg, 0.24 mmol) according to the **GP1** at rt for 16 h. Purification by flash chromatography on silica gel using petroleum ether/EtOAc (5/1) afforded **3ak** as colorless oil (68.2 mg, 73% yield).

3ak: **R*f*** = 0.4 (petroleum ether/EtOAc = 3/1). HPLC analysis (Chiralpak AD-H, *i*PrOH/hexane = 15/85, 1.0 mL/min, 254 nm; tr (major) = 10.03 min, tr (minor) = 10.55 min) gave the isomeric composition of the product: 99% ee. $[\alpha]_D^{20}$ = +86.1 (*c* = 1.50, $CHCl_3$). **^{1}H NMR** (400 MHz, $CDCl_3$): δ 7.36 (d, *J* = 7.6 Hz, 2H), 7.31-7.22 (m, 9H), 7.13-7.08 (m, 1H), 6.94-6.84 (m, 4H), 4.89 (s, 1H), 3.98 (AB, *J* = 14.8 Hz, 1H), 3.87 (AB, *J* = 14.8 Hz, 1H), 3.59 (t, *J* = 9.4 Hz, 1H), 3.53 (s, 3H), 2.90 (d, *J* = 10.0 Hz, 1H), 2.78 (t, *J* = 9.2 Hz, 1H), 2.41 (d, *J* = 10.0 Hz, 1H) ppm. **^{13}C NMR** (100 MHz, $CDCl_3$): δ 191.2,

162.5 (d, J = 244 Hz), 142.4, 141.1, 140.8 (d, J = 7 Hz), 137.9, 129.6 (d, J = 8 Hz), 129.3, 128.7, 128.2, 127.9, 127.6, 126.8, 126.2, 125.4, 123.5 (d, J = 3 Hz), 115.0 (d, J = 18 Hz), 114.8 (d, J = 19 Hz), 81.7, 73.7 (d, J = 2 Hz), 58.8, 54.1, 49.6, 35.3, 34.9 ppm. **^{19}F NMR** (376 MHz, $CDCl_3$): δ -112.755 ppm. **HRMS** (ESI) *m/z*: $[M+H]^+$ calcd. for $C_{29}H_{27}FN_3O_2$: 468.2072; found: 468.2082.

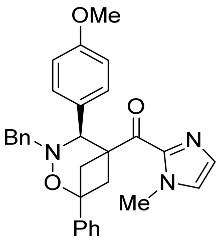

3al
$C_{30}H_{29}N_3O_3$
M = 479.58 g/mol

(*S*)-(3-benzyl-4-(4-methoxyphenyl)-1-phenyl-2-oxa-3-azabicyclo[3.1.1]heptan-5-yl)(1-methyl-1H-imidazol-2-yl)methanone: (**3al**) Prepared from ((1-methyl-1H-imidazol-2-yl)(3-phenylbicyclo[1.1.0]butan-1-yl)methanone (**1a**, 47.6 mg, 0.20 mmol) and (*Z*)-*N*-benzyl-1-(4-methoxyphenyl)methanimine oxide (**2l**, 57.9 mg, 0.24 mmol) according to the **GP1** at rt for 16 h. Purification by flash chromatography on silica gel using petroleum ether/EtOAc (5/1) afforded **3al** as a white solid (76.7 mg, 80% yield).

3al: $\mathbf{R}_f$ = 0.4 (petroleum ether/EtOAc = 3/1). Mp: 145-147 °C. HPLC analysis (Chiralpak AD-H, ^{i}PrOH/hexane = 15/85, 1.0 mL/min, 254 nm; tr (major) = 14.42 min, tr (minor) = 16.83 min) gave the isomeric composition of the product: 97% ee. $[\alpha]_D^{20}$ = +52.0 (c = 1.88, $CHCl_3$). **^{1}H NMR** (400 MHz, $CDCl_3$): δ 7.37 (d, J = 7.6 Hz, 2H), 7.29-7.20 (m, 9H), 7.07 (d, J = 8.0 Hz, 2H), 6.89 (s, 1H), 6.68 (d, J = 8.0 Hz, 2H), 4.82 (s, 1H), 3.96 (AB, J = 15.2 Hz, 1H), 3.83 (AB, J = 15.2 Hz, 1H), 3.72 (s, 3H), 3.63 (t, J = 9.4 Hz, 1H), 3.49 (s, 3H), 2.91 (d, J = 9.6 Hz, 1H), 2.78 (t, J = 9.2 Hz, 1H), 2.40 (d, J = 10.0 Hz, 1H) ppm. **^{13}C NMR** (100 MHz, $CDCl_3$): δ 191.8, 159.2, 142.6, 141.4, 138.3, 130.2, 129.1, 129.0, 128.6, 128.1, 127.8, 127.5, 126.6, 125.9, 125.4, 113.5, 81.6, 73.7, 58.3, 55.1, 54.0, 49.7, 35.3, 34.9 ppm. **HRMS** (ESI) *m/z*: $[M+H]^+$ calcd. for $C_{30}H_{30}N_3O_3$: 480.2282; found: 480.2272.

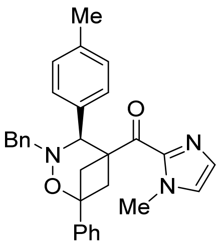

3am
$C_{30}H_{29}N_3O_2$
M = 463.58 g/mol

(*S*)-(3-benzyl-1-phenyl-4-(*p*-tolyl)-2-oxa-3-azabicyclo[3.1.1]heptan-5-yl)(1-methyl-1H-imidazol-2-yl)methanone: (**3am**) Prepared from ((1-methyl-1H-imidazol-2-yl)(3-phenylbicyclo[1.1.0]butan-1-yl)methanone (**1a**, 47.6 mg, 0.20 mmol) and (*Z*)-*N*-benzyl-1-(*p*-tolyl)methanimine oxide (**2m**, 54.0 mg, 0.24 mmol) according to the **GP1** at rt for 16 h. Purification by flash chromatography on silica gel using petroleum ether/EtOAc (5/1) afforded **3am** as a white solid (69.5 mg, 75% yield).

3am: $\mathbf{R}_f$ = 0.4 (petroleum ether/EtOAc = 3/1). Mp: 91-93 °C. HPLC analysis (Chiralpak AD-H, ^{i}PrOH/hexane = 15/85, 1.0 mL/min, 254 nm; tr (major) = 10.45 min, tr (minor) = 12.95 min) gave the isomeric composition of the product: 98% ee. $[\alpha]_D^{20}$ = +75.4 (*c* = 1.50, $CHCl_3$). **^{1}H NMR** (400 MHz, $CDCl_3$): δ 7.37 (d, *J* = 7.2 Hz, 2H), 7.29-7.20 (m, 9H), 7.02 (d, *J* = 8.0 Hz, 2H), 6.94 (d, *J* = 7.6 Hz, 2H), 6.89 (s, 1H), 4.82 (s, 1H), 4.00 (AB, *J* = 14.8 Hz, 1H), 3.83 (AB, *J* = 14.8 Hz, 1H), 3.64 (t, *J* = 9.2 Hz, 1H), 3.44 (s, 3H), 2.92 (d, *J* = 9.6 Hz, 1H), 2.79 (t, *J* = 9.2 Hz, 1H), 2.40 (d, *J* = 10.0 Hz, 1H), 2.23 (s, 3H) ppm. **^{13}C NMR** (100 MHz, $CDCl_3$): δ 191.8, 142.7, 141.4, 138.3, 137.7, 135.1, 129.1, 128.8, 128.6, 128.1, 127.8, 127.7, 127.5, 126.6, 125.9, 125.4, 81.6, 74.0, 58.4, 54.1, 49.7, 35.2, 35.0, 21.1 ppm. **HRMS** (ESI) *m/z*: $[M+H]^+$ calcd. for $C_{30}H_{30}N_3O_2$: 464.2333; found: 464.2325.

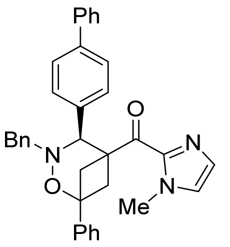

3an
$C_{35}H_{31}N_3O_2$
M = 525.65 g/mol

(*S*)-(4-([1,1'-biphenyl]-4-yl)-3-benzyl-1-phenyl-2-oxa-3-azabicyclo[3.1.1]heptan-5-yl)(1-methyl-1H-imidazol-2-yl)methanone: (**3an**) Prepared from ((1-methyl-1H-imidazol-2-yl)(3-phenylbicyclo[1.1.0]butan-1-yl)methanone (**1a**, 47.6 mg, 0.20 mmol) and (*Z*)-1-([1,1'-biphenyl]-4-yl)-N-benzylmethanimine oxide (**2n**, 70.0 mg, 0.24 mmol) according to the **GP1** at rt for 16 h. Purification by flash chromatography on silica gel using petroleum ether/EtOAc (5/1) afforded **3an** as a white solid (98.8 mg, 94% yield).

3an: $\mathbf{R_f}$ = 0.4 (petroleum ether/EtOAc = 3/1). Mp: 125-127 °C. HPLC analysis (Chiralpak AD-H, ^{i}PrOH/hexane = 15/85, 1.0 mL/min, 254 nm; tr (major) = 15.70 min, tr (minor) = 18.52 min) gave the isomeric composition of the product: 99% ee. $[\alpha]_D^{20}$ = +4.5 (*c* = 2.48, $CHCl_3$). **^{1}H NMR** (400 MHz, $CDCl_3$): δ 7.50 (d, *J* = 7.6 Hz, 2H), 7.42-7.37 (m, 6H), 7.33-7.27 (m, 7H), 7.24–7.20 (m, 5H), 6.88 (s, 1H), 4.90 (s, 1H), 4.05 (AB, *J* = 14.8 Hz, 1H), 3.89 (AB, *J* = 15.2 Hz, 1H), 3.69 (t, *J* = 9.4 Hz, 1H), 3.43 (s, 3H), 2.94 (d, *J* = 9.6 Hz, 1H), 2.82 (t, *J* = 9.0 Hz, 1H), 2.45 (d, *J* = 10.0 Hz, 1H) ppm. **^{13}C NMR** (100 MHz, $CDCl_3$): δ 191.6, 142.6, 141.3, 140.8, 140.5, 138.1, 137.2, 129.2, 128.8, 128.7, 128.3, 128.2, 127.9, 127.6, 127.3, 126.9, 126.7, 126.6, 125.9, 125.4, 81.7, 74.1, 58.6, 54.1, 49.8, 35.2, 35.0 ppm. **HRMS** (ESI) *m/z*: $[M+H]^+$ calcd. for $C_{35}H_{32}N_3O_2$: 526.2489; found: 526.2475.

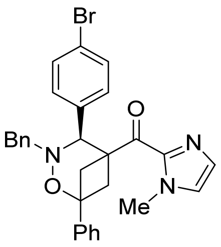

3ao
$C_{29}H_{26}BrN_3O_2$
M = 528.45 g/mol

(*S*)-(3-benzyl-4-(4-bromophenyl)-1-phenyl-2-oxa-3-azabicyclo[3.1.1]heptan-5-yl)(1-methyl-1H-imidazol-2-yl)methanone: (**3ao**) Prepared from ((1-methyl-1H-imidazol-2-yl)(3-phenylbicyclo[1.1.0]butan-1-yl)methanone (**1a**, 47.6 mg, 0.20 mmol) and (*Z*)-*N*-benzyl-1-(4-bromophenyl)methanimine oxide (**2o**, 69.6 mg, 0.24 mmol) according to the **GP1** at rt for 16 h. Purification by flash chromatography on silica gel using petroleum ether/EtOAc (5/1) afforded **3ao** as a white solid (87.7mg, 83% yield).

3ao: **R*f*** = 0.4 (petroleum ether/EtOAc = 3/1). Mp: 125-127 °C. HPLC analysis (Chiralpak AD-H, ^{i}PrOH/hexane = 15/85, 1.0 mL/min, 254 nm; tr (major) = 12.33 min, tr (minor) = 13.94 min) gave the isomeric composition of the product: 99.6% ee. $[\alpha]_D^{20}$ = +26.9 (*c* = 2.10, $CHCl_3$). **^{1}H NMR** (400 MHz, $CDCl_3$): δ 7.34 (d, *J* = 7.6 Hz, 2H), 7.30-7.21 (m, 11H), 7.05 (d, *J* = 8.0 Hz, 2H), 6.93 (s, 1H), 4.88 (s, 1H), 3.95 (AB, *J* = 14.8 Hz, 1H), 3.85 (AB, *J* = 14.8 Hz, 1H), 3.60 (t, *J* = 9.8 Hz, 1H), 3.55 (s, 3H), 2.89 (d, *J* = 9.6 Hz, 1H), 2.76 (t, *J* = 9.2 Hz, 1H), 2.41 (d, *J* = 10.0 Hz, 1H) ppm. **^{13}C NMR** (100 MHz, $CDCl_3$): δ 191.2, 142.3, 141.1, 137.8, 137.2, 131.3, 129.6, 129.3, 128.7, 128.2, 127.9, 127.6, 126.8, 126.2, 125.4, 122.0, 81.7, 73.6, 58.6, 54.0, 49.7, 35.4, 34.9 ppm. **HRMS** (ESI) *m/z*: $[M+H]^+$ calcd. for $C_{29}H_{27}BrN_3O_2$: 528.1281; found: 528.1270.

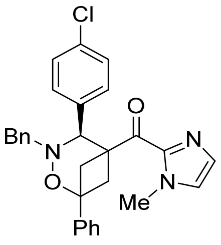

3ap
$C_{29}H_{26}ClN_3O_2$
M = 484.00 g/mol

(*S*)-(3-benzyl-4-(4-chlorophenyl)-1-phenyl-2-oxa-3-azabicyclo[3.1.1]heptan-5-yl)(1-methyl-1H-imidazol-2-yl)methanone: (**3ap**) Prepared from ((1-methyl-1H-imidazol-2-yl)(3-phenylbicyclo[1.1.0]butan-1-yl)methanone (**1a**, 47.6 mg, 0.20 mmol) and (*Z*)-*N*-benzyl-1-(4-chlorophenyl)methanimine oxide (**2p**, 58.8 mg, 0.24 mmol) according to the **GP1** at rt for 16 h. Purification by flash chromatography on silica gel using petroleum ether/EtOAc (5/1) afforded **3ap** as a white solid (77.4 mg, 80% yield).

3ap: $\mathbf{R_f}$ = 0.4 (petroleum ether/EtOAc = 3/1). Mp: 107-109 °C. HPLC analysis (Chiralpak AD-H, ^{i}PrOH/hexane = 15/85, 1.0 mL/min, 254 nm; tr (major) = 12.08 min, tr (minor) = 14.57 min) gave the isomeric composition of the product: 98% ee. $[\alpha]_D^{20}$ = +44.8 (*c* = 1.88, $CHCl_3$). **^{1}H NMR** (400 MHz, $CDCl_3$): δ 7.34 (d, *J* = 7.6 Hz, 2H), 7.30-7.20 (m, 9H), 7.13-7.19 (m, 4H), 6.92 (s, 1H), 4.89 (s, 1H), 3.95 (AB, *J* = 14.8 Hz, 1H), 3.85 (AB, *J* = 14.8 Hz, 1H), 3.60 (t, *J* = 9.4 Hz, 1H), 3.54 (s, 3H), 2.89 (d, *J* = 9.6 Hz, 1H), 2.77 (t, *J* = 9.2 Hz, 1H), 2.41 (d, *J* = 10.0 Hz, 1H) ppm. **^{13}C NMR** (100 MHz, $CDCl_3$): δ 191.2, 142.3, 141.1, 137.9, 136.7, 133.8, 129.29, 129.26, 128.6, 128.3, 128.2, 127.9, 127.6, 126.7, 126.2, 125.4, 81.7, 73.6, 58.6, 54.0, 50.0, 35.4, 34.9 ppm. **HRMS** (ESI) *m/z*: $[M+H]^+$ calcd. for $C_{29}H_{27}ClN_3O_2$: 484.1786; found: 484.1778.

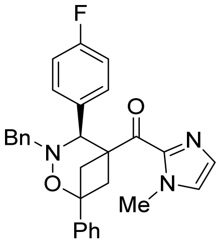

3aq
$C_{29}H_{26}FN_3O_2$
M = 467.54 g/mol

(*S*)-(3-benzyl-4-(4-fluorophenyl)-1-phenyl-2-oxa-3-azabicyclo[3.1.1]heptan-5-yl)(1-methyl-1H-imidazol-2-yl)methanone: (**3aq**) Prepared from ((1-methyl-1H-imidazol-2-yl)(3-phenylbicyclo[1.1.0]butan-1-yl)methanone (**1a**, 47.6 mg, 0.20 mmol) and (*Z*)-*N*-benzyl-1-(4-fluorophenyl)methanimine oxide (**2q**, 55.0 mg, 0.24 mmol) according to the **GP1** at rt for 16 h. Purification by flash chromatography on silica gel using petroleum ether/EtOAc (5/1) afforded **3aq** as colorless oil (77.6 mg, 83% yield).

3aq: **R*f*** = 0.4 (petroleum ether/EtOAc = 3/1). HPLC analysis (Chiralpak AD-H, ^{i}PrOH/hexane = 15/85, 1.0 mL/min, 254 nm; tr (major) = 11.00 min, tr (minor) = 14.11 min) gave the isomeric composition of the product: 98% ee. $[\alpha]_D^{20}$ = +68.1 (*c* = 1.00, $CHCl_3$). **^{1}H NMR** (400 MHz, $CDCl_3$): δ 7.35 (d, *J* = 8.8 Hz, 2H), 7.30-7.20 (m, 9H), 7.16-7.13 (m, 2H), 6.90 (s, 1H), 6.84 (t, *J* = 8.8 Hz, 2H), 4.89 (s, 1H), 3.96 (AB, *J* = 14.8 Hz, 1H), 3.85 (AB, *J* = 14.8 Hz, 1H), 3.61 (t, *J* = 9.4 Hz, 1H), 3.52 (s, 3H), 2.90 (d, *J* = 9.6 Hz, 1H), 2.78 (t, *J* = 9.0 Hz, 1H), 2.41 (d, *J* = 10.0 Hz, 1H) ppm. **^{13}C NMR** (100 MHz, $CDCl_3$): δ 191.4, 162.4 (d, *J* = 246 Hz), 142.4, 141.2, 138.0, 134.0 (d, *J* = 3 Hz), 129.6 (d, *J* = 8 Hz), 129.2, 128.6, 128.2, 127.9, 127.6, 126.7, 126.1, 125.4, 115.0 (d, *J* = 21 Hz), 114.9, 81.7, 73.5, 58.5, 54.0, 49.7, 35.3, 34.9 ppm. **^{19}F NMR** (376 MHz, $CDCl_3$): δ -113.657ppm. **HRMS** (ESI) *m/z*: $[M+H]^+$ calcd. for $C_{29}H_{27}FN_3O_2$: 468.2082; found: 468.2073.

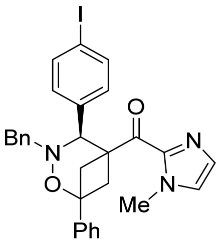

3ar
$C_{29}H_{26}IN_3O_2$
M = 575.45 g/mol

(*S*)-(3-benzyl-4-(4-iodophenyl)-1-phenyl-2-oxa-3-azabicyclo[3.1.1]heptan-5-yl)(1-methyl-1H-imidazol-2-yl)methanone: (**3ar**) Prepared from ((1-methyl-1H-imidazol-2-yl)(3-phenylbicyclo[1.1.0]butan-1-yl)methanone (**1a**, 47.6 mg, 0.20 mmol) and (*Z*)-*N*-benzyl-1-(4-iodophenyl)methanimine oxide (**2r**, 80.9 mg, 0.24 mmol) according to the **GP1** at rt for 16 h. Purification by flash chromatography on silica gel using petroleum ether/EtOAc (5/1) afforded **3ar** as a white solid (92.1 mg, 80% yield).

3ar: **R*f*** = 0.4 (petroleum ether/EtOAc = 3/1). Mp: 155-157 °C. HPLC analysis (Chiralpak AD-H, ^{i}PrOH/hexane = 15/85, 1.0 mL/min, 254 nm; tr (major) = 12.19 min, tr (minor) = 13.19 min) gave the isomeric composition of the product: 99% ee. $[\alpha]_D^{20}$ = +13.5 (*c* = 2.25, $CHCl_3$). **^{1}H NMR** (400 MHz, $CDCl_3$): δ 7.48 (d, *J* = 7.6 Hz, 2H), 7.34 (d, *J* = 7.6

Hz, 2H), 7.29-7.23 (m, 8H), 7.20 (s, 1H), 6.91 (d, *J* = 8.4 Hz, 3H), 4.86 (s, 1H), 3.95 (AB, *J* = 14.8 Hz, 1H), 3.85 (AB, *J* = 14.8 Hz, 1H), 3.59 (t, *J* = 9.4 Hz, 1H), 3.54 (s, 3H), 2.89 (d, *J* = 10.0 Hz, 1H), 2.76 (t, *J* = 9.2 Hz, 1H), 2.41 (d, *J* = 10.4 Hz, 1H) ppm. **^{13}C NMR** (100 MHz, $CDCl_3$): δ 191.2, 142.3, 141.1, 137.9, 137.8, 137.3, 129.8, 129.3, 128.6, 128.2, 127.9, 127.6, 126.7, 126.2, 125.4, 93.8, 81.7, 73.7, 58.6, 54.0, 49.6, 35.4, 34.9 ppm. **HRMS** (ESI) *m/z*: $[M+H]^+$ calcd. for $C_{29}H_{27}IN_3O_2$: 576.1142; found: 576.1133.

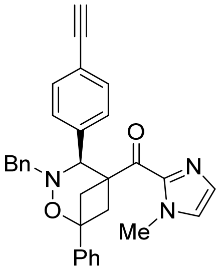

3as
$C_{31}H_{27}N_3O_2$
M = 473.58 g/mol

(*S*)-(3-benzyl-4-(4-ethynylphenyl)-1-phenyl-2-oxa-3-azabicyclo[3.1.1]heptan-5-yl)(1-methyl-1H-imidazol-2-yl)methanone: (**3as**) Prepared from ((1-methyl-1H-imidazol-2-yl)(3-phenylbicyclo[1.1.0]butan-1-yl)methanone (**1a**, 47.6 mg, 0.20 mmol) and (*Z*)-*N*-benzyl-1-(4-ethynylphenyl)methanimine oxide (**2s**, 56.5 mg, 0.24 mmol) according to the **GP1** at rt for 16 h. Purification by flash chromatography on silica gel using petroleum ether/EtOAc (5/1) afforded **3as** as a white solid (66.3 mg, 70% yield).

3as: $\mathbf{R_f}$ = 0.4 (petroleum ether/EtOAc = 3/1). Mp: 93-95 °C. HPLC analysis (Chiralpak IC, ^{i}PrOH/hexane = 5/95, 0.8 mL/min, 254 nm; tr (minor) = 10.68 min, tr (major) = 11.58 min) gave the isomeric composition of the product: 99% ee. $[\alpha]_D^{20}$ = +19.2 (*c* = 1.50, $CHCl_3$). **^{1}H NMR** (400 MHz, $CDCl_3$): δ 7.34 (d, *J* = 7.6 Hz, 2H), 7.30-7.21 (m, 11H), 7.12 (d, *J* = 7.6 Hz, 2H), 6.90 (s, 1H), 4.89 (s, 1H), 3.94 (AB, *J* = 14.8 Hz, 1H), 3.85 (AB, *J* = 14.8 Hz, 1H), 3.61 (t, *J* = 9.4 Hz, 1H), 3.50 (s, 3H), 3.03 (s, 1H), 2.90 (d, *J* = 9.6 Hz, 1H), 2.78 (t, *J* = 9.2 Hz, 1H), 2.42 (d, *J* = 10.0 Hz, 1H) ppm. **^{13}C NMR** (100 MHz, $CDCl_3$): δ 191.2, 142.4, 141.2, 139.0, 137.9, 131.9, 129.2, 128.7, 128.2, 128.0, 127.9, 127.6, 126.7, 126.1, 125.4, 121.8, 83.3, 81.7, 77.5, 74.0, 58.7, 54.1, 49.7, 35.3, 35.0 ppm. **HRMS** (ESI) *m/z*: $[M+H]^+$ calcd. for $C_{31}H_{28}N_3O_2$: 474.2176; found: 474.2169.

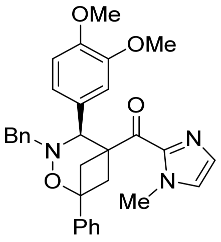

3at
$C_{31}H_{31}N_3O_4$
M = 509.61 g/mol

(*S*)-(3-benzyl-4-(3,4-dimethoxyphenyl)-1-phenyl-2-oxa-3-azabicyclo[3.1.1]heptan-5-yl)(1-methyl-1H-imidazol-2-yl)methanone: (**3at**) Prepared from ((1-methyl-1H-imidazol-2-yl)(3-phenylbicyclo[1.1.0]butan-1-yl)methanone (**1a**, 47.6 mg, 0.20 mmol) and (*Z*)-*N*-benzyl-1-(3,4-dimethoxyphenyl)methanimine oxide (**2t**, 65.1 mg, 0.24 mmol) according to the **GP1** at rt for 16 h. Purification by flash chromatography on silica gel using petroleum ether/EtOAc (5/1) afforded **3at** as a white solid (87.7 mg, 86% yield).

3at: $\mathbf{R_f}$ = 0.3 (petroleum ether/EtOAc = 1/1). Mp: 159-161 °C. HPLC analysis (Chiralpak AD-H, ^{i}PrOH/hexane = 15/85, 1.0 mL/min, 254 nm; tr (major) = 13.12 min, tr (minor) = 16.81 min) gave the isomeric composition of the product: 98% ee. $[\alpha]_D^{20}$ = +51.2 (*c* = 2.10, $CHCl_3$). **^{1}H NMR** (400 MHz, $CDCl_3$): δ 7.38 (d, *J* = 7.6 Hz, 2H), 7.30-7.22 (m, 9H), 6.91 (s, 1H), 6.85 (d, *J* = 8.4 Hz, 1H), 6.69 (d, *J* = 8.4 Hz, 1H), 6.58 (s, 1H), 4.83 (s, 1H), 4.02 (AB, *J* = 14.8 Hz, 1H), 3.86 (AB, *J* = 14.8 Hz, 1H), 3.80 (s, 3H), 3.66 (s, 3H), 3.62 (t, *J* = 9.4 Hz, 1H), 3.53 (s, 3H), 2.90 (d, *J* = 9.6 Hz, 1H), 2.77 (t, *J* = 9.2 Hz, 1H), 2.40 (d, *J* = 10.0 Hz, 1H) ppm. **^{13}C NMR** (100 MHz, $CDCl_3$): δ 191.5, 148.5, 148.2, 142.7, 141.3, 138.3, 130.6, 129.0, 128.6, 128.1, 127.8, 127.5, 126.6, 125.9, 125.4, 119.8, 111.1, 110.9, 81.6, 73.9, 58.5, 55.7, 55.6, 54.1, 49.7, 35.4, 34.9 ppm. **HRMS** (ESI) *m/z*: $[M+H]^+$ calcd. for $C_{31}H_{32}N_3O_4$: 510.2387; found: 510.2378.

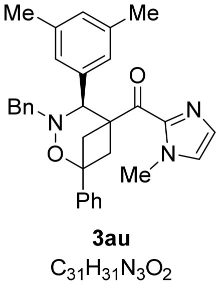

3au
$C_{31}H_{31}N_3O_2$
M = 477.61 g/mol

(*S*)-(3-benzyl-4-(3,5-dimethylphenyl)-1-phenyl-2-oxa-3-azabicyclo[3.1.1]heptan-5-yl)(1-methyl-1H-imidazol-2-yl)methanone: (**3au**) Prepared from ((1-methyl-1H-imidazol-2-yl)(3-phenylbicyclo[1.1.0]butan-1-yl)methanone (**1a**, 47.6 mg, 0.20 mmol) and (*Z*)-*N*-benzyl-1-(3,5-dimethylphenyl)methanimine oxide (**2u**, 57.4 mg, 0.24 mmol) according to the **GP1** at rt for 16 h. Purification by flash chromatography on silica gel using petroleum ether/EtOAc (5/1) afforded **3au** as colorless oil (82.1 mg, 86% yield).

3au: **R*f*** = 0.4 (petroleum ether/EtOAc = 3/1). HPLC analysis (Chiralpak AD-H, ^{i}PrOH/hexane = 10/90, 1.0 mL/min, 254 nm; tr (major) = 7.52 min, tr (minor) = 9.74 min) gave the isomeric composition of the product: 97% ee. $[\alpha]_D^{20}$ = +85.3 (*c* = 2.00, $CHCl_3$). **^{1}H NMR** (400 MHz, $CDCl_3$): δ 7.38 (d, *J* = 7.2 Hz, 2H), 7.30-7.21 (m, 9H), 6.88 (s, 1H), 6.78 (s, 1H), 6.69 (s, 2H), 4.72 (s, 1H), 4.01 (AB, *J* = 14.8 Hz, 1H), 3.83 (AB, *J* = 14.8 Hz, 1H), 3.62 (t, *J* = 9.4 Hz, 1H), 3.40 (s, 3H), 2.93 (d, *J* = 9.6 Hz, 1H), 2.79 (t, *J* = 9.2 Hz, 1H), 2.40 (d, *J* = 10.0 Hz, 1H), 2.15 (s, 6H) ppm. **^{13}C NMR** (100 MHz, $CDCl_3$): δ 191.8, 143.0, 141.4, 138.3, 137.9, 137.4, 129.6, 129.0, 128.7, 128.1, 127.8, 127.5, 126.6, 125.6, 125.5, 125.4, 81.7, 74.3, 58.6, 54.0, 49.6, 34.9, 34.8, 21.2 ppm. **HRMS** (ESI) *m/z*: $[M+H]^+$ calcd. for $C_{31}H_{32}N_3O_2$: 478.2489; found: 478.2478.

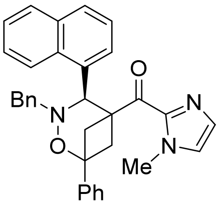

3av
$C_{33}H_{29}N_3O_2$
M = 499.61 g/mol

(*S*)-(3-benzyl-4-(naphthalen-1-yl)-1-phenyl-2-oxa-3-azabicyclo[3.1.1]heptan-5-yl)(1-methyl-1H-imidazol-2-yl)methanone: (**3av**) Prepared from ((1-methyl-1H-imidazol-2-yl)(3-phenylbicyclo[1.1.0]butan-1-yl)methanone (**1a**, 47.6 mg, 0.20 mmol) and (*Z*)-*N*-benzyl-1-(naphthalen-1-yl)methanimine oxide (**2v**, 62.7 mg, 0.24 mmol) according to the **GP1** at rt for 16 h. Purification by flash chromatography on silica gel using petroleum ether/EtOAc (5/1) afforded **3av** as a white solid (86.9 mg, 87% yield).

3av: **R*f*** = 0.4 (petroleum ether/EtOAc = 3/1). Mp: 180-182 °C. HPLC analysis (Chiralpak AD-H, *i*PrOH/hexane = 15/85, 1.0 mL/min, 254 nm; tr (major) = 11.64 min, tr (minor) = 16.06 min) gave the isomeric composition of the product: 99% ee. $[\alpha]_D^{20}$ = +165.5 (*c* = 2.00, $CHCl_3$). **^{1}H NMR** (400 MHz, $CDCl_3$): δ 8.10 (d, *J* = 7.2 Hz, 1H), 7.80 (d, *J* = 8.4 Hz, 1H), 7.70 (t, *J* = 7.8 Hz, 2H), 7.46 (t, *J* = 8.0 Hz, 1H), 7.36-7.28 (m, 7H), 7.26-7.18 (m, 5H), 7.04 (s, 1H), 6.50 (s, 1H), 5.60 (s, 1H), 3.98-3.82 (m, 3H), 3.09 (d, *J* = 10.0 Hz, 1H), 2.99 (t, *J* = 9.4 Hz, 1H), 2.83 (s, 3H), 2.45 (d, *J* = 10.4 Hz, 1H) ppm. **^{13}C NMR** (100 MHz, $CDCl_3$): δ 191.7, 142.5, 141.3, 138.0, 133.6, 133.4, 130.9, 128.8, 128.7, 128.4, 128.24, 128.17, 127.8, 127.6, 126.6, 125.8, 125.7, 125.5, 125.1, 124.9, 123.0, 81.7, 67.4, 58.6, 54.7, 49.9, 35.9, 34.3 ppm. **HRMS** (ESI) *m/z*: $[M+H]^+$ calcd. for $C_{33}H_{30}N_3O_2$: 500.2333; found: 500.2323.

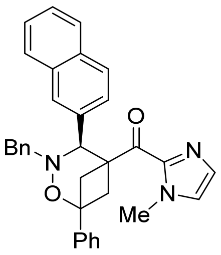

3aw
$C_{33}H_{29}N_3O_2$
M = 499.61 g/mol

(*S*)-(3-benzyl-4-(naphthalen-2-yl)-1-phenyl-2-oxa-3-azabicyclo[3.1.1]heptan-5-yl)(1-methyl-1H-imidazol-2-yl)methanone: (**3aw**) Prepared from ((1-methyl-1H-imidazol-2-yl)(3-phenylbicyclo[1.1.0]butan-1-yl)methanone (**1a**, 47.6 mg, 0.20 mmol) and (*Z*)-*N*-benzyl-1-(naphthalen-2-yl)methanimine oxide (**2w**, 62.7 mg, 0.24 mmol) according to the **GP1** at rt for 16 h. Purification by flash chromatography on silica gel using petroleum ether/EtOAc (5/1) afforded **3aw** as a white solid (82.9 mg, 83% yield).

3aw: **R*f*** = 0.4 (petroleum ether/EtOAc = 3/1). Mp: 173-175 °C. HPLC analysis (Chiralpak IC, *i*PrOH/hexane = 10/90, 0.8 mL/min, 254 nm; tr (minor) = 9.06 min, tr (major) = 9.91 min) gave the isomeric composition of the product: 98% ee. $[\alpha]_D^{20}$ = +51.8 (*c* = 2.00, $CHCl_3$). **^{1}H NMR** (400 MHz, $CDCl_3$): δ 7.75-7.72 (m, 1H), 7.67 (d, *J* = 8.4 Hz, 1H), 7.63-7.61 (m, 1H), 7.45-7.34 (m, 6H), 7.32-7.18 (m, 9H), 6.84 (s, 1H), 5.03

(s, 1H), 4.03 (AB, *J* = 14.8 Hz, 1H), 3.89 (AB, *J* = 14.8 Hz, 1H), 3.74 (t, *J* = 9.4 Hz, 1H), 3.19 (s, 3H), 2.96 (d, *J* = 10.0 Hz, 1H), 2.85 (t, *J* = 9.0 Hz, 1H), 2.47 (d, *J* = 10.0 Hz, 1H) ppm. **^{13}C NMR** (100 MHz, $CDCl_3$): δ 191.5, 142.7, 141.3, 138.1, 135.7, 133.2, 133.0, 129.2, 128.7, 128.2, 127.94, 127.85, 127.6, 127.5, 127.4, 126.7, 126.0, 125.9, 125.44, 125.35, 81.8, 74.4, 58.7, 54.2, 49.8, 35.1, 35.0 ppm. **HRMS** (ESI) *m/z*: $[M+H]^+$ calcd. for $C_{33}H_{30}N_3O_2$: 500.2333; found: 500.2323.

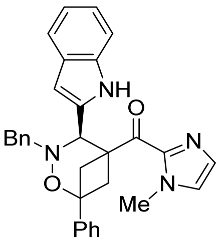

3ax
$C_{31}H_{28}N_4O_2$
M = 488.59 g/mol

(*R*)-(3-benzyl-4-(1H-indol-2-yl)-1-phenyl-2-oxa-3-azabicyclo[3.1.1]heptan-5-yl)(1-methyl-1H-imidazol-2-yl)methanone: (**3ax**) Prepared from ((1-methyl-1H-imidazol-2-yl)(3-phenylbicyclo[1.1.0]butan-1-yl)methanone (**1a**, 47.6 mg, 0.20 mmol) and (*Z*)-*N*-benzyl-1-(1H-indol-2-yl)methanimine oxide (**2x**, 60.1 mg, 0.24 mmol) according to the **GP1** at rt for 16 h. Purification by flash chromatography on silica gel using petroleum ether/EtOAc (5/1) afforded **3ax** as a white solid (30.3 mg, 31% yield).

3ax: **R*f*** = 0.4 (petroleum ether/EtOAc = 3/1). Mp: 188-190 °C. HPLC analysis (Chiralpak AD-H, ^{i}PrOH/hexane = 15/85, 1.0 mL/min, 254 nm; tr (minor) = 14.81 min, tr (major) = 28.58 min) gave the isomeric composition of the product: 95% ee. $[\alpha]_D^{20}$ = +18.1 (*c* = 0.75, $CHCl_3$). **^{1}H NMR** (400 MHz, $CDCl_3$): δ 8.45 (s, 1H), 7.40 (d, *J* = 8.0 Hz, 1H), 7.37-7.22 (m, 12H), 7.10 (t, *J* = 7.6 Hz, 1H), 7.01 (t, *J* = 7.6 Hz, 1H), 6.92 (s, 1H), 5.88 (s, 1H), 5.24 (s, 1H), 4.10 (AB, *J* = 14.8 Hz, 1H), 3.96 (AB, *J* = 14.8 Hz, 1H), 3.57 (t, *J* = 9.4 Hz, 1H), 3.51 (s, 3H), 2.86 (d, *J* = 9.6 Hz, 1H), 2.75 (t, *J* = 9.2 Hz, 1H), 2.50 (d, *J* = 10.4 Hz, 1H) ppm. **^{13}C NMR** (100 MHz, $CDCl_3$): δ 190.5, 142.4, 141.0, 137.5, 135.8, 134.9, 129.2, 129.0, 128.3, 128.0, 127.7, 126.9, 126.3, 125.4, 121.9, 120.2,

119.7, 111.1, 101.4, 81.8, 68.1, 59.2, 53.4, 49.0, 35.5, 34.8 ppm. **HRMS** (ESI) *m/z*: $[M+H]^+$ calcd. for $C_{31}H_{29}N_4O_2$: 489.2275; found: 489.2275.

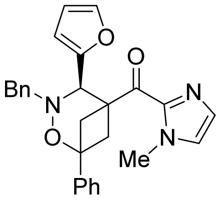

3ay
$C_{27}H_{25}N_3O_3$
M = 439.52 g/mol

(*R*)-(3-benzyl-4-(furan-2-yl)-1-phenyl-2-oxa-3-azabicyclo[3.1.1]heptan-5-yl)(1-methyl-1H-imidazol-2-yl)methanone: (**3ay**) Prepared from ((1-methyl-1H-imidazol-2-yl)(3-phenylbicyclo[1.1.0]butan-1-yl)methanone (**1a**, 47.6 mg, 0.20 mmol) and (*Z*)-*N*-benzyl-1-(furan-2-yl)methanimine oxide (**2y**, 48.3 mg, 0.24 mmol) according to the **GP1** at rt for 16 h. Purification by flash chromatography on silica gel using petroleum ether/EtOAc (5/1) afforded **3ay** as a white solid (52.7 mg, 60% yield).

3ay: $\mathbf{R_f}$ = 0.4 (petroleum ether/EtOAc = 3/1). Mp: 107-109 °C. HPLC analysis (Chiralpak AD-H, ^{i}PrOH/hexane = 15/85, 1.0 mL/min, 254 nm; tr (major) = 14.63 min, tr (minor) = 19.42 min) gave the isomeric composition of the product: 99% ee. $[\alpha]_D^{20}$ = +56.4 (*c* = 1.00, $CHCl_3$). **^{1}H NMR** (400 MHz, $CDCl_3$): δ 7.39 (d, *J* = 7.6 Hz, 2H), 7.32-7.21 (m, 10H), 7.05 (s, 1H), 6.97 (s, 1H), 6.18 (s, 1H), 5.01 (s, 1H), 4.11 (AB, *J* = 14.8 Hz, 1H), 3.89 (AB, *J* = 14.8 Hz, 1H), 3.73 (s, 3H), 3.50 (t, *J* = 9.4 Hz, 1H), 2.83 (d, *J* = 9.6 Hz, 1H), 2.71 (t, *J* = 9.2 Hz, 1H), 2.38 (d, *J* = 10.4 Hz, 1H) ppm. **^{13}C NMR** (100 MHz, $CDCl_3$): δ 191.3, 143.0, 142.3, 141.3, 140.4, 138.2, 129.2, 128.6, 128.2, 127.9, 127.5, 126.7, 126.2, 125.4, 122.5, 109.1, 81.7, 66.2, 58.6, 53.4, 49.1, 35.6, 34.4 ppm. **HRMS** (ESI) *m/z*: $[M+H]^+$ calcd. for $C_{27}H_{26}N_3O_3$: 440.1969; found: 440.1965.

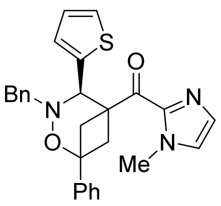

3az
$C_{27}H_{25}N_3O_2S$
M = 455.58 g/mol

(*R*)-(3-benzyl-1-phenyl-4-(thiophen-2-yl)-2-oxa-3-azabicyclo[3.1.1]heptan-5-yl)(1-methyl-1H-imidazol-2-yl)methanone: (**3az**) Prepared from ((1-methyl-1H-imidazol-2-yl)(3-phenylbicyclo[1.1.0]butan-1-yl)methanone (**1a**, 47.6 mg, 0.20 mmol) and (*Z*)-*N*-benzyl-1-(thiophen-2-yl)methanimine oxide (**2z**, 52.2 mg, 0.24 mmol) according to the **GP1** at rt for 16 h. Purification by flash chromatography on silica gel using petroleum ether/EtOAc (5/1) afforded **3az** as a white solid (72.9 mg, 80% yield).

3az: $\mathbf{R}_f$ = 0.4 (petroleum ether/EtOAc = 3/1). Mp: 91-93 °C. HPLC analysis (Chiralpak AD-H, ^{i}PrOH/hexane = 15/85, 1.0 mL/min, 254 nm; tr (minor) = 12.91 min, tr (major) = 14.54 min) gave the isomeric composition of the product: 99.7% ee. $[\alpha]_D^{20}$ = +71.2 (*c* = 1.75, $CHCl_3$). **^{1}H NMR** (400 MHz, $CDCl_3$): δ 7.42 (d, *J* = 7.6 Hz, 2H), 7.32-7.21 (m, 9H), 7.17 (d, *J* = 5.2 Hz, 1H), 6.97 (s, 1H), 6.74 (t, *J* = 4.2 Hz, 1H), 6.35 (s, 1H), 5.37 (s, 1H), 4.12 (AB, *J* = 14.8 Hz, 1H), 3.89 (AB, *J* = 14.8 Hz, 1H), 3.64 (s, 3H), 3.59 (t, *J* = 9.4 Hz, 1H), 2.87 (d, *J* = 9.6 Hz, 1H), 2.76 (t, *J* = 9.2 Hz, 1H), 2.37 (d, *J* = 10.4 Hz, 1H) ppm. **^{13}C NMR** (100 MHz, $CDCl_3$): δ 190.7, 142.4, 141.14, 141.09, 138.0, 129.2, 128.7, 128.2, 127.9, 127.6, 126.7, 126.2, 125.8, 125.7, 125.4, 124.9, 81.7, 69.8, 58.7, 54.1, 48.6, 35.5, 34.1 ppm. **HRMS** (ESI) *m/z*: $[M+H]^+$ calcd. for $C_{27}H_{26}N_3O_2S$: 456.1740; found: 456.1735.

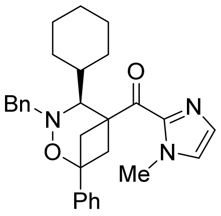

3aaa
$C_{29}H_{33}N_3O_2$
M = 455.60 g/mol

(*S*)-(3-benzyl-4-cyclohexyl-1-phenyl-2-oxa-3-azabicyclo[3.1.1]heptan-5-yl)(1-methyl-1H-imidazol-2-yl)methanone: (**3aaa**) Prepared from ((1-methyl-1H-imidazol-2-yl)(3-phenylbicyclo[1.1.0]butan-1-yl)methanone (**1a**, 47.6 mg, 0.20 mmol) and (*Z*)-*N*-benzyl-1-cyclohexylmethanimine oxide (**2aa**, 52.2 mg, 0.24 mmol) according to the **GP1** at rt for 16 h. Purification by flash chromatography on silica gel using petroleum ether/EtOAc (5/1) afforded **3aaa** as a white solid (54.7 mg, 60% yield).

3aaa: $\mathbf{R}_f$ = 0.4 (petroleum ether/EtOAc = 3/1). Mp: 131-133 °C. HPLC analysis (Chiralpak AD-H, ^{i}PrOH/hexane = 15/85, 1.0 mL/min, 254 nm; tr (major) = 7.40 min, tr (minor) = 8.31 min) gave the isomeric composition of the product: 70% ee. $[\alpha]_D^{20}$ = +67.1 (*c* = 1.38, $CHCl_3$). **^{1}H NMR** (600 MHz, $CDCl_3$): δ 7.49 (d, *J* = 7.2 Hz, 2H), 7.33 (t, *J* = 5.0 Hz, 2H), 7.24 (s, 5H), 7.20-7.19 (m, 2H), 7.01 (s, 1H), 4.44 (AB, *J* = 14.8 Hz, 1H), 4.27 (AB, *J* = 14.8 Hz, 1H), 4.12 (s, 1H), 4.00 (s, 3H), 3.07 (t, *J* = 9.4 Hz, 1H), 2.78 (t, *J* = 9.4 Hz, 1H), 2.60 (d, *J* = 10.8 Hz, 1H), 2.49 (d, *J* = 10.2 Hz, 1H), 1.84 (d, *J* = 12.6 Hz, 1H), 1.65-1.54 (m, 5H), 1.17-1.11 (m, 1H), 1.07-0.96 (m, 3H), 0.78-0.74 (m, 1H) ppm. **^{13}C NMR** (100 MHz, $CDCl_3$): δ 192.9, 142.1, 141.4, 139.0, 129.2, 128.8, 128.1, 128.0, 127.4, 126.7, 126.3, 125.4, 81.4, 73.2, 63.2, 52.4, 48.5, 43.0, 36.6, 36.2, 29.9, 29.3, 27.2, 27.1, 26.5 ppm. **HRMS** (ESI) *m/z*: $[M+H]^+$ calcd. for $C_{29}H_{34}N_3O_2$: 456.2646; found: 456.2638.

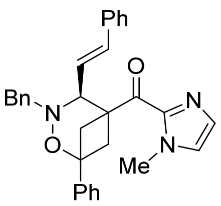

3abb
$C_{31}H_{29}N_3O_2$
M = 475.59 g/mol

(*S,E*)-(3-benzyl-1-phenyl-4-styryl-2-oxa-3-azabicyclo[3.1.1]heptan-5-yl)(1-methyl-1H-imidazol-2-yl)methanone: (**3abb**) Prepared from ((1-methyl-1H-imidazol-2-yl)(3-phenylbicyclo[1.1.0]butan-1-yl)methanone (**1a**, 47.6 mg, 0.20 mmol) and (1*Z*,2*E*)-*N*-benzyl-3-phenylprop-2-en-1-imine oxide (**2bb**, 57.0 mg, 0.24 mmol) according to the

GP1 at rt for 16 h. Purification by flash chromatography on silica gel using petroleum ether/EtOAc (5/1) afforded **3abb** as a white solid (73.2 mg, 77% yield).

3abb: **R*f*** = 0.4 (petroleum ether/EtOAc = 3/1). Mp: 141-143 °C. HPLC analysis (Chiralpak AD-H, *ⁱ*PrOH/hexane = 10/90, 1.0 mL/min, 254 nm; tr (minor) = 13.50 min, tr (major) = 14.24 min) gave the isomeric composition of the product: 97% ee. $[\alpha]_D^{20}$ = +12.3 (*c* = 1.75, $CHCl_3$). **^{1}H NMR** (400 MHz, $CDCl_3$): δ 7.44 (d, *J* = 7.6 Hz, 2H), 7.30-7.27 (m, 6H), 7.22-7.18 (m, 6H), 7.15-7.13 (m, 2H), 6.90 (s, 1H), 6.19-6.08 (m, 2H), 4.50 (d, *J* = 8.2 Hz, 1H), 4.28 (AB, *J* = 14.8 Hz, 1H), 3.98 (AB, *J* = 14.8 Hz, 1H), 3.69 (s, 3H), 3.50 (t, *J* = 9.2 Hz, 1H), 2.83 (d, *J* = 9.6 Hz, 1H), 2.71 (t, *J* = 9.0 Hz, 1H), 2.39 (d, *J* = 10.0 Hz, 1H) ppm. **^{13}C NMR** (100 MHz, $CDCl_3$): δ 191.9, 142.5, 141.3, 138.1, 136.3, 133.9, 129.3, 128.7, 128.4, 128.1, 127.9, 127.8, 127.5, 126.6, 126.4, 126.2, 125.8, 125.4, 81.6, 72.8, 58.8, 52.5, 48.7, 35.6, 34.8 ppm. **HRMS** (ESI) *m/z*: $[M+H]^+$ calcd. for $C_{31}H_{30}N_3O_2$: 476.2333; found: 476.2324.

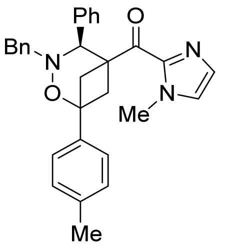

3bb

$C_{30}H_{29}N_3O_2$

M = 463.58 g/mol

(*S*)-(3-benzyl-4-phenyl-1-(*p*-tolyl)-2-oxa-3-azabicyclo[3.1.1]heptan-5-yl)(1-methyl-1H-imidazol-2-yl)methanone: (**3bb**) Prepared from (1-methyl-1H-imidazol-2-yl)(3-(p-tolyl)bicyclo[1.1.0]butan-1-yl)methanone (**1b**, 50.5 mg, 0.20 mmol) and (*Z*)-*N*-benzyl-1-phenylmethanimine oxide (**2b**, 50.7 mg, 0.24 mmol) according to the **GP1** at rt for 16 h. Purification by flash chromatography on silica gel using petroleum ether/EtOAc (5/1) afforded **3bb** as colorless oil (83.4 mg, 90% yield).

3bb: **R*f*** = 0.4 (petroleum ether/EtOAc = 3/1). HPLC analysis (Chiralpak AD-H, *ⁱ*PrOH/hexane = 15/85, 1.0 mL/min, 254 nm; tr (minor) = 10.34 min, tr (major) = 21.60 min) gave the isomeric composition of the product: 99% ee. $[\alpha]_D^{20}$ = +90.2 (*c* = 2.00,

CHCl3). **1H NMR** (400 MHz, CDCl3): δ 7.36 (d, *J* = 7.6 Hz, 2H), 7.28-7.23 (m, 2H), 7.21-7.18 (m, 4H), 7.13-7.08 (m, 7H), 6.86 (s, 1H), 4.83 (s, 1H), 3.98 (AB, *J* = 14.8 Hz, 1H), 3.84 (AB, *J* = 14.8 Hz, 1H), 3.63 (t, *J* = 9.4 Hz, 1H), 3.40 (s, 3H), 2.90 (d, *J* = 9.6 Hz, 1H), 2.79 (t, *J* = 9.0 Hz, 1H), 2.39 (d, *J* = 10.0 Hz, 1H), 2.29 (s, 3H) ppm. **13C NMR** (100 MHz, CDCl3): δ 191.7, 142.6, 138.5, 138.2, 137.2, 129.1, 128.8, 128.6, 128.1, 128.0, 127.9, 127.8, 126.6, 125.8, 125.3, 81.6, 74.3, 58.5, 54.1, 49.8, 35.1, 34.9, 21.1 ppm. **HRMS** (ESI) *m/z*: $[M+H]^+$ calcd. for $C_{30}H_{30}N_3O_2$: 464.2333; found: 464.2323.

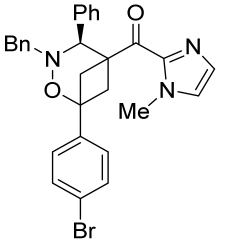

3cb
$C_{29}H_{26}BrN_3O_2$
M = 528.45 g/mol

(*S*)-(3-benzyl-1-(4-bromophenyl)-4-phenyl-2-oxa-3-azabicyclo[3.1.1]heptan-5-yl)(1-methyl-1H-imidazol-2-yl)methanone: (**3cb**) Prepared from (3-(4-bromophenyl)bicyclo[1.1.0]butan-1-yl)(1-methyl-1H-imidazol-2-yl)methanone (**1c**, 63.4 mg, 0.20 mmol) and (*Z*)-*N*-benzyl-1-phenylmethanimine oxide (**2b**, 50.7 mg, 0.24 mmol) according to the **GP1** at rt for 16 h. Purification by flash chromatography on silica gel using petroleum ether/EtOAc (5/1) afforded **3cb** as colorless oil (100.4 mg, 95% yield).

3cb: **R*f*** = 0.4 (petroleum ether/EtOAc = 3/1). HPLC analysis (Chiralpak AD-H, ^{i}PrOH/hexane = 15/85, 1.0 mL/min, 254 nm; tr (minor) = 11.66 min, tr (major) = 28.74 min) gave the isomeric composition of the product: 99% ee. $[\alpha]_D^{20}$ = +82.1 (*c* = 1.25, CHCl3). **1H NMR** (400 MHz, CDCl3): δ 7.40 (d, *J* = 8.2 Hz, 2H), 7.35 (d, *J* = 7.6 Hz, 2H), 7.30-7.25 (m, 3H), 7.23-7.20 (m, 2H), 7.16-7.14 (m, 7H), 6.89 (s, 1H), 4.83 (s, 1H), 3.98 (AB, *J* = 14.8 Hz, 1H), 3.83 (AB, *J* = 14.8 Hz, 1H), 3.62 (t, *J* = 9.2 Hz, 1H), 3.43 (s, 3H), 2.88 (d, *J* = 9.6 Hz, 1H), 2.78 (t, *J* = 9.2 Hz, 1H), 2.38 (d, *J* = 10.0 Hz, 1H) ppm. **13C NMR** (100 MHz, CDCl3): δ 191.4, 142.6, 140.4, 138.1, 137.9, 131.3, 129.2, 128.6, 128.2, 127.89, 127.86, 127.2, 126.7, 126.0, 121.6, 81.2, 74.4, 58.6, 54.0, 49.8, 35.2,

35.1 ppm. **HRMS** (ESI) *m/z*: $[M+H]^+$ calcd. for $C_{29}H_{27}BrN_3O_2$: 528.1281; found: 528.1271.

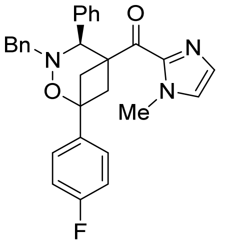

3db
$C_{29}H_{26}FN_3O_2$
M = 467.54 g/mol

(*S*)-(3-benzyl-1-(4-fluorophenyl)-4-phenyl-2-oxa-3-azabicyclo[3.1.1]heptan-5-yl)(1-methyl-1H-imidazol-2-yl)methanone: (**3db**) Prepared from (3-(4-fluorophenyl)bicyclo[1.1.0]butan-1-yl)(1-methyl-1H-imidazol-2-yl)methanone (**1d**, 51.2 mg, 0.20 mmol) and (*Z*)-*N*-benzyl-1-phenylmethanimine oxide (**2b**, 50.6 mg, 0.24 mmol) according to the **GP1** at rt for 16 h. Purification by flash chromatography on silica gel using petroleum ether/EtOAc (5/1) afforded **3db** as colorless oil (87.6 mg, 94% yield).

3db: $\mathbf{R}_f$ = 0.4 (petroleum ether/EtOAc = 3/1). HPLC analysis (Chiralpak AD-H, ^{i}PrOH/hexane = 15/85, 1.0 mL/min, 254 nm; tr (minor) = 10.27 min, tr (major) = 17.57 min) gave the isomeric composition of the product: 98% ee. $[\alpha]_D^{20}$ = +89.7 (*c* = 1.00, $CHCl_3$). **^{1}H NMR** (400 MHz, $CDCl_3$): δ 7.35 (d, *J* = 8.0 Hz, 2H), 7.30-7.20 (m, 6H), 7.14 (s, 5H), 6.96 (d, *J* = 9.0 Hz, 2H), 6.88 (s, 1H), 4.83 (s, 1H), 3.98 (AB, *J* = 14.8 Hz, 1H), 3.83 (d, *J* = 14.8 Hz, 1H), 3.63 (t, *J* = 9.4 Hz, 1H), 3.42 (s, 3H), 2.89 (d, *J* = 9.6 Hz, 1H), 2.79 (t, *J* = 9.2 Hz, 1H), 2.38 (d, *J* = 10.0 Hz, 1H) ppm. **^{13}C NMR** (100 MHz, $CDCl_3$): δ 191.5, 162.2 (d, *J* = 245 Hz), 142.6, 138.1 (d, *J* = 11 Hz), 137.3 (d, *J* = 3 Hz), 129.2, 128.6, 128.2, 128.1, 127.9, 127.23, 127.15, 126.7, 125.9, 115.0 (d, *J* = 22 Hz), 81.3, 74.4, 58.6, 54.0, 49.8, 35.12, 35.06 ppm. **^{19}F NMR** (376 MHz, $CDCl_3$) δ -114.912 ppm. **HRMS** (ESI) *m/z*: $[M+H]^+$ calcd. for $C_{29}H_{27}FN_3O_2$: 468.2082; found: 468.2073.

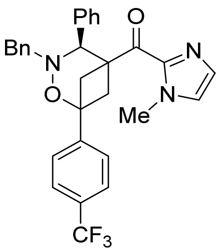

3eb
$C_{30}H_{26}F_3N_3O_2$
M = 517.55 g/mol

(S)-(3-benzyl-4-phenyl-1-(4-(trifluoromethyl)phenyl)-2-oxa-3-azabicyclo[3.1.1]heptan-5-yl)(1-methyl-1H-imidazol-2-yl)methanone: (**3eb**) Prepared from (1-methyl-1H-imidazol-2-yl)(3-(4-(trifluoromethyl)phenyl)bicyclo[1.1.0]butan-1-yl)methanone (**1e**, 61.2 mg, 0.20 mmol) and (*Z*)-*N*-benzyl-1-phenylmethanimine oxide (**2b**, 50.7 mg, 0.24 mmol) according to the **GP1** at rt for 16 h. Purification by flash chromatography on silica gel using petroleum ether/EtOAc (5/1) afforded **3eb** as colorless oil (86.6 mg, 84% yield).

3eb: **R_f** = 0.4 (petroleum ether/EtOAc = 3/1). HPLC analysis (Chiralpak AD-H, ^{i}PrOH/hexane = 15/85, 1.0 mL/min, 254 nm; tr (minor) = 9.51 min, tr (major) = 23.71 min) gave the isomeric composition of the product: 98% ee. $[\alpha]_D^{20}$ = +93.9 (*c* = 1.00, $CHCl_3$). **^{1}H NMR** (600 MHz, $CDCl_3$): δ 7.54 (d, *J* = 7.8 Hz, 2H), 7.39-7.35 (m, 4H), 7.30 (t, *J* = 7.2 Hz, 2H), 7.25-7.21 (m, 2H), 7.18-7.14 (m, 5H), 6.91 (s, 1H), 4.86 (s, 1H), 4.00 (AB, *J* = 14.8 Hz, 1H), 3.84 (AB, *J* = 14.8 Hz, 1H), 3.65 (t, *J* = 9.6 Hz, 1H), 3.45 (s, 3H), 2.93 (d, *J* = 9.6 Hz, 1H), 2.82 (t, *J* = 9.2 Hz, 1H), 2.43 (d, *J* = 10.0 Hz, 1H) ppm. **^{13}C NMR** (150 MHz, $CDCl_3$): δ 191.3, 145.1, 142.5, 138.0, 137.8, 129.7 (q, *J* = 32 Hz), 129.2, 128.6, 128.23, 128.21, 127.9, 127.8, 126.8, 126.0, 125.7, 125.2 (q, *J* = 4 Hz), 124.1 (q, *J* = 270 Hz), 81.2, 74.5, 58.7, 54.1, 49.9, 35.2 ppm. **^{19}F NMR** (565 MHz, $CDCl_3$) δ -62.520 ppm. **HRMS** (ESI) *m/z*: $[M+K]^+$ calcd. for $C_{30}H_{26}F_3N_3O_2K$: 556.1609; found: 556.1603.

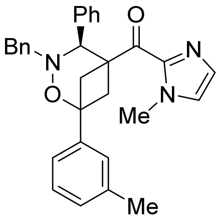

3fb
$C_{30}H_{29}N_3O_2$
M = 463.58 g/mol

(*S*)-(3-benzyl-4-phenyl-1-(*m*-tolyl)-2-oxa-3-azabicyclo[3.1.1]heptan-5-yl)(1-methyl-1H-imidazol-2-yl)methanone: (**3fb**) Prepared from (1-methyl-1H-imidazol-2-yl)(3-(*m*-tolyl)bicyclo[1.1.0]butan-1-yl)methanone (**1f**, 50.5 mg, 0.20 mmol) and (*Z*)-*N*-benzyl-1-phenylmethanimine oxide (**2b**, 50.7 mg, 0.24 mmol) according to the **GP1** at rt for 16 h. Purification by flash chromatography on silica gel using petroleum ether/EtOAc (5/1) afforded **3fb** as a white solid (79.7 mg, 86% yield).

3fb: $\mathbf{R}_f$ = 0.4 (petroleum ether/EtOAc = 3/1). Mp: 121-123 °C. HPLC analysis (Chiralpak AD-H, ^{i}PrOH/hexane = 10/90, 1.0 mL/min, 254 nm; tr (minor) = 5.06 min, tr (major) = 6.24 min) gave the isomeric composition of the product: 99% ee. $[\alpha]_D^{20}$ = +84.9 (*c* = 2.00, $CHCl_3$). **^{1}H NMR** (400 MHz, $CDCl_3$): δ 7.37 (d, *J* = 7.6 Hz, 2H), 7.27 (t, *J* = 7.6 Hz, 2H), 7.23-7.17 (m, 3H), 7.16-7.08 (m, 7H), 7.04 (d, *J* = 7.6 Hz, 1H), 6.87 (s, 1H), 4.83 (s, 1H), 3.99 (AB, *J* = 14.8 Hz, 1H), 3.85 (AB, *J* = 14.8 Hz, 1H), 3.64 (t, *J* = 9.4 Hz, 1H), 3.41 (s, 3H), 2.92 (d, *J* = 9.6 Hz, 1H), 2.79 (t, *J* = 9.2 Hz, 1H), 2.40 (d, *J* = 10.0 Hz, 1H), 2.28 (s, 3H) ppm. **^{13}C NMR** (100 MHz, $CDCl_3$): δ 191.7, 142.6, 141.3, 138.2, 138.1, 137.7, 129.1, 128.7, 128.3, 128.10, 128.06, 127.9, 127.8, 126.6, 126.2, 125.9, 122.4, 81.7, 74.4, 58.5, 54.1, 49.7, 35.1, 35.1, 21.4 ppm. **HRMS** (ESI) *m/z*: $[M+H]^+$ calcd. for $C_{30}H_{30}N_3O_2$: 464.2333; found: 464.2326.

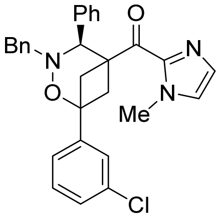

3gb
$C_{29}H_{26}ClN_3O_2$
M = 484.00 g/mol

(*S*)-(3-benzyl-1-(3-chlorophenyl)-4-phenyl-2-oxa-3-azabicyclo[3.1.1]heptan-5-yl)(1-methyl-1H-imidazol-2-yl)methanone: (**3gb**) Prepared from (3-(3-chlorophenyl)bicyclo[1.1.0]butan-1-yl)(1-methyl-1H-imidazol-2-yl)methanone (**1g**, 54.5 mg, 0.20 mmol) and (*Z*)-*N*-benzyl-1-phenylmethanimine oxide (**2b**, 50.7 mg, 0.24 mmol) according to the **GP1** at rt for 16 h. Purification by flash chromatography on silica gel using petroleum ether/EtOAc (5/1) afforded **3gb** as a white solid (90.0 mg, 93% yield).

3gb: **R*f*** = 0.4 (petroleum ether/EtOAc = 3/1). Mp: 137-139 °C. HPLC analysis (Chiralpak AD-H, *ⁱ*PrOH/hexane = 15/85, 1.0 mL/min, 254 nm; tr (minor) = 8.06 min, tr (major) = 11.52 min) gave the isomeric composition of the product: 98% ee. $[\alpha]_D^{20}$ = +84.3 (*c* = 2.00, $CHCl_3$). **^{1}H NMR** (400 MHz, $CDCl_3$): δ 7.36 (d, *J* = 7.2 Hz, 2H), 7.31-7.27 (m, 3H), 7.24-7.19 (m, 4H), 7.14 (s, 6H), 6.88 (s, 1H), 4.84 (s, 1H), 3.99 (AB, *J* = 14.8 Hz, 1H), 3.84 (AB, *J* = 14.8 Hz, 1H), 3.62 (t, *J* = 9.4 Hz, 1H), 3.42 (s, 3H), 2.89 (d, *J* = 10.0 Hz, 1H), 2.79 (t, *J* = 9.2 Hz, 1H), 2.39 (d, *J* = 10.0 Hz, 1H) ppm. **^{13}C NMR** (100 MHz, $CDCl_3$): δ 191.3, 143.3, 142.5, 138.0, 137.9, 134.1, 129.5, 129.2, 128.7, 128.2, 127.9, 127.8, 127.6, 126.8, 126.0, 125.9, 123.5, 81.2, 74.4, 58.6, 54.0, 49.9, 35.1 ppm. **HRMS** (ESI) *m/z*: $[M+H]^+$ calcd. for $C_{29}H_{27}ClN_3O_2$: 484.1786; found: 484.1778.

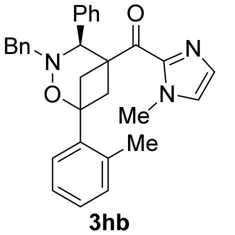

3hb
$C_{30}H_{29}N_3O_2$
M = 463.58 g/mol

(*S*)-(3-benzyl-4-phenyl-1-(*o*-tolyl)-2-oxa-3-azabicyclo[3.1.1]heptan-5-yl)(1-methyl-1H-imidazol-2-yl)methanone: (**3hb**) Prepared from (1-methyl-1H-imidazol-2-yl)(3-(*o*-tolyl)bicyclo[1.1.0]butan-1-yl)methanone (**1h**, 50.5 mg, 0.20 mmol) and (*Z*)-*N*-benzyl-1-phenylmethanimine oxide (**2b**, 50.7 mg, 0.24 mmol) according to the **GP1** at rt for 16 h. Purification by flash chromatography on silica gel using petroleum ether/EtOAc (5/1) afforded **3hb** as colorless oil (83.4 mg, 90% yield).

3hb: **R_f** = 0.4 (petroleum ether/EtOAc = 3/1). HPLC analysis (Chiralpak AD-H, ^{i}PrOH/hexane = 15/85, 1.0 mL/min, 254 nm; tr (minor) = 10.12 min, tr (major) = 15.60 min) gave the isomeric composition of the product: 94% ee. $[\alpha]_D^{20}$ = +111.5 (*c* = 2.00, $CHCl_3$). **^{1}H NMR** (400 MHz, $CDCl_3$): δ 7.29 (d, *J* = 7.2 Hz, 2H), 7.24-7.22 (m, 3H), 7.19-7.09 (m, 9H), 7.03 (d, *J* = 7.2 Hz, 1H), 6.87 (s, 1H), 4.82 (s, 1H), 3.93 (AB, *J* = 14.4 Hz, 1H), 3.88 (t, *J* = 9.0 Hz, 1H), 3.74 (AB, *J* = 14.4 Hz, 1H), 3.39 (s, 3H), 2.98-2.91 (m, 2H), 2.36 (d, *J* = 10.0 Hz, 1H), 2.19 (s, 3H) ppm. **^{13}C NMR** (100 MHz, $CDCl_3$): δ 191.6, 142.7, 138.5, 138.3, 138.2, 130.9, 129.1, 129.0, 128.2, 128.12, 128.06, 127.9, 127.7, 126.66, 126.65, 125.8, 125.2, 82.9, 74.3, 58.7, 54.0, 49.2, 35.1, 33.4, 19.6 ppm. **HRMS** (ESI) *m/z*: $[M+H]^+$ calcd. for $C_{30}H_{30}N_3O_2$: 464.2333; found: 464.2325.

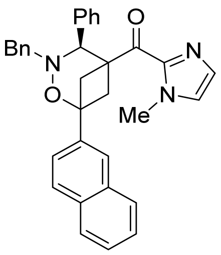

3ib
$C_{33}H_{29}N_3O_2$
M = 499.61 g/mol

(*S*)-(3-benzyl-1-(naphthalen-2-yl)-4-phenyl-2-oxa-3-azabicyclo[3.1.1]heptan-5-yl)(1-methyl-1H-imidazol-2-yl)methanone: (**3ib**) Prepared from (1-methyl-1H-imidazol-2-yl)(3-(naphthalen-2-yl)bicyclo[1.1.0]butan-1-yl)methanone (**1i**, 57.7 mg, 0.20 mmol) and (*Z*)-*N*-benzyl-1-phenylmethanimine oxide (**2b**, 50.7 mg, 0.24 mmol) according to the **GP1** at rt for 16 h. Purification by flash chromatography on silica gel using petroleum ether/EtOAc (5/1) afforded **3ib** as a white solid (83.9 mg, 84% yield).

3ib: **R_f** = 0.4 (petroleum ether/EtOAc = 3/1). Mp: 123-125 °C. HPLC analysis (Chiralpak AD-H, ^{i}PrOH/hexane = 15/85, 1.0 mL/min, 254 nm; tr (minor) = 9.25 min, tr (major) = 28.45 min) gave the isomeric composition of the product: 99% ee. $[\alpha]_D^{20}$ = +115.5 (*c* = 2.00, $CHCl_3$). **^{1}H NMR** (400 MHz, $CDCl_3$): δ 7.78-7.71 (m, 4H), 7.45-7.39 (m, 5H), 7.30 (t, *J* = 7.6 Hz, 2H), 7.25-7.23 (m, 2H), 7.16 (s, 5H), 6.88 (s, 1H), 4.89 (s, 1H), 4.02 (AB, *J* = 14.6 Hz, 1H), 3.88 (AB, *J* = 14.8 Hz, 1H), 3.72 (t, *J* = 9.4 Hz, 1H), 3.42 (s, 3H), 3.05

(d, *J* = 8.4 Hz, 1H), 2.90 (t, *J* = 9.1 Hz, 1H), 2.49 (d, *J* = 10.0 Hz, 1H) ppm. **^{13}C NMR** (100 MHz, $CDCl_3$): δ 191.6, 142.6, 138.7, 138.2, 138.1, 133.0, 132.8, 129.2, 128.8, 128.2, 128.1, 127.92, 127.90, 127.86, 127.5, 126.7, 125.94, 125.92, 125.88, 124.4, 123.6, 81.8, 74.5, 58.7, 54.2, 49.8, 35.1, 35.1 ppm. **HRMS** (ESI) *m/z*: [M+H]$^+$ calcd. for $C_{33}H_{30}N_3O_2$: 500.2333; found: 500.2322.

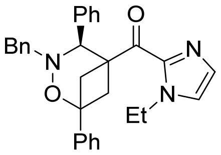

3jb
$C_{30}H_{29}N_3O_2$
M = 463.58 g/mol

(*S*)-(3-benzyl-1,4-diphenyl-2-oxa-3-azabicyclo[3.1.1]heptan-5-yl)(1-ethyl-1H-imidazol-2-yl)methanone: (**3jb**) Prepared from ((1-ethyl-1H-imidazol-2-yl)(3-phenylbicyclo[1.1.0]butan-1-yl)methanone (**1j**, 50.4 mg, 0.20 mmol) and (*Z*)-*N*-benzyl-1-phenylmethanimine oxide (**2b**, 50.6 mg, 0.24 mmol) according to the **GP1** at rt for 16 h. Purification by flash chromatography on silica gel using petroleum ether/EtOAc (5/1) afforded **3jb** as a white solid (74.1 mg, 80% yield).

3jb: **R*f*** = 0.4 (petroleum ether/EtOAc = 3/1). Mp: 107-109 ^{o}C. HPLC analysis (Chiralpak AD-H, ^{i}PrOH/hexane = 15/85, 1.0 mL/min, 254 nm; tr (minor) = 8.56 min, tr (major) = 16.27 min) gave the isomeric composition of the product: 99% ee. $[\alpha]_D^{20}$ = +78.1 (*c* = 1.75, $CHCl_3$). **^{1}H NMR** (400 MHz, $CDCl_3$): δ 7.37 (d, *J* = 7.6 Hz, 2H), 7.30-7.21 (m, 9H), 7.16-7.13 (m, 5H), 6.95 (s, 1H), 4.90 (s, 1H), 4.16-4.08 (m, 1H), 3.98 (AB, *J* = 14.8 Hz, 1H), 3.86 (AB, *J* = 15.2 Hz, 1H), 3.80-3.73 (m, 1H), 3.67 (t, *J* = 9.4 Hz, 1H), 2.94 (d, *J* = 9.6 Hz, 1H), 2.82 (t, *J* = 9.0 Hz, 1H), 2.42 (d, *J* = 10.4 Hz, 1H), 0.87 (t, *J* = 7.0 Hz, 3H) ppm. **^{13}C NMR** (100 MHz, $CDCl_3$): δ 191.4, 141.7, 141.4, 138.3, 138.2, 129.3, 128.6, 128.20, 128.15, 128.0, 127.8, 127.5, 126.6, 125.4, 124.5, 81.7, 74.5, 58.5, 54.2, 50.0, 43.2, 35.1, 15.9 ppm. **HRMS** (ESI) *m/z*: [M+H]$^+$ calcd. for $C_{30}H_{30}N_3O_2$: 464.2333; found: 464.2331.

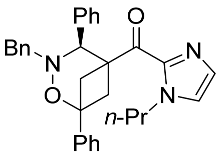

3kb
$C_{31}H_{31}N_3O_2$
M = 477.61 g/mol

(*S*)-(3-benzyl-1,4-diphenyl-2-oxa-3-azabicyclo[3.1.1]heptan-5-yl)(1-propyl-1H-imidazol-2-yl)methanone: (**3kb**) Prepared from ((1-propyl-1H-imidazol-2-yl)(3-phenylbicyclo[1.1.0]butan-1-yl)methanone (**1k**, 53.2 mg, 0.20 mmol) and (*Z*)-*N*-benzyl-1-phenylmethanimine oxide (**2b**, 50.6 mg, 0.24 mmol) according to the **GP1** at rt for 16 h. Purification by flash chromatography on silica gel using petroleum ether/EtOAc (5/1) afforded **3x** as a white solid (77.4 mg, 81% yield).

3kb: $\mathbf{R}_f$ = 0.4 (petroleum ether/EtOAc = 3/1). Mp: 115-117 °C. HPLC analysis (Chiralpak AD-H, ^{i}PrOH/hexane = 15/85, 1.0 mL/min, 254 nm; tr (minor) = 8.04 min, tr (major) = 15.44 min) gave the isomeric composition of the product: 99% ee. $[\alpha]_D^{26}$ = +61.4 (*c* = 1.88, $CHCl_3$). **^{1}H NMR** (400 MHz, $CDCl_3$): δ 7.37 (d, *J* = 7.6 Hz, 2H), 7.30-7.26 (m, 6H), 7.25-7.21 (m, 3H), 7.19-7.17 (m, 2H), 7.13-7.12 (m, 3H), 6.93 (s, 1H), 4.92 (s, 1H), 4.19-4.12 (m, 1H), 3.98 (AB, *J* = 14.8 Hz, 1H), 3.86 (AB, *J* = 14.8 Hz, 1H), 3.70-3.60 (m, 2H), 2.93 (d, *J* = 9.6 Hz, 1H), 2.81 (t, *J* = 9.0 Hz, 1H), 2.41 (d, *J* = 10.0 Hz, 1H), 1.26-1.12 (m, 2H), 0.64 (t, *J* = 7.4 Hz, 3H) ppm. **^{13}C NMR** (100 MHz, $CDCl_3$): δ 191.3, 141.7, 141.4, 138.3, 138.2, 129.1, 128.6, 128.22, 128.15, 128.1, 128.0, 127.8, 127.5, 126.6, 125.42, 125.40, 81.7, 74.5, 58.5, 54.2, 50.0, 49.8, 35.2, 23.7, 10.9 ppm. **HRMS** (ESI) *m/z*: $[M+H]^+$ calcd. for $C_{31}H_{32}N_3O_2$: 478.2489; found: 478.2484.

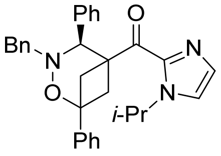

3lb
$C_{31}H_{31}N_3O_2$
M = 477.61 g/mol

(*S*)-(3-benzyl-1,4-diphenyl-2-oxa-3-azabicyclo[3.1.1]heptan-5-yl)(1-isopropyl-1H-imidazol-2-yl)methanone: (**3lb**) Prepared from ((1-isopropyl-1H-imidazol-2-yl)(3-

phenylbicyclo[1.1.0]butan-1-yl)methanone (**1l**, 53.2 mg, 0.20 mmol) and (*Z*)-*N*-benzyl-1-phenylmethanimine oxide (**2b**, 50.6 mg, 0.24 mmol) according to the **GP1** at rt for 16 h. Purification by flash chromatography on silica gel using petroleum ether/EtOAc (5/1) afforded **3lb** as a white solid (69.7 mg, 73% yield).

3lb: $\mathbf{R_f}$ = 0.4 (petroleum ether/EtOAc = 3/1). Mp: 101-103 °C. HPLC analysis (Chiralpak AD-H, ^{i}PrOH/hexane = 15/85, 1.0 mL/min, 254 nm; tr (minor) = 6.65 min, tr (major) = 21.65 min) gave the isomeric composition of the product: 98% ee. $[\alpha]_D^{20}$ = +37.3 (*c* = 1.75, $CHCl_3$). **^{1}H NMR** (400 MHz, $CDCl_3$): δ 7.37 (d, *J* = 7.6 Hz, 2H), 7.32-7.28 (m, 9H), 7.19-7.11 (m, 6H), 4.91-4.85 (m, 2H), 3.97 (AB, *J* = 14.8 Hz, 1H), 3.85 (AB, *J* = 14.8 Hz, 1H), 3.66 (t, *J* = 9.2 Hz, 1H), 2.95 (d, *J* = 9.6 Hz, 1H), 2.83 (t, *J* = 9.2 Hz, 1H), 2.43 (d, *J* = 10.0 Hz, 1H), 1.30 (d, *J* = 6.8 Hz, 3H), 0.81 (d, *J* = 5.6 Hz, 3H) ppm. **^{13}C NMR** (100 MHz, $CDCl_3$): δ 191.6, 141.7, 141.4, 138.3, 129.6, 128.6, 128.2, 128.13, 128.06, 127.9, 127.8, 127.5, 126.6, 125.4, 120.3, 81.7, 74.6, 58.4, 54.5, 50.2, 48.6, 35.1, 23.2, 23.0 ppm. **HRMS** (ESI) *m/z*: $[M+H]^+$ calcd. for $C_{31}H_{32}N_3O_2$: 478.2489; found: 478.2487.

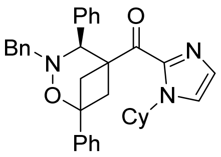

3mb
$C_{34}H_{35}N_3O_2$
M = 517.67 g/mol

(*S*)-(3-benzyl-1,4-diphenyl-2-oxa-3-azabicyclo[3.1.1]heptan-5-yl)(1-cyclohexyl-1H-imidazol-2-yl)methanone: (**3mb**) Prepared from ((1-cyclohexyl-1H-imidazol-2-yl)(3-phenylbicyclo[1.1.0]butan-1-yl)methanone (**1m**, 58.8 mg, 0.20 mmol) and (*Z*)-*N*-benzyl-1-phenylmethanimine oxide (**2b**, 50.6 mg, 0.24 mmol) according to the **GP1** at rt for 16 h. Purification by flash chromatography on silica gel using petroleum ether/EtOAc (5/1) afforded **3mb** as a white solid (72.0 mg, 71% yield).

3mb: $\mathbf{R_f}$ = 0.4 (petroleum ether/EtOAc = 3/1). Mp: 125-127 °C. HPLC analysis (Chiralpak AD-H, ^{i}PrOH/hexane = 15/85, 1.0 mL/min, 254 nm; tr (minor) = 6.51 min, tr (major) = 25.27 min) gave the isomeric composition of the product: 98% ee. $[\alpha]_D^{20}$ =

+27.5 (*c* = 1.75, $CHCl_3$). **^{1}H NMR** (400 MHz, $CDCl_3$): δ 7.37 (d, *J* = 7.6 Hz, 2H), 7.30-7.21 (m, 9H), 7.16-7.11 (m, 6H), 4.90 (s, 1H), 4.43-4.35 (m, 1H), 3.98 (AB, *J* = 14.8 Hz, 1H), 3.85 (AB, *J* = 14.8 Hz, 1H), 3.66 (t, *J* = 9.6 Hz, 1H), 2.95 (d, *J* = 9.6 Hz, 1H), 2.83 (t, *J* = 9.2 Hz, 1H), 2.43 (d, *J* = 10.0 Hz, 1H), 1.98 (d, *J* = 10.4 Hz, 1H), 1.77 (d, *J* = 12.0 Hz, 1H), 1.62 (d, *J* = 11.6 Hz, 2H), 1.40-1.26 (m, 2H), 1.17-1.06 (m, 4H) ppm. **^{13}C NMR** (100 MHz, $CDCl_3$): δ 191.7, 141.8, 141.4, 138.2, 129.4, 128.6, 128.2, 128.1, 128.0, 127.84, 127.81, 127.5, 126.6, 125.4, 120.8, 81.7, 74.6, 58.4, 56.2, 54.5, 50.2, 35.1, 34.0, 33.8, 25.40, 25.36, 25.2 ppm. **HRMS** (ESI) *m/z*: $[M+H]^+$ calcd. for $C_{34}H_{36}N_3O_2$: 518.2802; found: 518.2796.

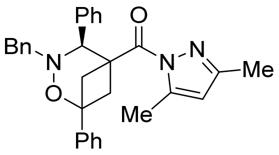

3nb

$C_{30}H_{29}N_3O_2$

M = 463.58 g/mol

(*S*)-(3-benzyl-1,4-diphenyl-2-oxa-3-azabicyclo[3.1.1]heptan-5-yl)(3,5-dimethyl-1H-pyrazol-1-yl)methanone: (**3nb**) Prepared from (3,5-dimethyl-1H-pyrazol-1-yl)(3-phenylbicyclo[1.1.0]butan-1-yl)methanone (**1n**, 50.5 mg, 0.20 mmol) and (*Z*)-*N*-benzyl-1-phenylmethanimine oxide (**2b**, 50.7 mg, 0.24 mmol) according to the **GP1** at rt for 16 h. Purification by flash chromatography on silica gel using petroleum ether/EtOAc (20/1) afforded **3nb** as a white solid (91.9 mg, 99% yield).

3nb: **R*f*** = 0.6 (petroleum ether/EtOAc = 10/1). Mp: 103-105 °C. HPLC analysis (Chiralpak AD-H, ^{i}PrOH/hexane = 2/98, 1.0 mL/min, 254 nm; tr (major) = 9.53 min, tr (minor) = 13.49 min) gave the isomeric composition of the product: 99% ee. $[\alpha]_D^{20}$ = +62.8 (*c* = 0.50, $CHCl_3$). **^{1}H NMR** (400 MHz, $CDCl_3$): δ 7.38 (d, *J* = 8.0 Hz, 2H), 7.31-7.27 (m, 6H), 7.24-7.20 (m, 2H), 7.17 (s, 5H), 5.84 (s, 1H), 4.81 (s, 1H), 4.02 (AB, *J* = 14.8 Hz, 1H), 3.84 (AB, *J* = 14.8 Hz, 1H), 3.72 (t, *J* = 9.6 Hz, 1H), 2.96 (d, *J* = 9.6 Hz, 1H), 2.74 (t, *J* = 9.4 Hz, 1H), 2.41 (d, *J* = 10.0 Hz, 1H), 2.30 (s, 3H), 2.05 (s, 3H) ppm. **^{13}C NMR** (100 MHz, $CDCl_3$): δ 172.6, 151.9, 144.0, 141.2, 138.1, 137.7, 128.7, 128.3, 128.2, 128.1, 127.8, 127.53, 127.47, 126.7, 125.3, 110.2, 81.5, 73.6, 58.7, 51.7, 50.0,

35.9, 13.9, 13.5 ppm. **HRMS** (ESI) *m/z*: $[M+H]^+$ calcd. for $C_{30}H_{30}N_3O_2$: 464.2333; found: 464.2319.

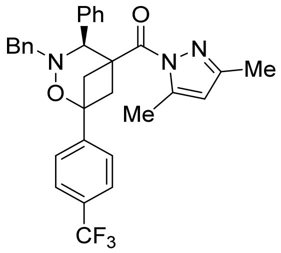

3ob
$C_{31}H_{28}F_3N_3O_2$
M = 531.58 g/mol

(*S*)-(3-benzyl-4-phenyl-1-(4-(trifluoromethyl)phenyl)-2-oxa-3-azabicyclo[3.1.1]heptan-5-yl)(3,5-dimethyl-1H-pyrazol-1-yl)methanone: (**3ob**) Prepared from (3,5-dimethyl-1H-pyrazol-1-yl)(3-(4-(trifluoromethyl)phenyl)bicyclo[1.1.0]butan-1-yl)methanone (**1o**, 64.0 mg, 0.20 mmol) and (*Z*)-*N*-benzyl-1-phenylmethanimine oxide (**2b**, 50.7 mg, 0.24 mmol) according to the **GP1** at rt for 16 h. Purification by flash chromatography on silica gel using petroleum ether/EtOAc (20/1) afforded **3ob** as a colorless oil (89.0 mg, 84% yield).

3ob: R_f = 0.6 (petroleum ether/EtOAc = 10/1). HPLC analysis (Chiralpak OD-H, ^{i}PrOH/hexane = 2/98, 0.8 mL/min, 254 nm; tr (major) = 5.83 min, tr (minor) = 6.52 min) gave the isomeric composition of the product: 92% ee. $[\alpha]_D^{20}$ = +49.0 (*c* = 1.00, $CHCl_3$). **^{1}H NMR** (400 MHz, $CDCl_3$): δ 7.54 (d, *J* = 8.0 Hz, 2H), 7.38 (d, *J* = 8.0 Hz, 4H), 7.31 (d, *J* = 7.2 Hz, 2H), 7.26-7.23 (m, 1H), 7.20-7.16 (m, 5H), 5.86 (s, 1H), 4.82 (s, 1H), 4.02 (AB, *J* = 14.4 Hz, 1H), 3.84 (AB, *J* = 14.4 Hz, 1H), 3.72 (t, *J* = 9.6 Hz, 1H), 2.97 (d, *J* = 10.0 Hz, 1H), 2.76 (t, *J* = 9.2 Hz, 1H), 2.41 (d, *J* = 10.0 Hz, 1H), 2.31 (s, 3H), 2.06 (s, 3H) ppm. **^{13}C NMR** (100 MHz, $CDCl_3$): δ 172.3, 152.2, 144.9, 144.1, 137.9, 137.4, 129.7 (q, *J* = 32 Hz), 128.7, 128.41, 128.38, 127.9, 127.4, 126.8, 125.7, 125.2 (q, *J* = 4 Hz), 124.1 (q, *J* = 270 Hz), 110.4, 81.1, 73.7, 58.8, 51.8, 50.2, 36.1, 13.9, 13.5 ppm. **^{19}F NMR** (376 MHz, $CDCl_3$) δ -62.528 ppm. **HRMS** (ESI) *m/z*: $[M+H]^+$ calcd. for $C_{31}H_{28}F_3N_3O_2$: 532.2206; found: 532.2213.

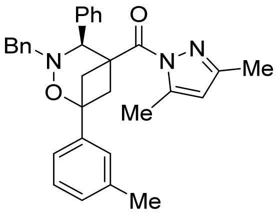

3pb
$C_{31}H_{31}N_3O_2$
M = 477.61 g/mol

(*S*)-(3-benzyl-4-phenyl-1-(m-tolyl)-2-oxa-3-azabicyclo[3.1.1]heptan-5-yl)(3,5-dimethyl-1H-pyrazol-1-yl)methanone: (**3pb**) Prepared from (3,5-dimethyl-1H-pyrazol-1-yl)(3-(m-tolyl)bicyclo[1.1.0]butan-1-yl)methanone (**1p**, 53.2 mg, 0.20 mmol) and (*Z*)-*N*-benzyl-1-phenylmethanimine oxide (**2b**, 50.7 mg, 0.24 mmol) according to the **GP1** at rt for 16 h. Purification by flash chromatography on silica gel using petroleum ether/EtOAc (20/1) afforded **3pb** as a colorless oil (95.4 mg, 99% yield).

3pb: $\mathbf{R}_f$ = 0.6 (petroleum ether/EtOAc = 10/1). HPLC analysis (Chiralpak AD-H, ^{i}PrOH/hexane = 2/98, 1.0 mL/min, 254 nm; tr (major) = 6.69 min, tr (minor) = 9.00 min) gave the isomeric composition of the product: 99.5% ee. $[\alpha]_D^{20}$ = +58.0 (*c* = 1.25, $CHCl_3$). **^{1}H NMR** (400 MHz, $CDCl_3$): δ 7.31 (d, *J* = 7.6 Hz, 2H), 7.22 (t, *J* = 7.6 Hz, 2H), 7.16-7.11 (m, 2H), 7.08 (s, 5H), 7.02-6.95 (m, 3H), 5.75 (s, 1H), 4.72 (s, 1H), 3.94 (AB, *J* = 14.8 Hz, 1H), 3.76 (AB, *J* = 14.8 Hz, 1H), 3.62 (t, *J* = 9.6 Hz, 1H), 2.88 (d, *J* = 10.0 Hz, 1H), 2.65 (t, *J* = 9.2 Hz, 1H), 2.31 (d, *J* = 10.0 Hz, 1H), 2.22 (s, 3H), 2.20 (s, 3H), 1.97 (s, 3H) ppm. **^{13}C NMR** (100 MHz, $CDCl_3$): δ 172.6, 151.9, 144.0, 141.1, 138.1, 137.74, 137.69, 128.8, 128.3, 128.2, 128.1, 127.8, 127.5, 126.7, 126.2, 122.4, 110.2, 81.5, 73.5, 58.7, 51.8, 49.9, 36.0, 21.4, 13.9, 13.5 ppm. **HRMS** (ESI) *m/z*: $[M+H]^+$ calcd. for $C_{31}H_{32}N_3O_2$: 478.2489; found: 478.2501.

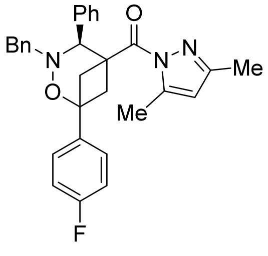

3qb
$C_{30}H_{28}FN_3O_2$
M = 481.57 g/mol

(*S*)-(3-benzyl-1-(4-fluorophenyl)-4-phenyl-2-oxa-3-azabicyclo[3.1.1]heptan-5-yl)(3,5-dimethyl-1H-pyrazol-1-yl)methanone: (**3qb**) Prepared from (3,5-dimethyl-1H-pyrazol-1-yl)(3-(4-fluorophenyl)bicyclo[1.1.0]butan-1-yl)methanone (**1q**, 54.0 mg, 0.20 mmol) and (*Z*)-*N*-benzyl-1-phenylmethanimine oxide (**2b**, 50.7 mg, 0.24 mmol) according to the **GP1** at rt for 16 h. Purification by flash chromatography on silica gel using petroleum ether/EtOAc (20/1) afforded **3qb** as a colorless oil (93.4 mg, 97% yield).

3qb: **R*f*** = 0.6 (petroleum ether/EtOAc = 10/1). HPLC analysis (Chiralpak AD-H, ^{i}PrOH/hexane = 1/99, 1.0 mL/min, 254 nm; tr (major) = 12.55 min, tr (minor) = 16.69 min) gave the isomeric composition of the product: 99% ee. $[\alpha]_D^{20}$ = +56.7 (*c* = 1.50, $CHCl_3$). **^{1}H NMR** (400 MHz, $CDCl_3$): δ 7.37 (d, *J* = 6.8 Hz, 2H), 7.30 (t, *J* = 7.4 Hz, 2H), 7.26-7.22 (m, 3H), 7.16 (s, 5H), 6.96 (t, *J* = 8.8 Hz, 2H), 5.85 (s, 1H), 4.80 (s, 1H), 4.01 (AB, *J* = 14.8 Hz, 1H), 3.83 (AB, *J* = 14.8 Hz, 1H), 3.70 (t, *J* = 9.6 Hz, 1H), 2.93 (d, *J* = 10.0 Hz, 1H), 2.73 (t, *J* = 9.4 Hz, 1H), 2.37 (d, *J* = 10.4 Hz, 1H), 2.31 (s, 3H), 2.05 (s, 3H) ppm. **^{13}C NMR** (100 MHz, $CDCl_3$): δ 172.4, 162.2 (d, *J* = 244 Hz), 152.0, 144.1, 138.0, 137.6, 137.1 (d, *J* = 3 Hz), 128.7, 128.3, 127.9, 127.5, 127.1 (d, *J* = 8 Hz), 126.7, 115.0 (d, *J* = 22 Hz), 110.3, 81.1, 73.6, 58.7, 51.7, 50.0, 36.0, 13.9, 13.5 ppm. **^{19}F NMR** (376 MHz, $CDCl_3$) δ -114.853 ppm. **HRMS** (ESI) *m/z*: $[M+H]^+$ calcd. for $C_{30}H_{29}FN_3O_2$: 482.2238; found: 482.2227.

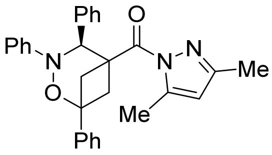

3nd

$C_{29}H_{27}N_3O_2$

M = 449.55 g/mol

(*S*)-(3,5-dimethyl-1H-pyrazol-1-yl)(1,3,4-triphenyl-2-oxa-3-azabicyclo[3.1.1]heptan-5-yl)methanone: (**3nd**)[3a] Prepared from (3,5-dimethyl-1H-pyrazol-1-yl)(3-phenylbicyclo[1.1.0]butan-1-yl)methanone (**1n**, 50.5 mg, 0.20 mmol) and (*Z*)-N,1-diphenylmethanimine oxide (**2d**, 47.3 mg, 0.24 mmol) according to the **GP1** at rt for 16 h. Purification by flash chromatography on silica gel using petroleum ether/EtOAc (20/1) afforded **3nd** as a white solid (48.0 mg, 53% yield).

3nd: **R$_f$** = 0.6 (petroleum ether/EtOAc = 10/1). HPLC analysis (Chiralpak AD-H, ^{i}PrOH/hexane = 2/98, 1.0 mL/min, 254 nm; tr (major) = 9.29 min, tr (minor) = 10.91 min) gave the isomeric composition of the product: 28% ee. $[\alpha]_D^{20}$ = +14.9 (*c* = 1.20, $CHCl_3$).**^{1}H NMR** (400 MHz, $CDCl_3$): δ 7.50-7.48 (m,2H), 7.43-7.40 (m, 2H), 7.36-7.35 (m, 1H), 7.21-7.16 (m, 7H), 7.07-7.05 (m, 2H), 6.89-6.86 (m, 1H), 5.94 (s, 2H), 3.25 (t, *J* = 9.4 Hz, 1H), 3.18 (t, *J* = 9.4 Hz, 1H), 2.77 (d, *J* = 10.0 Hz, 1H), 2.58 (d, *J* = 10.4 Hz, 1H), 2.31 (s, 3H), 2.25 (s, 3H) ppm. **^{13}C NMR** (100 MHz, $CDCl_3$): δ 172.0, 152.4, 150.0, 144.30, 140.29, 138.3, 128.9, 128.5, 128.4, 128.3, 128.2, 127.1, 125.9, 121.5, 116.7, 110.7, 83.5, 70.2, 51.8, 45.3, 36.6, 13.97, 13.91 ppm.

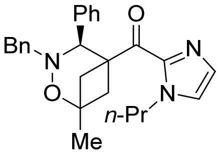

3rb
$C_{26}H_{29}N_3O_2$
M = 415.54 g/mol

(*S*)-(3-benzyl-1-methyl-4-phenyl-2-oxa-3-azabicyclo[3.1.1]heptan-5-yl)(1-propyl-1H-imidazol-2-yl)methanone: (**3rb**) Prepared from (3-methylbicyclo[1.1.0]butan-1-yl)(1-propyl-1H-imidazol-2-yl)methanone (**1r**, 40.8 mg, 0.20 mmol) and (*Z*)-*N*-benzyl-1-phenylmethanimine oxide (**2b**, 50.6 mg, 0.24 mmol) according to the **GP1** at rt for 16 h. Purification by flash chromatography on silica gel using petroleum ether/EtOAc (5/1) afforded **3rb** as a colorless oil (25.0 mg, 30% yield).

3rb: **R$_f$** = 0.6 (petroleum ether/EtOAc = 3/1). HPLC analysis (Chiralpak AD-H, ^{i}PrOH/hexane = 10/90, 1.0 mL/min, 254 nm; tr (minor) = 5.67 min, tr (major) = 14.57 min) gave the isomeric composition of the product: 24% ee. $[\alpha]_D^{20}$ = +5.1 (*c* = 0.51, $CHCl_3$). **^{1}H NMR** (400 MHz, $CDCl_3$): δ 7.37-7.31 (m, 2H), 7.26 (t, *J* = 7.6 Hz, 2H), 7.21-7.18 (m, 2H), 7.12-7.09 (m, 5H), 6.90 (s, 1H), 4.77 (s, 1H), 4.14-4.07 (m, 1H), 3.89 (AB, *J* = 14.8 Hz, 1H), 3.75 (AB, *J* = 14.8 Hz, 1H), 3.64-3.57 (m, 1H), 3.30 (t, *J* = 9.4 Hz, 1H), 2.51 (t, *J* = 9.2 Hz, 1H), 2.32 (d, *J* = 9.6 Hz, 1H), 2.10 (d, *J* = 10.0 Hz, 1H), 1.27 (s, 3H), 1.21-1.11 (m, 2H), 0.63 (t, *J* = 7.4 Hz, 3H) ppm. **^{13}C NMR** (100 MHz, $CDCl_3$): δ 191.6, 141.7, 138.4, 138.4, 129.0, 128.6, 128.1, 128.0, 127.82, 127.80, 126.5, 125.2,

79.1, 74.3, 58.5, 54.6, 51.3, 49.8, 33.6, 23.7, 23.4, 10.9 ppm. **HRMS** (ESI) *m/z*: $[M+Na]^+$ calcd. for $C_{26}H_{29}N_3O_2Na$: 438.2152; found: 438.2158.

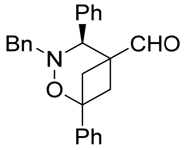

4nb
$C_{25}H_{23}NO_2$
M = 369.46 g/mol

(*S*)-3-benzyl-1,4-diphenyl-2-oxa-3-azabicyclo[3.1.1]heptane-5-carbaldehyde (**4nb**): Prepared from (*S*)-(3-benzyl-1,4-diphenyl-2-oxa-3-azabicyclo[3.1.1]heptan-5-yl)(3,5-dimethyl-1H-pyrazol-1-yl)methanone (**3nb**, 46.3 mg, 0.1 mmol) according to the **GP3** at -40 °C for 30 min. Purification by flash chromatography on silica gel using petroleum ether/ethyl acetate (10/1) afforded **4nb** as a colorless oil (20.0 mg, 54% yield).

4nb: $\mathbf{R_f}$ = 0.4 (petroleum ether/EtOAc = 10/1). HPLC analysis (Chiralpak AD-H, ^{i}PrOH/hexane = 2/98, 1.0 mL/min, 254 nm; tr (minor) = 7.54 min, tr (major) = 8.66 min) gave the isomeric composition of the product: 99% ee. $[\alpha]_D^{20}$ = +99.0 (*c* = 0.50, $CHCl_3$). ^{1}**H NMR** (600 MHz, $CDCl_3$): δ 9.23 (s, 1H), 7.53-7.52 (m, 2H), 7.40-7.38 (m, 2H), 7.36-7.33 (m, 3H), 7.32-7.28 (m, 6H), 7.27-7.23 (m, 2H), 4.29 (s, 1H), 4.13 (AB, *J* = 14.8 Hz, 1H), 3.81 (AB, *J* = 14.8 Hz, 1H), 3.53 (t, *J* = 9.6 Hz, 1H), 2.69 (d, *J* = 9.6 Hz, 1H), 2.30 (d, *J* = 9.6 Hz, 1H), 2.06 (t, *J* = 9.0 Hz, 1H) ppm. ^{13}**C NMR** (150 MHz, $CDCl_3$): δ 200.5, 140.7, 137.28, 137.25, 129.2, 129.1, 128.9, 128.3, 128.0, 127.94, 127.89, 127.0, 125.3, 81.7, 72.0, 58.1, 52.2, 45.8, 35.1 ppm. **HRMS** (ESI) *m/z*: $[M+Na]^+$ calcd. for $C_{25}H_{23}NO_2Na$: 392.1621; found: 392.1624.

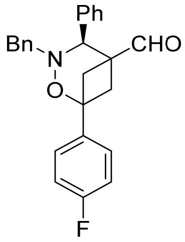

4qb
$C_{25}H_{22}FNO_2$
M = 387.45 g/mol

(*S*)-3-benzyl-1-(4-fluorophenyl)-4-phenyl-2-oxa-3-azabicyclo[3.1.1]heptane-5-carbaldehyde (**4qb**): Prepared from (*S*)-(3-benzyl-1-(4-fluorophenyl)-4-phenyl-2-oxa-3-azabicyclo[3.1.1]heptan-5-yl)(3,5-dimethyl-1H-pyrazol-1-yl)methanone (**3qb**, 48.1 mg, 0.1 mmol) according to the **GP3** at -40 °C for 30 min. Purification by flash chromatography on silica gel using petroleum ether/ethyl acetate (10/1) afforded **4qb** as a colorless oil (23.3 mg, 60% yield).

(**4qb**): $\mathbf{R}_f$ = 0.4 (petroleum ether/EtOAc = 10/1). $[\alpha]_D^{20}$ = +133.6 (*c* = 0.70, $CHCl_3$). **^{1}H NMR** (400 MHz, $CDCl_3$): δ 9.22 (s, 1H), 7.52 (d, *J* = 7.2 Hz, 2H), 7.41-7.28 (m, 7H), 7.26-7.22 (m, 3H), 6.98 (t, *J* = 8.6 Hz, 2H), 4.28 (s, 1H), 4.11 (AB, *J* = 14.8 Hz, 1H), 3.79 (AB, *J* = 14.8 Hz, 1H), 3.51 (t, *J* = 9.2 Hz, 1H), 2.66 (d, *J* = 9.2 Hz, 1H), 2.26 (d, *J* = 9.6 Hz, 1H), 2.05 (t, *J* = 9.2 Hz, 1H) ppm. **^{13}C NMR** (100 MHz, $CDCl_3$): δ 200.4, 162.4 (d, J = 146 Hz), 137.2 (d, *J* = 7 Hz), 136.6 (d, *J* = 3 Hz), 129.2, 129.0, 128.0, 127.2, 127.1, 127.0, 115.1 (d, *J* = 21 Hz), 81.2, 72.0, 58.1, 52.1, 45.8, 35.3 ppm. **^{19}F NMR** (376 MHz, $CDCl_3$) δ -114.222 ppm. **HRMS** (ESI) *m/z*: $[M+H]^+$ calcd. for $C_{25}H_{23}FNO_2$: 388.1707; found: 388.1702.

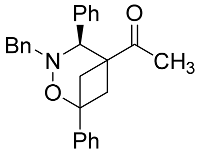

5

$C_{26}H_{25}NO_2$

M = 383.49 g/mol

(*S*)-1-(3-benzyl-1,4-diphenyl-2-oxa-3-azabicyclo[3.1.1]heptan-5-yl)ethan-1-one (**5**): $\mathbf{R}_f$ = 0.3 (petroleum ether/EtOAc = 10/1). HPLC analysis (Chiralpak AD-H, ^{i}PrOH/hexane = 10/90, 1.0 mL/min, 254 nm; tr (minor) = 5.06 min, tr (major) = 6.24 min) gave the isomeric composition of the product: 99% ee. $[\alpha]_D^{20}$ = +53.2 (*c* = 0.58, $CHCl_3$). **^{1}H NMR** (400 MHz, $CDCl_3$): δ 7.50-7.47 (m, 2H), 7.37-7.23 (m, 13H), 4.25 (s, 1H), 4.09 (AB, *J* = 14.8 Hz, 1H), 3.79 (d, *J* = 14.8 Hz, 1H), 3.60 (t, *J* = 9.4 Hz, 1H), 2.59 (d, *J* = 9.6 Hz, 1H), 2.29 (d, *J* = 10.0 Hz, 1H), 2.21 (t, *J* = 9.2 Hz, 1H), 1.68 (s, 3H) ppm. **^{13}C NMR** (100 MHz, $CDCl_3$): δ 208.8, 140.8, 137.5, 137.4, 129.0, 128.9, 128.8, 128.3, 128.2,

127.9, 127.8, 126.9, 125.3, 80.6, 73.2, 58.3, 54.4, 47.4, 35.3, 26.6 ppm. **HRMS** (ESI) *m/z*: $[M+H]^+$ calcd. for $C_{26}H_{26}NO_2$: 384.1958; found: 384.1950.

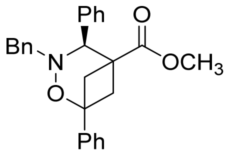

6

$C_{26}H_{25}NO_3$
M = 399.49 g/mol

Methyl (*S*)-3-benzyl-1,4-diphenyl-2-oxa-3-azabicyclo[3.1.1]heptane-5-carboxylate (**6**): **R*f*** = 0.6 (petroleum ether/EtOAc = 10/1). HPLC analysis (Chiralpak AD-H, *i*PrOH/hexane = 2/98, 1.0 mL/min, 254 nm; tr (minor) = 9.25 min, tr (major) = 10.40 min) gave the isomeric composition of the product: 98% ee. $[\alpha]_D^{20}$ = +65.4 (*c* = 1.00, $CHCl_3$). **^{1}H NMR** (400 MHz, $CDCl_3$): δ 7.50 (d, *J* = 6.4 Hz, 2H), 7.36-7.22 (m, 13H), 4.24 (s, 1H), 4.09 (AB, *J* = 14.8 Hz, 1H), 3.80 (AB, *J* = 14.8 Hz, 1H), 3.58 (t, *J* = 9.6 Hz, 1H), 3.31 (s, 3H), 2.72 (d, *J* = 9.4 Hz, 1H), 2.37 (d, *J* = 10.0 Hz, 1H), 2.22 (t, *J* = 9.2 Hz, 1H) ppm. **^{13}C NMR** (100 MHz, $CDCl_3$): δ 172.7, 140.8, 137.9, 137.4, 129.0, 128.5, 128.4, 128.2, 128.1, 127.9, 127.7, 126.9, 125.3, 81.3, 73.1, 58.6, 51.3, 48.6, 47.7, 35.7 ppm. **HRMS** (ESI) *m/z*: $[M+H]^+$ calcd. for $C_{26}H_{26}NO_3$: 400.1907; found: 400.1901.

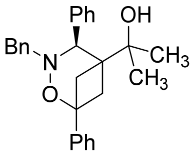

7

$C_{27}H_{29}NO_2$
M = 399.53 g/mol

(*S*)-2-(3-benzyl-1,4-diphenyl-2-oxa-3-azabicyclo[3.1.1]heptan-5-yl)propan-2-ol (**7**): **R*f*** = 0.3 (petroleum ether/EtOAc = 10/1). HPLC analysis (Chiralpak AD-H, *i*PrOH/hexane = 5/95, 0.8 mL/min, 254 nm; tr (minor) = 12.11 min, tr (major) = 12.63 min) gave the isomeric composition of the product: 99% ee. $[\alpha]_D^{20}$ = +22.0 (*c* = 0.75, $CHCl_3$). **^{1}H NMR** (400 MHz, $CDCl_3$): δ 7.60 (s, 1H), 7.38-7.21 (m, 14H), 4.09 (s, 1H), 3.98 (AB, *J* = 14.6 Hz, 1H), 3.66 (d, *J* = 14.8 Hz, 1H), 3.32 (t, *J* = 9.2 Hz, 1H), 2.82 (d, *J* = 9.6 Hz, 1H), 2.22 (d, *J* = 10.0 Hz, 1H), 1.79 (t, *J* = 9.0 Hz, 1H), 1.12 (s, 3H), 0.65 (s, 3H) ppm. **^{13}C NMR**

(100 MHz, $CDCl_3$): δ 142.1, 140.0, 138.0, 130.0, 129.1, 128.6, 128.1, 127.8, 127.4, 126.7, 125.4, 80.0, 74.9, 72.0, 58.4, 50.7, 43.8, 33.2, 26.4, 25.4 ppm. **HRMS** (ESI) *m/z*: $[M+H]^+$ calcd. for $C_{27}H_{30}NO_2$: 400.2271; found: 400.2263.

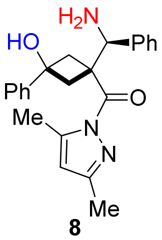

8

$C_{23}H_{25}N_3O_2$
M = 375.47 g/mol

(*S*)-(1-(amino(phenyl)methyl)-3-hydroxy-3-phenylcyclobutyl)(3,5-dimethyl-1H-pyrazol-1-yl)methanone (**8**): **R_f** = 0.5 (petroleum ether/EtOAc = 1/1). $[\alpha]_D^{20}$ = +32.7 (*c* = 0.30, $CHCl_3$). **^{1}H NMR** (400 MHz, $CDCl_3$): δ 7.47 (d, *J* = 8.4, 2H), 7.32 (t, *J* = 7.4 Hz, 2H), 7.22-7.20 (m, 4H), 6.93-6.91 (m, 2H), 5.87 (s, 1H), 5.06 (s, 1H), 3.48 (d, *J* = 14.0 Hz, 1H), 3.26 (d, *J* = 14.4 Hz, 1H), 3.04 (dd, *J* = 14.4, 4.4 Hz, 1H), 2.85 (dd, *J* = 14.4, 4.0 Hz, 1H), 2.26 (s, 3H), 2.17 (s, 3H) ppm. **^{13}C NMR** (100 MHz, $CDCl_3$): δ 174.5, 151.5, 146.1, 144.5, 143.1, 128.6, 128.1, 127.8, 126.6, 125.8, 125.0, 110.2, 71.2, 57.6, 50.4, 50.3, 40.3, 14.02, 13.95 ppm. **HRMS** (ESI) *m/z*: $[M+K]^+$ calcd. for $C_{23}H_{25}N_3O_2K$: 414.1578; found: 414.1582.

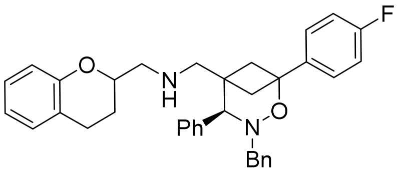

9

$C_{35}H_{35}FN_2O_2$
M = 534.68 g/mol

***N*-(((*S*)-3-benzyl-1-(4-fluorophenyl)-4-phenyl-2-oxa-3-azabicyclo[3.1.1]heptan-5-yl)methyl)-1-(chroman-2-yl)methanamine** (**9**): **R_f** = 0.3 (petroleum ether/EtOAc = 3/1). $[\alpha]_D^{20}$ = +50.8 (*c* = 0.25, $CHCl_3$). **^{1}H NMR** (600 MHz, $CDCl_3$): δ 7.55 (d, *J* = 7.2 Hz, 2H), 7.35-7.30 (m, 5H), 7.28-7.25 (m, 4H), 7.22 (t, *J* = 7.2 Hz, 1H), 7.10-7.07 (m, 1H), 7.03 (d, *J* = 7.8 Hz, 1H), 6.97 (t, *J* = 8.4 Hz, 2H), 6.83 (t, *J* = 7.8 Hz, 1H), 6.78 (t, *J* = 9.0 Hz, 1H), 4.08 (s, 1H), 4.04-3.92 (m, 2H), 3.76-3.73 (m, 1H), 3.35-3.31 (m, 1H), 2.84-2.78 (m, 1H), 2.72-

2.69 (m, 1H), 2.60-2.53 (m, 1H), 2.50-2.46 (m, 1H), 2.30-2.22 (m, 2H), 1.98-1.94 (m, 1H), 1.89-1.83 (m, 2H), 1.74-1.65 (m, 1H) ppm. **^{13}C NMR** (100 MHz, $CDCl_3$): δ 162.2 (d, J = 244 Hz) 154.6, 139.1, 139.0, 138.1, 137.9, 129.6, 129.5, 128.9, 128.50, 128.45, 128.12, 128.09, 127.8, 127.21, 127.18, 127.15, 127.13, 126.7, 122.0, 120.1, 116.7, 116.6, 115.0, 114.8, 81.9, 81.8, 75.3, 75.3, 75.1, 74.9, 59.1, 55.0, 54.73, 54.68, 54.6, 46.0, 45.9, 43.6, 43.5, 36.7, 36.5, 25.5, 25.4, 24.6 ppm. **^{19}F NMR** (565 MHz, $CDCl_3$) δ -115.186 ppm. **HRMS** (ESI) *m/z*: $[M+Na]^+$ calcd. for $C_{35}H_{35}FN_2O_2$: 535.2755; found: 535.2744.

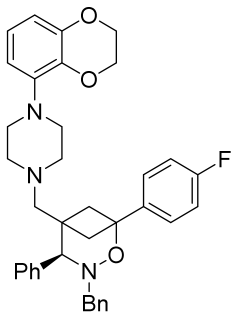

10
$C_{37}H_{38}FN_3O_3$
M = 591.73 g/mol

(*S*)-3-benzyl-5-((4-(2,3-dihydrobenzo[b][1,4]dioxin-5-yl)piperazin-1-yl)methyl)-1-(4-fluorophenyl)-4-phenyl-2-oxa-3-azabicyclo[3.1.1]heptane (**10**): **R_f** = 0.2 (petroleum ether/EtOAc = 10/1). $[\alpha]_D^{20}$ = +24.5 (c = 0.20, $CHCl_3$). **^{1}H NMR** (600 MHz, $CDCl_3$): δ 7.58 (d, J = 6.6 Hz, 2H), 7.35-7.31 (m, 4H), 7.29-7.20 (m, 6H), 6.96 (t, J = 9.0 Hz, 2H), 6.78 (t, J = 7.8 Hz, 1H), 6.59 (dd, J = 8.4, 1.2 Hz, 1H), 6.54 (dd, J = 7.8, 1.2 Hz, 1H), 4.29-4.28 (m, 2H), 4.23-4.22 (m, 2H), 4.16 (s, 1H), 4.03 (AB, J = 14.8 Hz, 1H), 3.75 (AB, J = 14.8 Hz, 1H), 3.32 (t, J = 9.0 Hz, 1H), 2.98 (s, 4H), 2.56 (s, 2H), 2.50 (d, J = 9.0 Hz, 1H), 2.14-2.06 (m, 4H), 1.98 (d, J = 13.2 Hz, 1H), 1.79 (d, J = 9.6 Hz, 1H) ppm. **^{13}C NMR** (150 MHz, $CDCl_3$): δ 162.1 (d, J = 243 Hz), 144.1, 141.9, 139.2, 138.3, 137.9 (d, J = 5 Hz), 136.4, 129.4, 128.8, 127.9, 127.8, 127.7, 127.1 (d, J = 9 Hz), 126.6, 120.6, 114.9 (d, J = 21 Hz), 111.8, 110.5, 82.5, 75.6, 64.3, 63.9, 63.6, 59.1, 54.1, 50.9, 48.5, 43.1, 37.3 ppm. **^{19}F NMR** (565 MHz, $CDCl_3$) δ -115.24 ppm. **HRMS** (ESI) *m/z*: $[M+Na]^+$ calcd. for $C_{37}H_{38}FN_3O_3Na$: 614.2789; found: 614.2775.

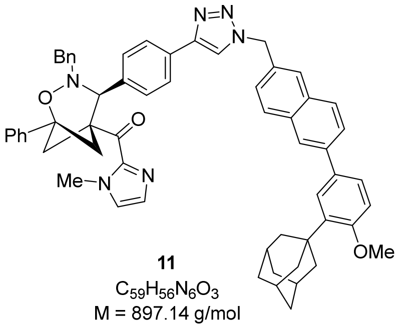

11
$C_{59}H_{56}N_6O_3$
M = 897.14 g/mol

((4*S*)-4-(4-(1-((6-(3-(adamantan-1-yl)-4-methoxyphenyl)naphthalen-2-yl)methyl)-1H-1,2,3-triazol-4-yl)phenyl)-3-benzyl-1-phenyl-2-oxa-3-azabicyclo[3.1.1]heptan-5-yl)(1-methyl-1H-imidazol-2-yl)methanone (**11**): Prepared from (*S*)-(3-benzyl-4-(4-ethynylphenyl)-1-phenyl-2-oxa-3-azabicyclo[3.1.1]heptan-5-yl)(1-methyl-1H-imidazol-2-yl)methanone (**3as**, 20.0 mg, 0.042 mmol) and 1-(5-(6-(azidomethyl)naphthalen-2-yl)-2-methoxyphenyl)adamantane (26.7 mg, 0.063 mmol) according to the **GP4** at rt for 5 h. Purification by flash chromatography on silica gel using petroleum ether/EtOAc (4/1) afforded **11** as a white solid (37.7 mg, 99% yield).

11: $\mathbf{R}_f$ = 0.2 (petroleum ether/EtOAc = 2/1). $[\alpha]_D^{20}$ = +0.9 (*c* = 1.00, $CHCl_3$). Mp: 141-143 °C. **^{1}H NMR** (400 MHz, $CDCl_3$): δ 7.98 (s, 1H), 7.87 (t, *J* = 9.8 Hz, 2H), 7.78-7.76 (m, 2H), 7.62-7.57 (m, 4H), 7.52 (d, *J* = 8.4 Hz, 1H), 7.38-7.33 (m, 3H), 7.29-7.24 (m, 7H), 7.20-7.17 (m, 4H), 6.99 (d, *J* = 8.4 Hz, 1H), 6.89 (s, 1H), 5.70 (d, *J* = 4.0 Hz, 2H), 4.89 (s, 1H), 3.98 (AB, *J* = 14.8 Hz, 1H), 3.89 (s, 3H), 3.85 (AB, *J* = 14.8 Hz, 1H), 3.63 (t, *J* = 9.4 Hz, 1H), 3.45 (s, 3H), 2.90 (d, *J* = 9.6 Hz, 1H), 2.79 (t, *J* = 9.2 Hz, 1H), 2.41 (d, *J* = 10.0 Hz, 1H), 2.18 (s, 6H), 2.10 (s, 3H), 1.80 (s, 6H) ppm. **^{13}C NMR** (100 MHz, $CDCl_3$): δ 191.4, 158.8, 147.8, 142.5, 141.3, 140.0, 139.0, 138.2, 138.0, 133.4, 132.6, 131.9, 131.4, 130.3, 129.3, 129.2, 128.7, 128.4, 128.24, 128.16, 127.84, 127.55, 127.2, 126.7, 126.6, 126.1, 125.9, 125.6, 125.6, 125.44, 125.40, 124.8, 119.5, 112.1, 81.7, 74.0, 58.6, 55.2, 54.5, 54.1, 49.7, 40.6, 37.1, 35.3, 34.9, 29.1 ppm. **HRMS** (ESI) *m/z*: $[M-H]^-$ calcd. for $C_{59}H_{55}N_6O_3$: 895.4341; found: 895.4353.

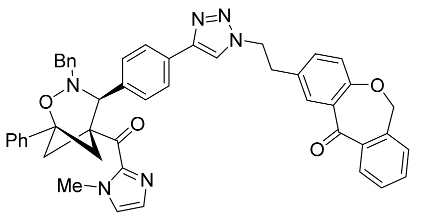

12
$C_{47}H_{40}N_6O_4$
M = 752.88 g/mol

(*S*)-2-(2-(4-(4-(3-benzyl-5-(1-methyl-1H-imidazole-2-carbonyl)-1-phenyl-2-oxa-3-azabicyclo[3.1.1]heptan-4-yl)phenyl)-1H-1,2,3-triazol-1-yl)ethyl)dibenzo[b,e]oxepin-11(6H)-one (**12**): Prepared from (*S*)-(3-benzyl-4-(4-ethynylphenyl)-1-phenyl-2-oxa-3-azabicyclo[3.1.1]heptan-5-yl)(1-methyl-1H-imidazol-2-yl)methanone (**3as**, 20.0 mg, 0.042 mmol) and 2-(2-azidoethyl)dibenzo[b,e]oxepin-11(6H)-one (17.6 mg, 0.063 mmol) according to the **GP4** at rt for 5 h. Purification by flash chromatography on silica gel using EtOAc afforded **12** as a colorless oil (31.6 mg, 99% yield).

12: $\mathbf{R_f}$ = 0.2 (petroleum ether/EtOAc = 1/1). $[\alpha]_D^{20}$ = +10.0 (*c* = 0.50, $CHCl_3$). **^{1}H NMR** (600 MHz, $CDCl_3$): δ 8.07 (d, *J* = 2.4 Hz, 1H), 7.87 (d, *J* = 7.8, 1H), 7.58-7.55 (m, 3H), 7.50-7.47 (m, 2H), 7.37-7.36 (m, 3H), 7.31-7.28 (m, 5H), 7.26-7.16 (m, 7H), 6.97 (d, *J* = 8.4 Hz, 1H), 6.90 (s, 1H), 5.18 (s, 2H), 4.90 (s, 1H), 4.62 (t, *J* = 7.2 Hz, 2H), 4.01 (AB, *J* = 14.8 Hz, 1H), 3.87 (AB, *J* = 14.8 Hz, 1H), 3.65 (t, *J* = 9.0 Hz, 1H), 3.46 (s, 3H), 3.26 (t, *J* = 7.2 Hz, 2H), 2.92 (d, *J* = 9.6 Hz, 1H), 2.81 (t, *J* = 9.0 Hz, 1H), 2.43 (d, *J* = 9.6 Hz, 1H) ppm. **^{13}C NMR** (100 MHz, $CDCl_3$): δ 191.5, 190.9, 160.4, 147.2, 142.5, 141.3, 140.3, 138.12, 138.06, 135.8, 135.5, 132.9, 131.6, 130.6, 130.3, 129.5, 129.3, 129.2, 128.7, 128.4, 128.2, 127.9, 127.6, 126.7, 126.1, 125.5, 125.4, 125.3, 121.3, 119.8, 81.7, 74.0, 73.6, 58.6, 54.1, 51.5, 49.7, 35.7, 35.3, 35.0 ppm. **HRMS** (ESI) *m/z*: $[M+K]^+$ calcd. for $C_{47}H_{40}N_6O_3K$: 791.2743; found: 791.2746.

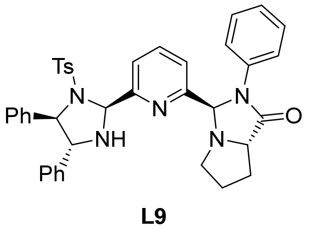

L9
$C_{39}H_{37}N_5O_3S$
M = 655.82 g/mol

(3*R*,7a*S*)-3-(6-((2*S*,4*R*,5*R*)-4,5-diphenyl-1-tosylimidazolidin-2-yl)pyridin-2-yl)-2-phenylhexahydro-1H-pyrrolo[1,2-c]imidazol-1-one: (**L9**) Prepared from (*S*)-*N*-phenylpyrrolidine-2-carboxamide (247.3 mg, 1.3 mmol, 1.0 equiv) according to the **GP5**. Purification by flash chromatography on silica gel afforded **L9** as a white solid (385 mg, 45% yield over 2 steps).

R*f* = 0.5 (petroleum ether/EtOAc = 1/3). **^{1}H NMR** (400 MHz, $CDCl_3$): δ 7.92 (d, *J* = 7.6 Hz, 1H), 7.83 (t, *J* = 7.6 Hz, 1H), 7.61 (d, *J* = 8.0 Hz, 2H), 7.38-7.33 (m, 3H), 7.22-7.06 (m, 10H), 7.01 (d, *J* = 7.2 Hz, 2H), 6.93 (t, *J* = 7.2 Hz, 1H), 6.86 (d, *J* = 7.2 Hz, 2H), 5.87 (s, 1H), 5.75 (s, 1H), 4.53 (d, *J* = 6.8 Hz, 1H), 4.14-4.08 (m, 2H), 3.49-3.40 (m, 2H), 2.92 (q, *J* = 8.4 Hz, 1H), 2.42 (s, 3H), 2.22-2.15 (m, 2H), 1.91-1.86 (m, 2H) ppm. **^{13}C NMR** (100 MHz, $CDCl_3$): δ 174.6, 158.9, 157.6, 143.8, 139.2, 138.9, 138.3, 137.4, 134.0, 129.5, 128.8, 128.3, 128.1, 127.9, 127.5, 127.4, 127.2, 126.9, 125.0, 123.5, 121.2, 120.4, 84.2, 77.6, 71.9, 69.4, 64.8, 56.3, 27.8, 24.9, 21.5 ppm.

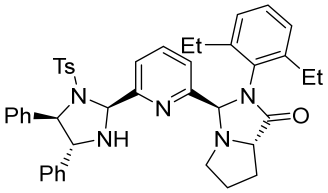

L10
$C_{43}H_{45}N_5O_3S$
M = 711.93 g/mol

(3*R*,7a*S*)-2-(2,6-diethylphenyl)-3-(6-((2*S*,4*R*,5*R*)-4,5-diphenyl-1-tosylimidazolidin-2-yl)pyridin-2-yl)hexahydro-1H-pyrrolo[1,2-c]imidazol-1-one: (**L10**) Prepared from (*S*)-*N*-(2,6-diethylphenyl)pyrrolidine-2-carboxamide (1.88 g, 10.0 mmol, 1.0 equiv) according to the **GP5**. Purification by flash chromatography on silica gel afforded **L10** as a white solid (1.10 g, 52% yield over 2 steps).

R*f* = 0.35 (petroleum ether/EtOAc = 5/1). **^{1}H NMR** (400 MHz, $CDCl_3$): δ 7.83 (t, *J* = 7.6 Hz, 1H), 7.68 (t, *J* = 7.2 Hz, 2H), 7.38 (d, *J* = 7.6 Hz, 2H), 7.25-7.15 (m, 8H), 7.06 (d, *J* = 7.6 Hz, 2H), 6.98-6.93 (m, 3H), 6.87 (d, *J* = 7.6 Hz, 1H), 6.76 (d, *J* = 7.2 Hz, 1H), 5.52 (s, 1H), 5.40 (s, 1H), 4.62 (d, *J* = 4.4 Hz, 1H), 4.29-4.26 (m, 1H), 4.10 (d, *J* = 4.4 Hz, 1H), 3.51-3.45 (m, 1H), 3.12-3.06 (m, 1H), 2.66-2.56 (m, 2H), 2.53-2.44 (m, 1H),

2.36 (s, 3H), 2.33-2.27 (m, 1H), 2.26-2.18 (m, 1H), 2.05-1.94 (m, 2H), 1.84-1.75 (m, 1H), 1.26-1.18 (m, 1H), 1.13 (t, *J* = 7.6 Hz, 3H), 0.72 (t, *J* = 7.6 Hz, 3H) ppm. **^{13}C NMR** (100 MHz, $CDCl_3$): δ 173.8, 158.0, 157.2, 143.4, 142.7, 141.2, 140.5, 140.1, 137.8, 134.2, 132.0, 129.3, 128.3, 128.2, 128.0, 127.6, 127.3, 127.1, 127.0, 126.5, 126.2, 126.1, 123.8, 121.3, 85.4, 78.2, 71.1, 69.4, 65.0, 56.9, 29.0, 25.5, 24.9, 22.7, 21.5, 14.6, 13.5 ppm.

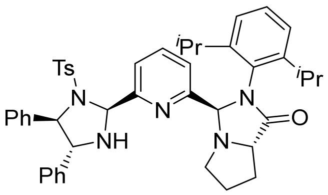

L11
$C_{45}H_{49}N_5O_3S$
M = 739.98 g/mol

(3*R*,7a*S*)-2-(2,6-diisopropylphenyl)-3-(6-((2*S*,4*R*,5*R*)-4,5-diphenyl-1-tosylimidazolidin-2-yl)pyridin-2-yl)hexahydro-1H-pyrrolo[1,2-c]imidazol-1-one: (**L11**) Prepared from (*S*)-*N*-(2,6-diisopropylphenyl)pyrrolidine-2-carboxamide (274.4 mg, 1.0 mmol, 1.0 equiv) according to the **GP5**. Purification by flash chromatography on silica gel afforded **L11** as a white solid (273.5 mg, 37% yield over 2 steps).

R_f = 0.4 (petroleum ether/EtOAc = 1/3). **^{1}H NMR** (400 MHz, $CDCl_3$): δ 7.86 (t, *J* = 7.6 Hz, 1H), 7.79 (d, *J* = 7.6 Hz, 1H), 7.71 (d, *J* = 7.6 Hz, 1H), 7.52 (d, *J* = 8.0 Hz, 2H), 7.20-7.09 (m, 10H), 7.05 (t, *J* = 7.6 Hz, 1H), 6.94-6.86 (m, 4H), 5.62 (s, 1H), 5.31 (s, 1H), 4.47 (d, *J* = 6.0 Hz, 1H), 4.35-4.32 (m, 1H), 4.15 (d, *J* = 6.4 Hz, 1H), 3.44-3.38 (m, 1H), 3.07-3.01 (m, 1H), 2.93-2.88 (m, 1H), 2.83 (s, br, 1H), 2.41 (s, 3H), 2.33-2.26 (m, 1H), 2.24-2.15 (m, 2H), 2.04-1.90 (m, 2H), 1.11 (d, *J* = 6.8 Hz, 3H), 1.05 (d, *J* = 6.8 Hz, 3H), 0.97 (d, *J* = 6.8 Hz, 3H), 0.21 (d, *J* = 6.8 Hz, 3H) ppm. **^{13}C NMR** (100 MHz, $CDCl_3$): δ 174.1, 158.2, 157.8, 147.9, 146.0, 143.7, 139.7, 139.6, 138.0, 133.9, 129.9, 129.5, 128.9, 128.2, 128.1, 127.8, 127.4, 127.3, 127.2, 126.7, 123.9, 123.7, 123.5, 121.7, 87.1, 77.8, 71.7, 69.5, 65.2, 56.9, 29.3, 29.0, 28.8, 25.4, 25.0, 24.8, 23.2, 22.6, 21.5 ppm.

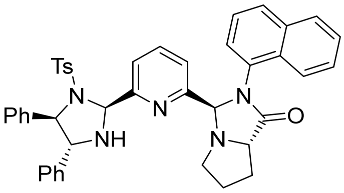

L12
$C_{43}H_{39}N_5O_3S$
M = 705.88 g/mol

(3*R*,7a*S*)-3-(6-((2*S*,4*R*,5*R*)-4,5-diphenyl-1-tosylimidazolidin-2-yl)pyridin-2-yl)-2-(naphthalen-1-yl)hexahydro-1H-pyrrolo[1,2-c]imidazol-1-one: (**L12**) Prepared from (*S*)-*N*-(naphthalen-1-yl)pyrrolidine-2-carboxamide (576.7 mg, 2.4 mmol, 1.0 equiv) according to the **GP5**. Purification by flash chromatography on silica gel afforded **L12** as a white solid (241.0 mg, 14% yield over 2 steps).

R_f = 0.35 (petroleum ether/EtOAc = 5/1). **^{1}H NMR** (400 MHz, $CDCl_3$): δ 7.86 (d, *J* = 8.0 Hz, 1H), 7.77 (t, *J* = 8.0 Hz, 1H), 7.72 (d, *J* = 7.6 Hz, 1H), 7.64-7.56 (m, 4H), 7.38-7.32 (m, 3H), 7.21-7.13 (m, 8H), 7.06 (d, *J* = 7.6 Hz, 3H), 6.86 (d, *J* = 7.2 Hz, 3H), 5.69 (s, 1H), 5.62 (s, 1H), 4.53 (d, *J* = 6.4 Hz, 1H), 4.45-4.42 (m, 1H), 4.05 (d, *J* = 6.4 Hz, 1H), 3.57-3.52 (m, 1H), 3.25-3.19 (m, 1H), 3.06 (s, br, 1H), 2.42 (s, 3H), 2.34-2.27 (m, 2H), 2.10-2.01 (m, 2H) ppm. **^{13}C NMR** (100 MHz, $CDCl_3$): δ 175.1, 158.7, 156.0, 143.8, 139.6, 139.0, 138.0, 134.3, 133.9, 132.3, 130.0, 129.5, 128.6, 128.4, 128.3, 128.2, 127.9, 127.5, 127.4, 127.0, 126.9, 126.7, 126.2, 125.3, 123.7, 121.9, 121.2, 86.6, 77.7, 71.7, 69.5, 65.1, 56.9, 28.4, 25.4, 21.5 ppm.

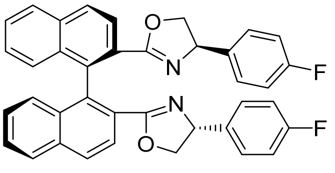

L21
$C_{38}H_{26}F_2N_2O_2$
M = 580.63 g/mol

R_f = 0.3 (petroleum ether/EtOAc = 2/1). **^{1}H NMR** (600 MHz, $CDCl_3$): δ 8.14 (d, *J* = 8.6 Hz, 2H), 7.98-7.95 (m, 4H), 7.55-7.52 (m, 2H), 7.31-7.25 (m, 4H), 6.72 (t, *J* = 8.6 Hz, 4H), 6.53 (dd, *J* = 8.6, 5.6 Hz, 4H), 5.04 (dd, *J* = 10.0, 8.2 Hz, 2H), 4.24 (dd, *J* = 10.2, 8.4 Hz, 2H), 3.67 (t, *J* = 8.4 Hz, 2H) ppm. **^{13}C NMR** (150 MHz, $CDCl_3$): δ 165.0, 161.8

(d, J = 243 Hz), 138.2, 138.1, 134.5, 132.9, 127.93 (d, J = 6 Hz), 127.86, 127.8, 127.1, 127.0, 126.7, 126.1, 125.6, 115.0 (d, J = 21 Hz), 74.3, 69.3 ppm. **^{19}F NMR** (565 MHz, $CDCl_3$) δ -115.87 ppm. **HRMS** (ESI) *m/z*: $[M+H]^+$ calcd. for $C_{38}H_{27}F_2N_2O_2$: 581.2035; found: 581.2032.

3. X-Ray Crystallography

Supplementary Table 3. Crystal data and structure refinement of compound 3ao

Crystallographic files (CDCC: 2345666). There are no A-alerts and B-alerts, see CIF/checkCIF). The crystals are grown by slow solvent (CH_2Cl_2/Et_2O/*n*-Hexane) evaporation at room temperature. CCDC number of (*S*)-(3-benzyl-4-(4-bromophenyl)-1-phenyl-2-oxa-3-azabicyclo[3.1.1]heptan-5-yl)(1-methyl-1H-imidazol-2-yl)methanone **3ao** (>99% *ee*) is 2345666. Flack parameter of **3ao:** 0.116(7)

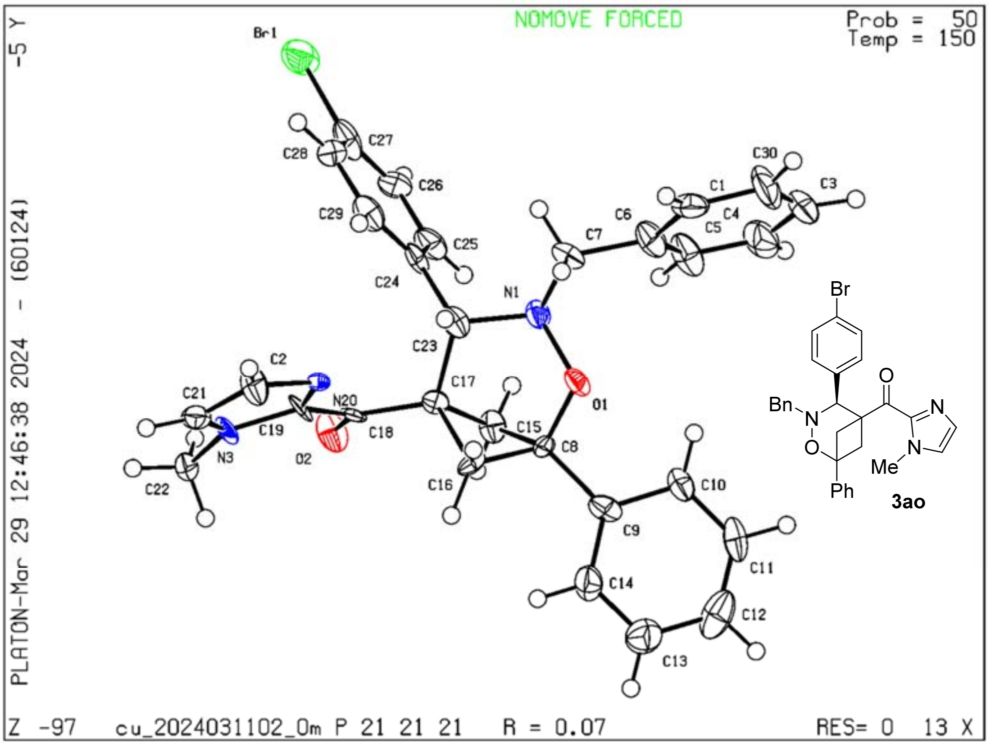

Datablock: cu_2024031102_0m

Bond precision: C-C = 0.0133 A Wavelength=1.54178

Cell:	a=10.0277(2)	b=10.9571(3)	c=26.1879(7)
	alpha=90	beta=90	gamma=90
Temperature:	150 K		

	Calculated	Reported
Volume	2877.38(12)	2877.38(12)
Space group	P 21 21 21	P 21 21 21
Hall group	P 2ac 2ab	P 2ac 2ab
Moiety formula	C29 H26 Br N3 O2 [+ solvent]	C29 H26 Br N3 O2
Sum formula	C29 H26 Br N3 O2 [+ solvent]	C29 H26 Br N3 O2
Mr	528.43	528.44
Dx,g cm-3	1.220	1.220
Z	4	4
Mu (mm-1)	2.162	2.162
F000	1088.0	1088.0
F000’	1087.99	
h,k,lmax	12,13,31	12,13,31
Nref	5262[2994]	5247
Tmin,Tmax	0.802,0.933	0.539,0.753
Tmin’	0.467	

Correction method= # Reported T Limits: Tmin=0.539 Tmax=0.753
AbsCorr = MULTI-SCAN

Data completeness= 1.75/1.00 Theta(max)= 68.329

4. Supplementary NMR and HPLC Spectra

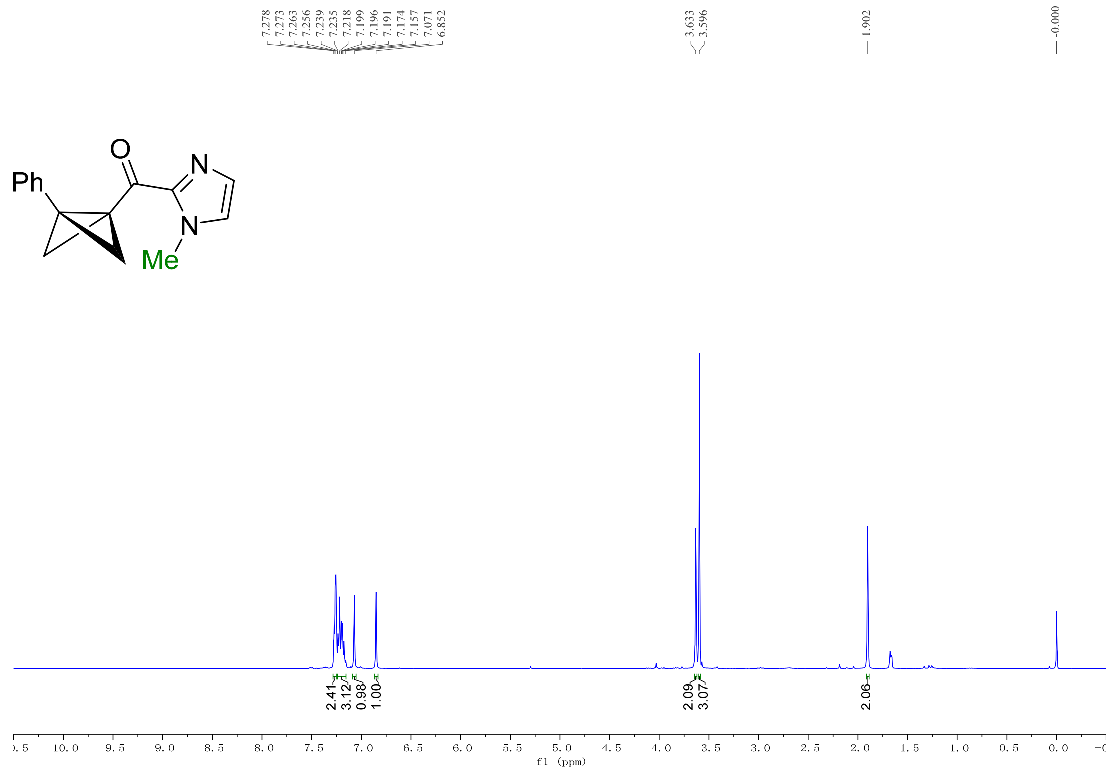

Supplementary Figure 8. ^{1}H NMR (400 MHz, $CDCl_3$) spectrum of compound **1a**

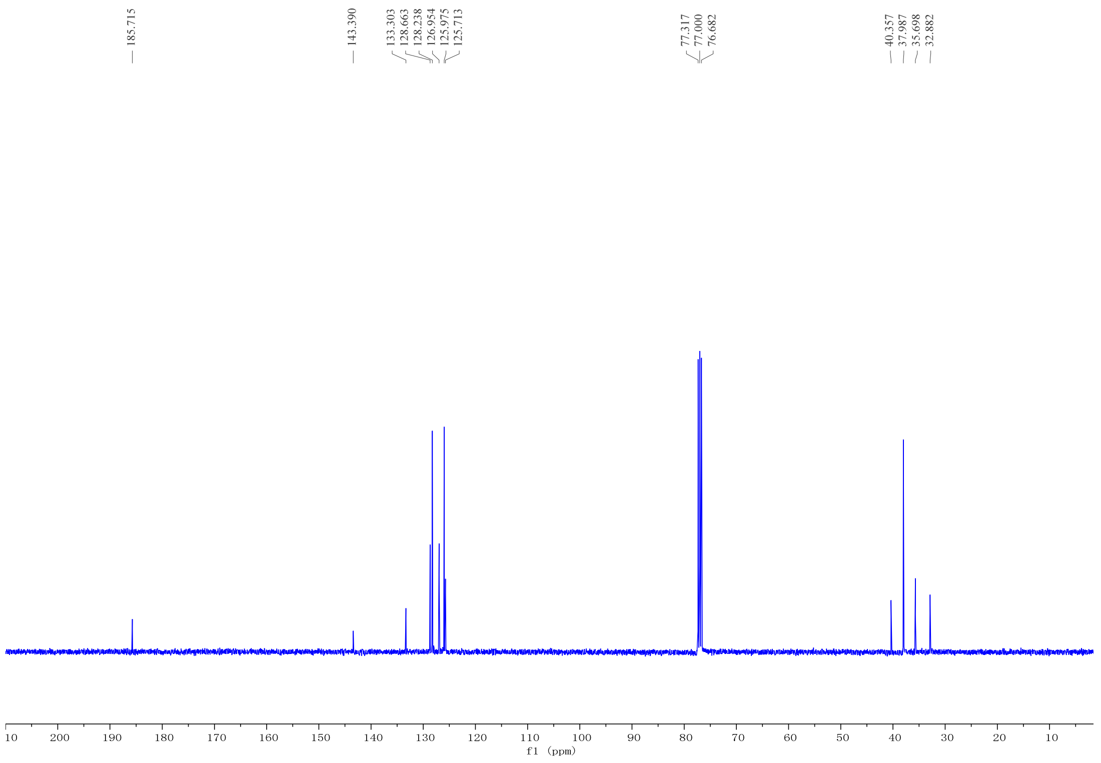

Supplementary Figure 9. ^{13}C NMR (100 MHz, $CDCl_3$) spectrum of compound **1a**

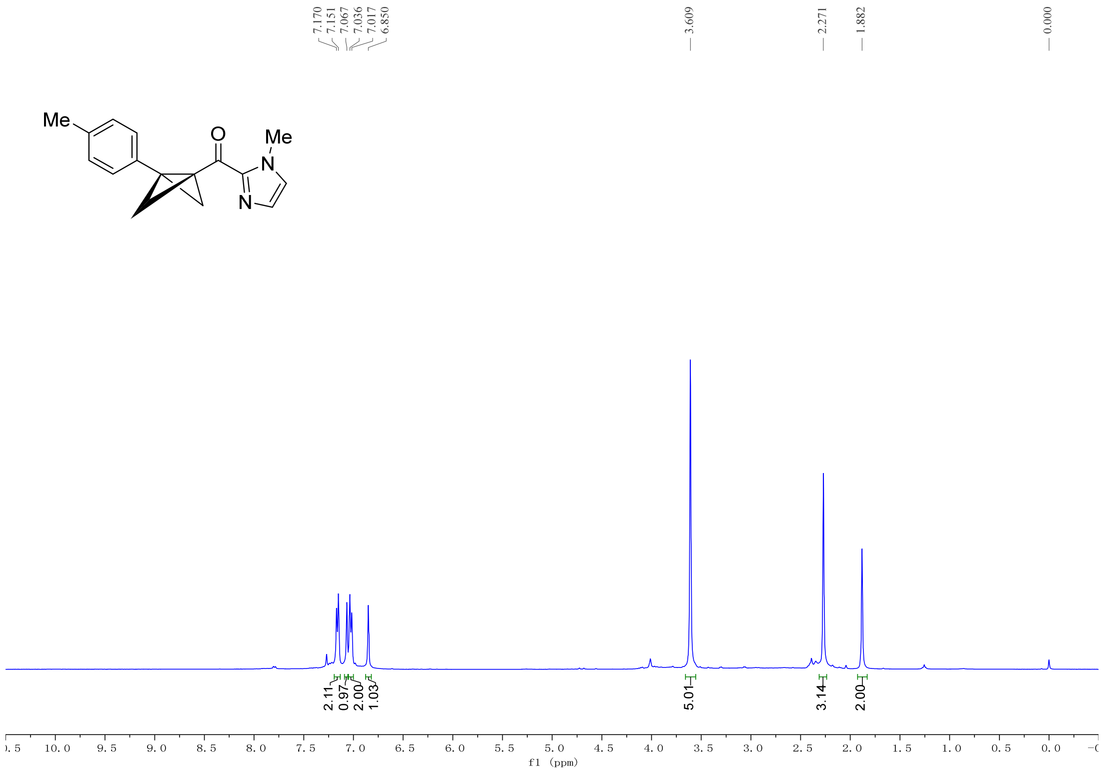

Supplementary Figure 10. ^{1}H NMR (400 MHz, $CDCl_3$) spectrum of compound **1b**

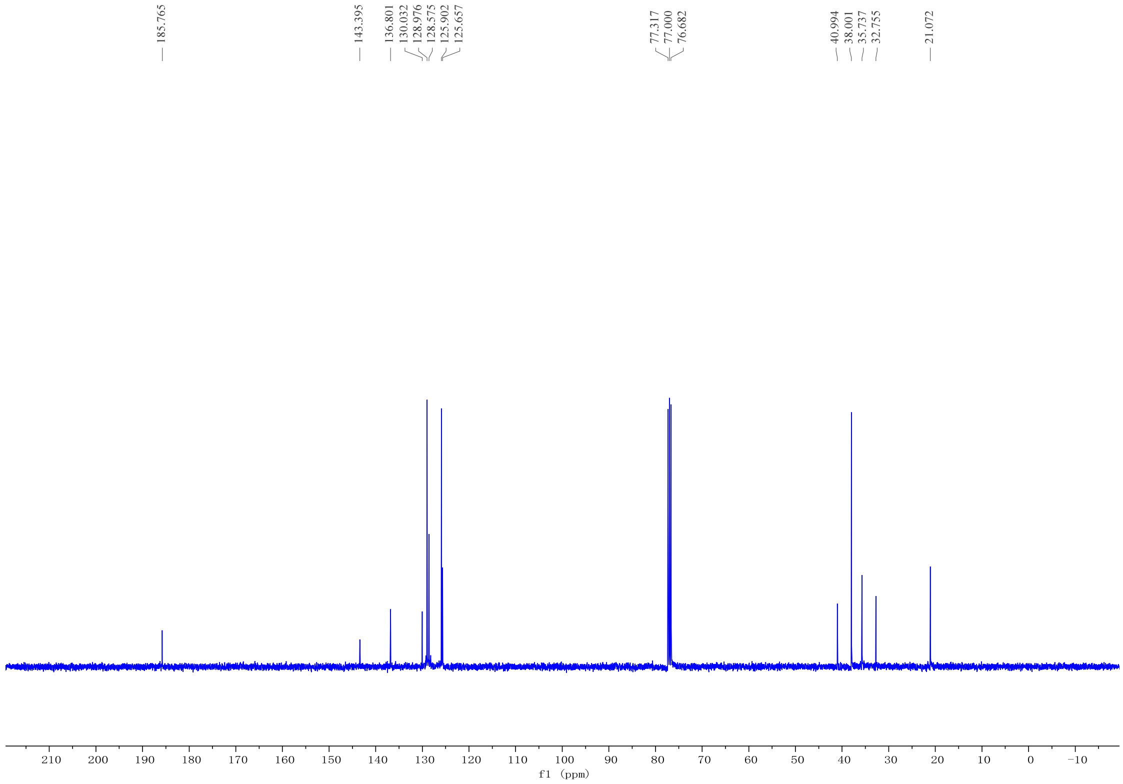

Supplementary Figure 11. ^{13}C NMR (100 MHz, $CDCl_3$) spectrum of compound **1b**

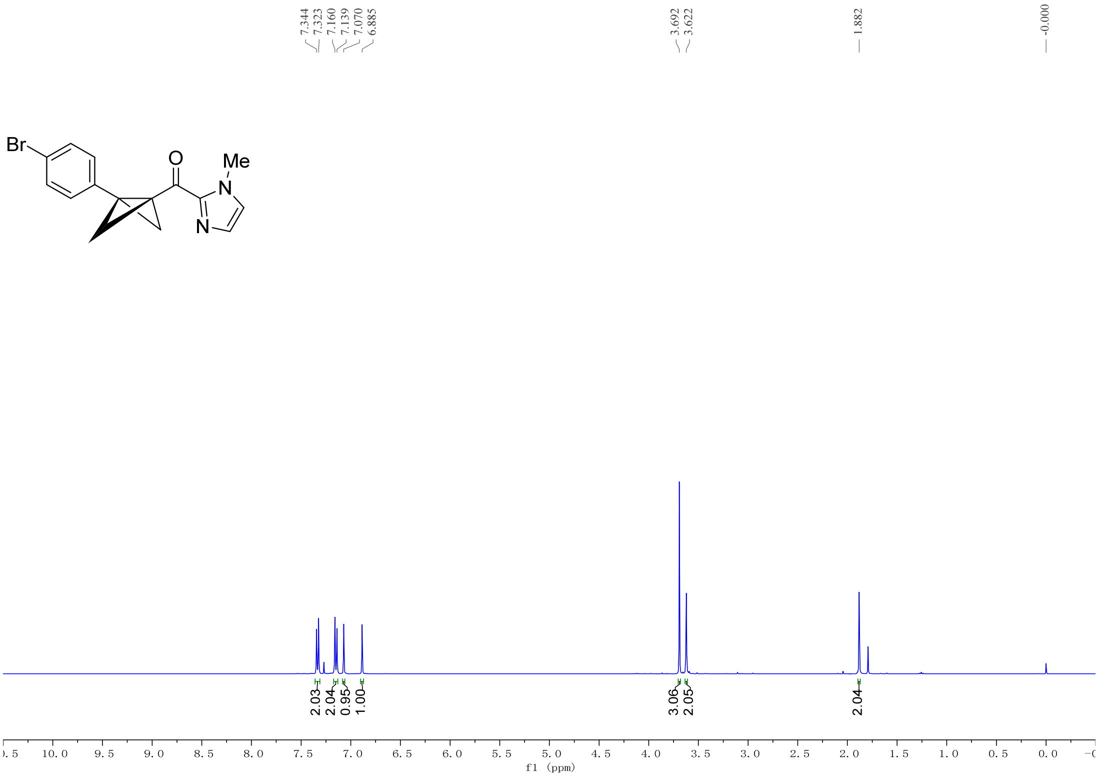

Supplementary Figure 12. ^{1}H NMR (400 MHz, $CDCl_3$) spectrum of compound **1c**

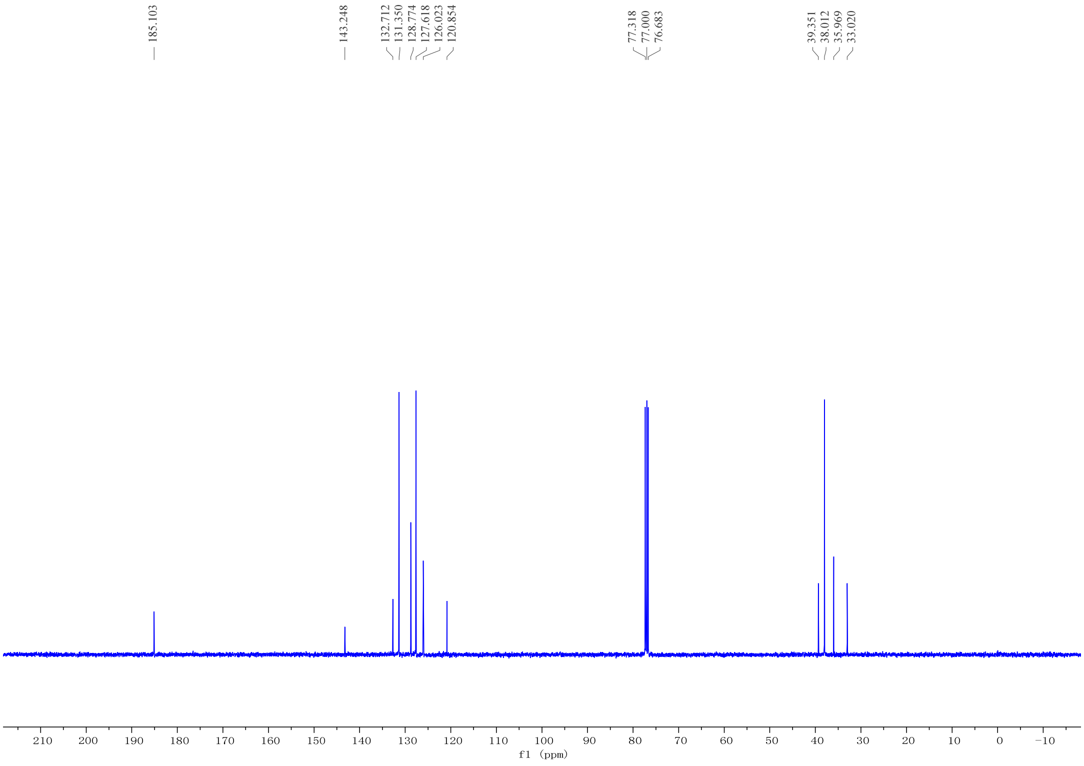

Supplementary Figure 13. ^{13}C NMR (100 MHz, $CDCl_3$) spectrum of compound **1c**

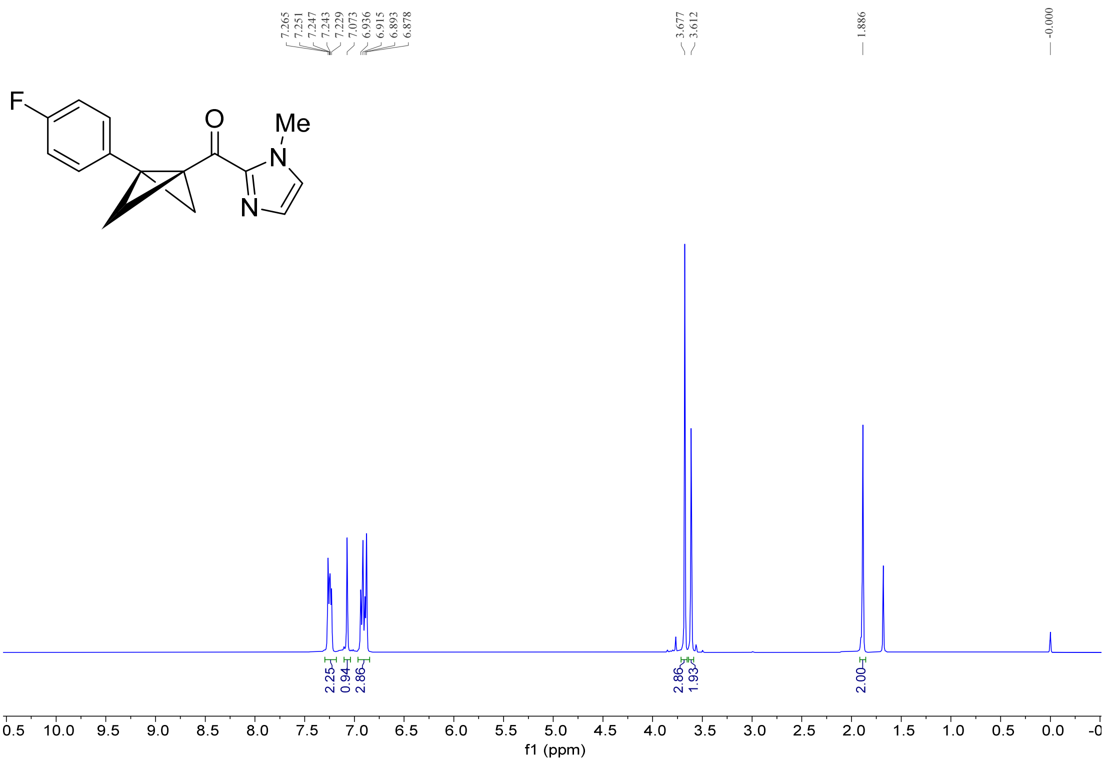

Supplementary Figure 14. ^{1}H NMR (400 MHz, $CDCl_3$) spectrum of compound **1d**

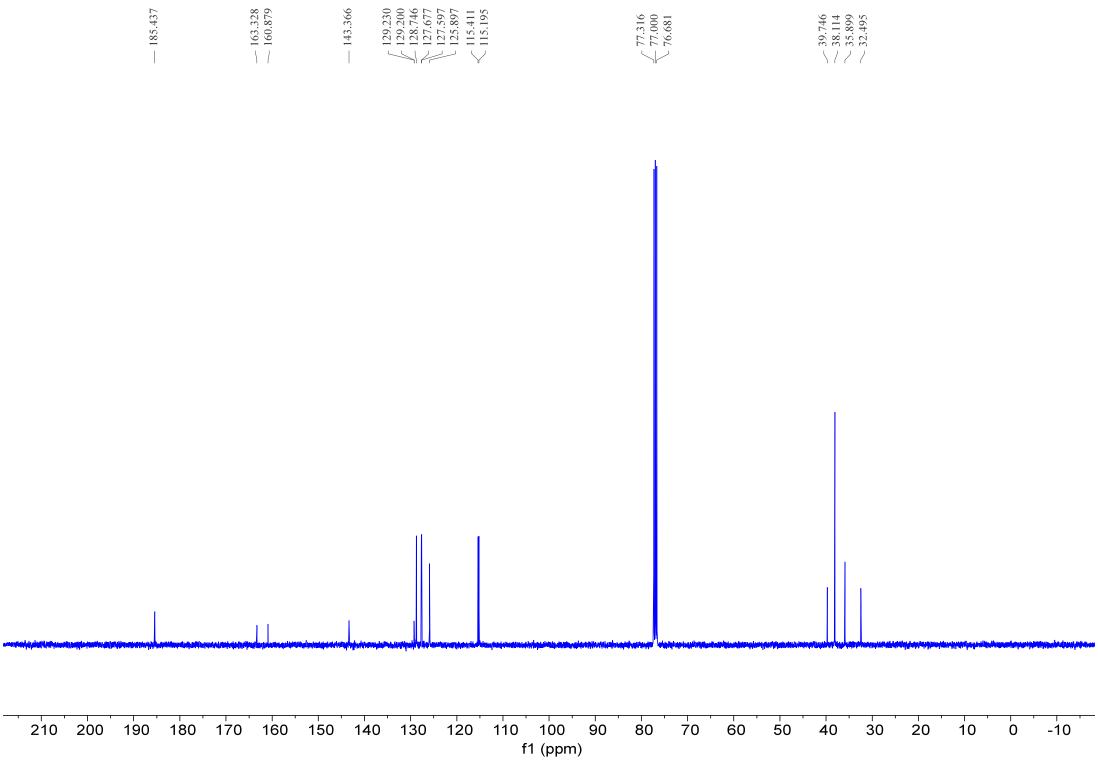

Supplementary Figure 15. ^{13}C NMR (100 MHz, $CDCl_3$) spectrum of compound **1d**

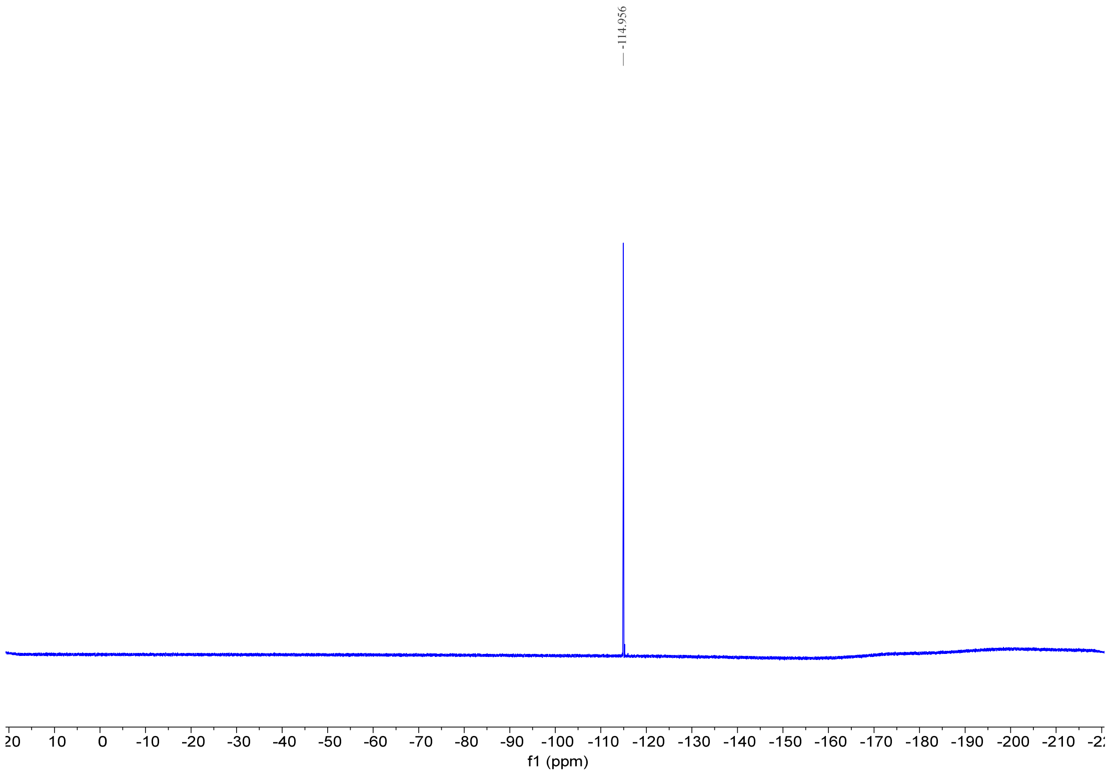

Supplementary Figure 16. ^{19}F NMR (376 MHz, $CDCl_3$) spectrum of compound **1d**

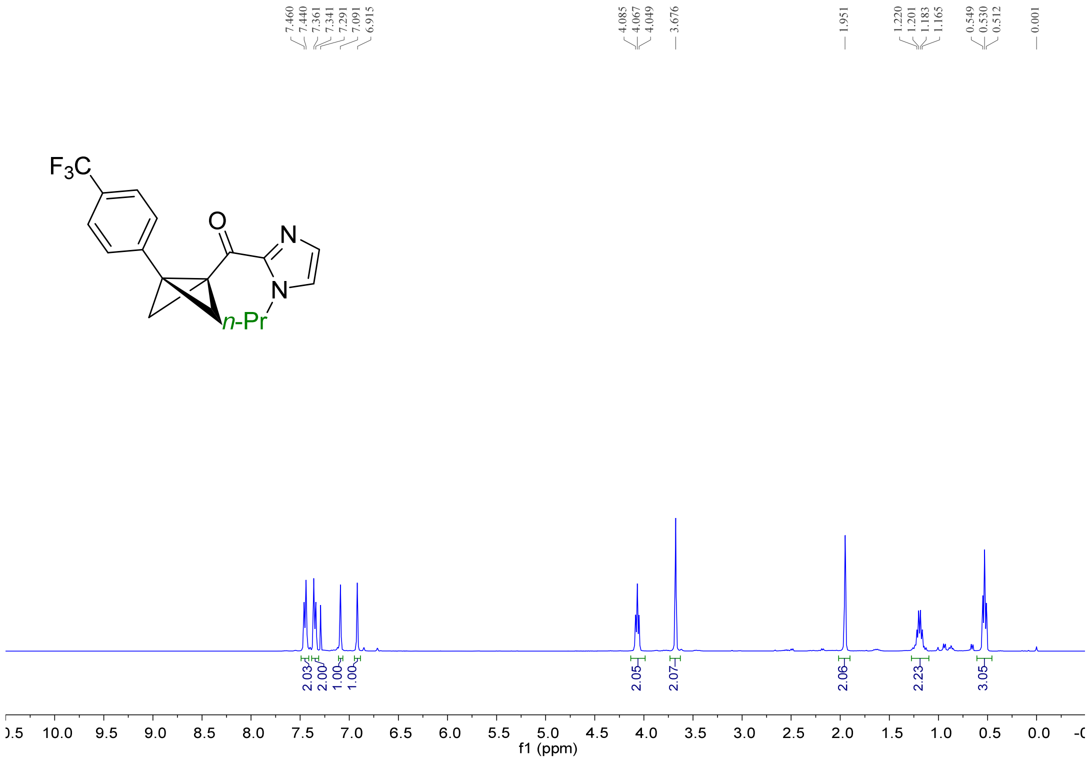

Supplementary Figure 17. ^1H NMR (400 MHz, $CDCl_3$) spectrum of compound **1e**

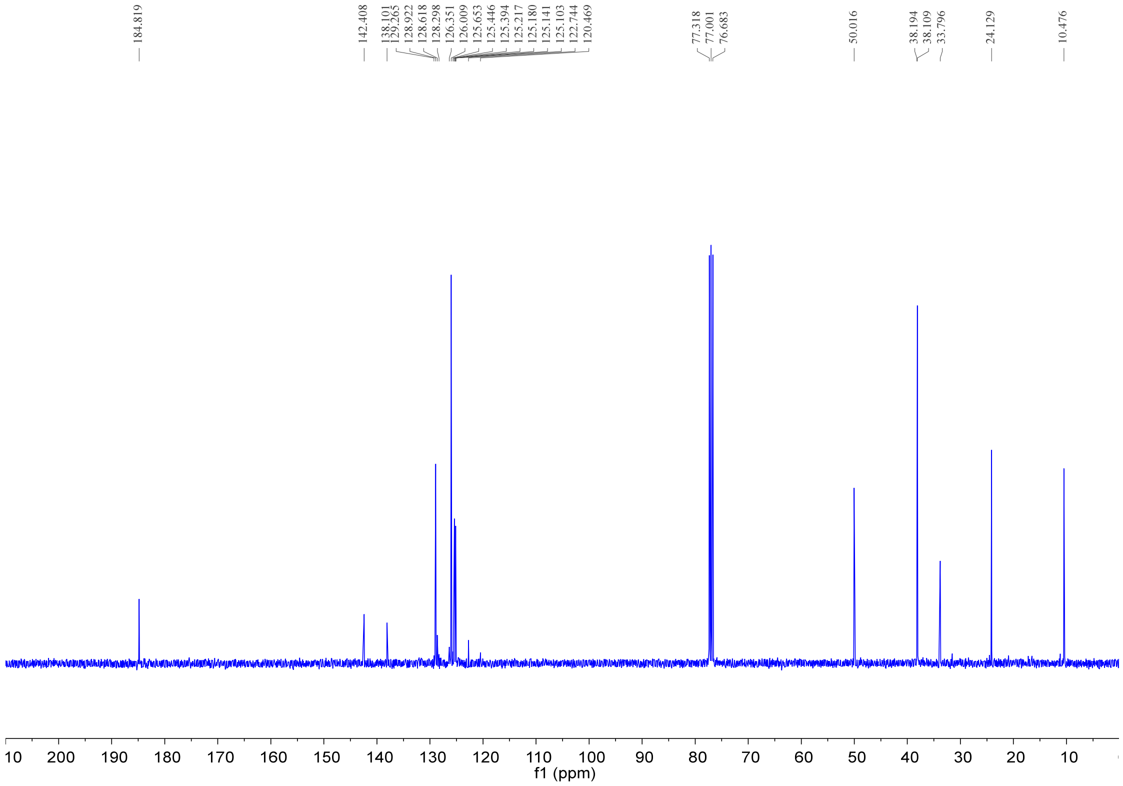

Supplementary Figure 18. ^{13}C NMR (100 MHz, $CDCl_3$) spectrum of compound **1e**

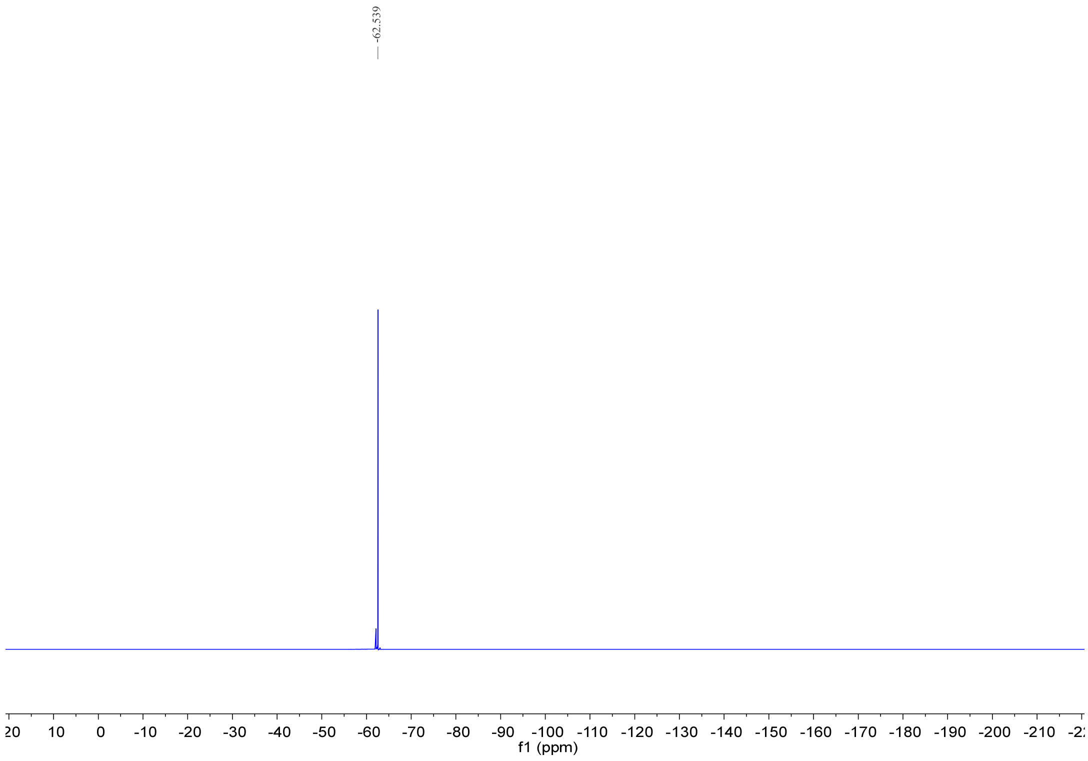

Supplementary Figure 19. ^{19}F NMR (376 MHz, $CDCl_3$) spectrum of compound **1e**

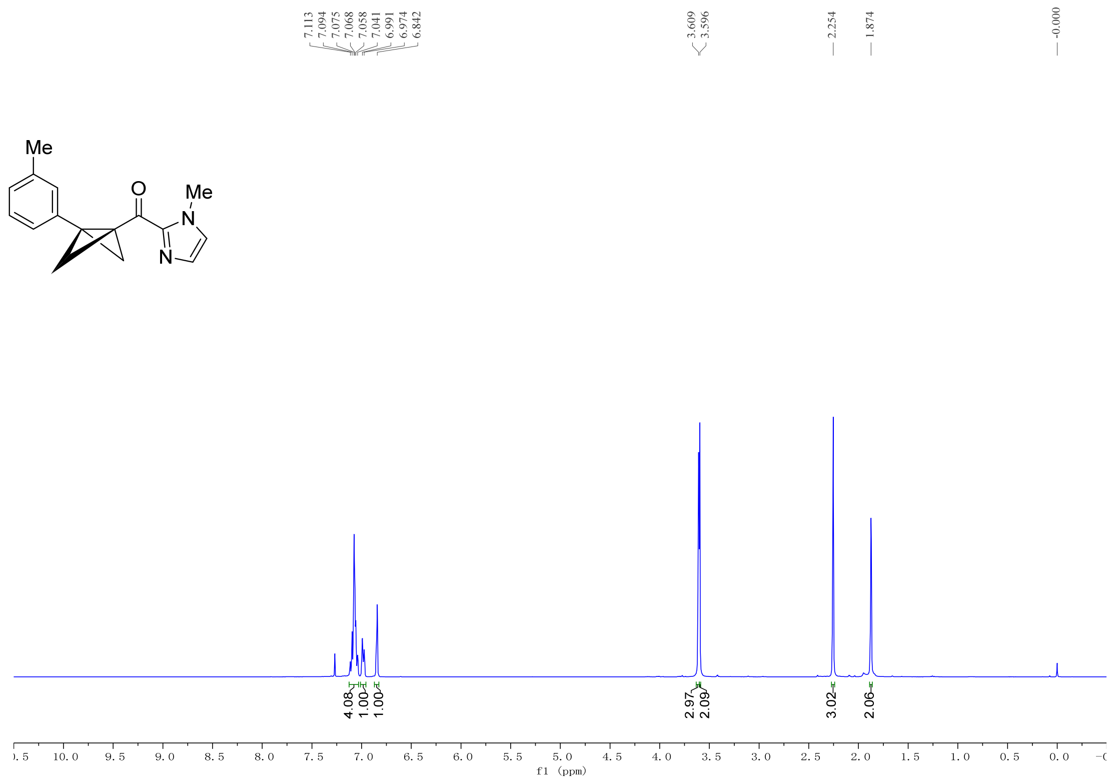

Supplementary Figure 20. ^{1}H NMR (400 MHz, $CDCl_3$) spectrum of compound **1f**

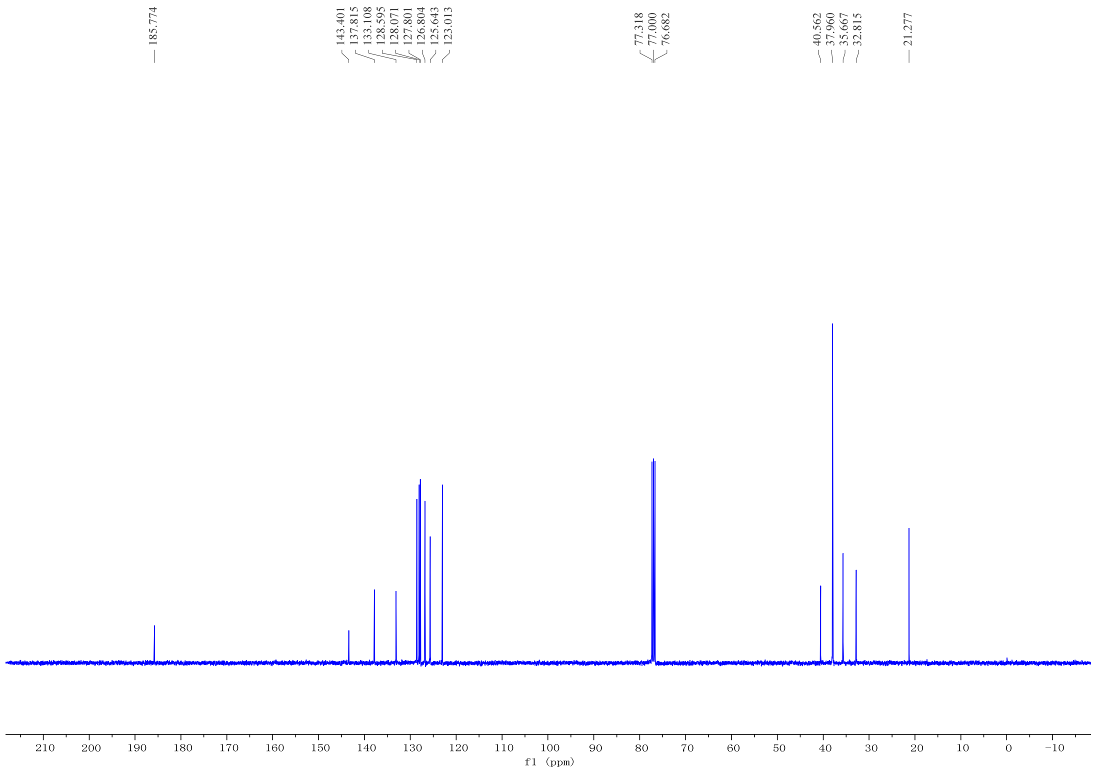

Supplementary Figure 21. ^{13}C NMR (100 MHz, $CDCl_3$) spectrum of compound **1f**

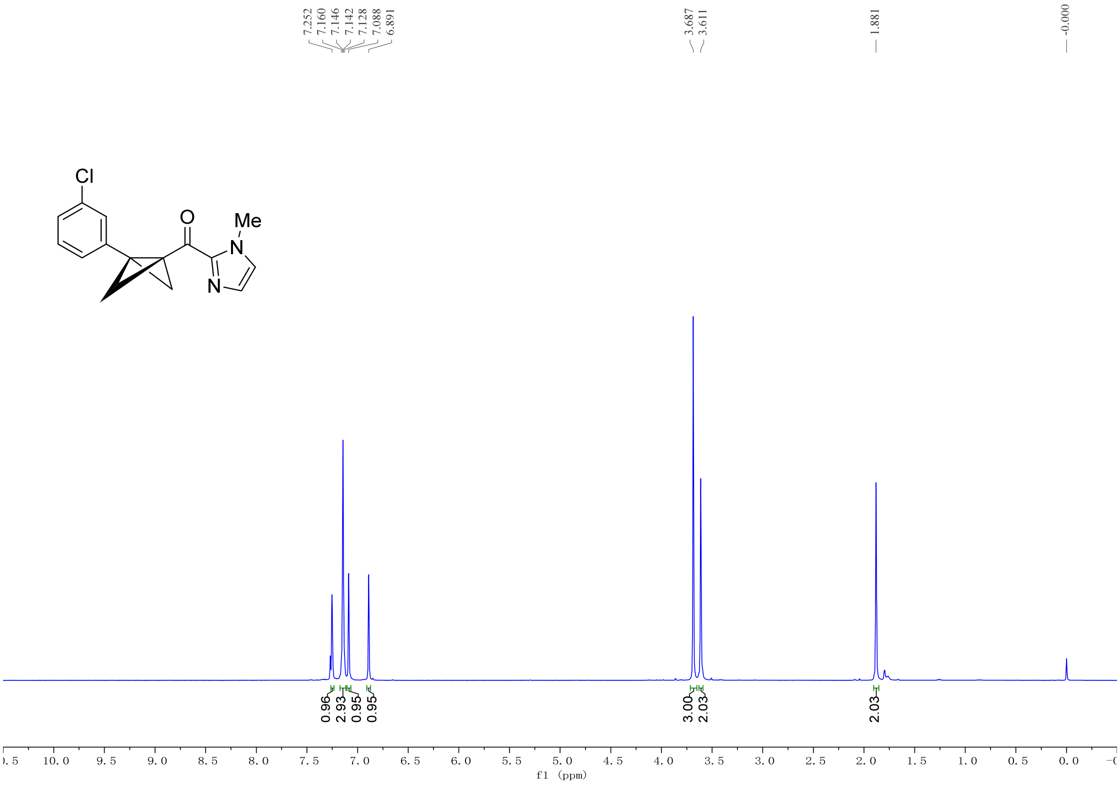

Supplementary Figure 22. ^{1}H NMR (400 MHz, $CDCl_3$) spectrum of compound **1g**

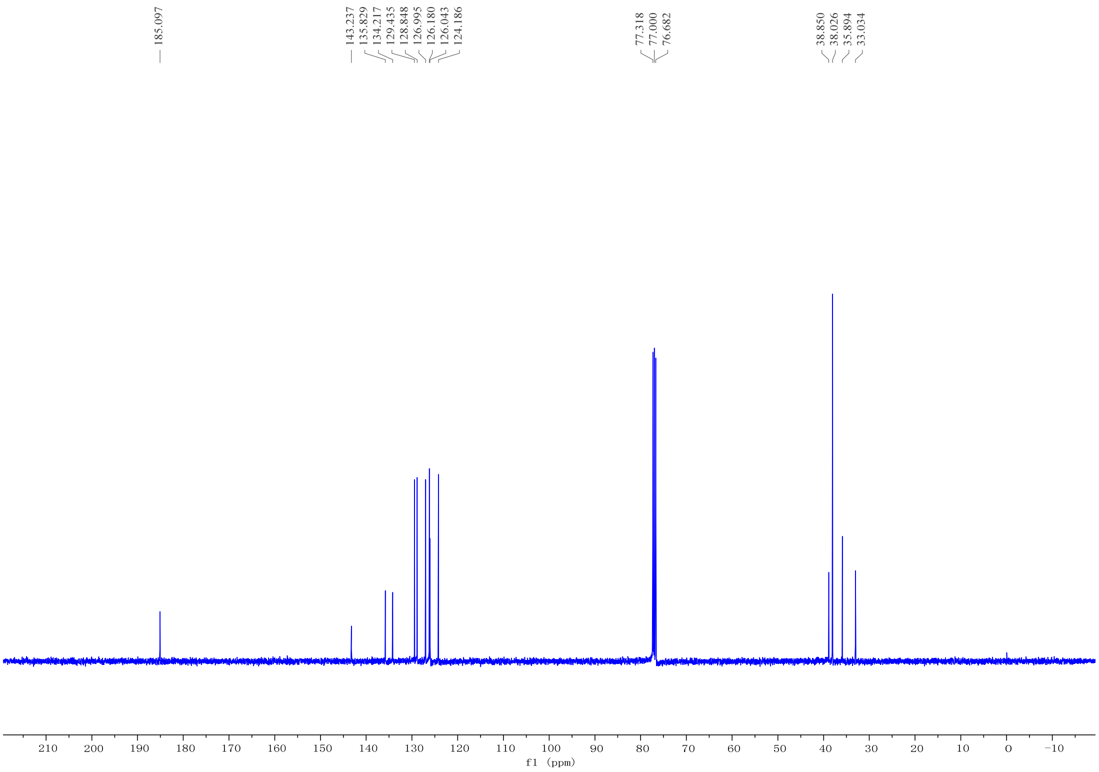

Supplementary Figure 23. ^{13}C NMR (100 MHz, $CDCl_3$) spectrum of compound **1g**

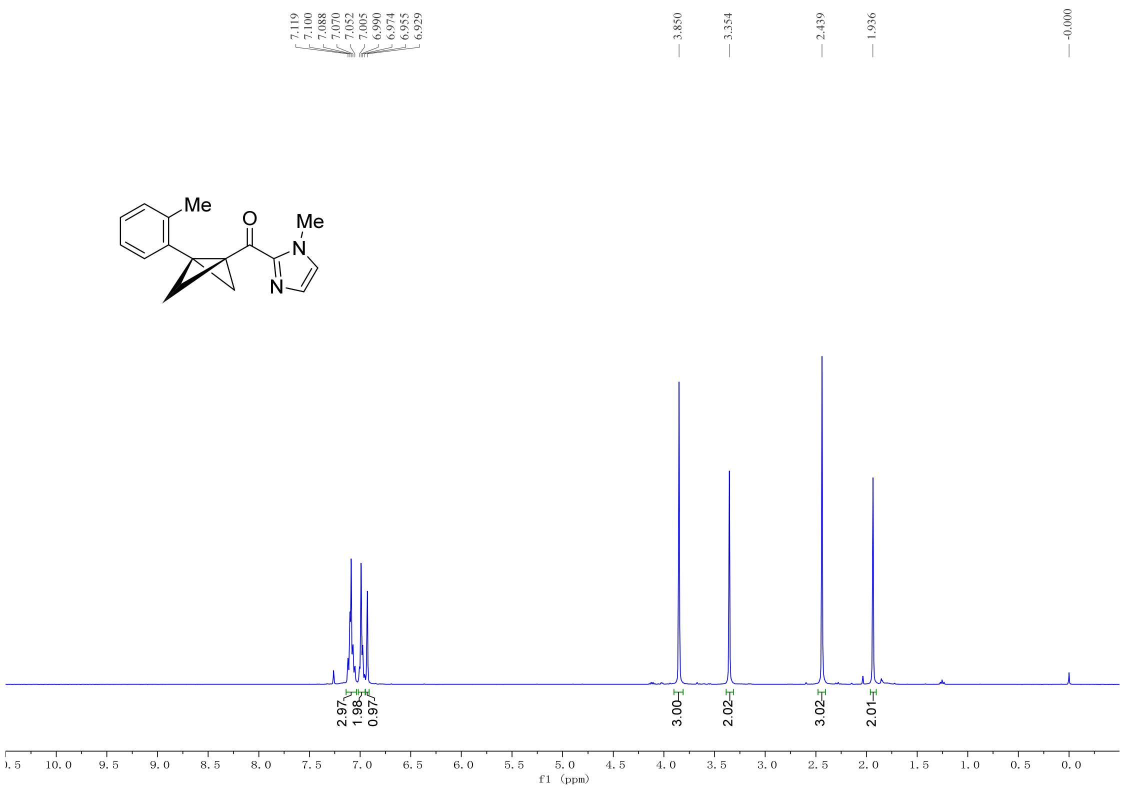

Supplementary Figure 24. ^{1}H NMR (400 MHz, $CDCl_3$) spectrum of compound **1h**

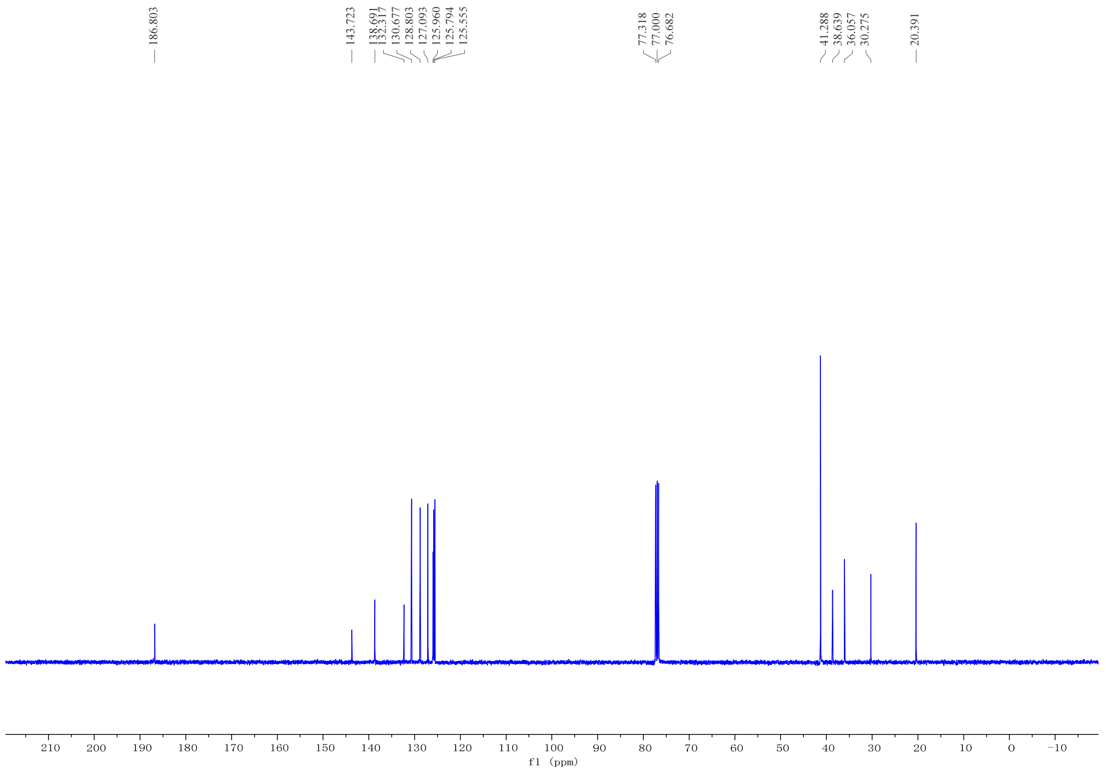

Supplementary Figure 25. ^{13}C NMR (100 MHz, $CDCl_3$) spectrum of compound **1h**

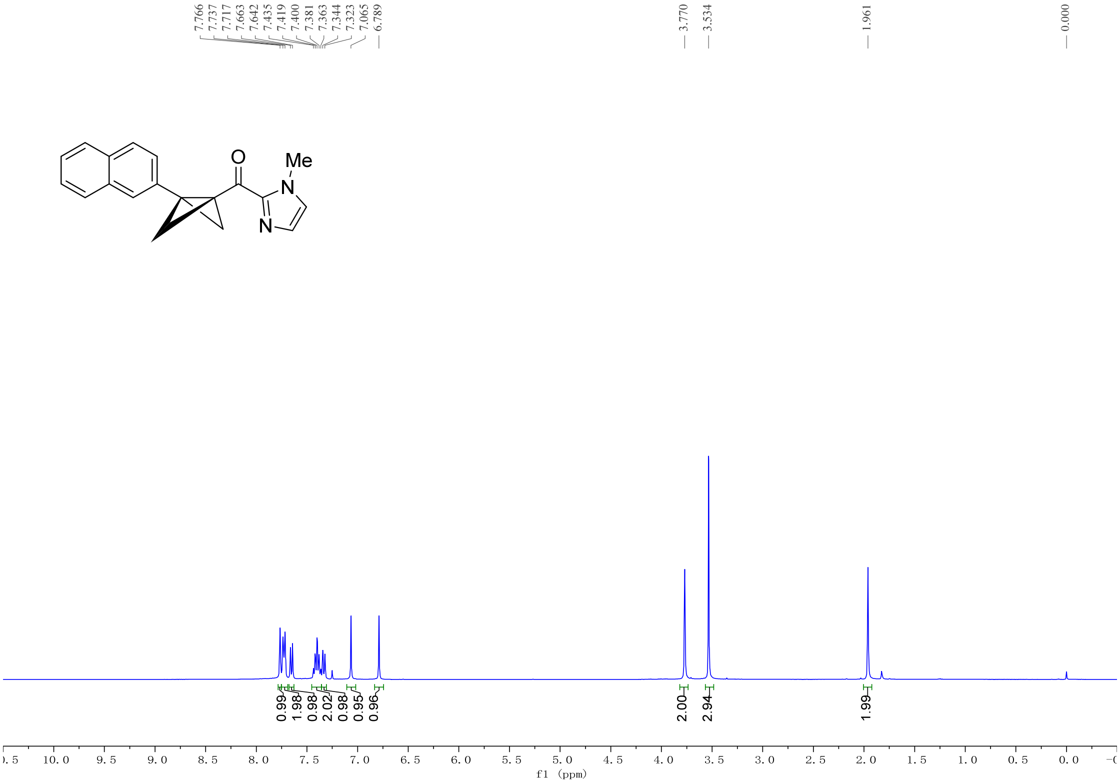

Supplementary Figure 26. ^1H NMR (400 MHz, $CDCl_3$) spectrum of compound **1i**

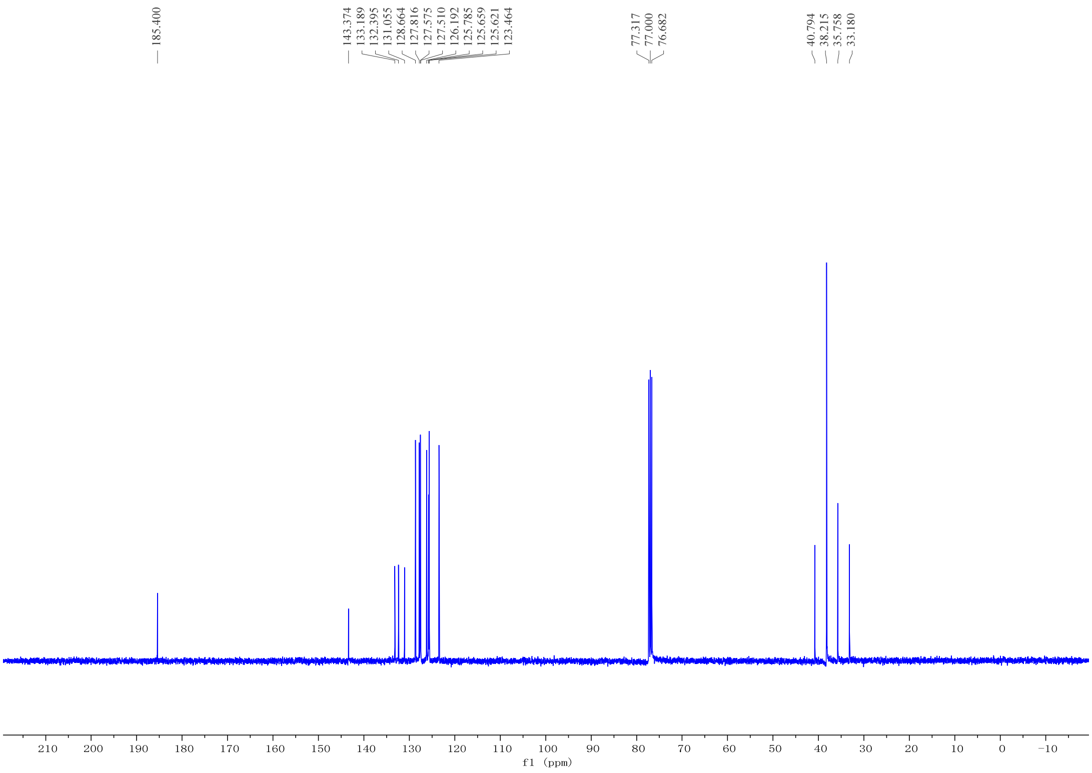

Supplementary Figure 27. ^{13}C NMR (100 MHz, $CDCl_3$) spectrum of compound **1i**

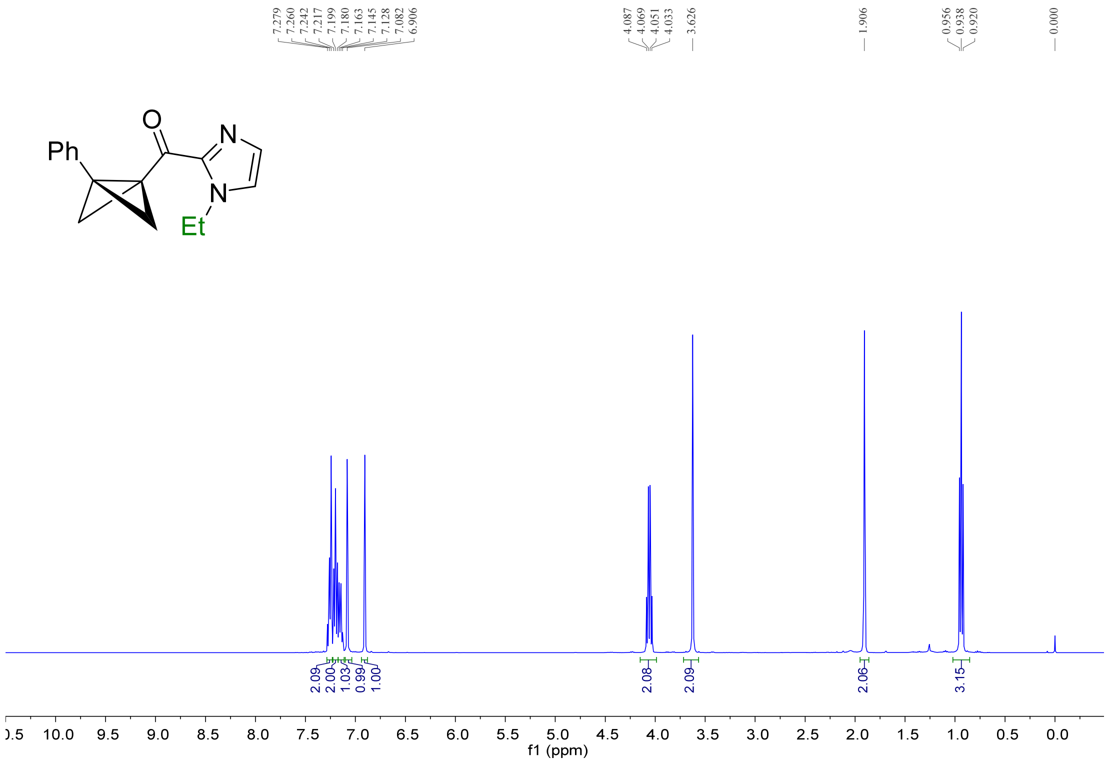

Supplementary Figure 28. ^{1}H NMR (400 MHz, $CDCl_3$) spectrum of compound **1j**

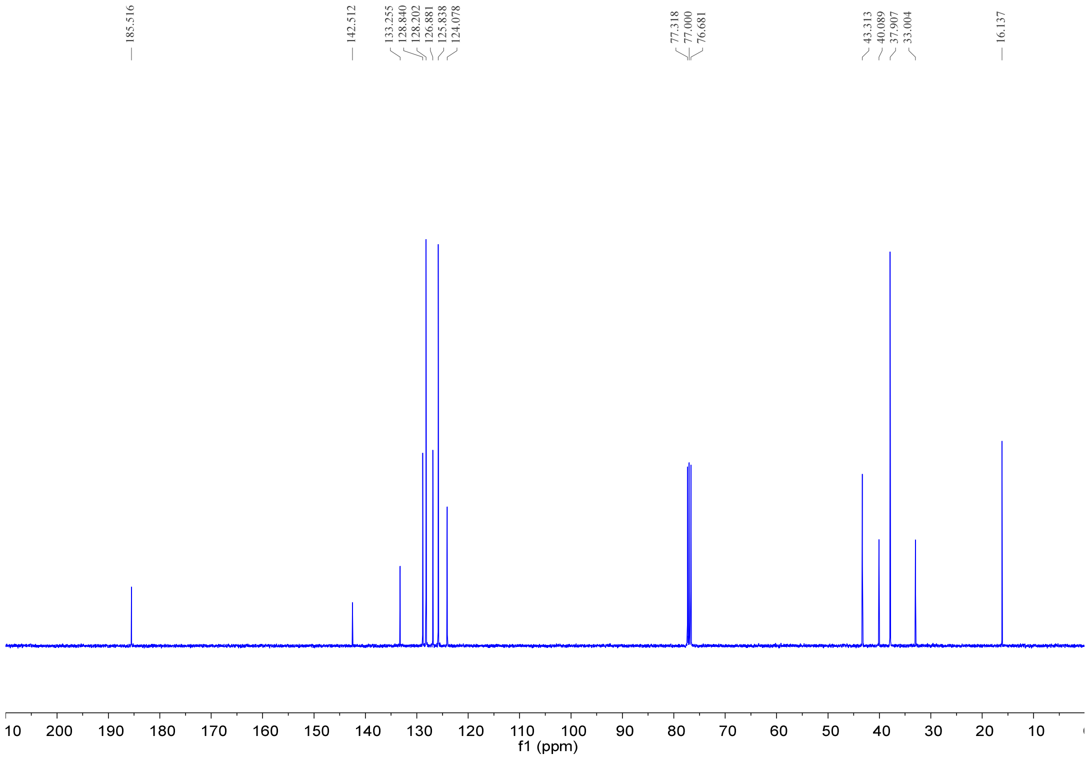

Supplementary Figure 29. ^{13}C NMR (100 MHz, $CDCl_3$) spectrum of compound **1j**

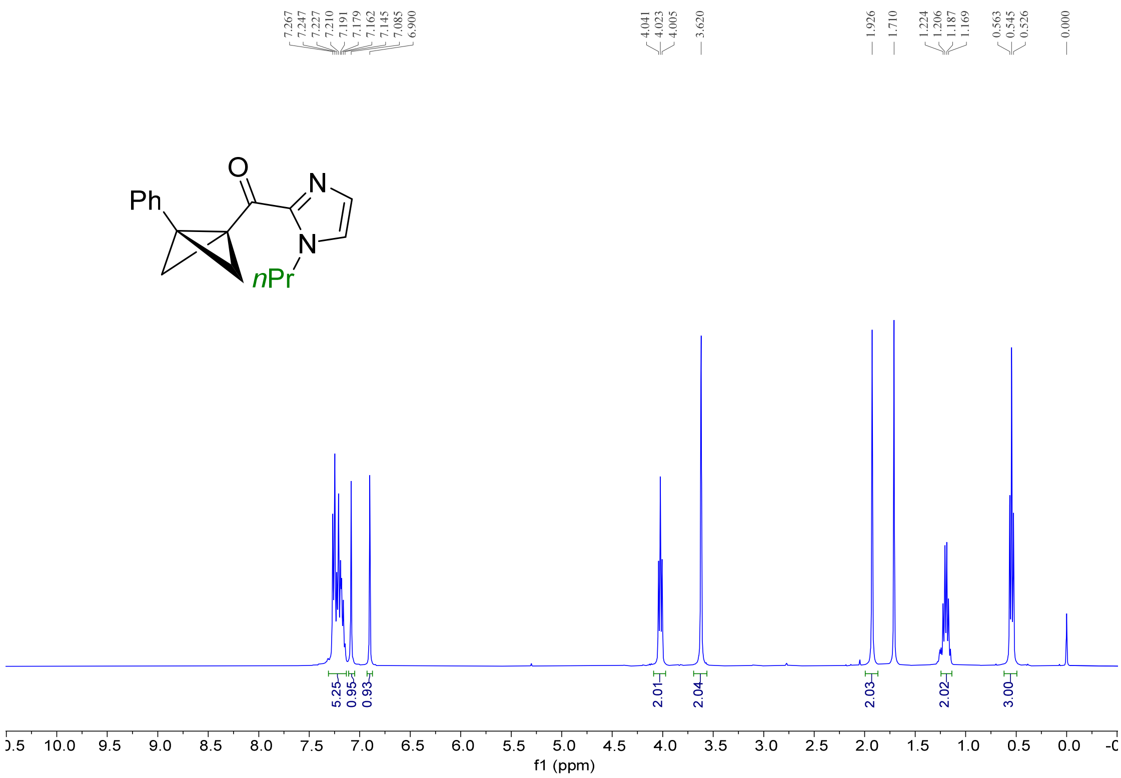

Supplementary Figure 30. ^{1}H NMR (400 MHz, $CDCl_3$) spectrum of compound **1k**

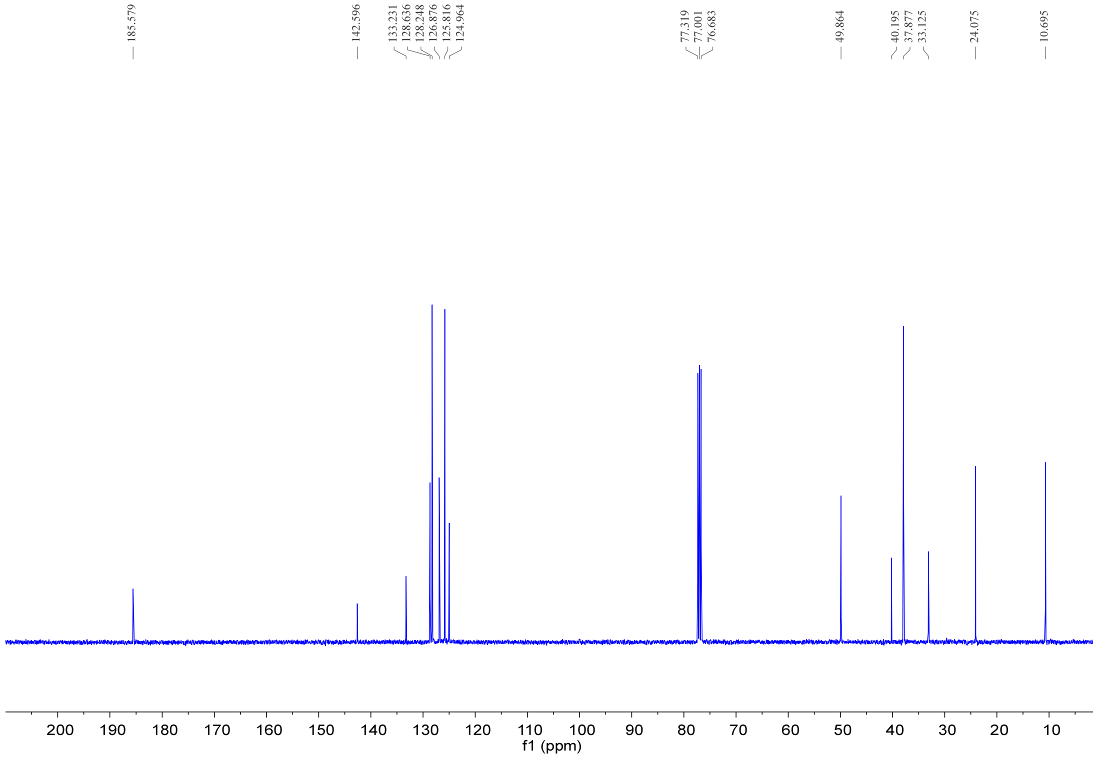

Supplementary Figure 31. ^{13}C NMR (100 MHz, $CDCl_3$) spectrum of compound **1k**

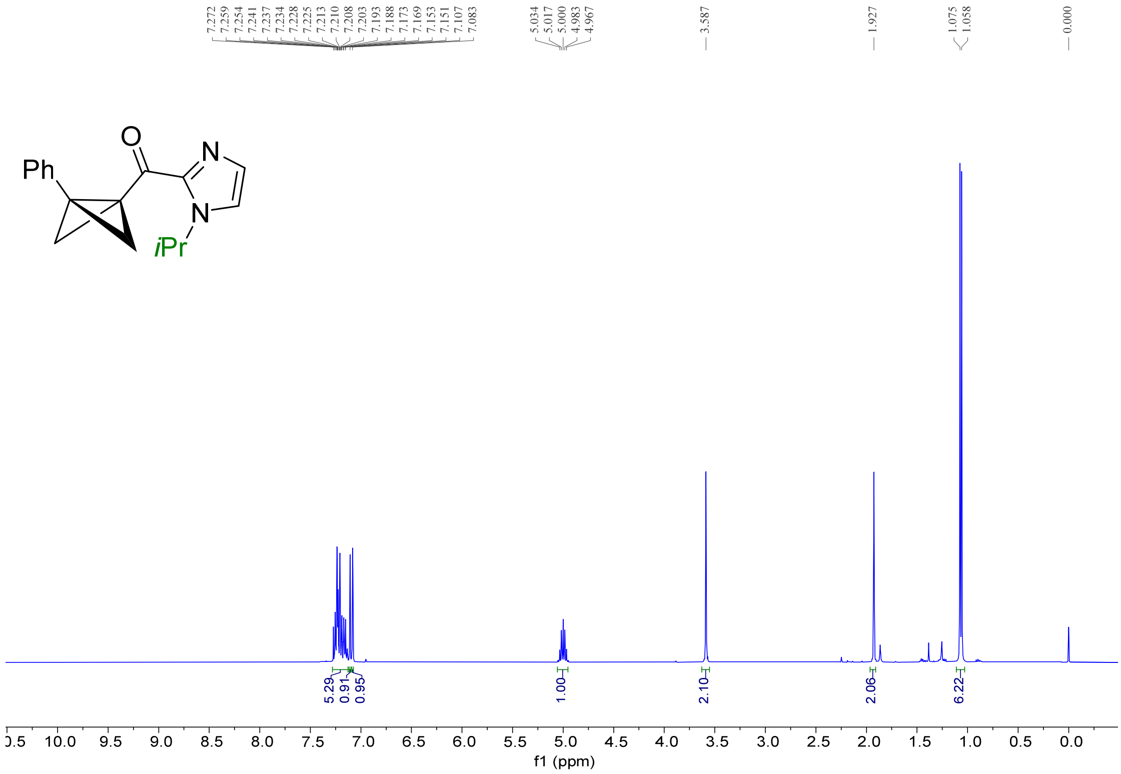

Supplementary Figure 32. ^{1}H NMR (400 MHz, $CDCl_3$) spectrum of compound **1l**

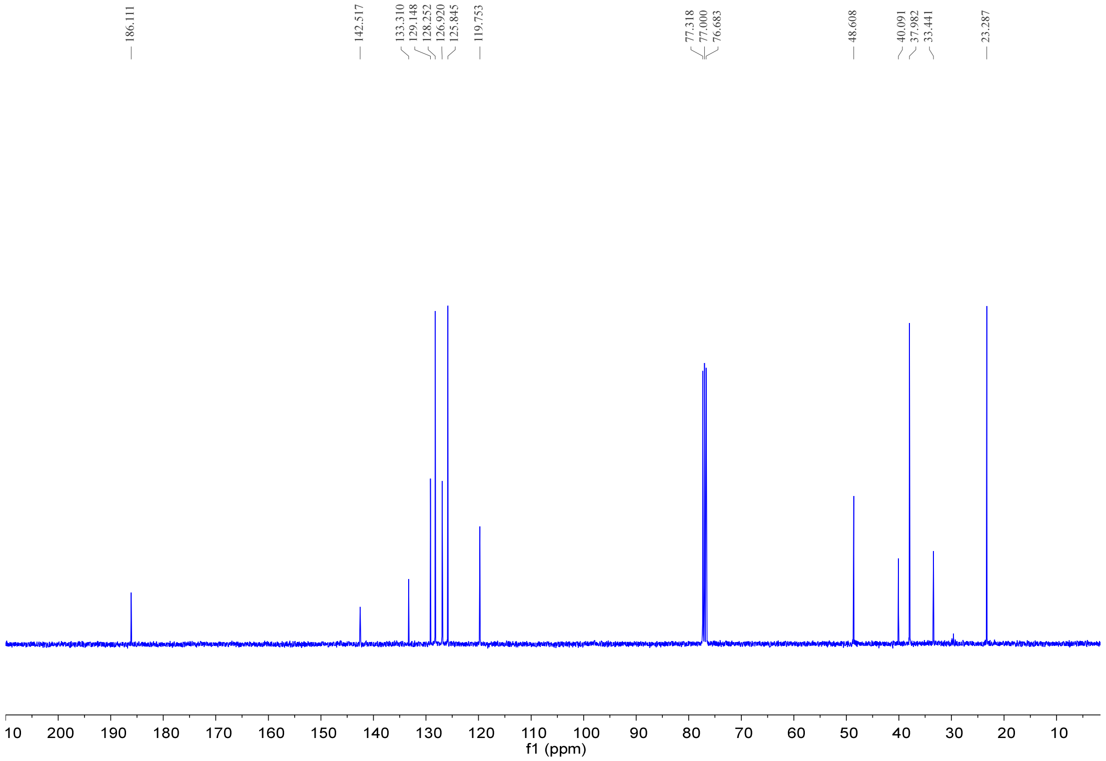

Supplementary Figure 33. ^{13}C NMR (100 MHz, $CDCl_3$) spectrum of compound **1l**

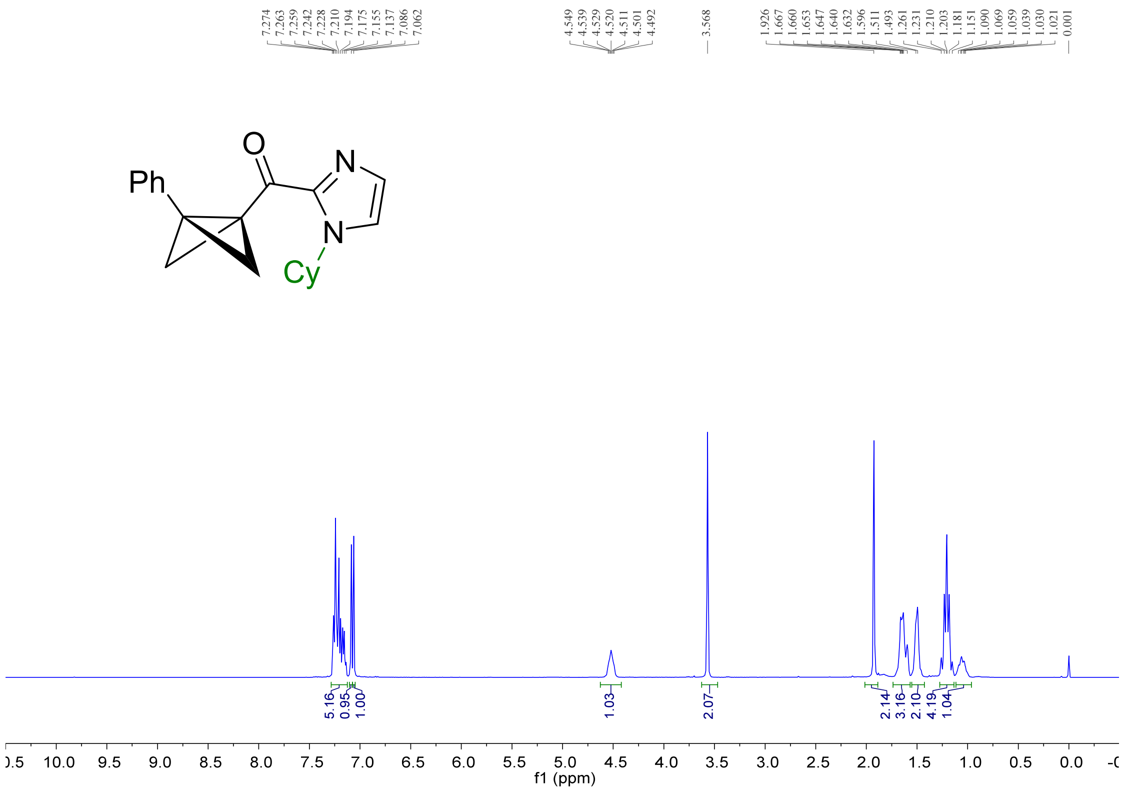

Supplementary Figure 34. ^{1}H NMR (400 MHz, $CDCl_3$) spectrum of compound **1m**

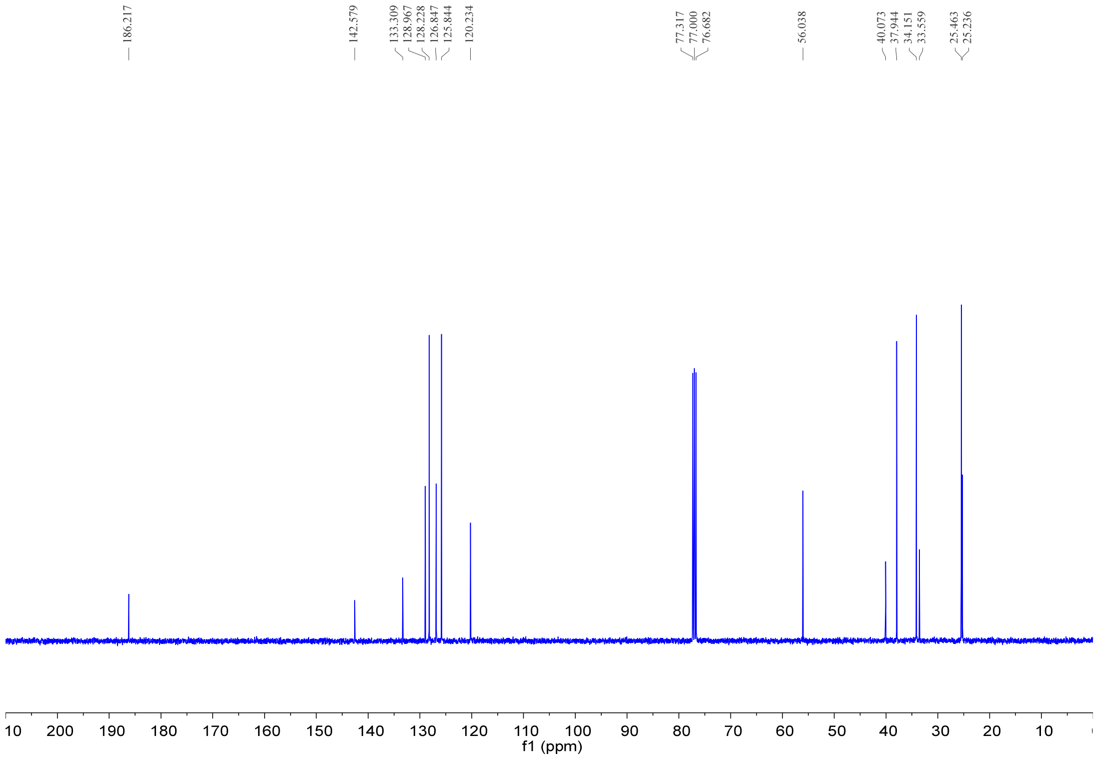

Supplementary Figure 35. ^{13}C NMR (100 MHz, $CDCl_3$) spectrum of compound **1m**

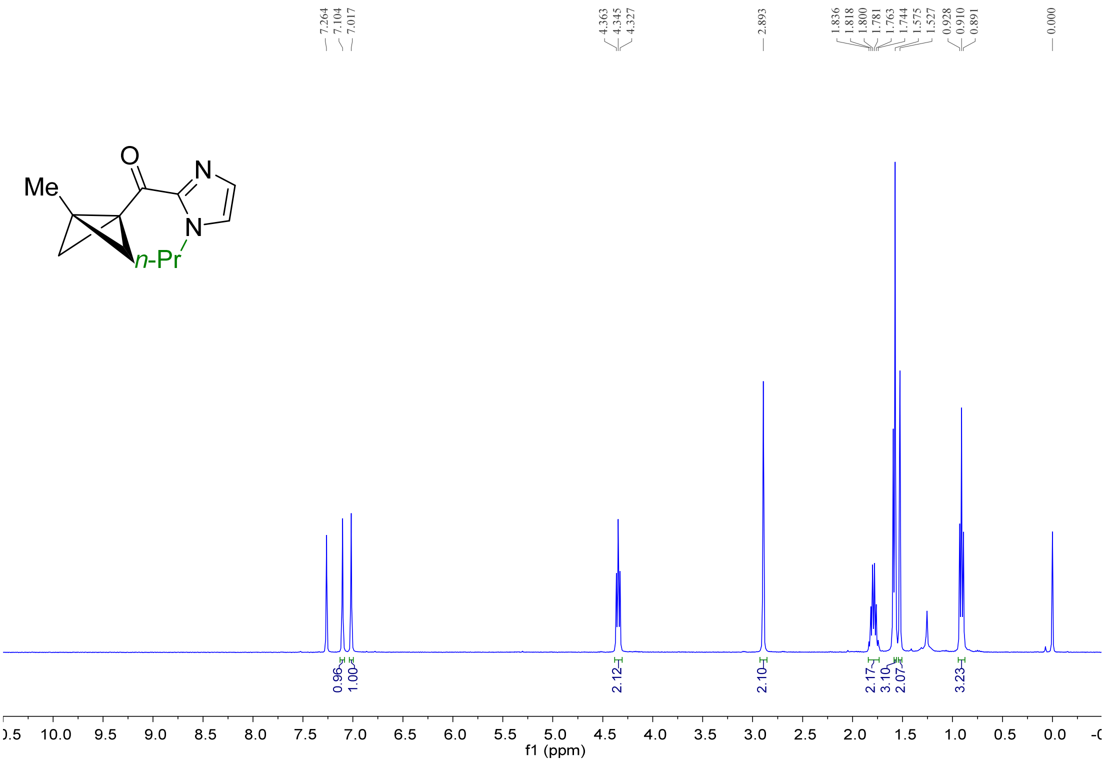

Supplementary Figure 36. ^{1}H NMR (400 MHz, $CDCl_3$) spectrum of compound **1q**

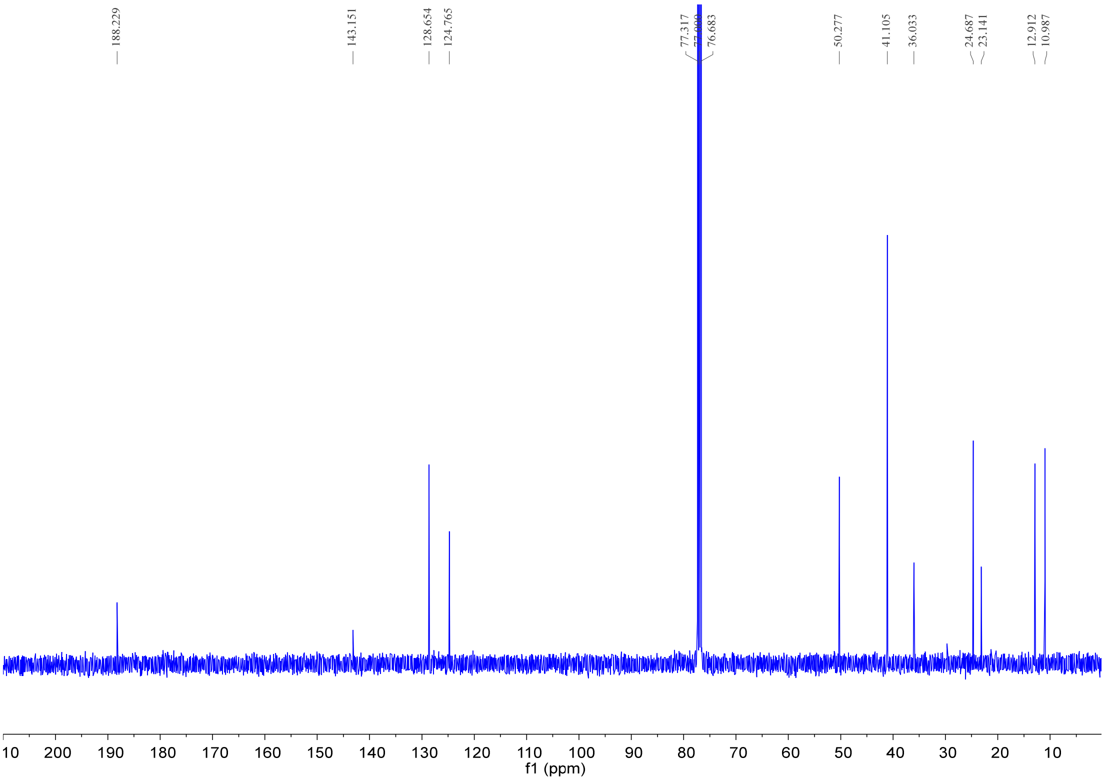

Supplementary Figure 37. ^{13}C NMR (100 MHz, $CDCl_3$) spectrum of compound **1q**

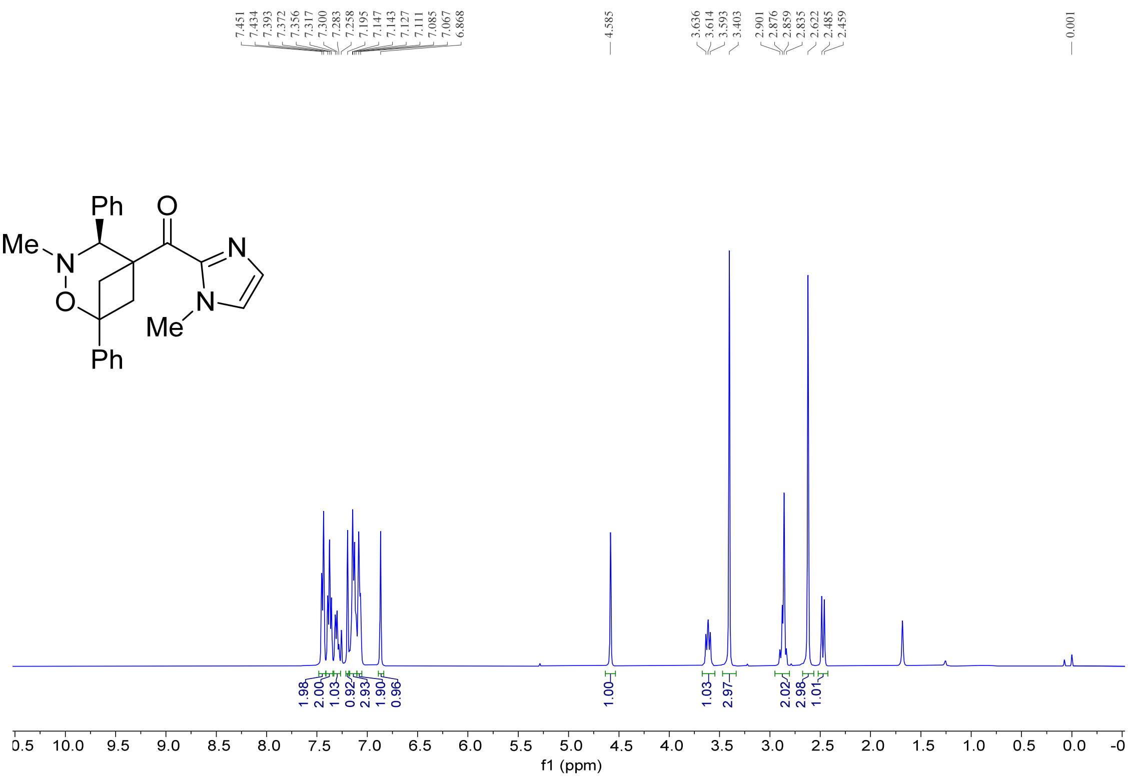

Supplementary Figure 38. ^{1}H NMR (400 MHz, $CDCl_3$) spectrum of compound **3aa**

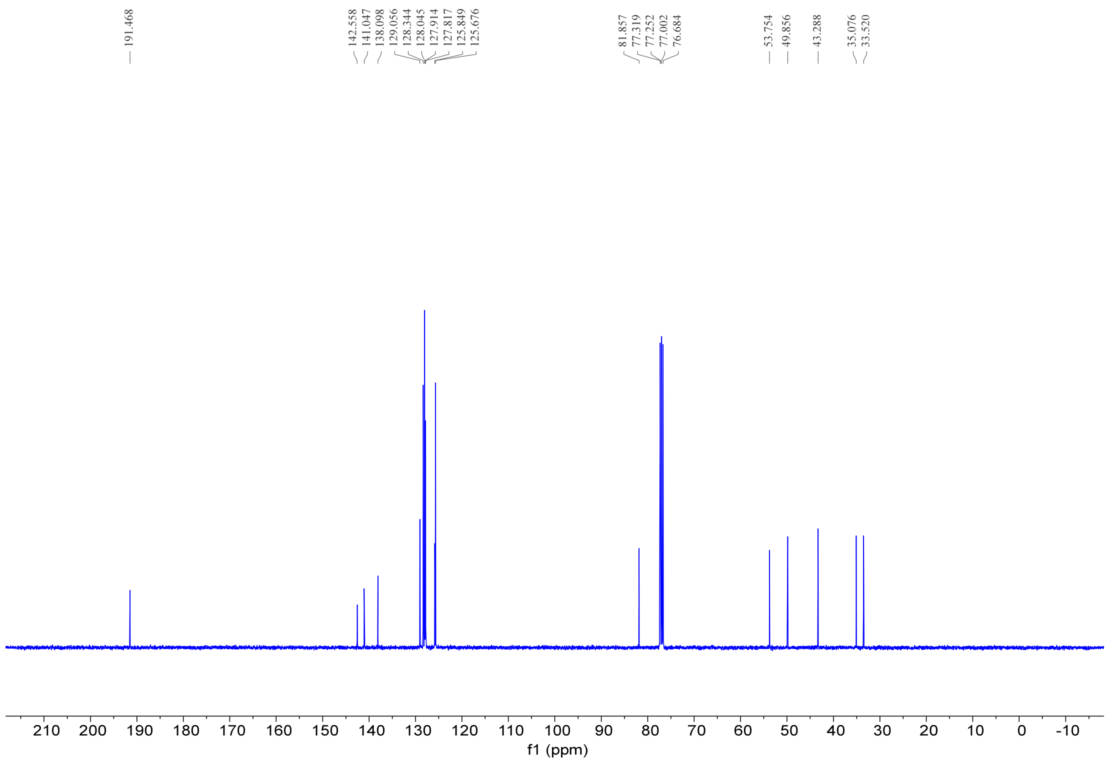

Supplementary Figure 39. ^{13}C NMR (100 MHz, $CDCl_3$) spectrum of compound **3aa**

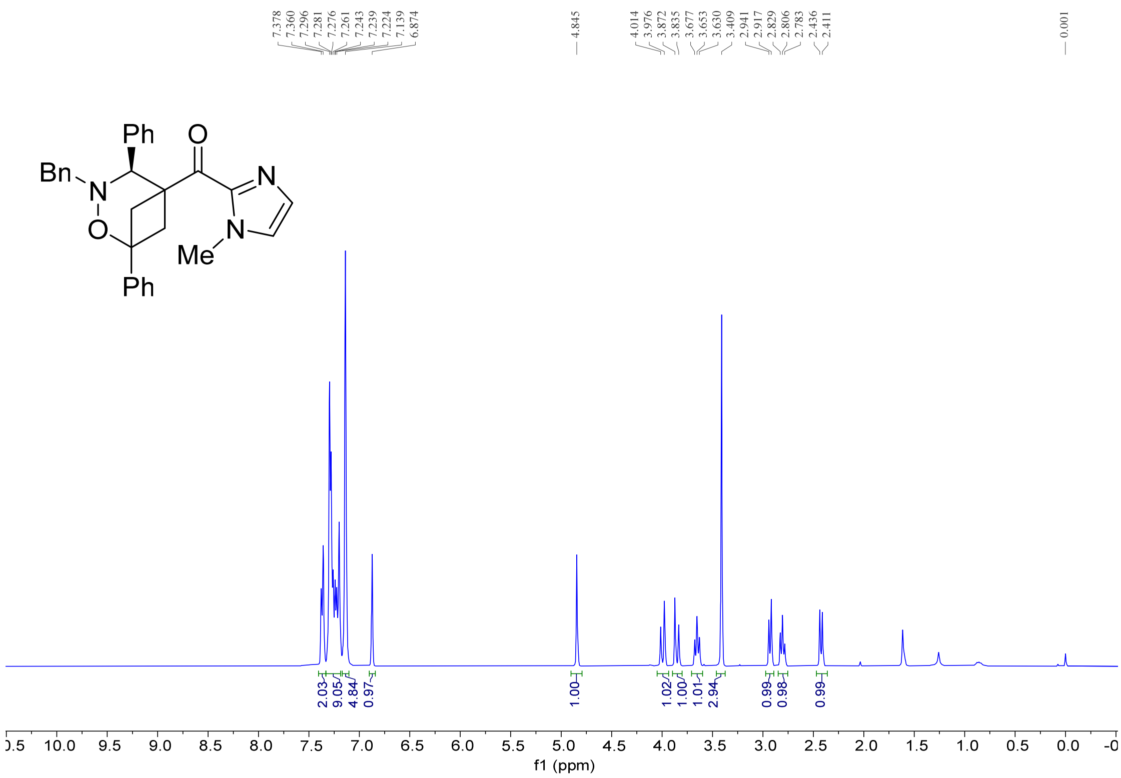

Supplementary Figure 40. ^{1}H NMR (400 MHz, $CDCl_3$) spectrum of compound **3ab**

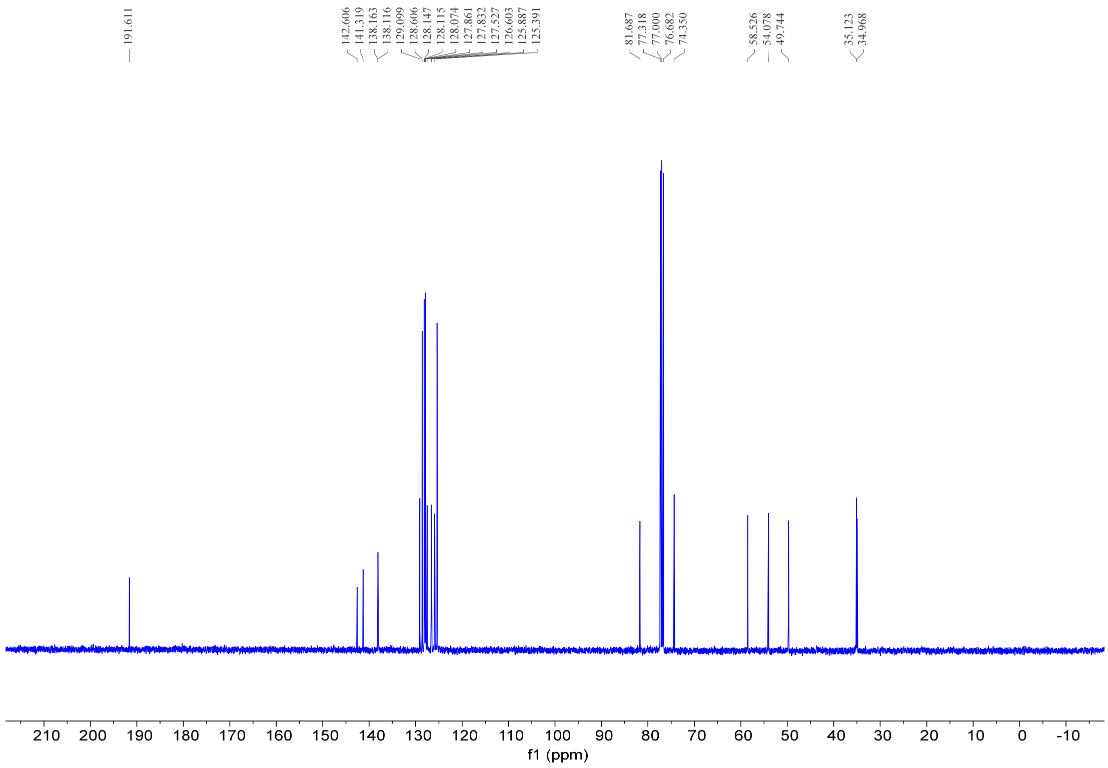

Supplementary Figure 41. ^{13}C NMR (100 MHz, $CDCl_3$) spectrum of compound **3ab**

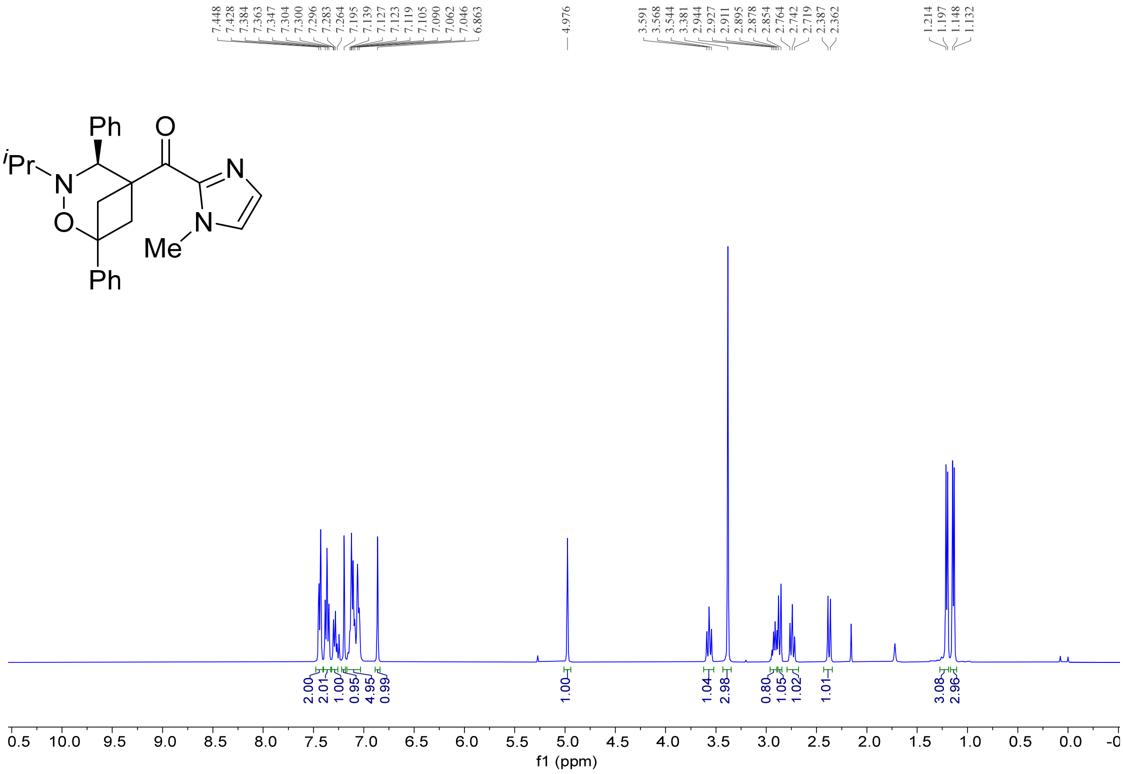

Supplementary Figure 42. ^{1}H NMR (400 MHz, $CDCl_3$) spectrum of compound **3ac**

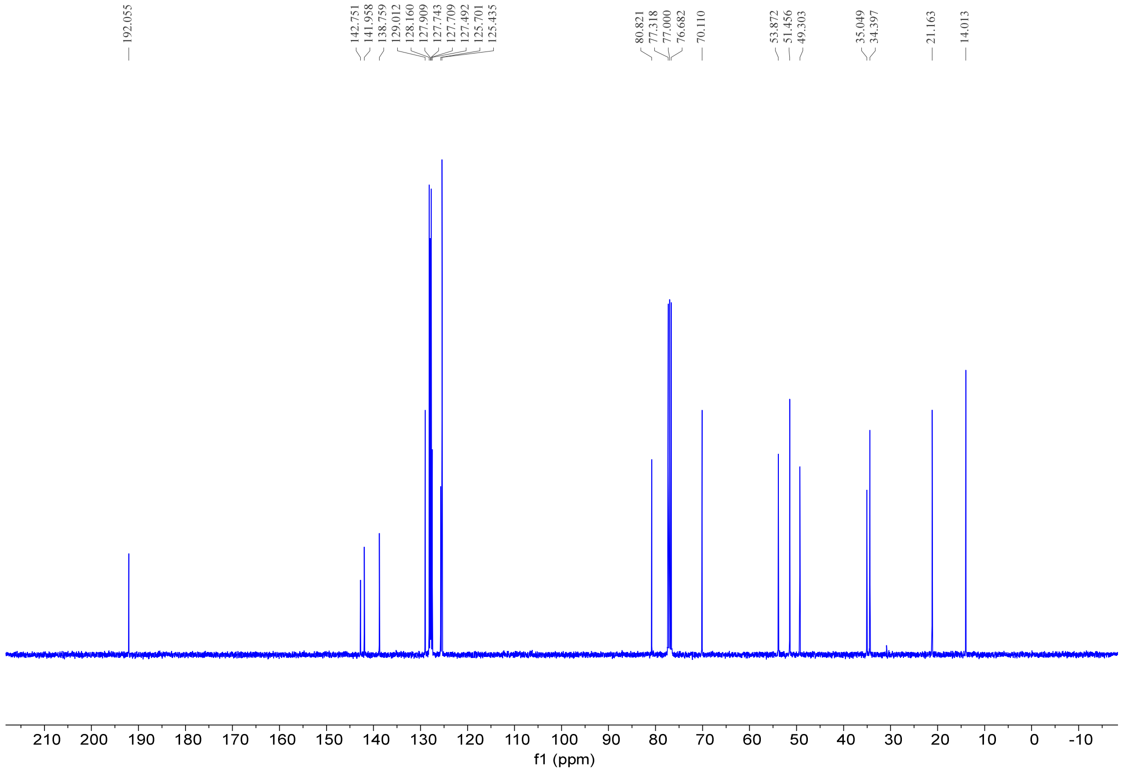

Supplementary Figure 43. ^{13}C NMR (100 MHz, $CDCl_3$) spectrum of compound **3ac**

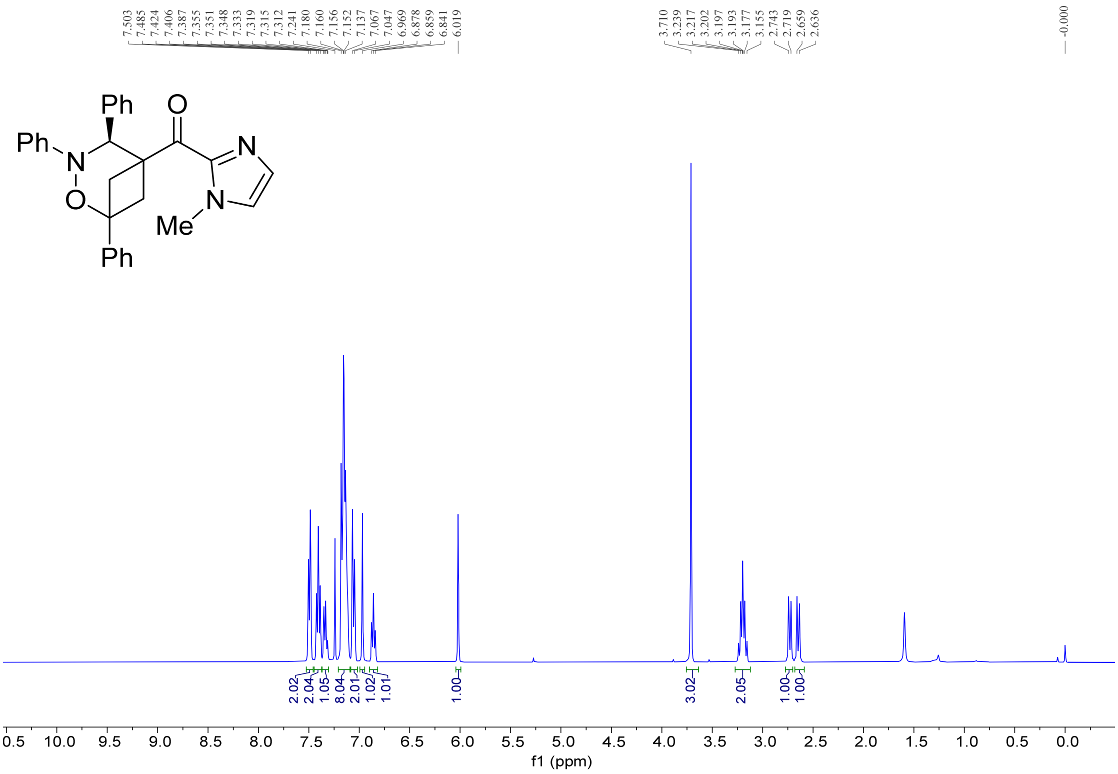

Supplementary Figure 44. ^{1}H NMR (400 MHz, $CDCl_3$) spectrum of compound **3ad**

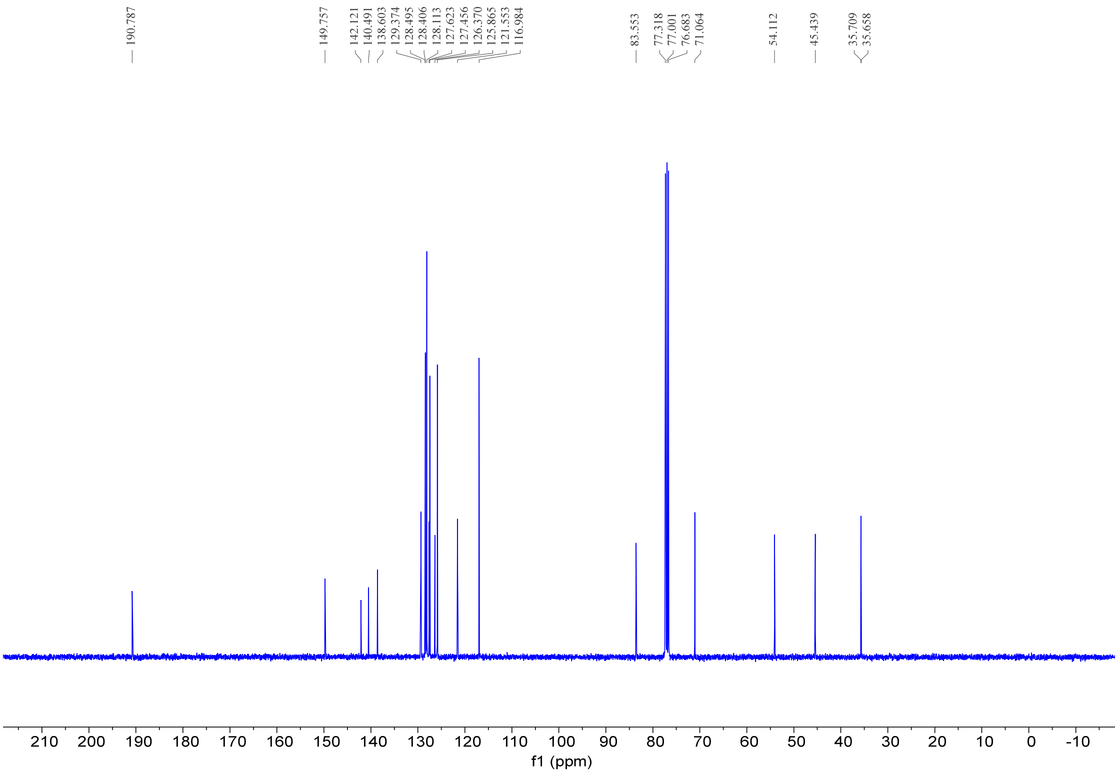

Supplementary Figure 45. ^{13}C NMR (100 MHz, $CDCl_3$) spectrum of compound **3ad**

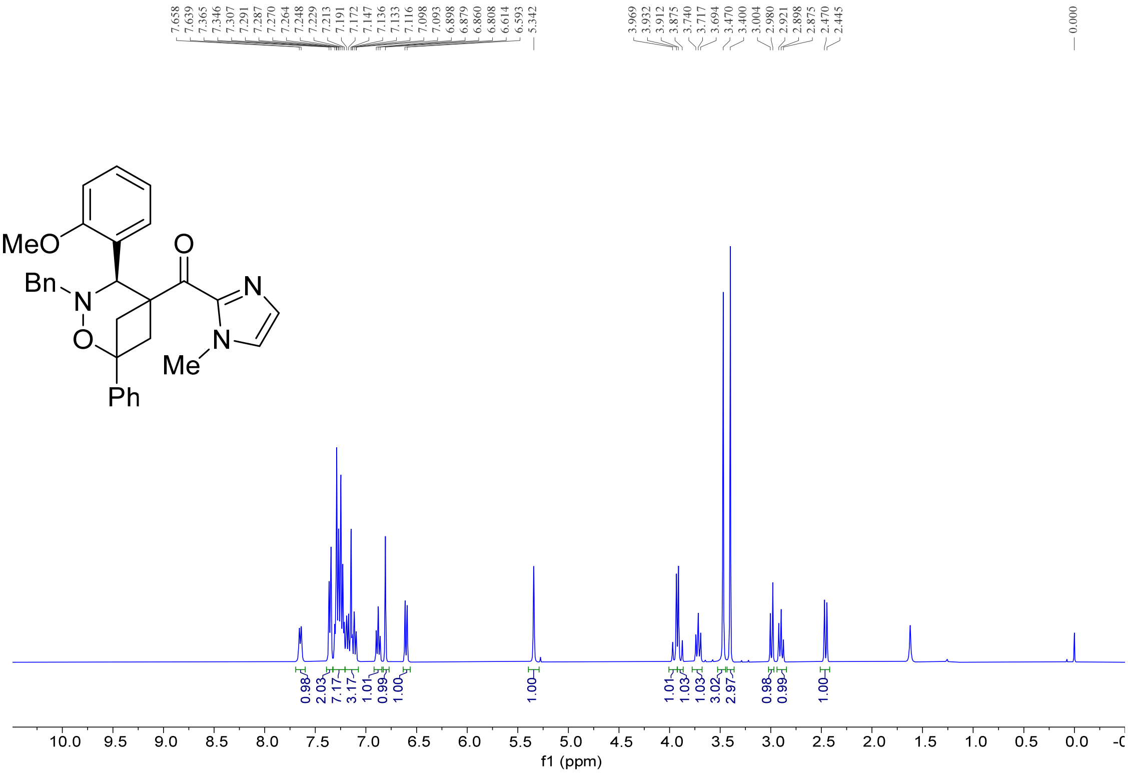

Supplementary Figure 46. ^{1}H NMR (400 MHz, $CDCl_3$) spectrum of compound **3ae**

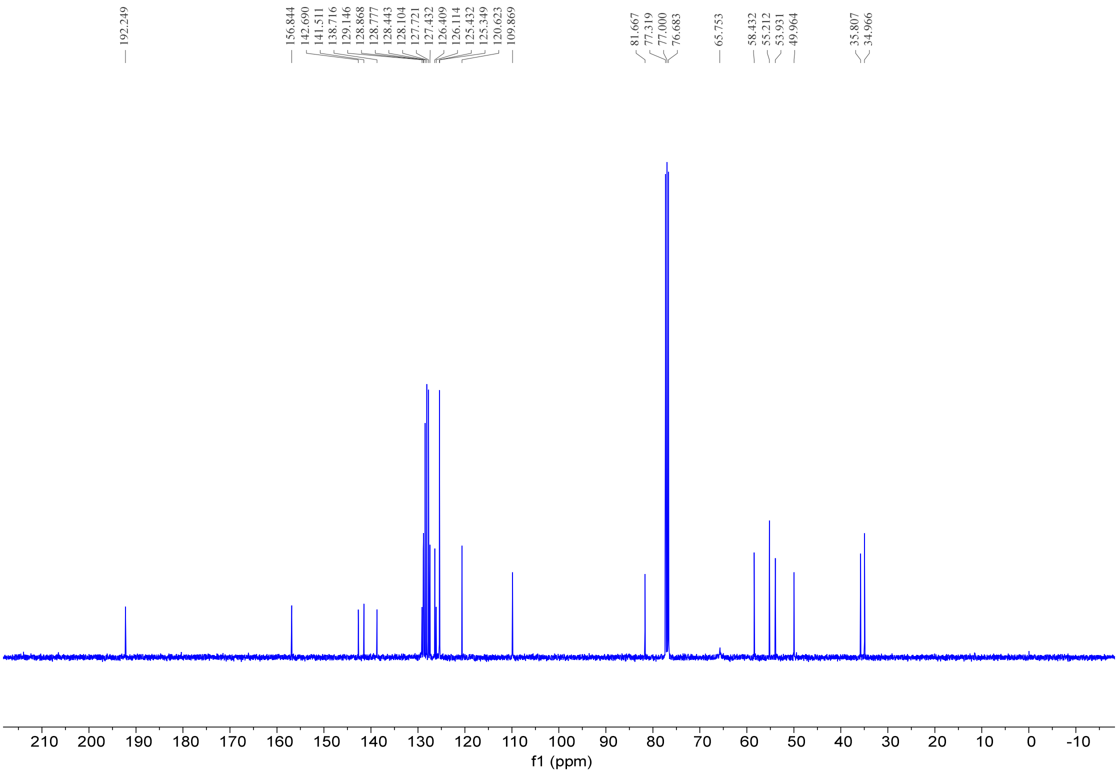

Supplementary Figure 47. ^{13}C NMR (100 MHz, $CDCl_3$) spectrum of compound **3ae**

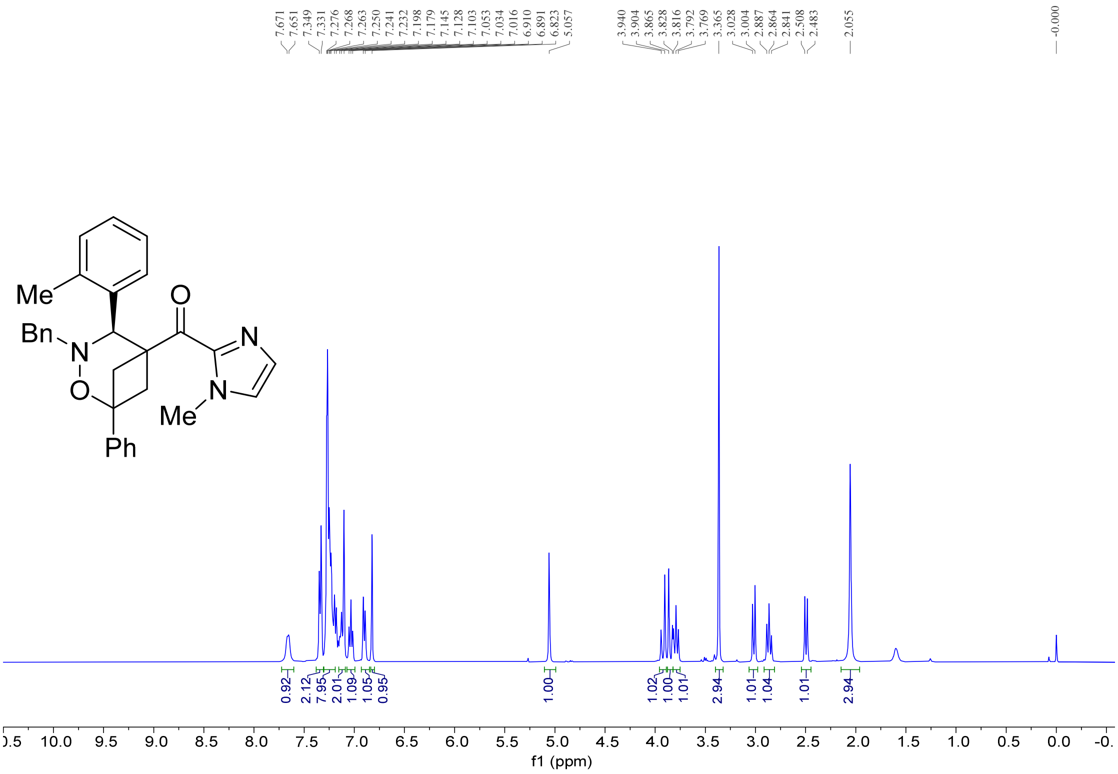

Supplementary Figure 48. ^{1}H NMR (400 MHz, $CDCl_3$) spectrum of compound **3af**

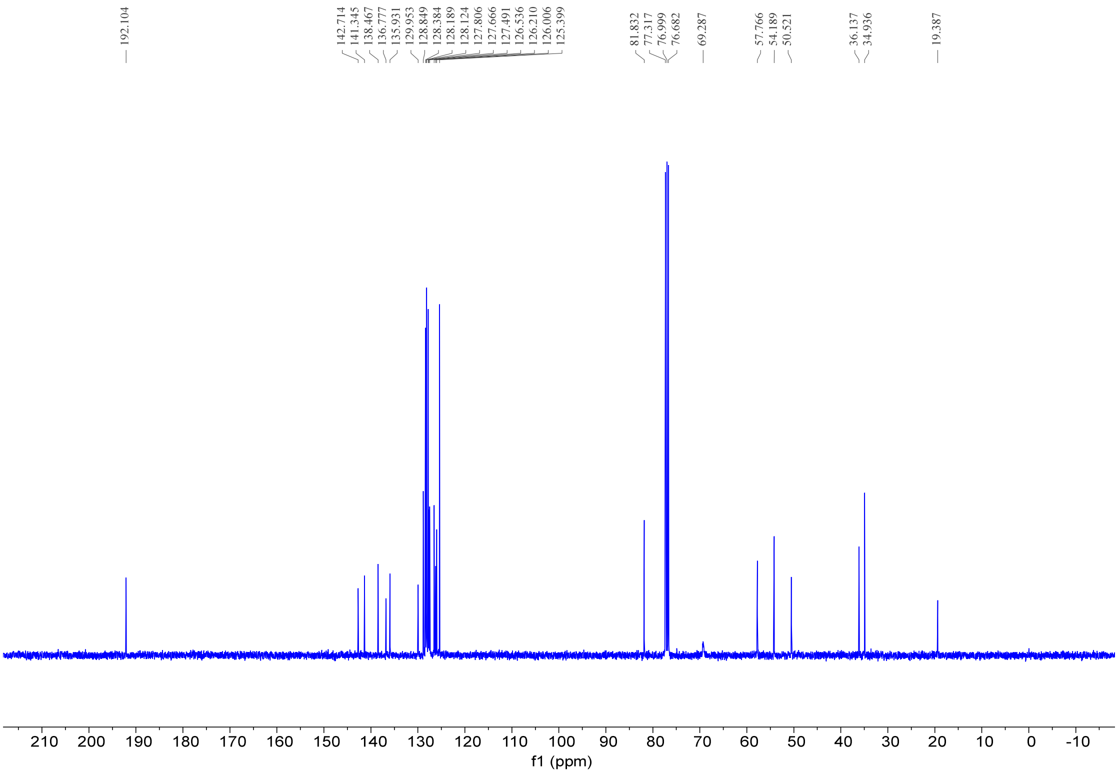

Supplementary Figure 49. ^{13}C NMR (100 MHz, $CDCl_3$) spectrum of compound **3af**

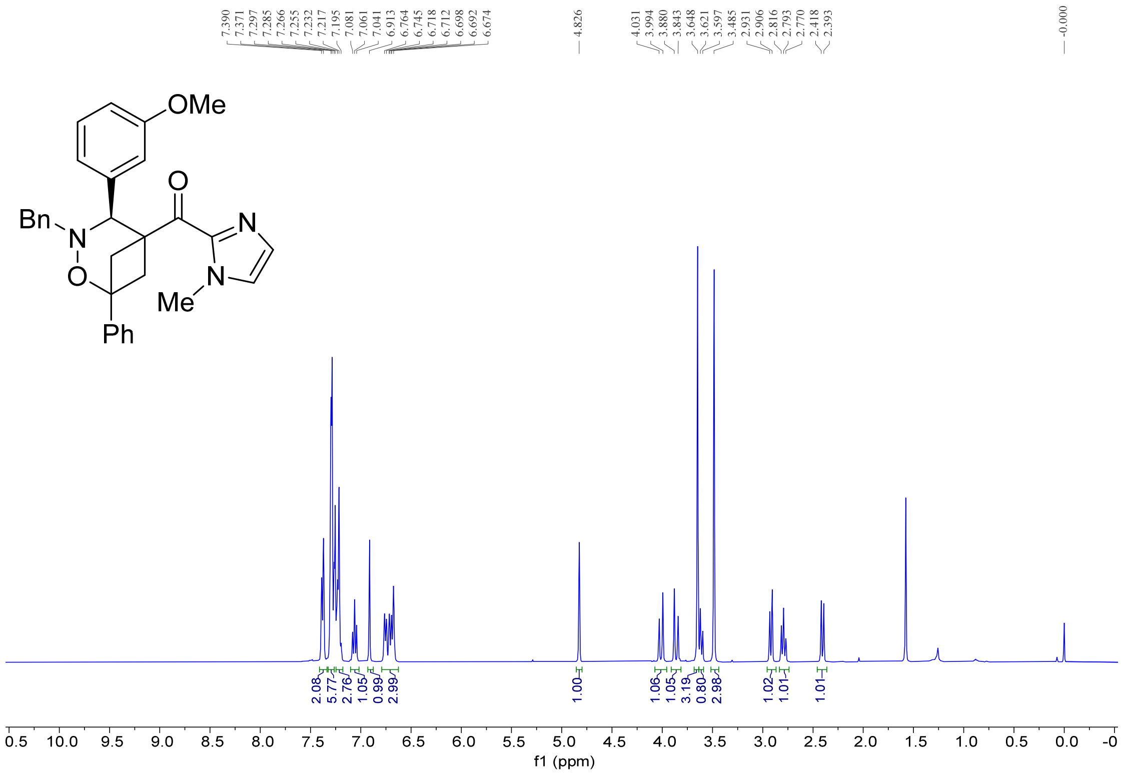

Supplementary Figure 50. ^{1}H NMR (400 MHz, $CDCl_3$) spectrum of compound **3ag**

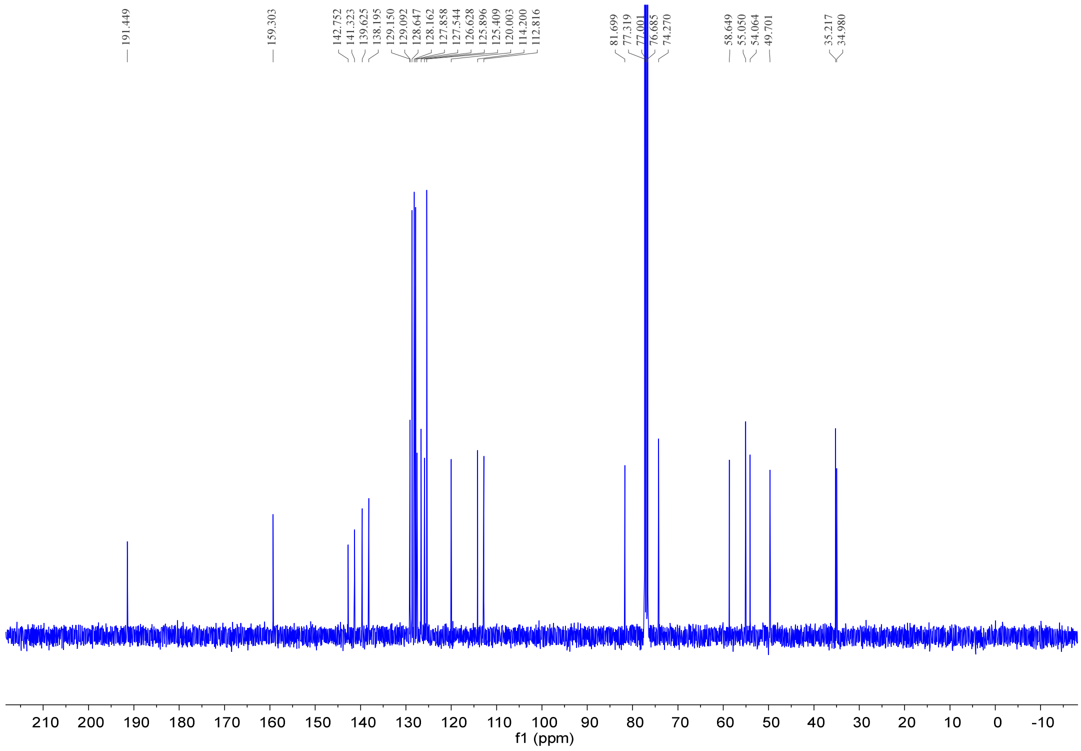

Supplementary Figure 51. ^{13}C NMR (100 MHz, $CDCl_3$) spectrum of compound **3ag**

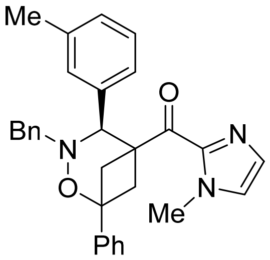

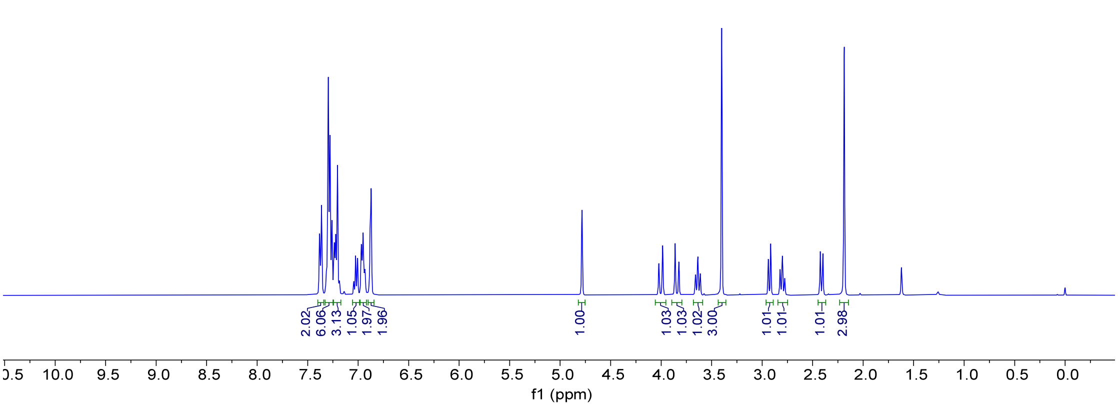

Supplementary Figure 52. ^{1}H NMR (400 MHz, $CDCl_3$) spectrum of compound **3ah**

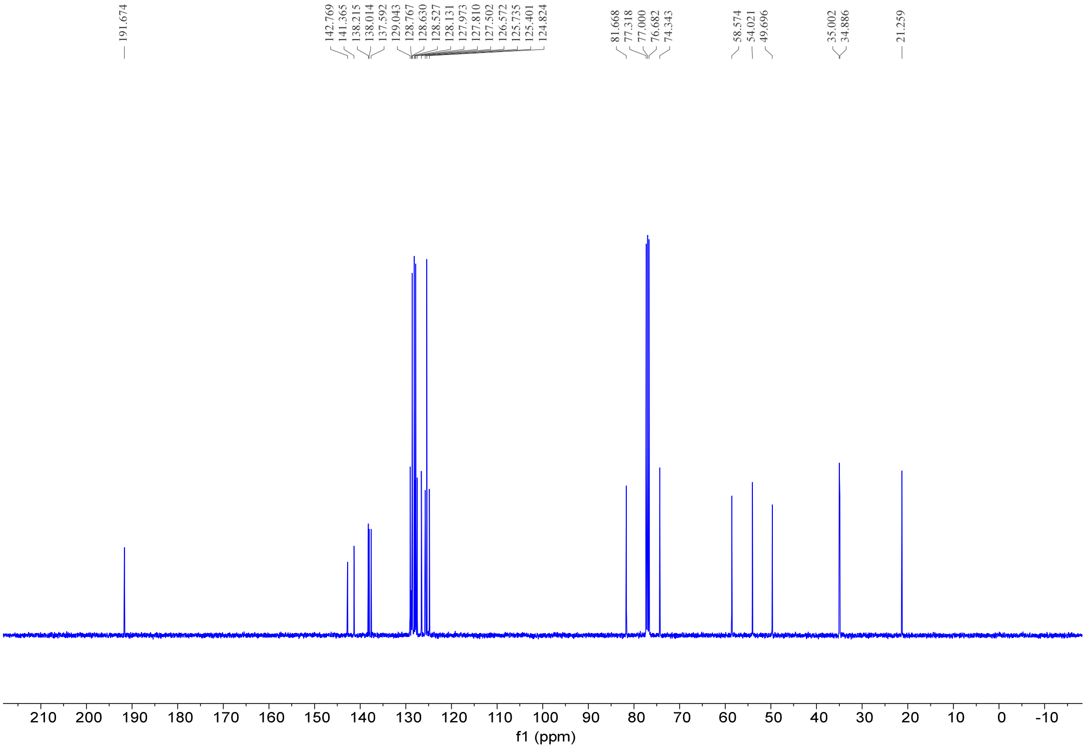

Supplementary Figure 53. ^{13}C NMR (100 MHz, $CDCl_3$) spectrum of compound **3ah**

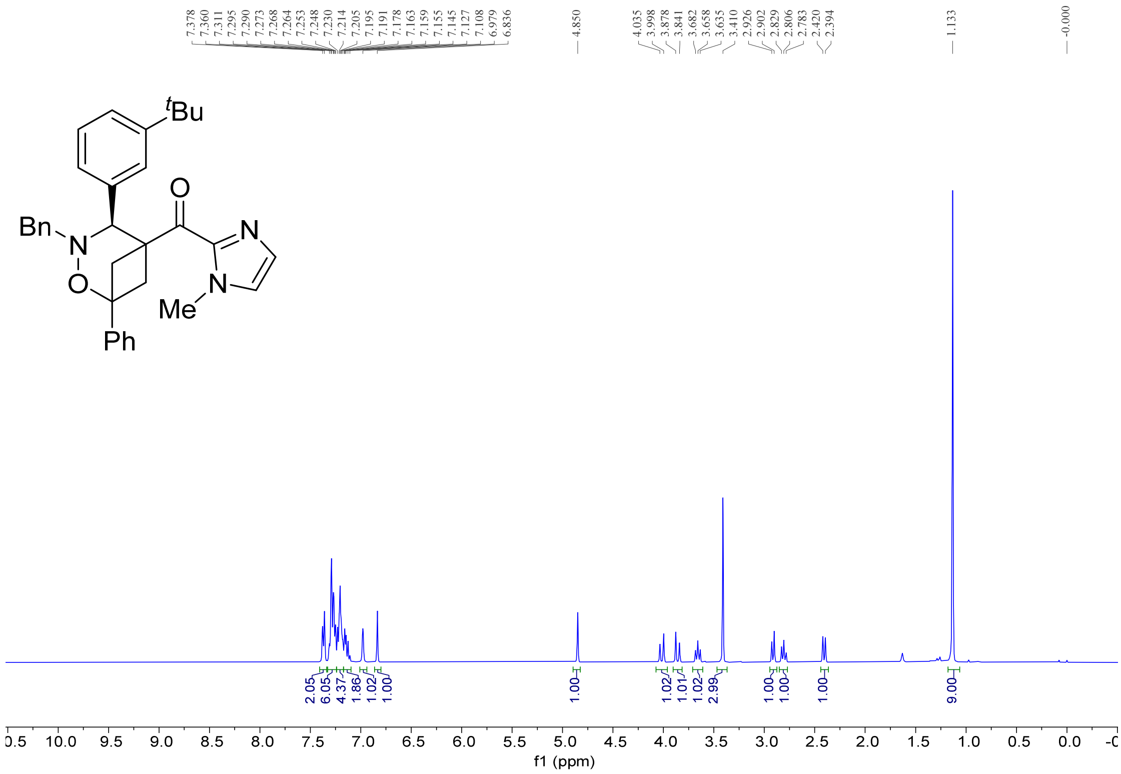

Supplementary Figure 54. ^{1}H NMR (400 MHz, $CDCl_3$) spectrum of compound **3ai**

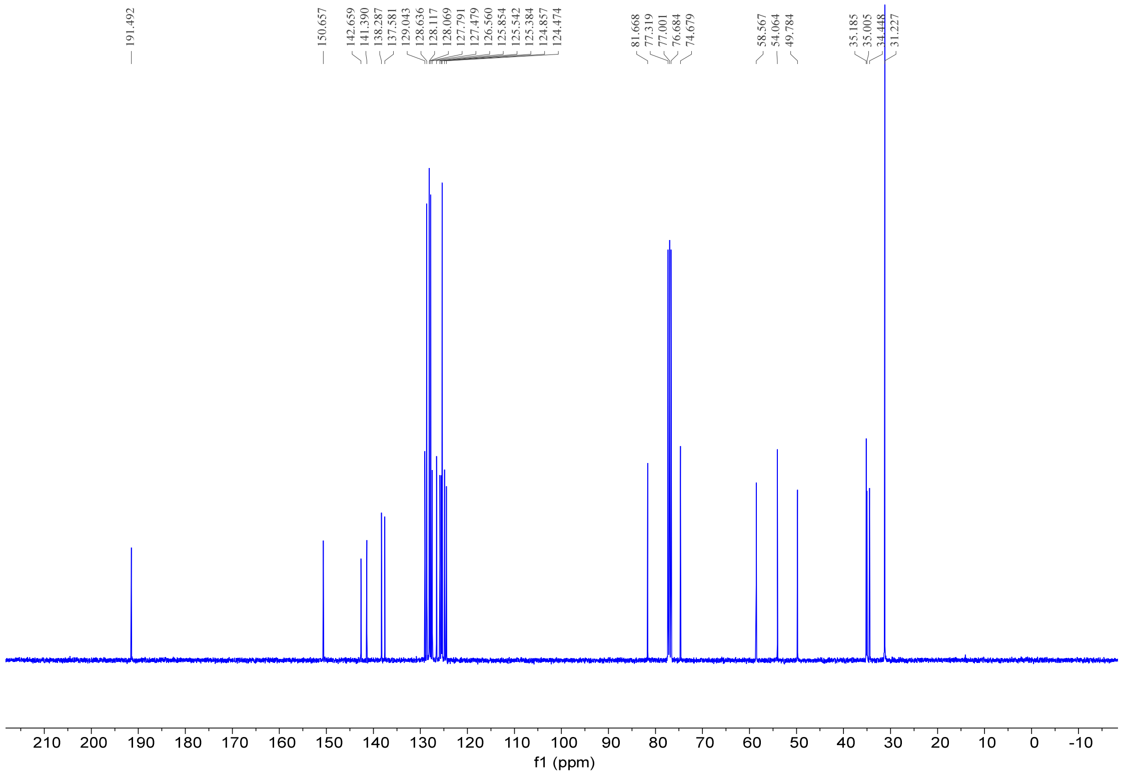

Supplementary Figure 55. ^{13}C NMR (100 MHz, $CDCl_3$) spectrum of compound **3ai**

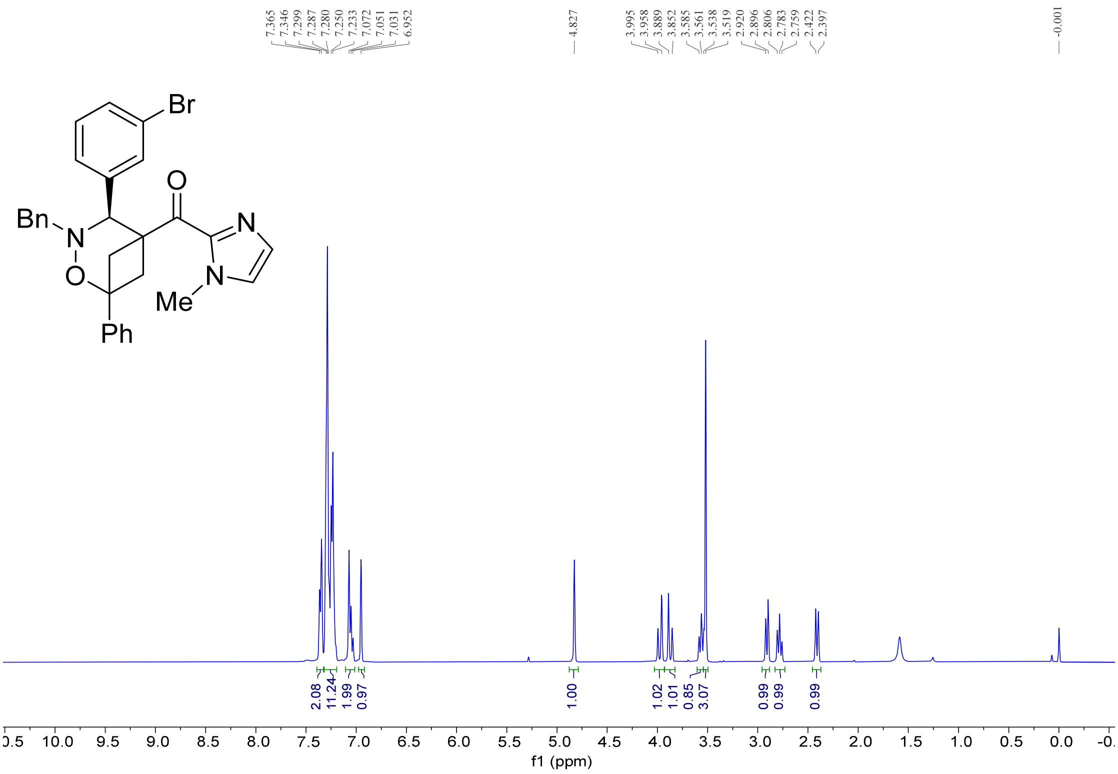

Supplementary Figure 56. ^{1}H NMR (400 MHz, $CDCl_3$) spectrum of compound **3aj**

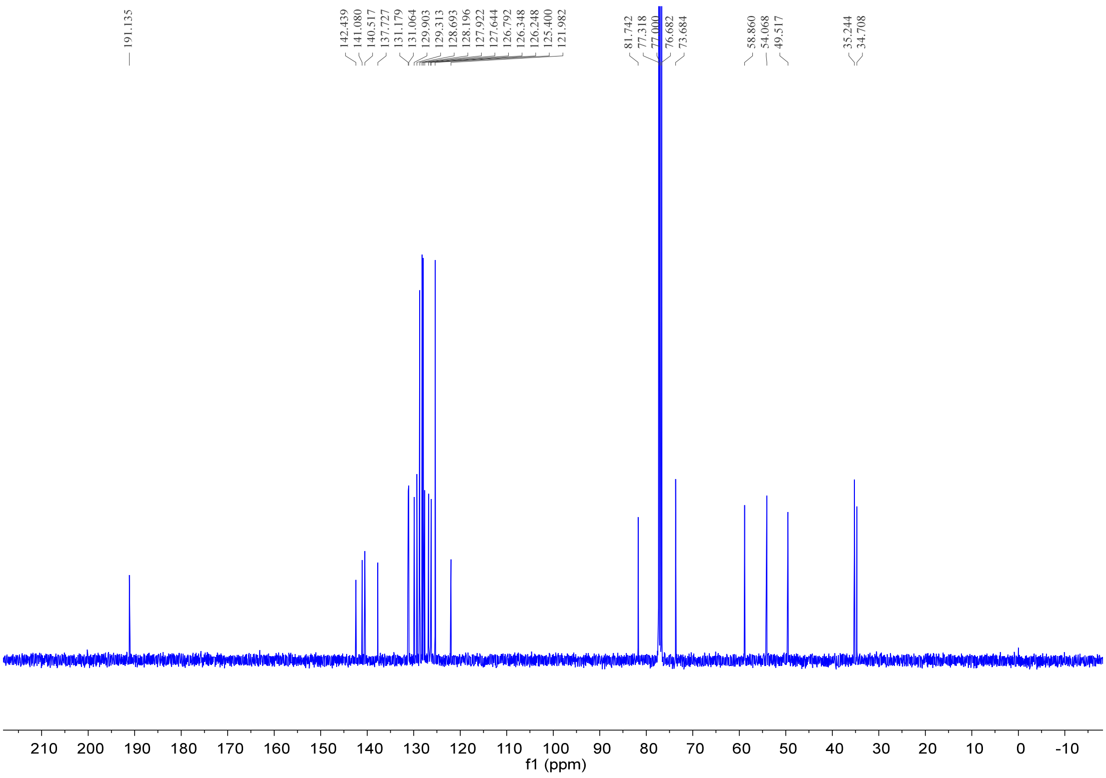

Supplementary Figure 57. ^{13}C NMR (100 MHz, $CDCl_3$) spectrum of compound **3aj**

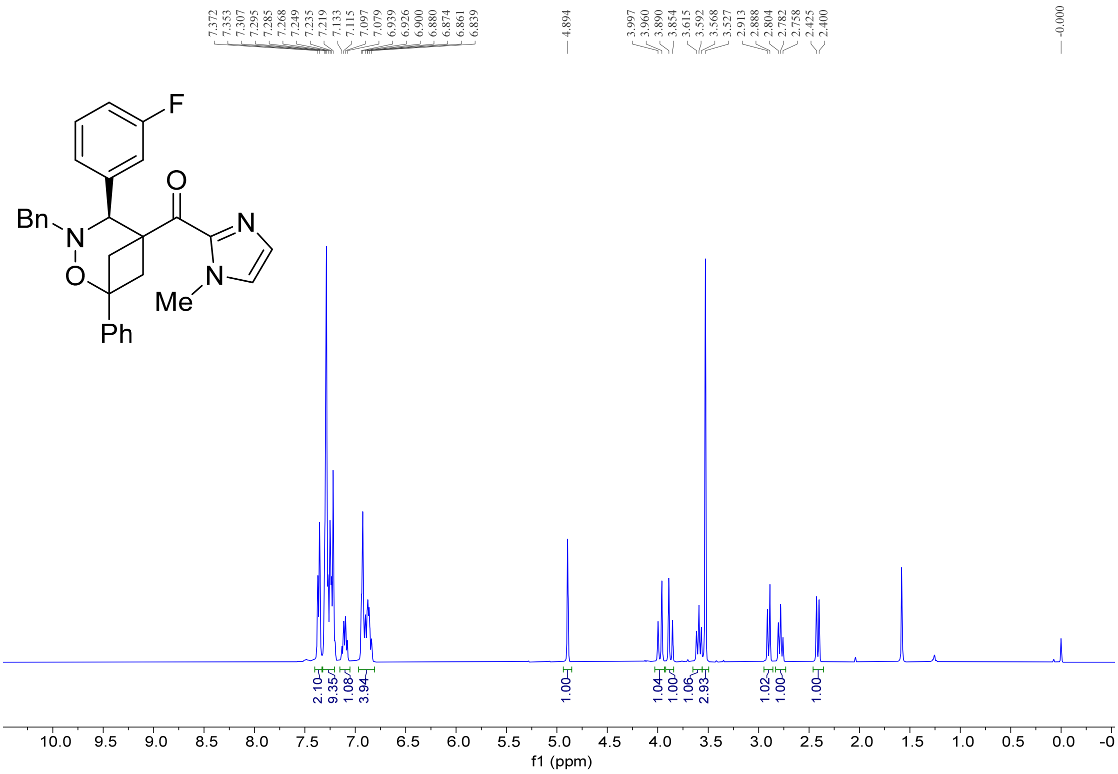

Supplementary Figure 58. ^{1}H NMR (400 MHz, $CDCl_3$) spectrum of compound **3ak**

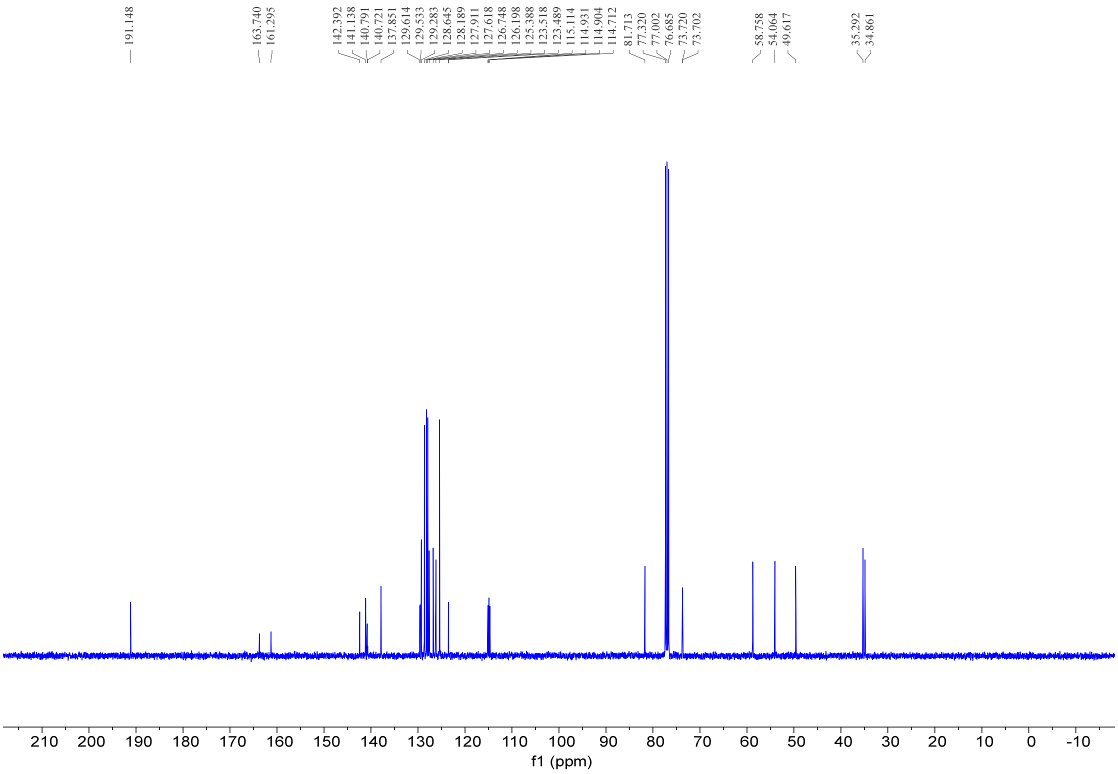

Supplementary Figure 59. ^{13}C NMR (100 MHz, $CDCl_3$) spectrum of compound **3ak**

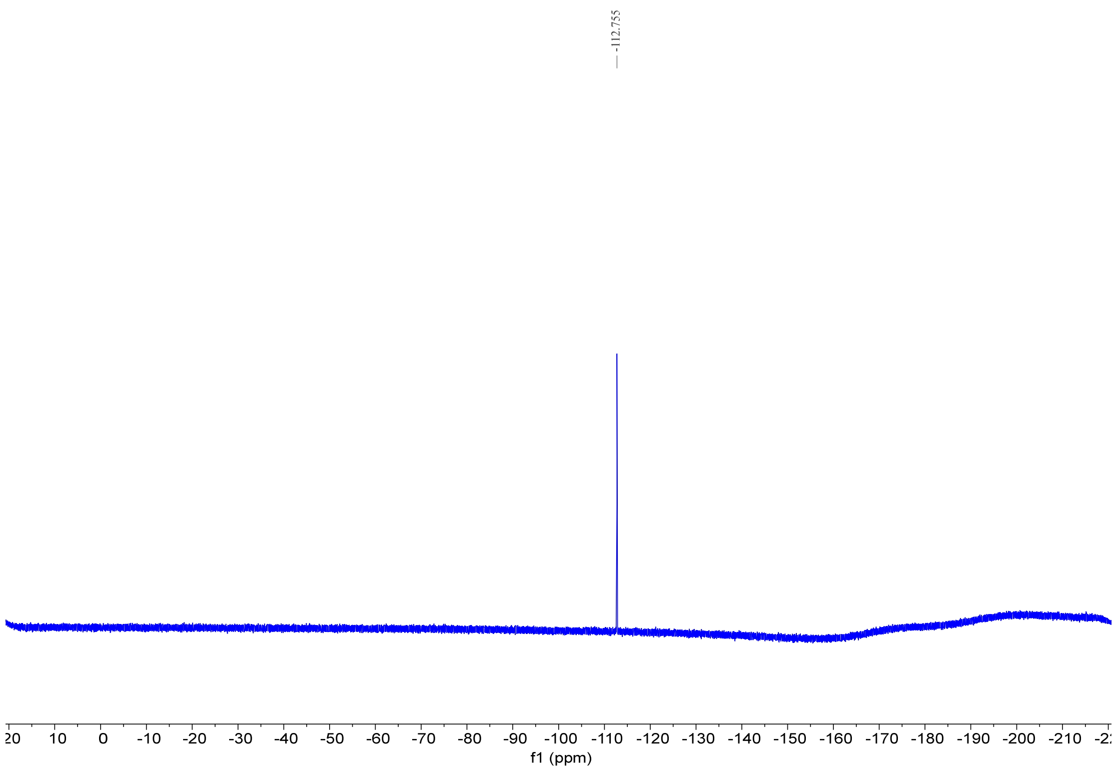

Supplementary Figure 60. ^{19}F NMR (376 MHz, $CDCl_3$) spectrum of compound **3ak**

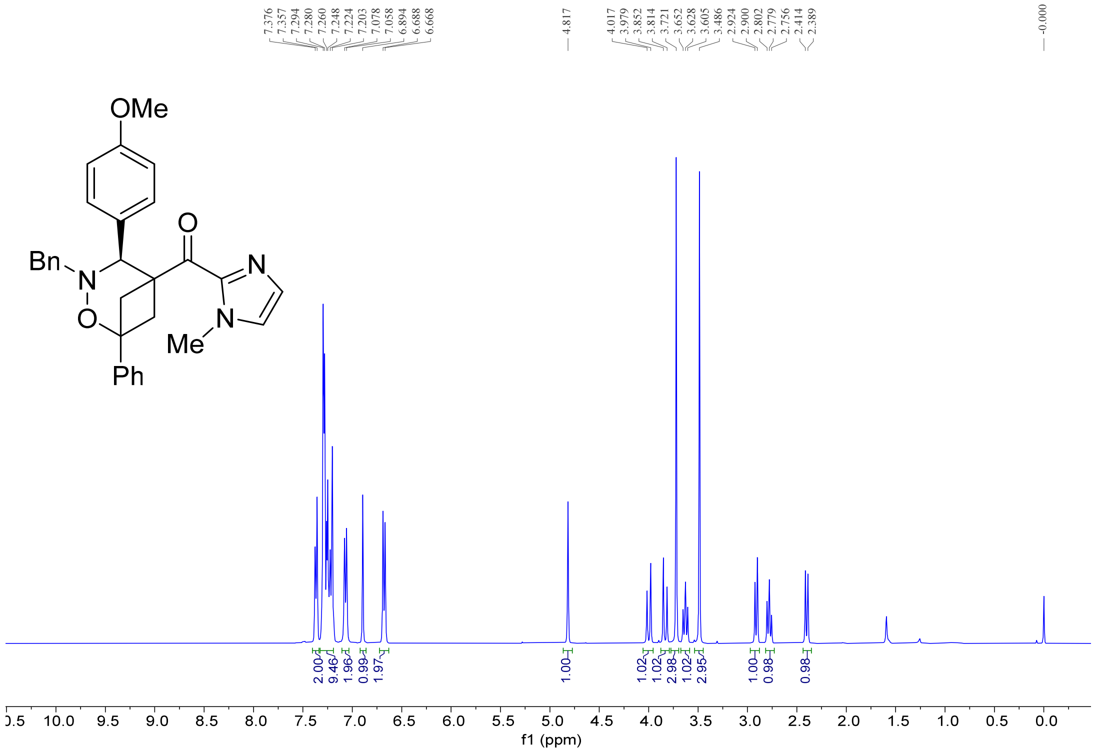

Supplementary Figure 61. ^{1}H NMR (400 MHz, $CDCl_3$) spectrum of compound **3al**

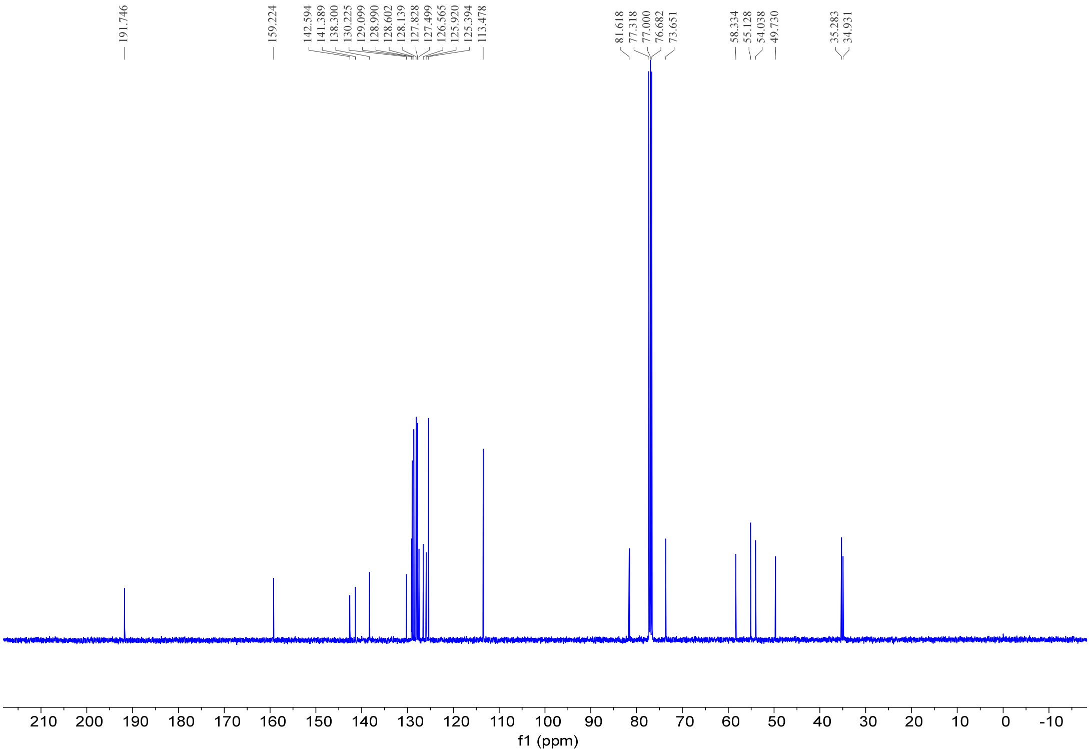

Supplementary Figure 62. ^{13}C NMR (100 MHz, $CDCl_3$) spectrum of compound **3al**

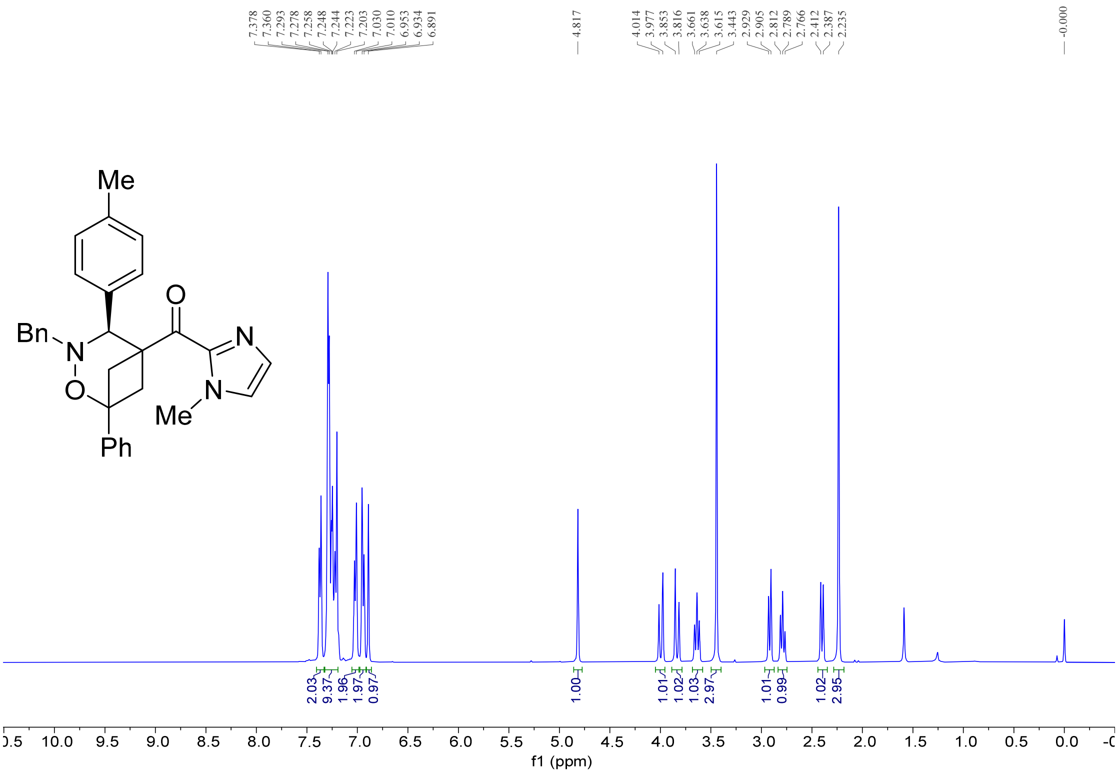

Supplementary Figure 63. ^{1}H NMR (400 MHz, $CDCl_3$) spectrum of compound **3am**

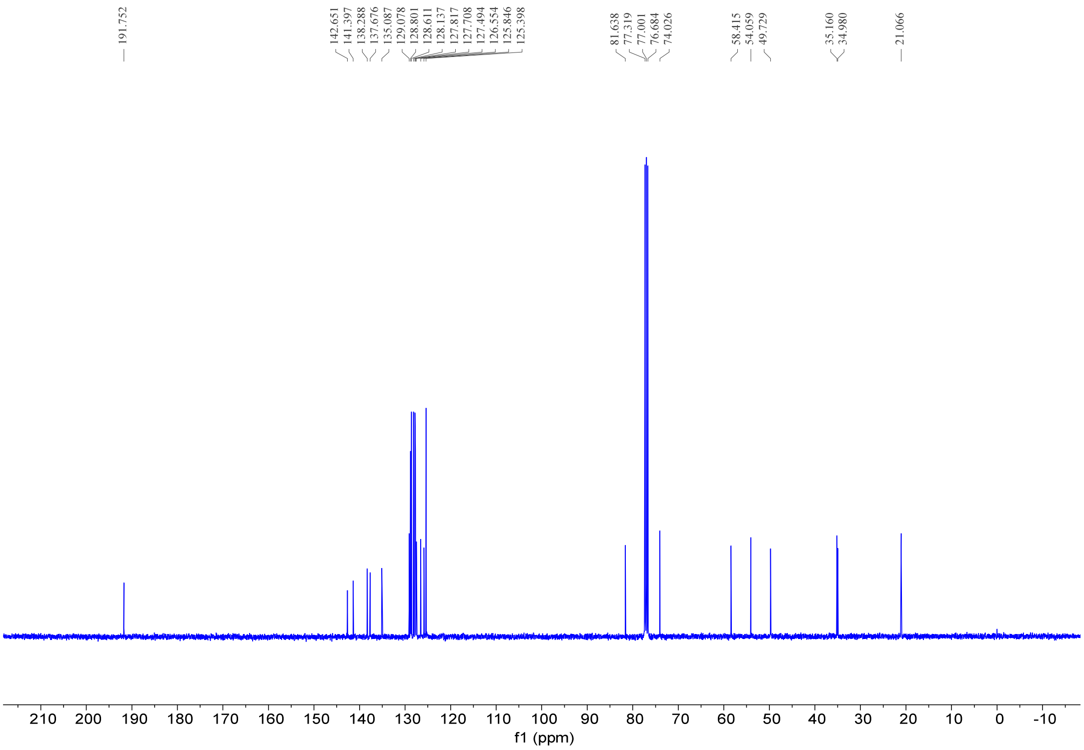

Supplementary Figure 64. ^{13}C NMR (100 MHz, $CDCl_3$) spectrum of compound **3am**

^{1}H and ^{13}C NMR Spectra for Compound 3an:

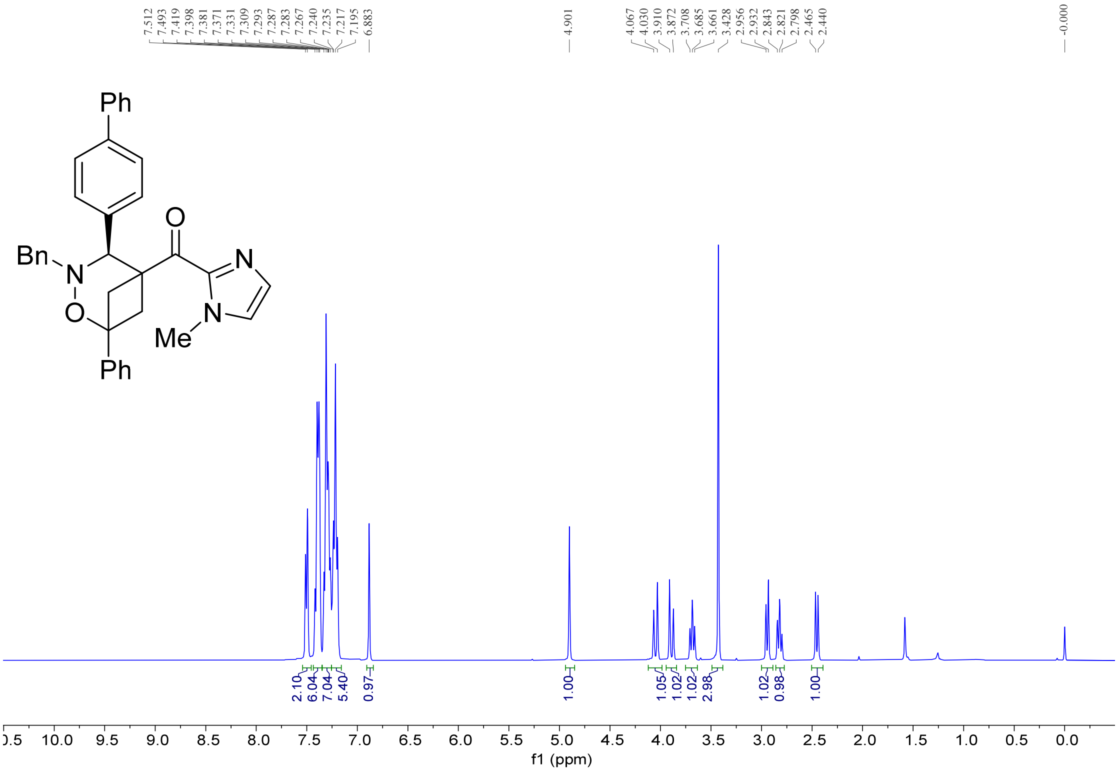

Supplementary Figure 65. ^{1}H NMR (400 MHz, $CDCl_3$) spectrum of compound **3an**

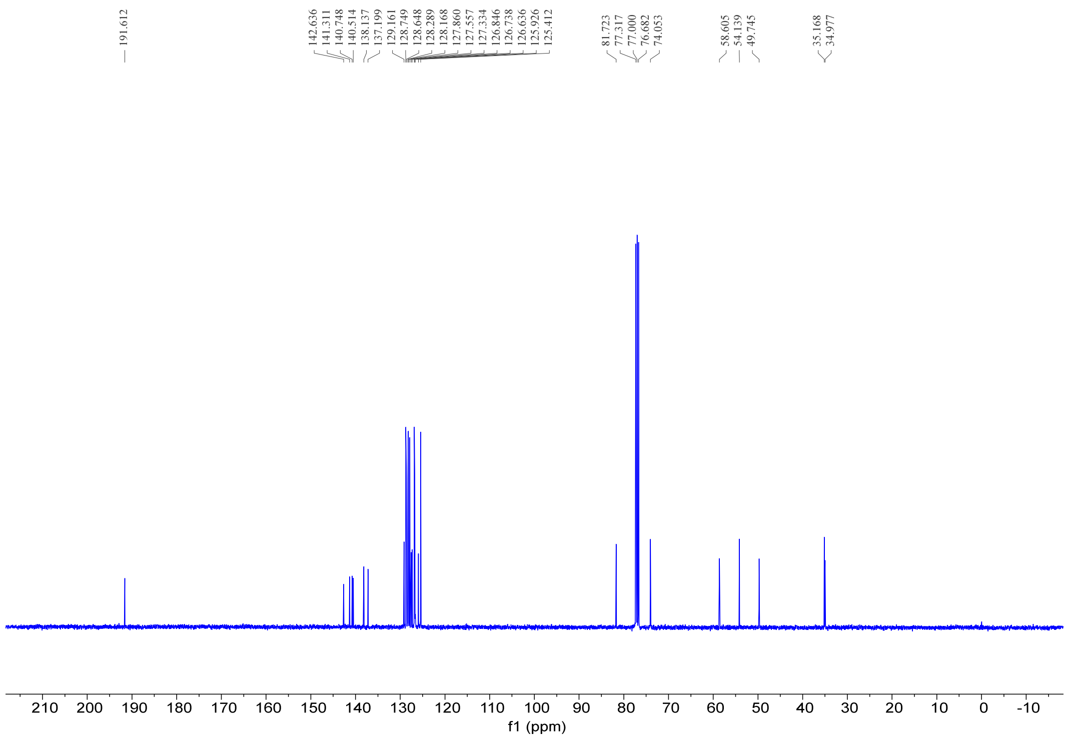

Supplementary Figure 66. ^{13}C NMR (100 MHz, $CDCl_3$) spectrum of compound **3an**

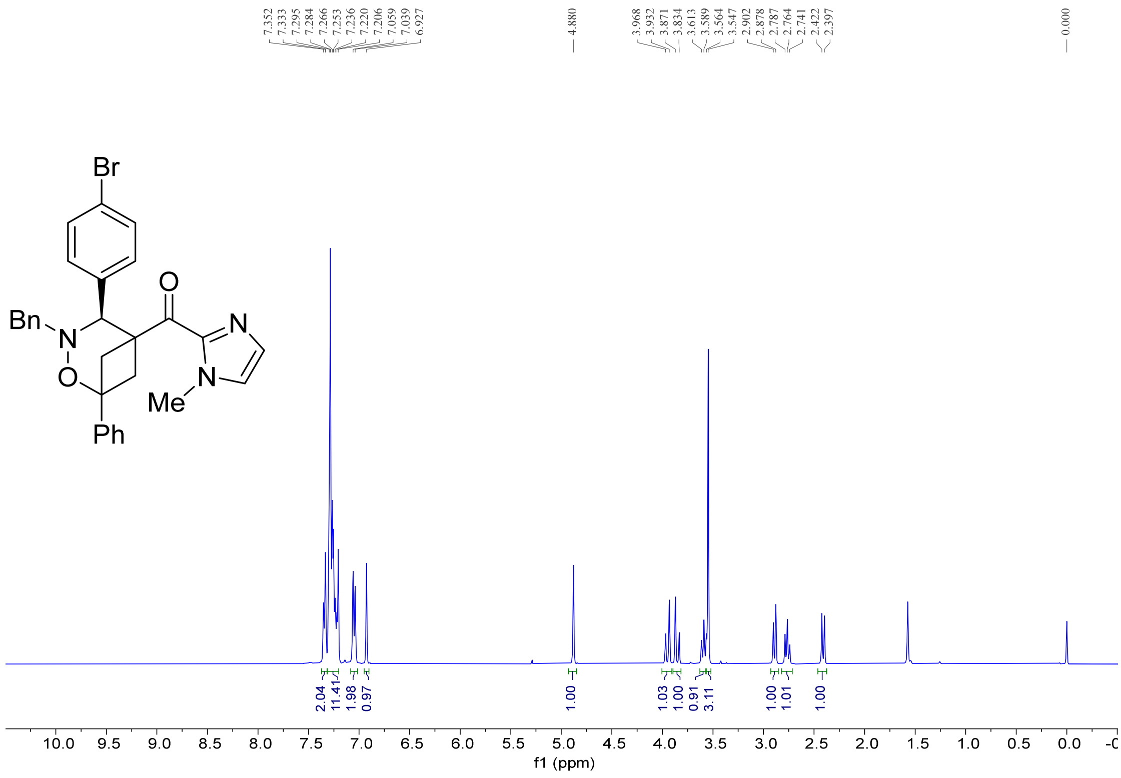

Supplementary Figure 67. ^{1}H NMR (400 MHz, $CDCl_3$) spectrum of compound **3ao**

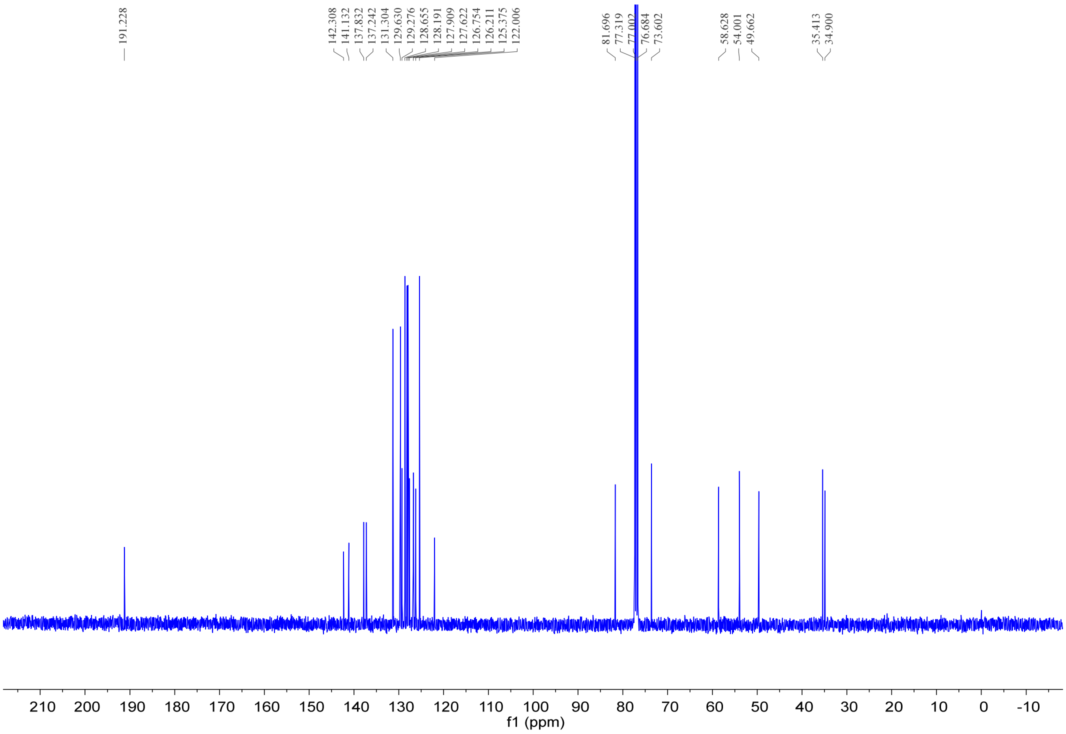

Supplementary Figure 68. ^{13}C NMR (100 MHz, $CDCl_3$) spectrum of compound **3ao**

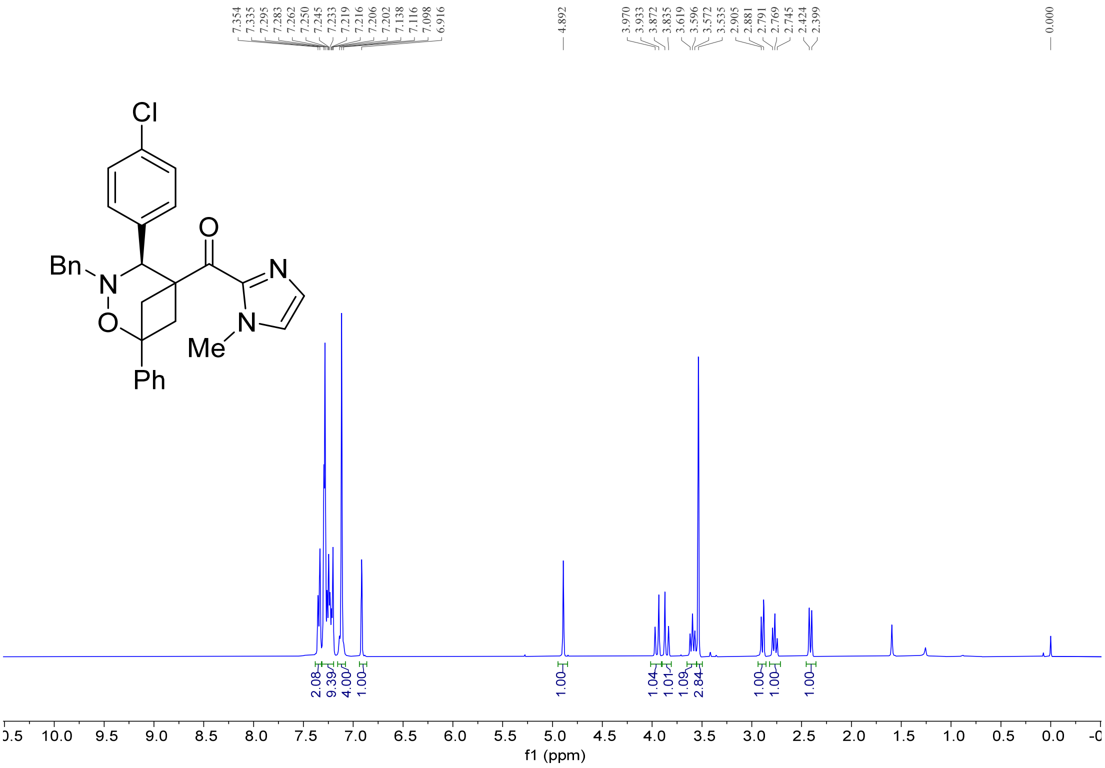

Supplementary Figure 69. ^{1}H NMR (400 MHz, $CDCl_3$) spectrum of compound **3ap**

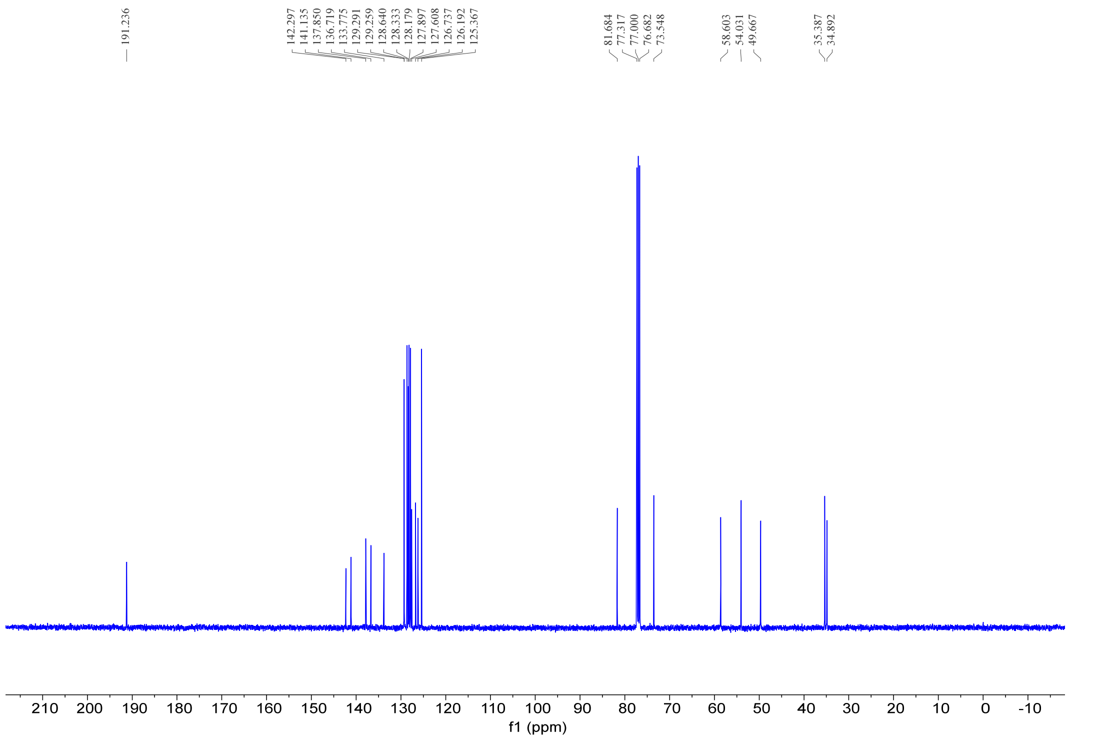

Supplementary Figure 70. ^{13}C NMR (100 MHz, $CDCl_3$) spectrum of compound **3ap**

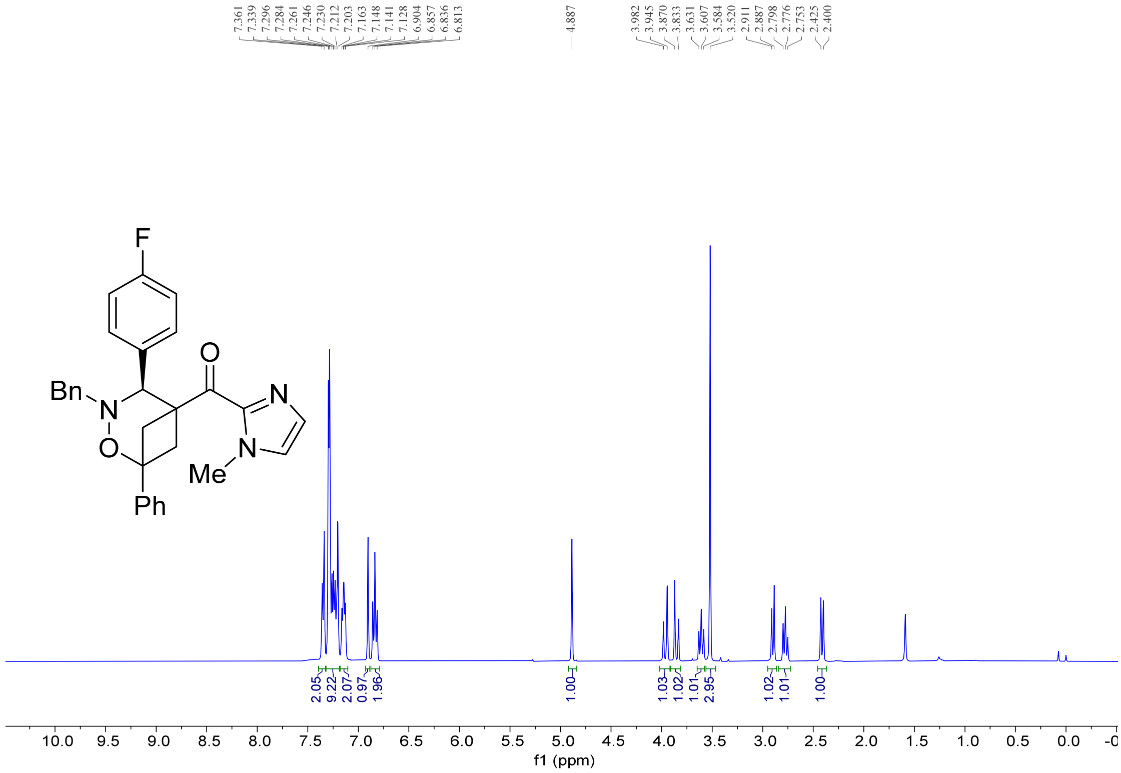

Supplementary Figure 71. ^{1}H NMR (400 MHz, $CDCl_3$) spectrum of compound **3aq**

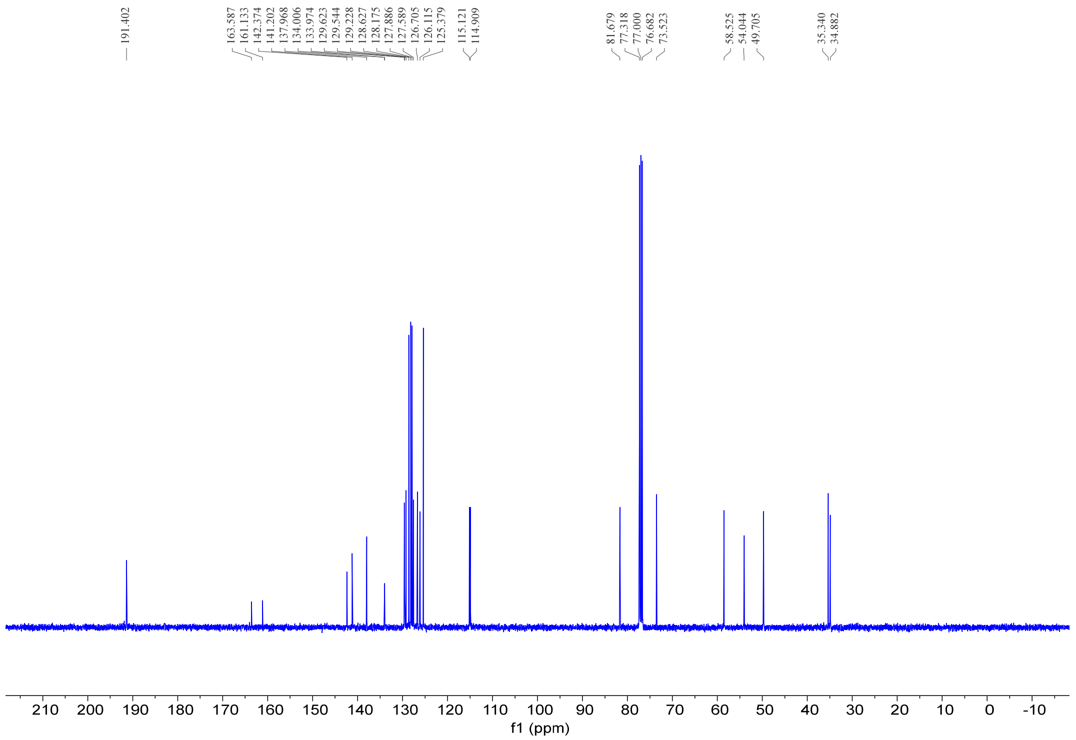

Supplementary Figure 72. ^{13}C NMR (100 MHz, $CDCl_3$) spectrum of compound **3aq**

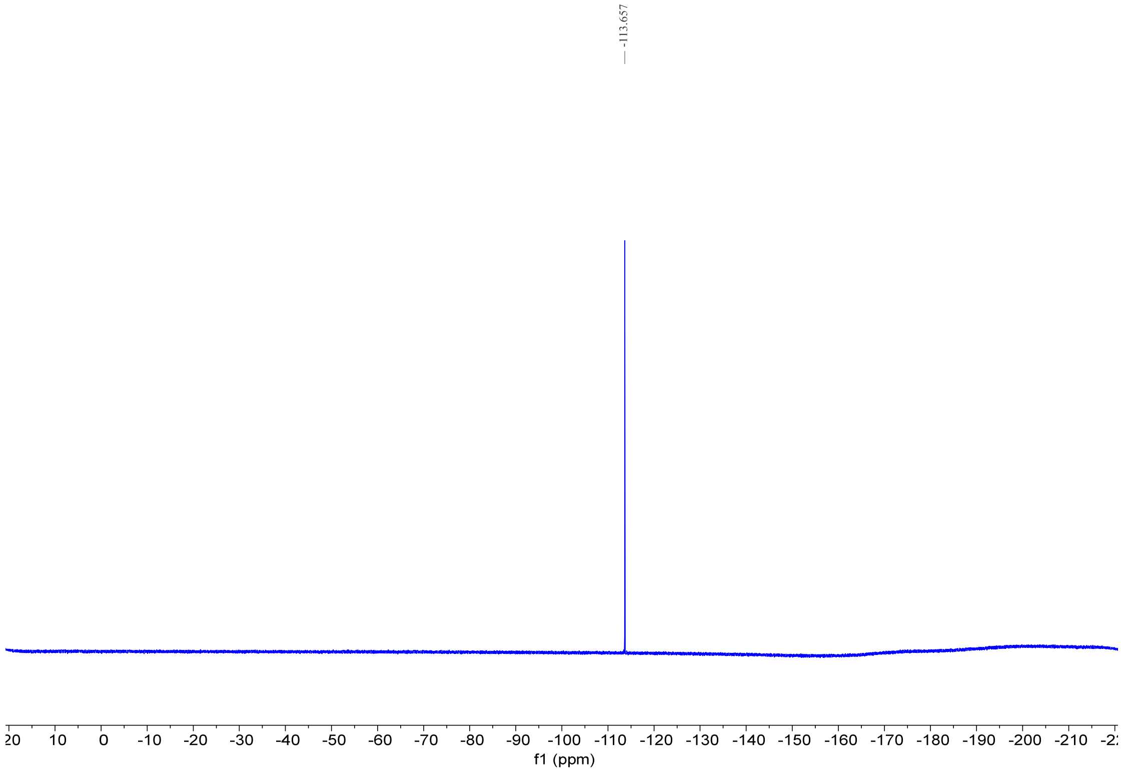

Supplementary Figure 73. ^{19}F NMR (376 MHz, $CDCl_3$) spectrum of compound **3aq**

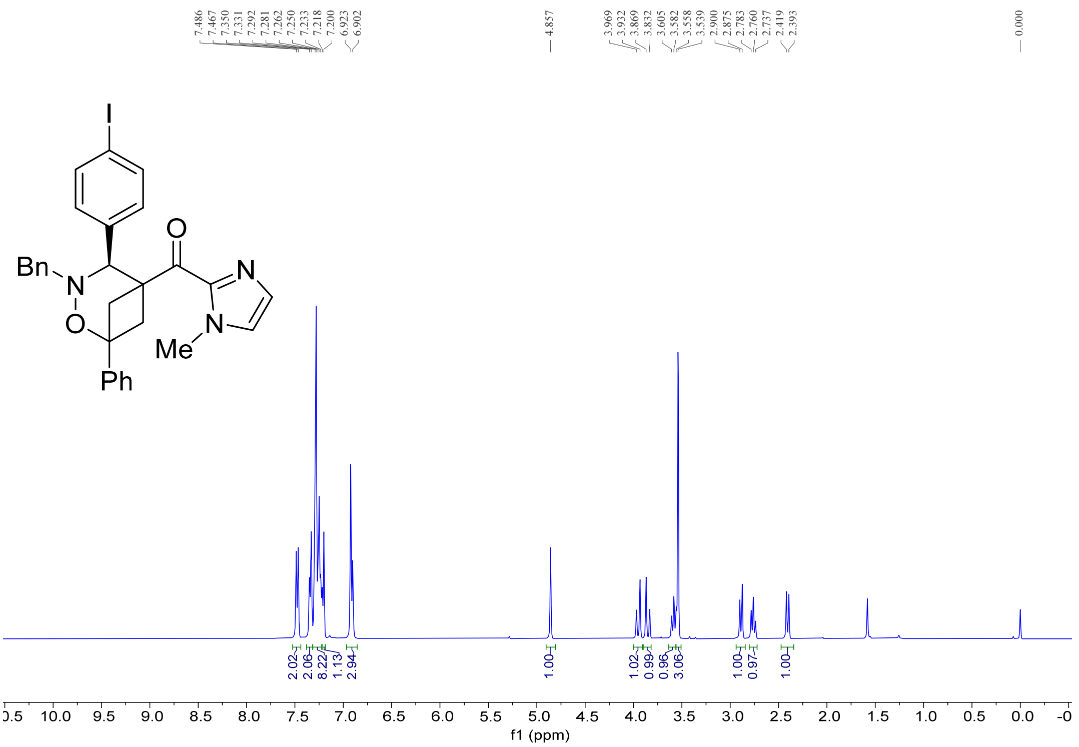

Supplementary Figure 74. ^{1}H NMR (400 MHz, $CDCl_3$) spectrum of compound **3ar**

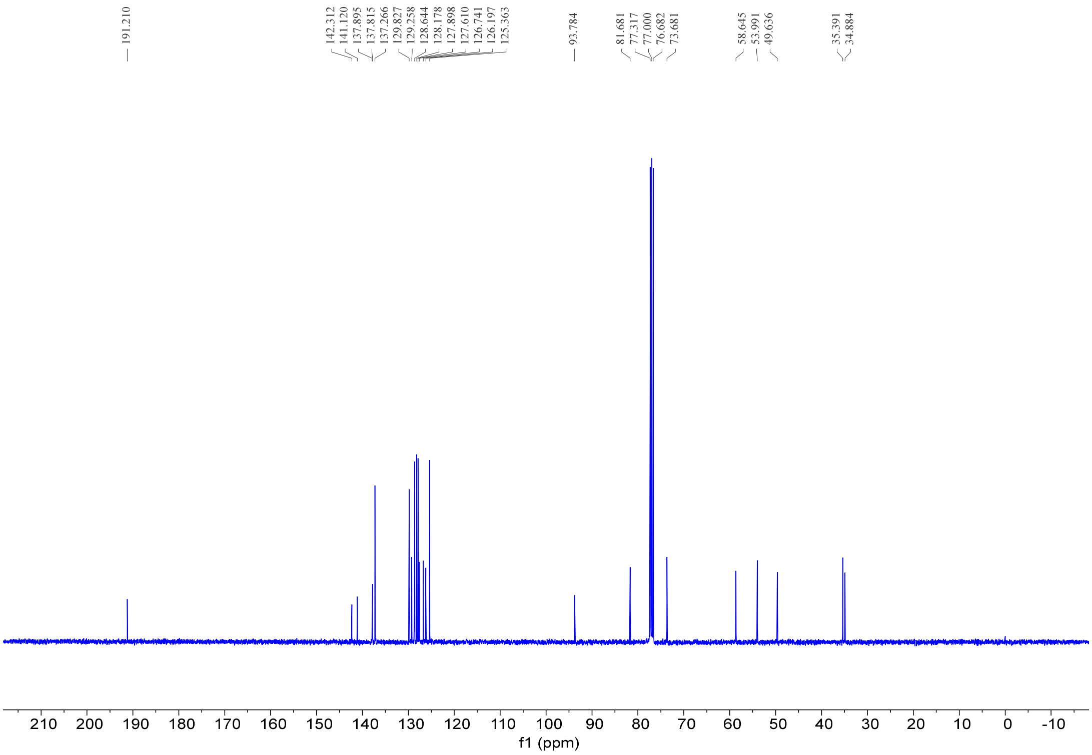

Supplementary Figure 75. ^{13}C NMR (100 MHz, $CDCl_3$) spectrum of compound **3ar**

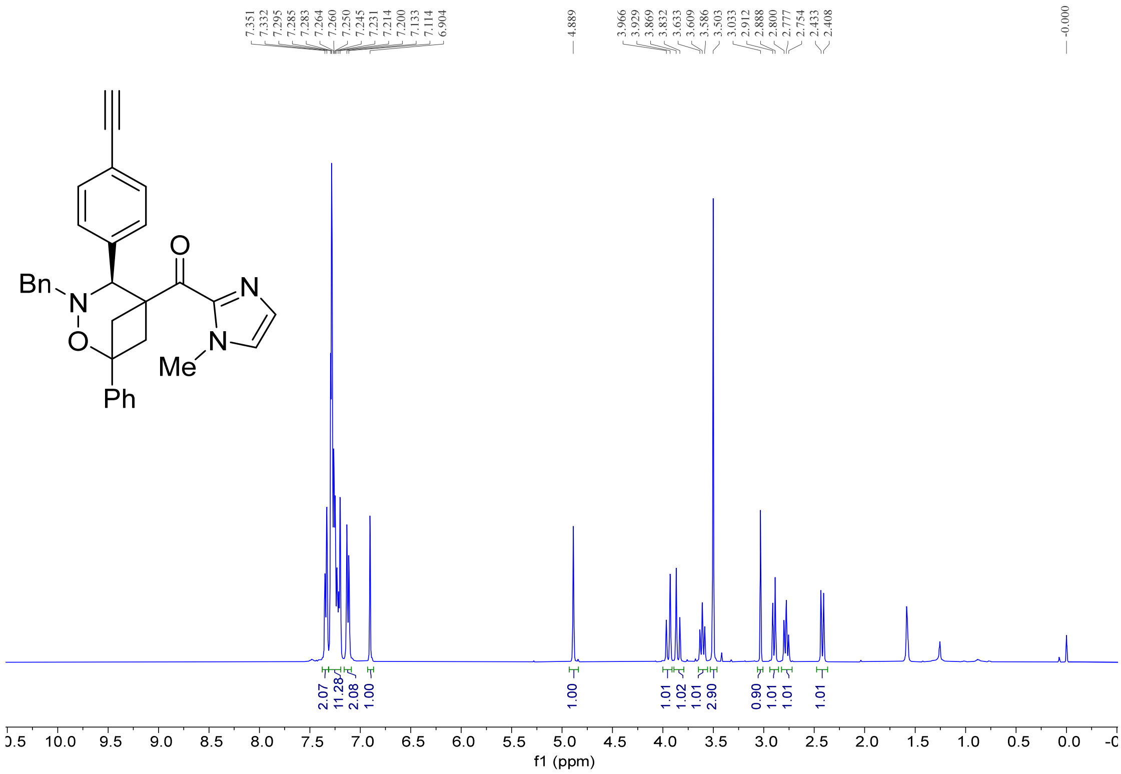

Supplementary Figure 76. ^{1}H NMR (400 MHz, $CDCl_3$) spectrum of compound **3as**

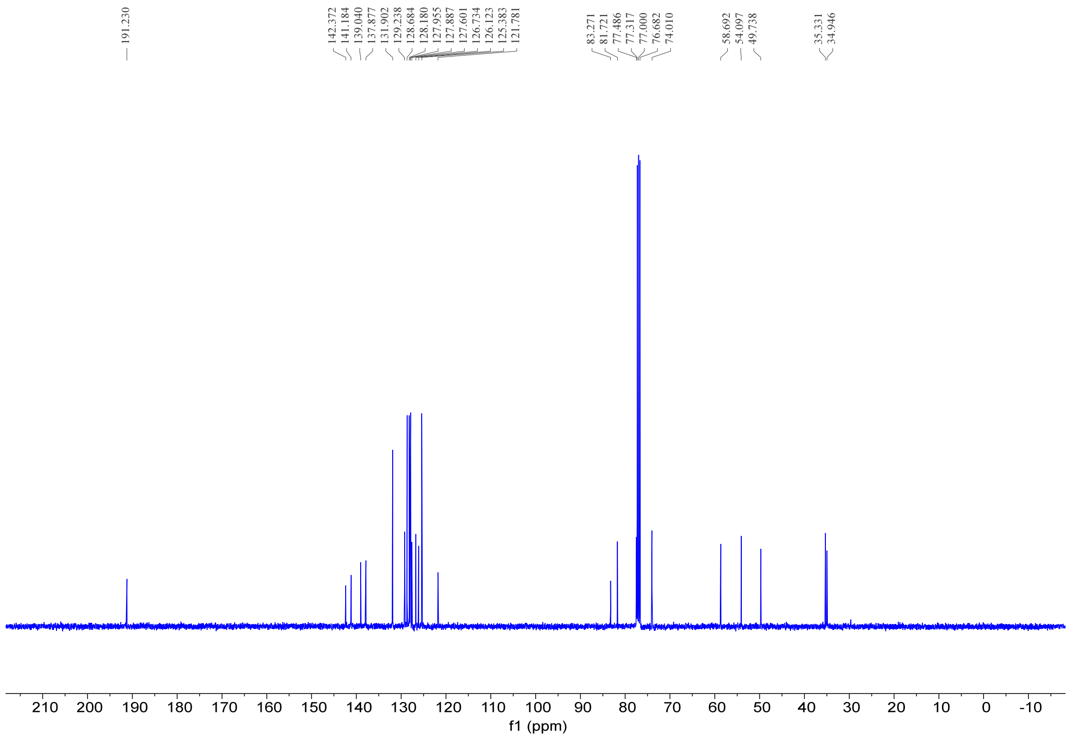

Supplementary Figure 77. ^{13}C NMR (100 MHz, $CDCl_3$) spectrum of compound **3as**

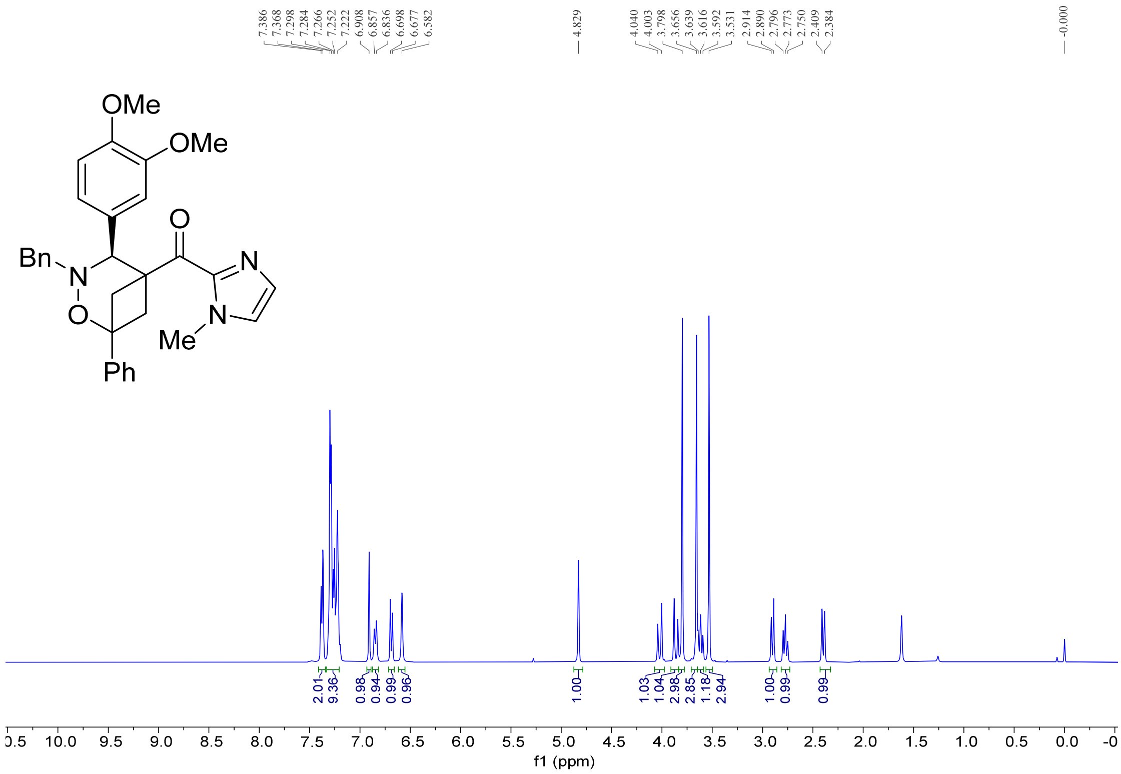

Supplementary Figure 78. ^{1}H NMR (400 MHz, $CDCl_3$) spectrum of compound **3at**

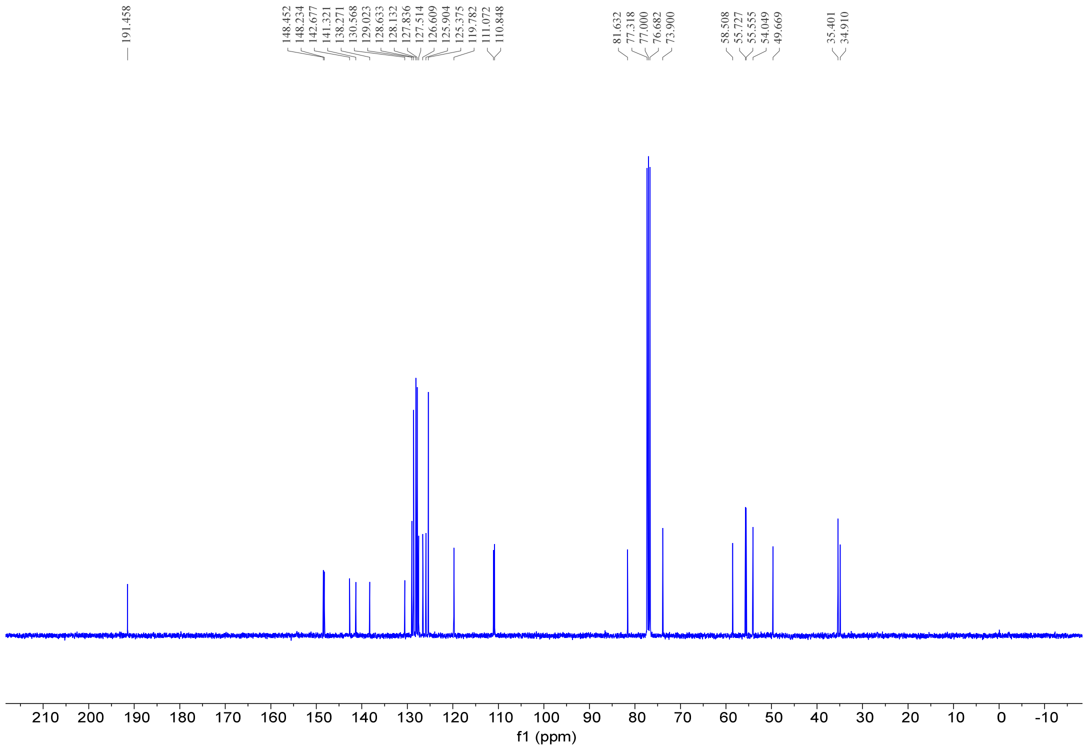

Supplementary Figure 79. ^{13}C NMR (100 MHz, $CDCl_3$) spectrum of compound **3at**

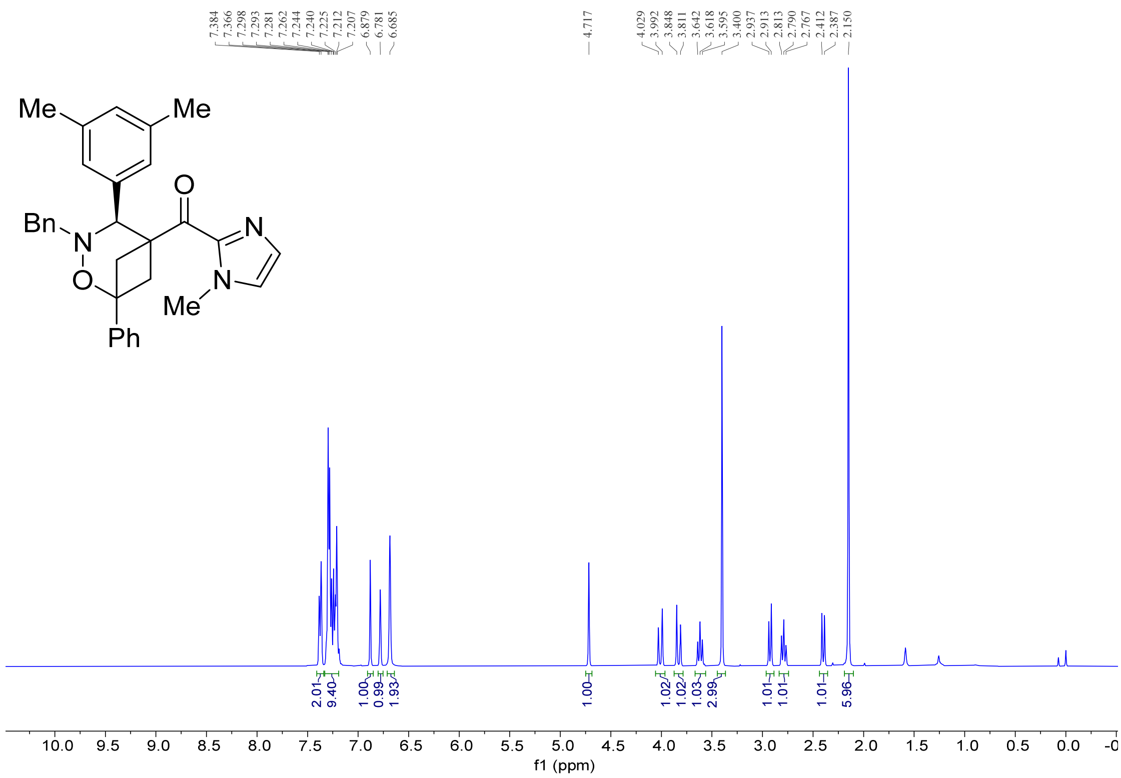

Supplementary Figure 80. ^{1}H NMR (400 MHz, $CDCl_3$) spectrum of compound **3au**

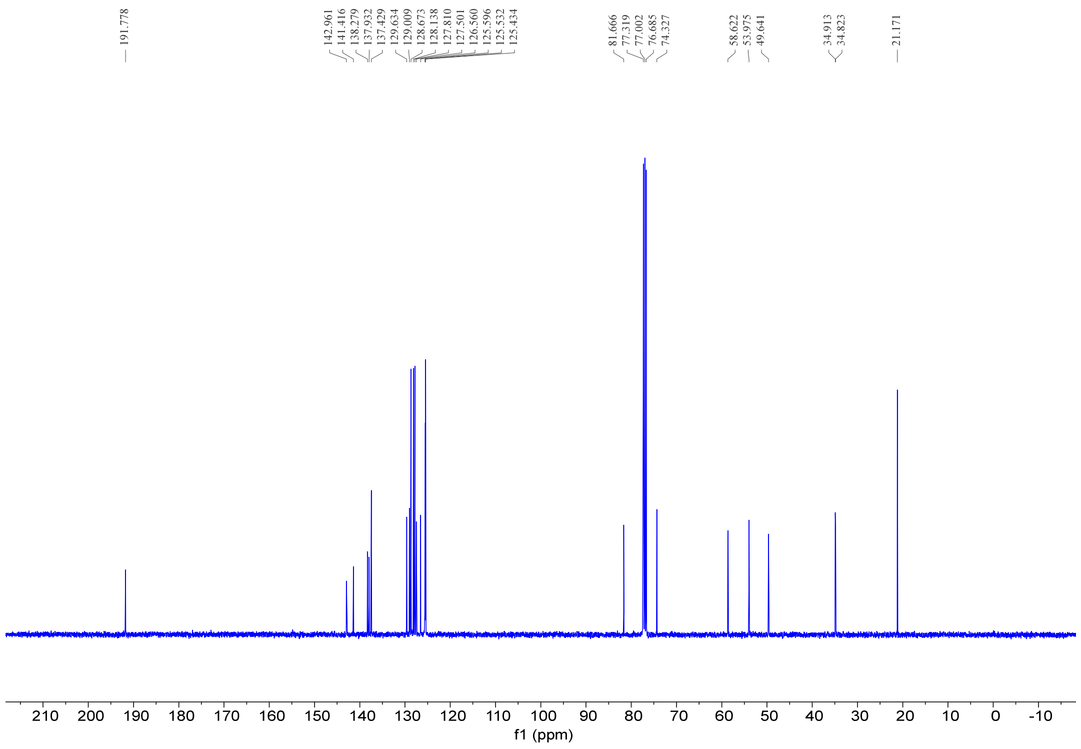

Supplementary Figure 81. ^{13}C NMR (100 MHz, $CDCl_3$) spectrum of compound **3au**

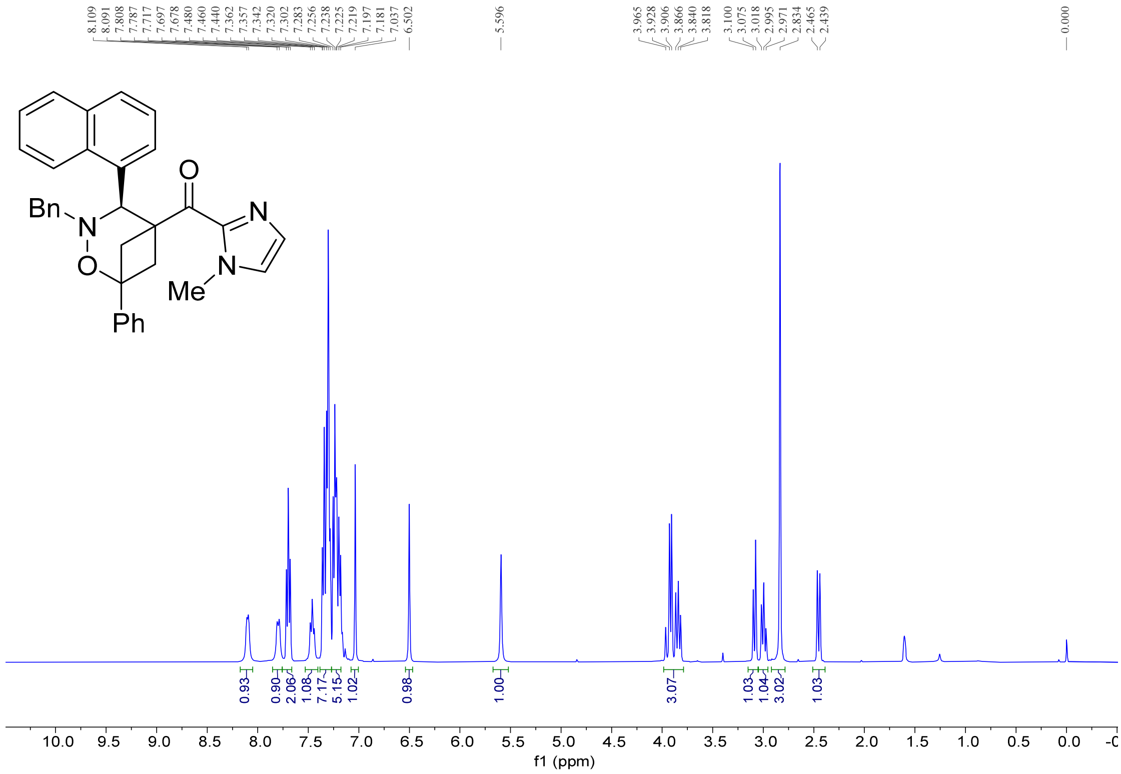

Supplementary Figure 82. ^{1}H NMR (400 MHz, $CDCl_3$) spectrum of compound **3av**

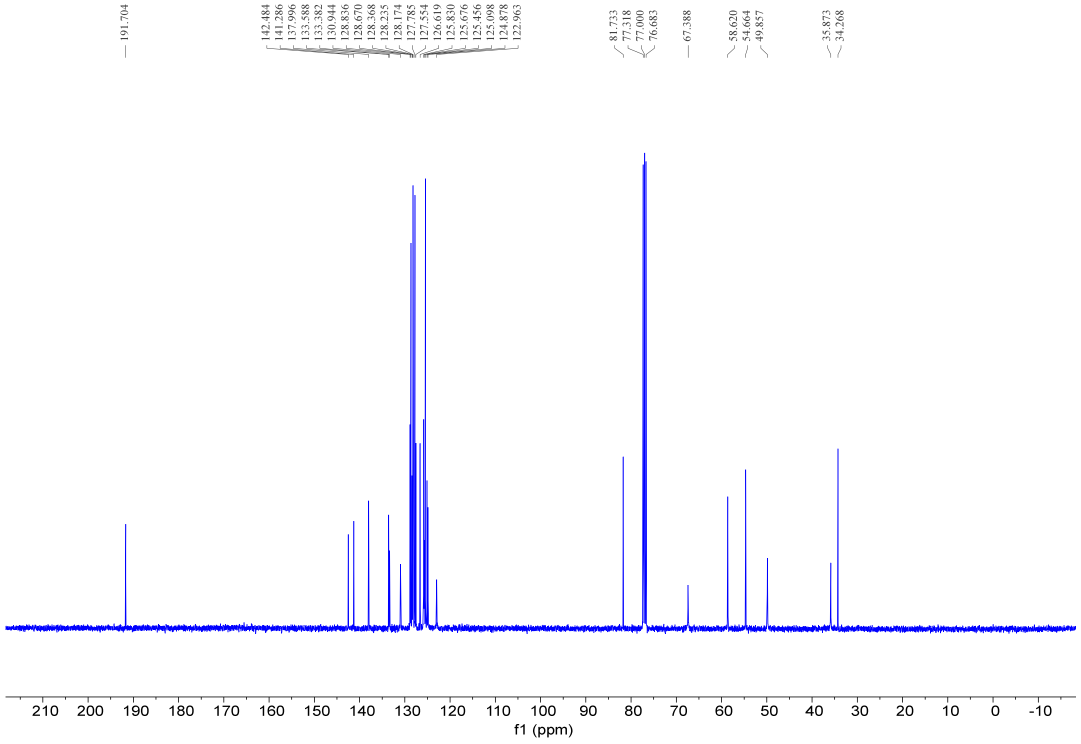

Supplementary Figure 83. ^{13}C NMR (100 MHz, $CDCl_3$) spectrum of compound **3av**

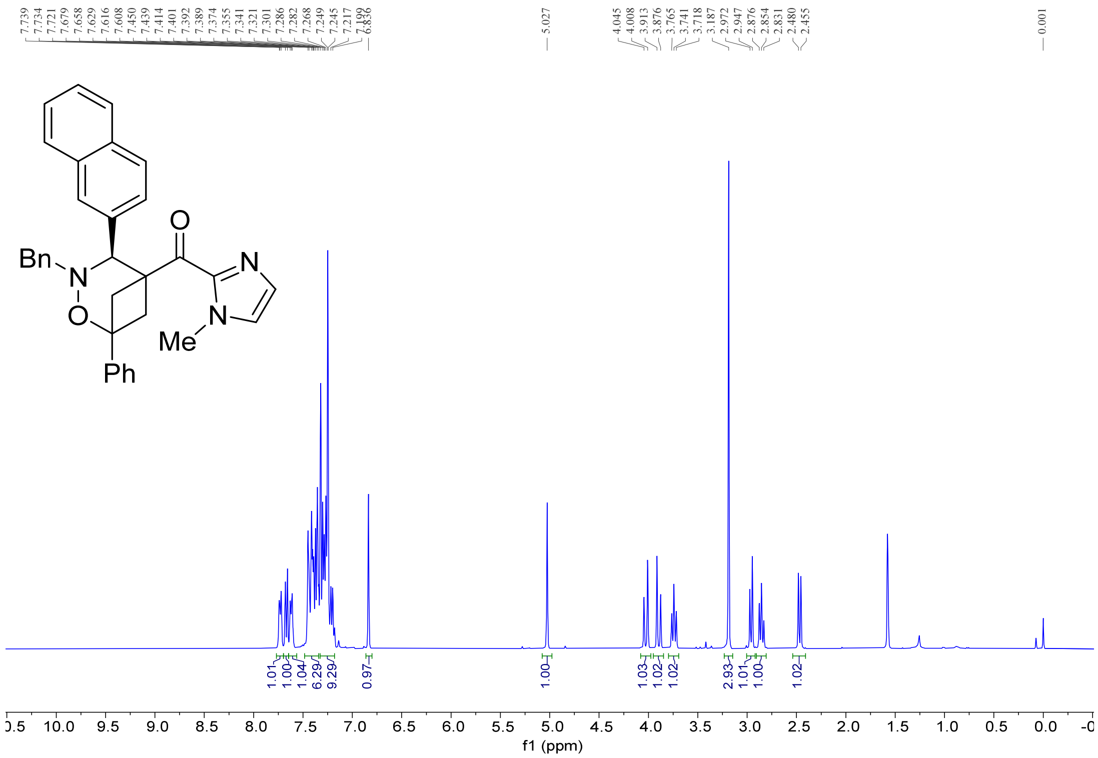

Supplementary Figure 84. ^{1}H NMR (400 MHz, $CDCl_3$) spectrum of compound **3aw**

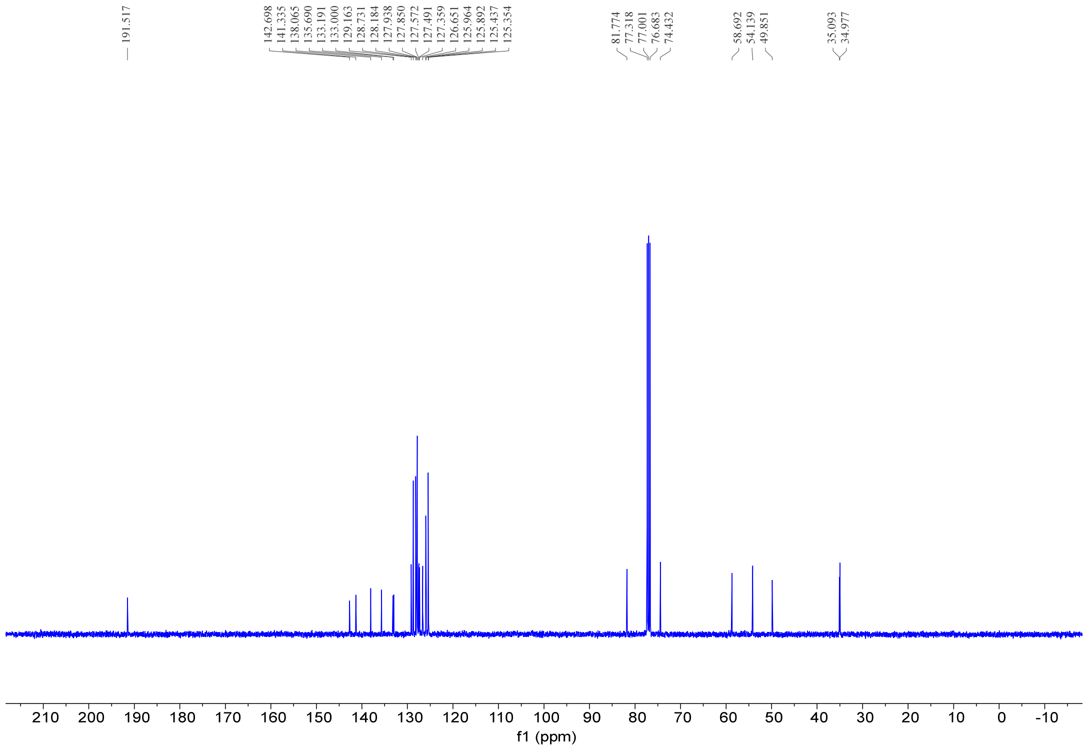

Supplementary Figure 85. ^{13}C NMR (100 MHz, $CDCl_3$) spectrum of compound **3aw**

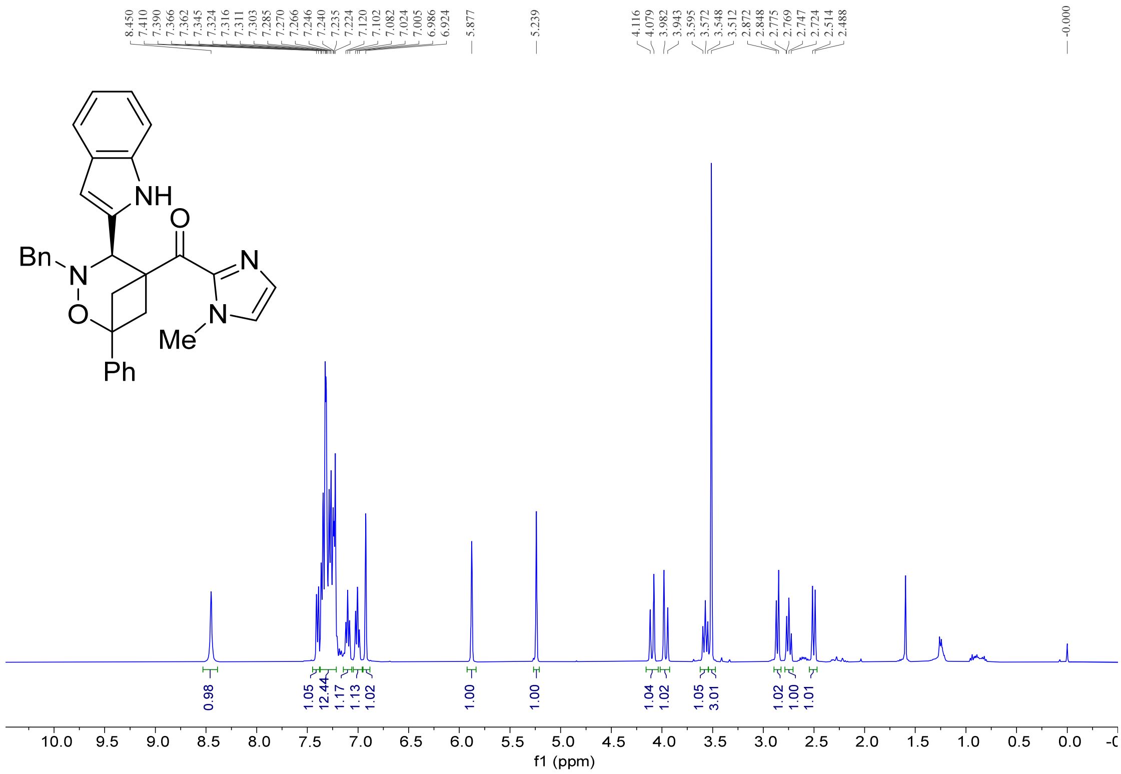

Supplementary Figure 86. ^{1}H NMR (400 MHz, $CDCl_3$) spectrum of compound **3ax**

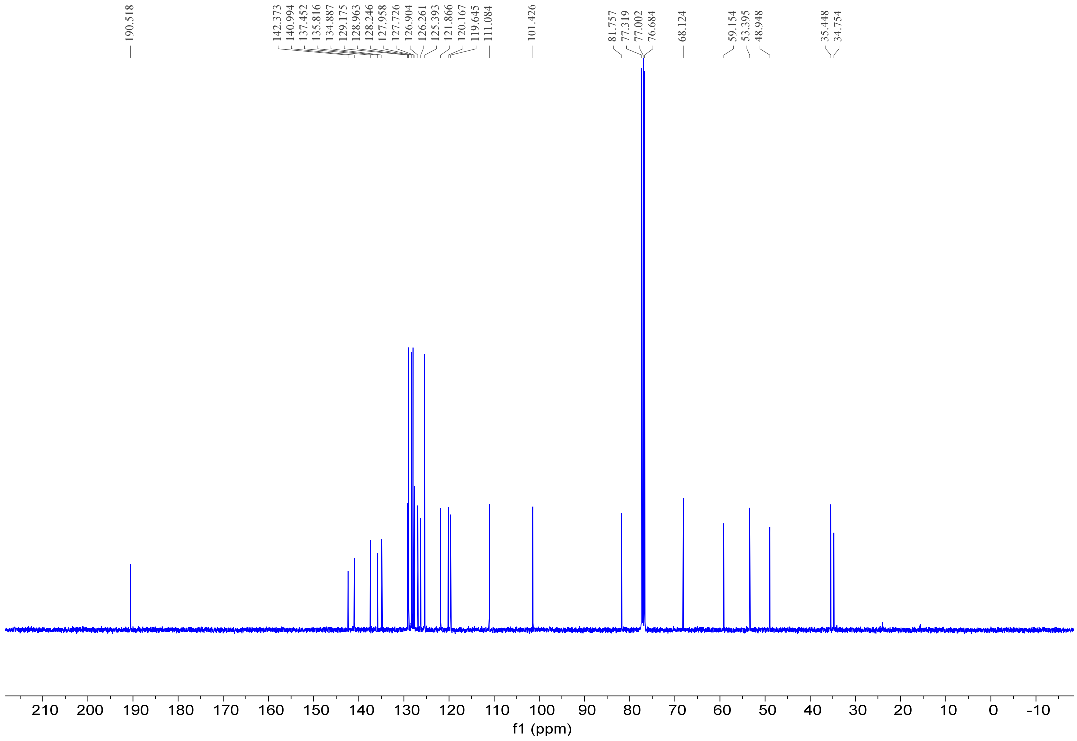

Supplementary Figure 87. ^{13}C NMR (100 MHz, $CDCl_3$) spectrum of compound **3ax**

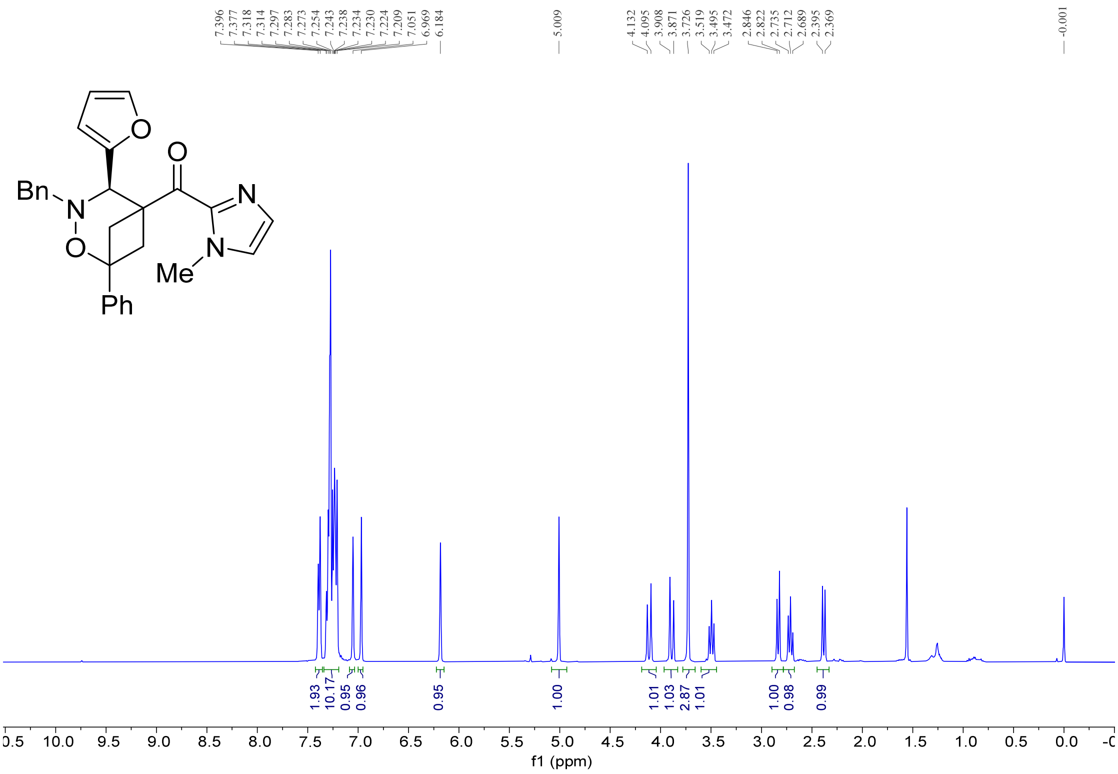

Supplementary Figure 88. ^{1}H NMR (400 MHz, $CDCl_3$) spectrum of compound **3ay**

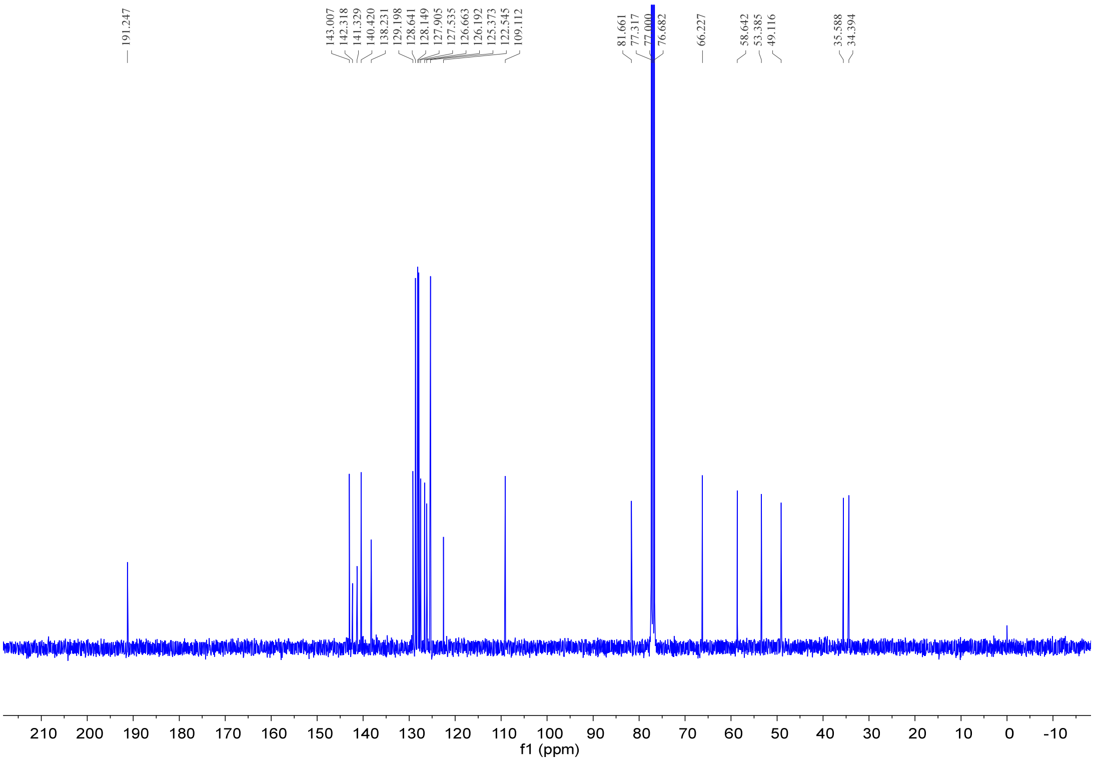

Supplementary Figure 89. ^{13}C NMR (100 MHz, $CDCl_3$) spectrum of compound **3ay**

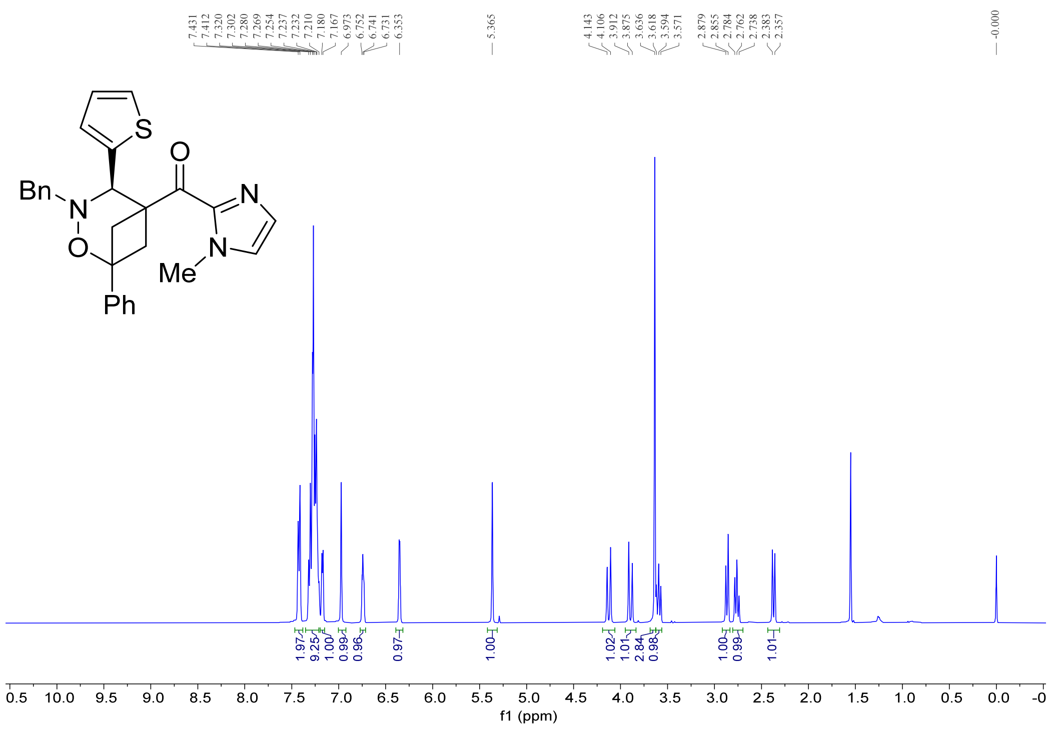

Supplementary Figure 90. ^{1}H NMR (400 MHz, $CDCl_3$) spectrum of compound **3az**

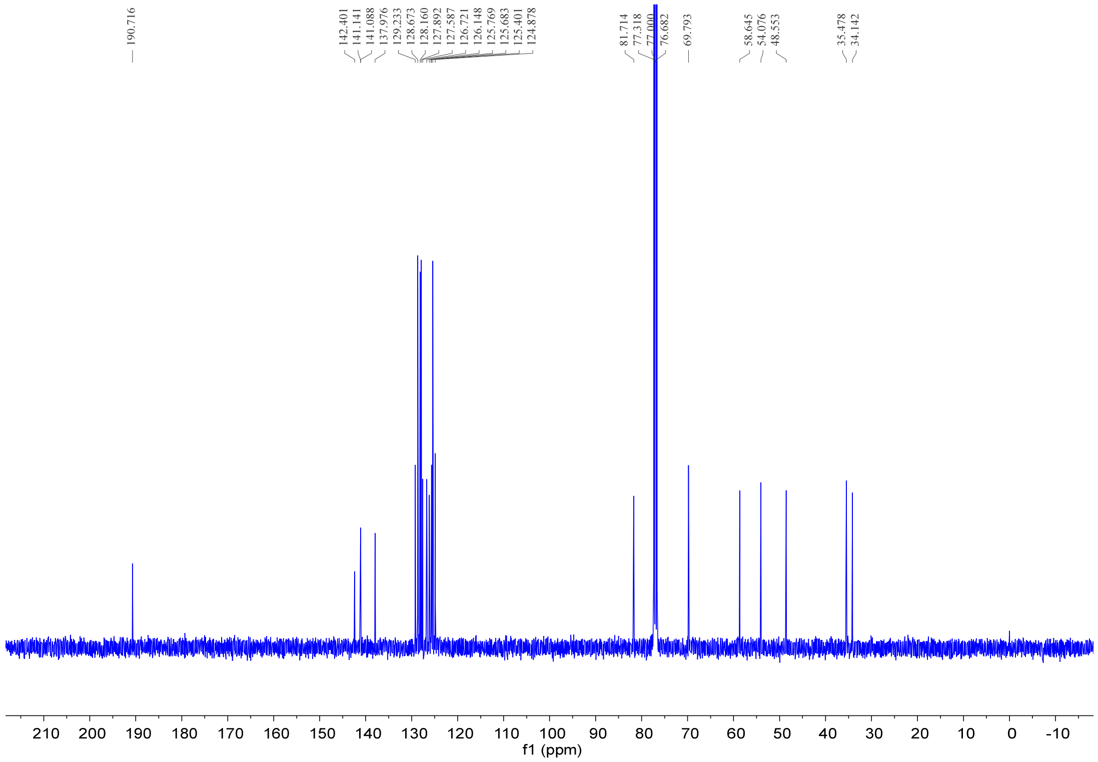

Supplementary Figure 91. ^{13}C NMR (100 MHz, $CDCl_3$) spectrum of compound **3az**

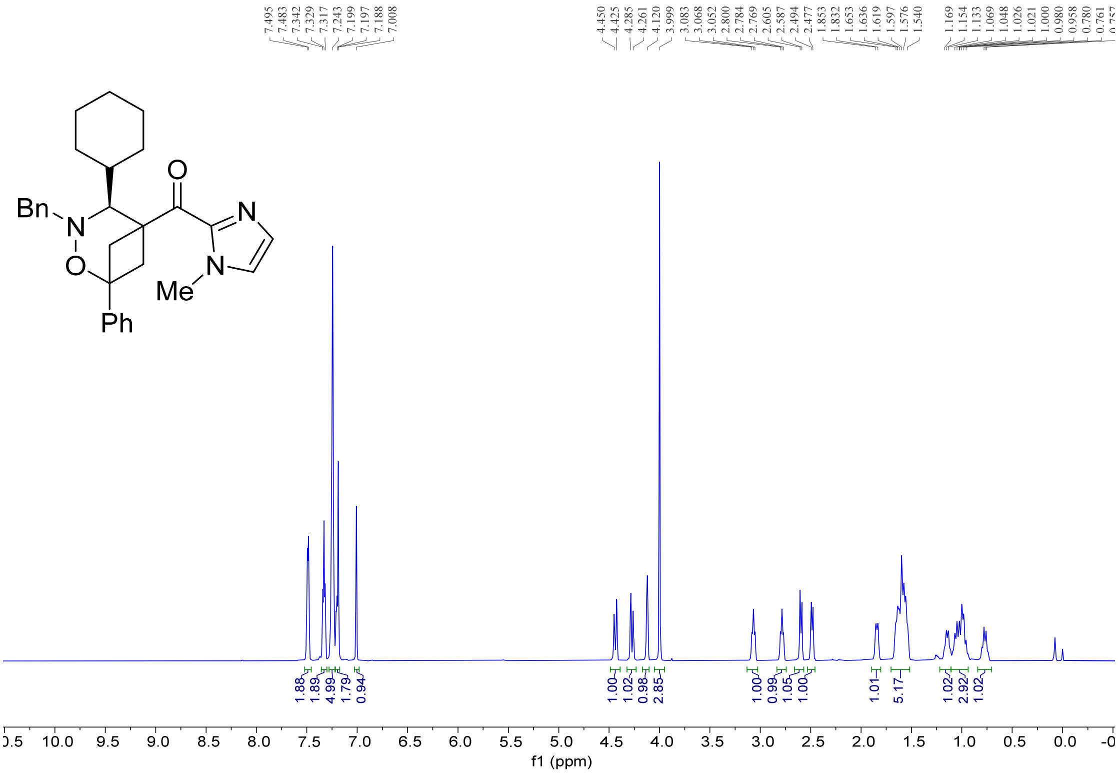

Supplementary Figure 92. ^{1}H NMR (600 MHz, $CDCl_3$) spectrum of compound **3aaa**

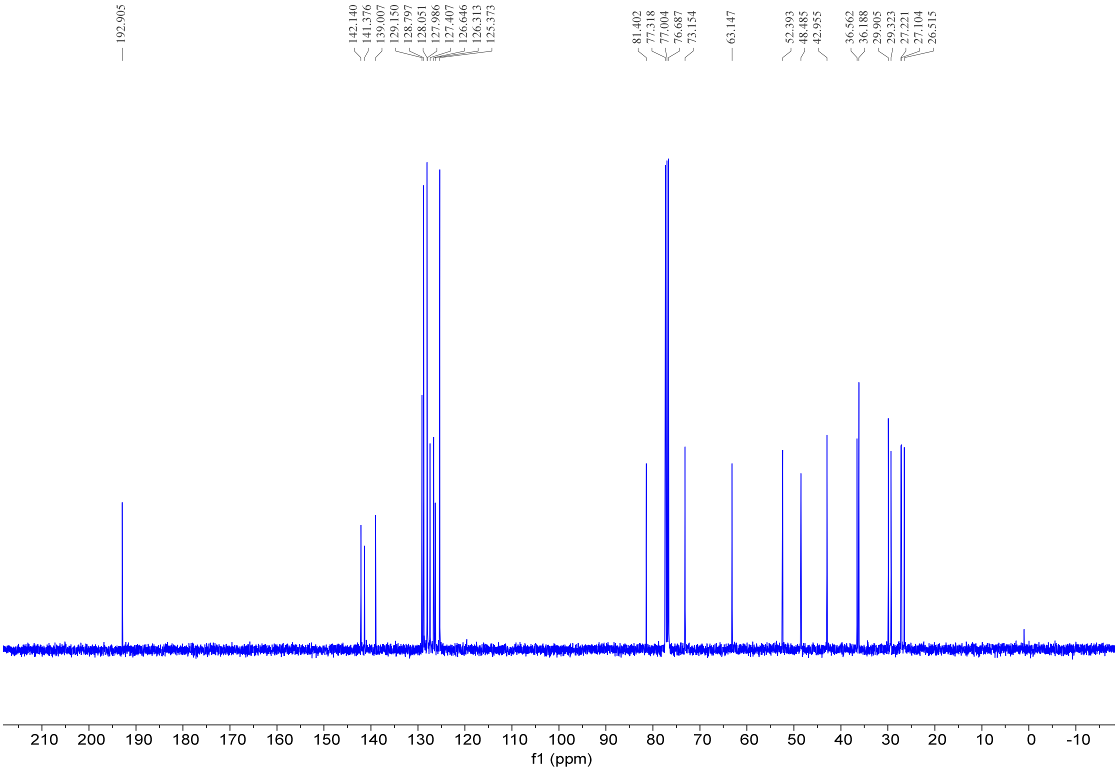

Supplementary Figure 93. ^{13}C NMR (100 MHz, $CDCl_3$) spectrum of compound **3aaa**

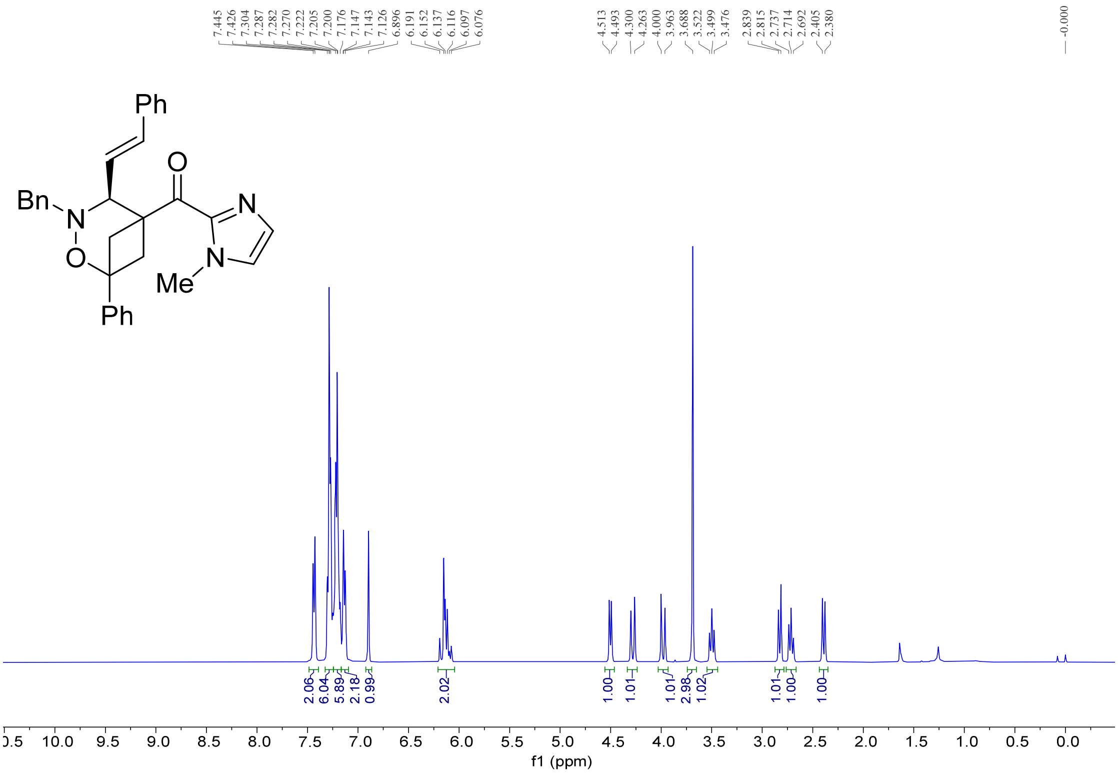

Supplementary Figure 94. ^{1}H NMR (400 MHz, $CDCl_3$) spectrum of compound **3abb**

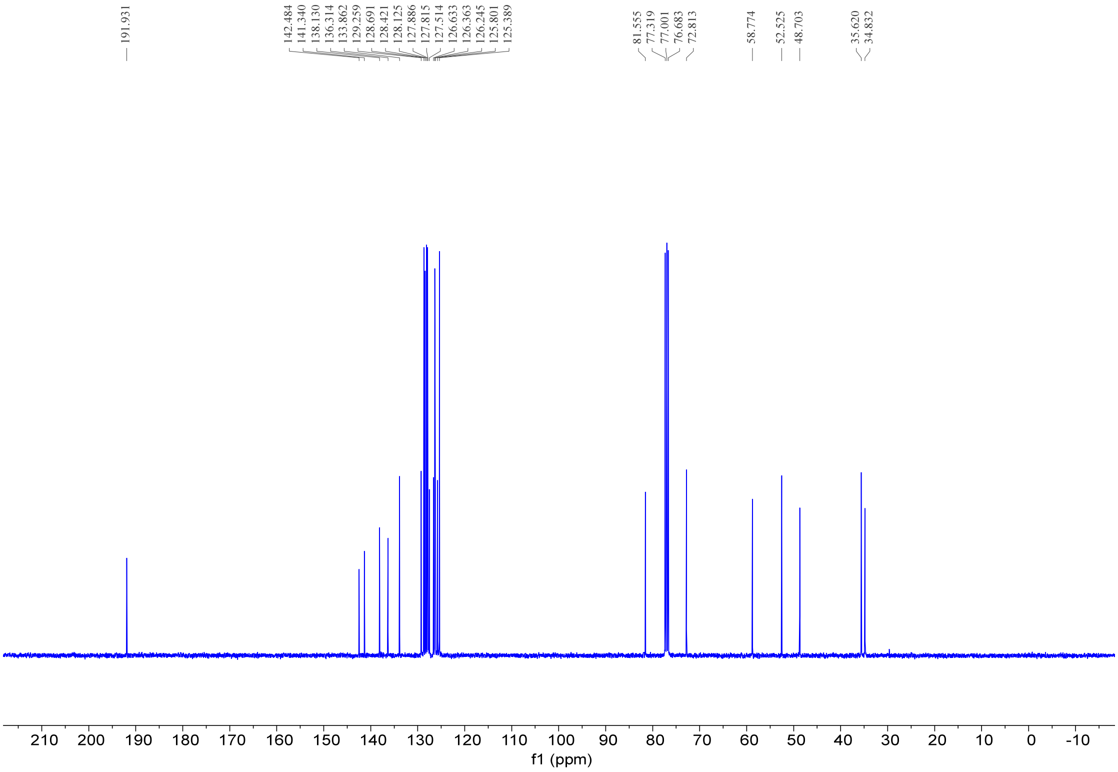

Supplementary Figure 95. ^{13}C NMR (100 MHz, $CDCl_3$) spectrum of compound **3abb**

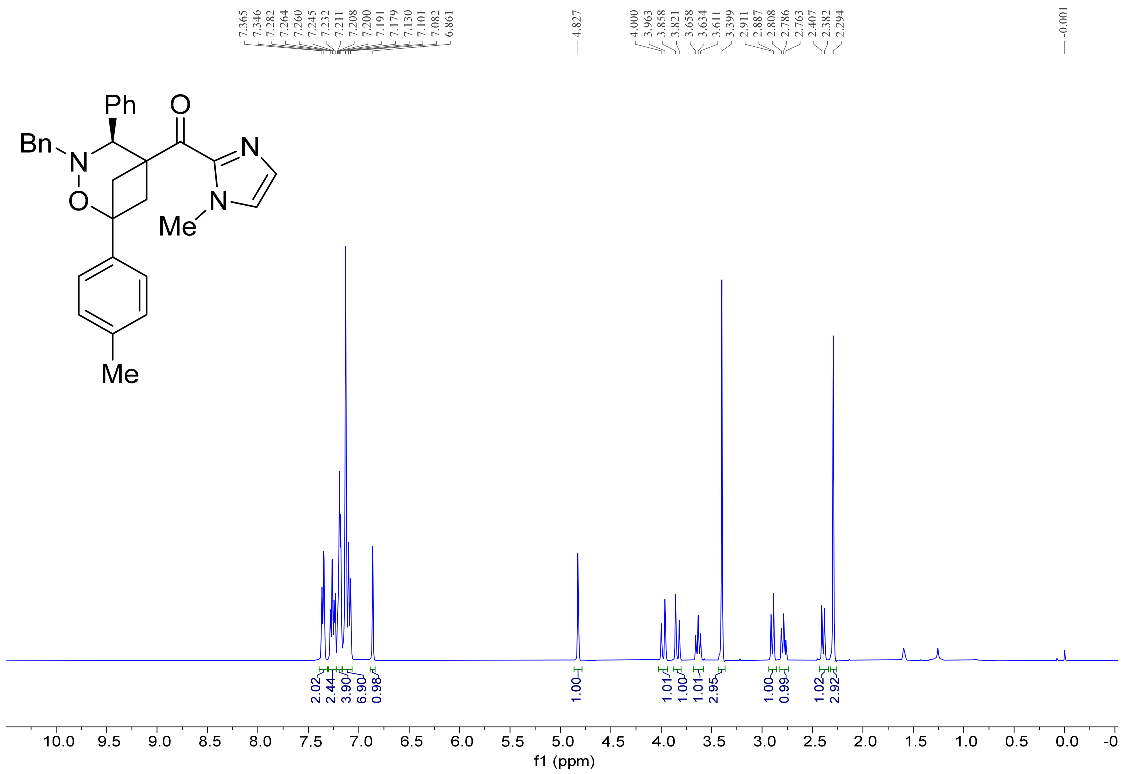

Supplementary Figure 96. ^{1}H NMR (400 MHz, $CDCl_3$) spectrum of compound **3bb**

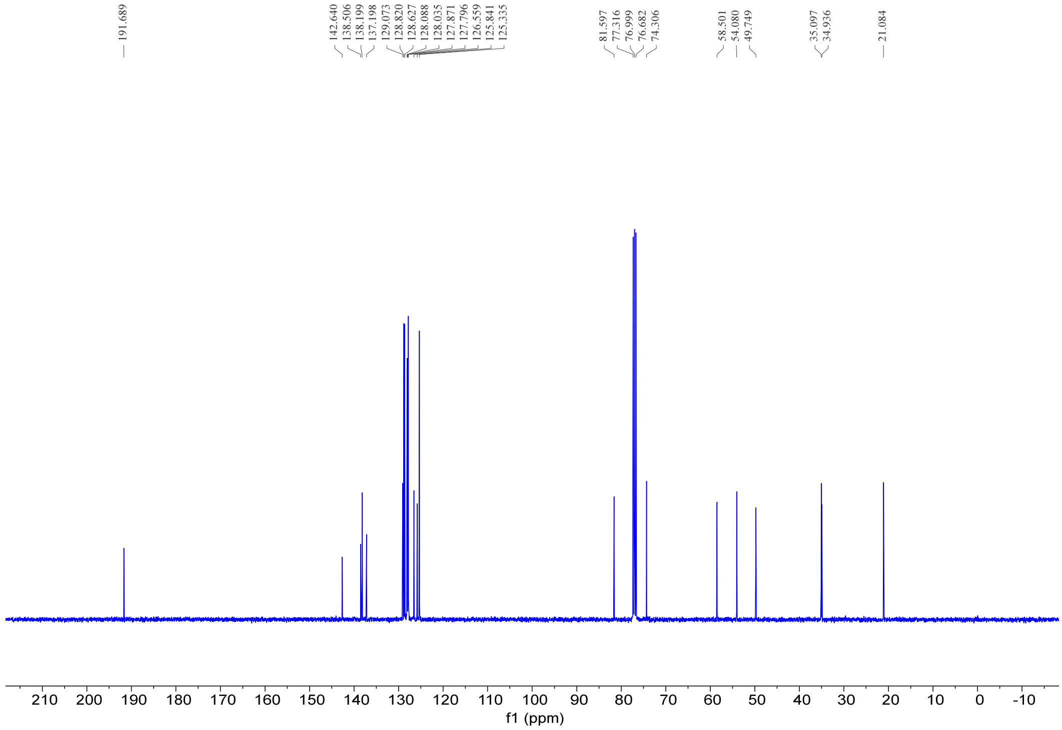

Supplementary Figure 97. ^{13}C NMR (100 MHz, $CDCl_3$) spectrum of compound **3bb**

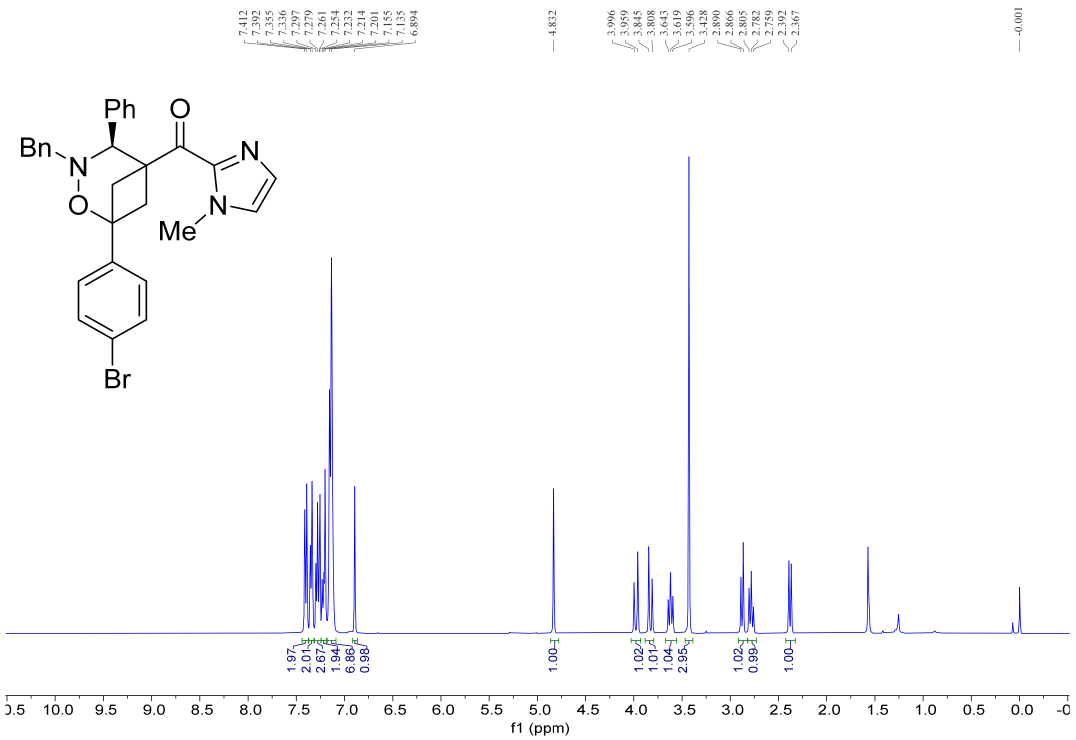

Supplementary Figure 98. ^{1}H NMR (400 MHz, $CDCl_3$) spectrum of compound **3cb**

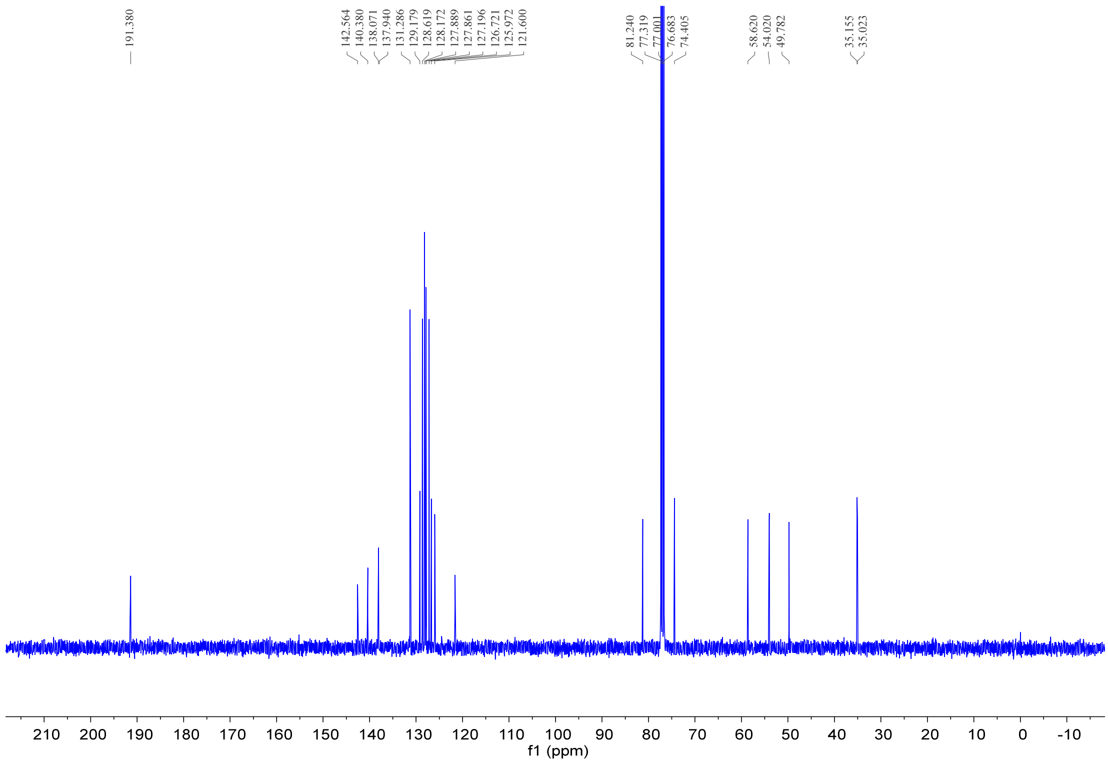

Supplementary Figure 99. ^{13}C NMR (100 MHz, $CDCl_3$) spectrum of compound **3cb**

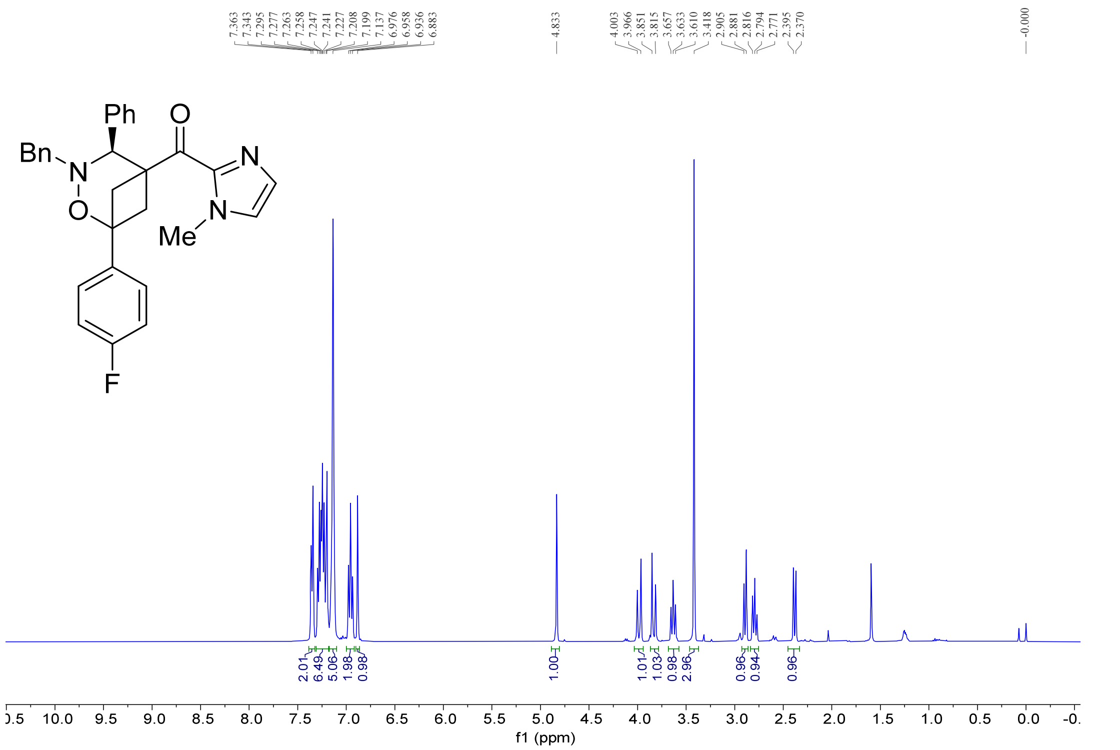

Supplementary Figure 100. ^{1}H NMR (400 MHz, $CDCl_3$) spectrum of compound **3db**

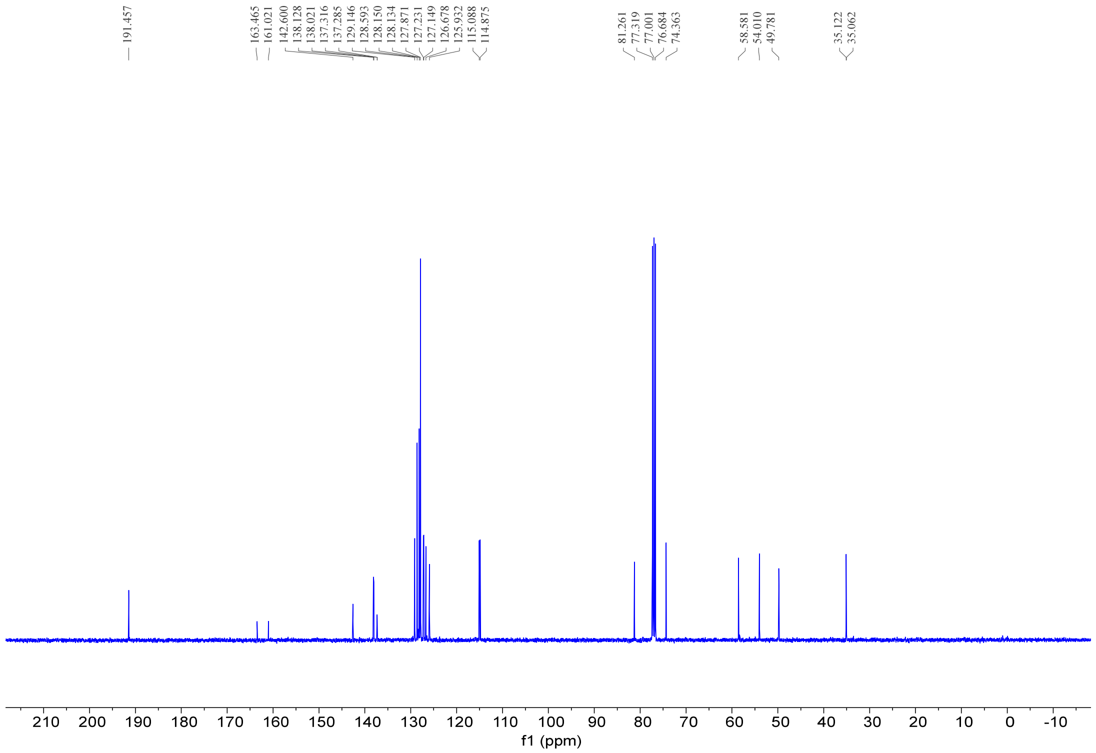

Supplementary Figure 101. ^{13}C NMR (100 MHz, $CDCl_3$) spectrum of compound **3db**

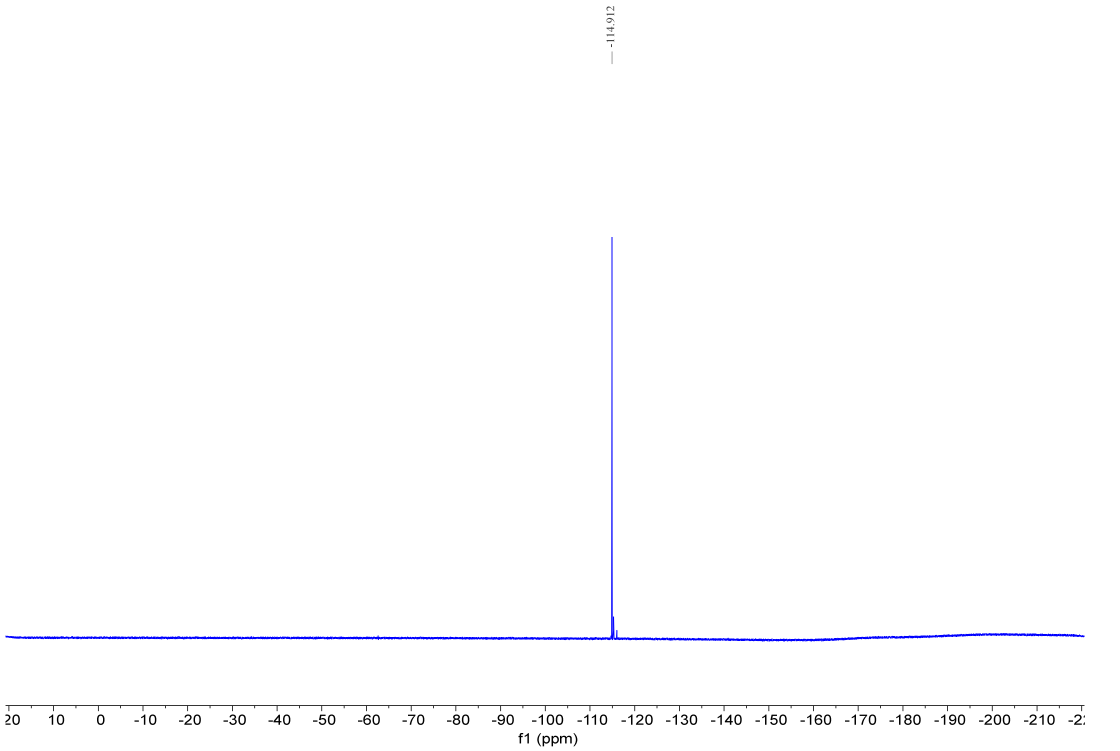

Supplementary Figure 102. ^{19}F NMR (376 MHz, $CDCl_3$) spectrum of compound **3db**

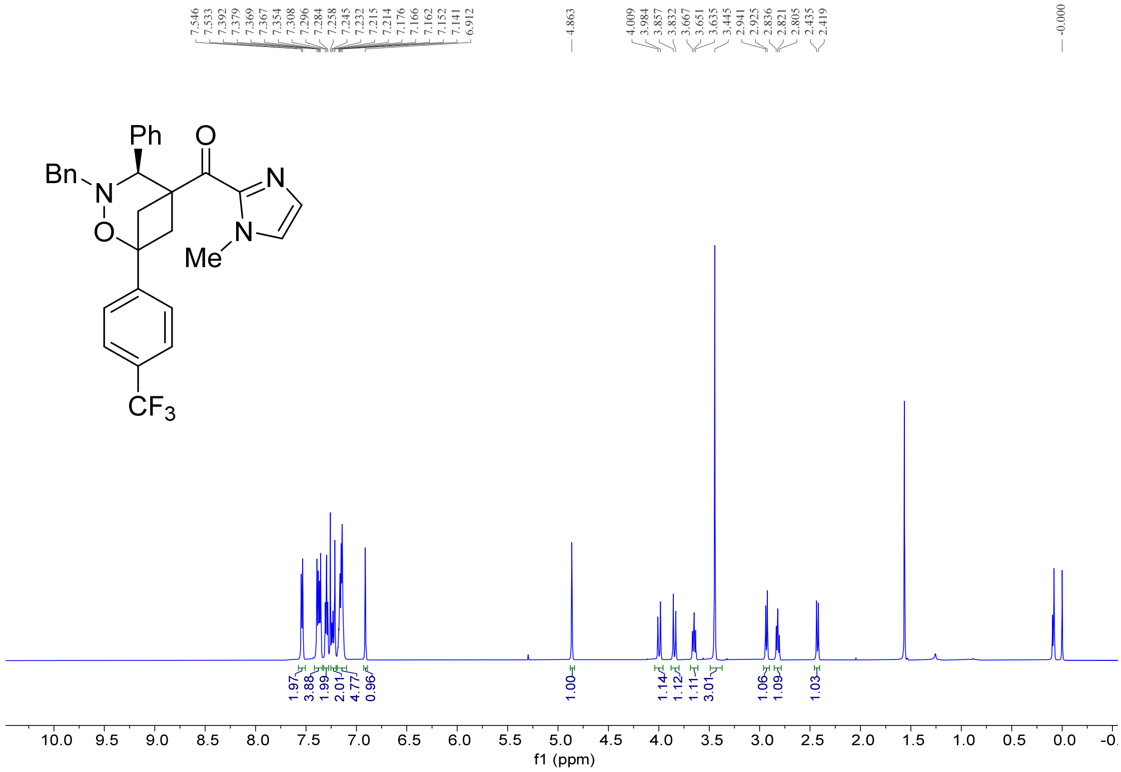

Supplementary Figure 103. ^{1}H NMR (600 MHz, $CDCl_3$) spectrum of compound **3eb**

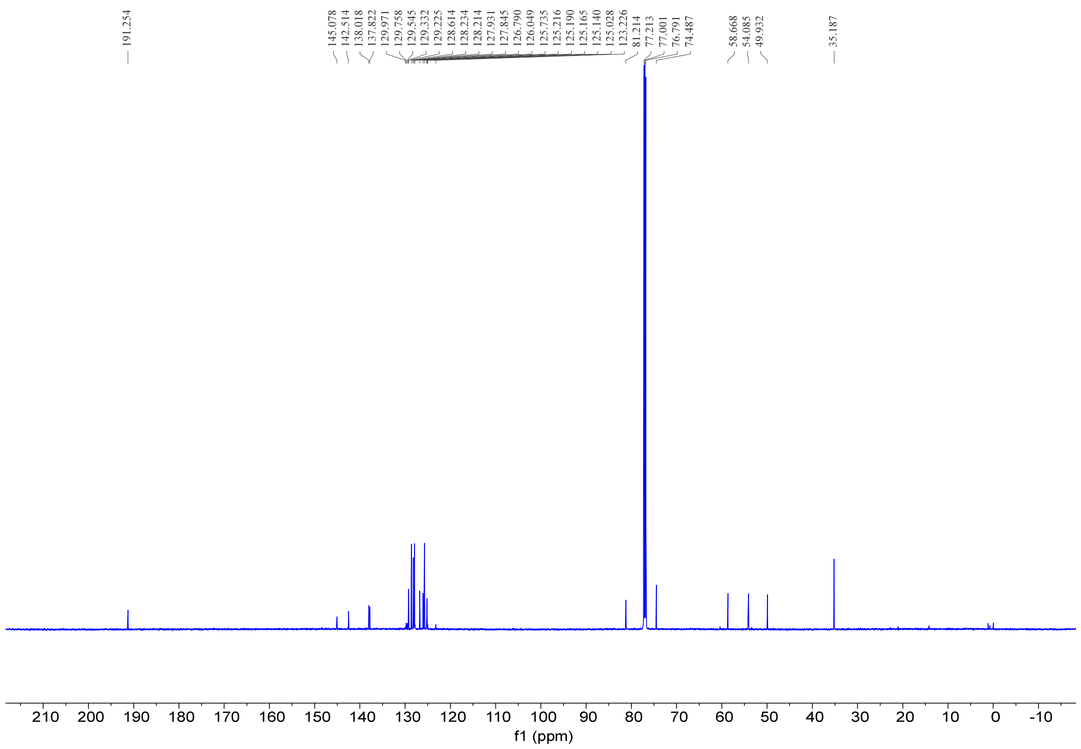

Supplementary Figure 104. ^{13}C NMR (100 MHz, $CDCl_3$) spectrum of compound **3eb**

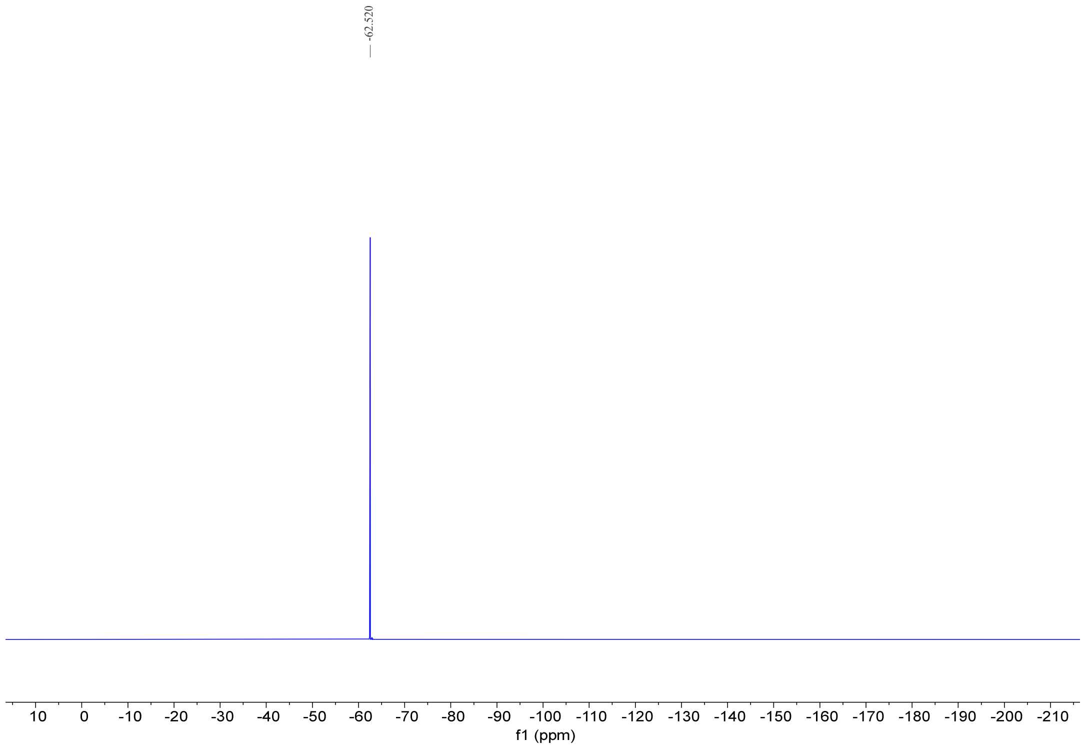

Supplementary Figure 105. ^{19}F NMR (565 MHz, $CDCl_3$) spectrum of compound **3eb**

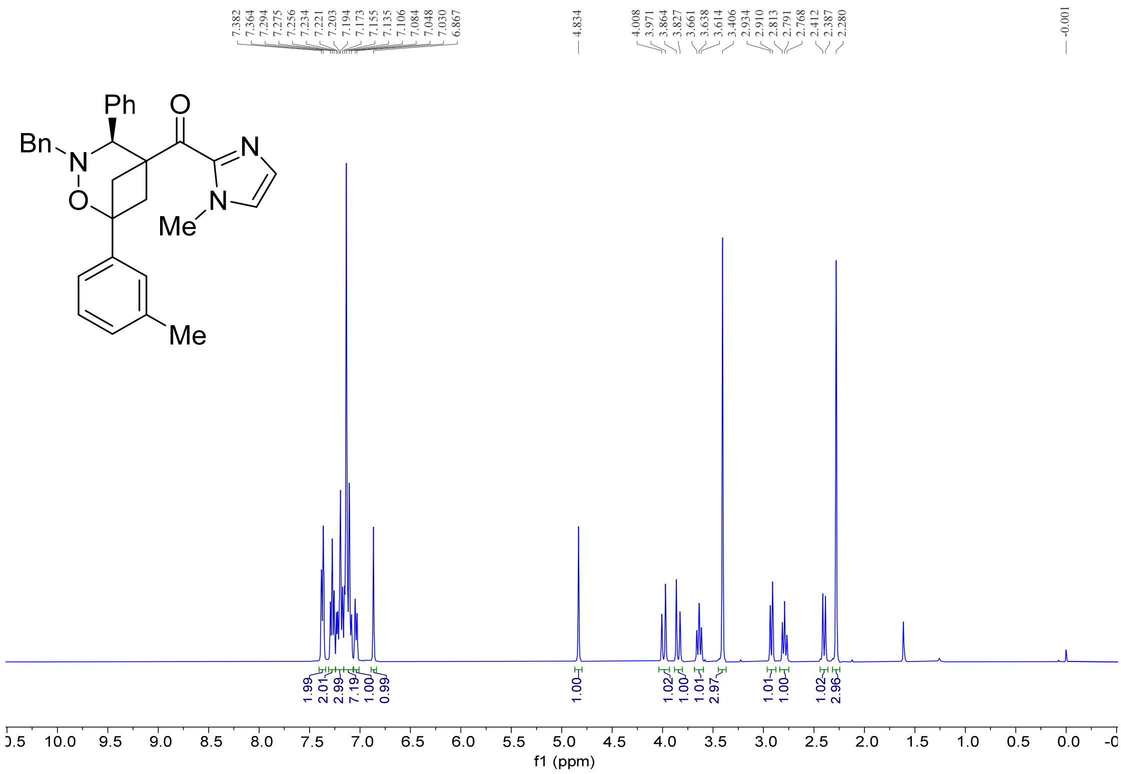

Supplementary Figure 106. ^{1}H NMR (400 MHz, $CDCl_3$) spectrum of compound **3fb**

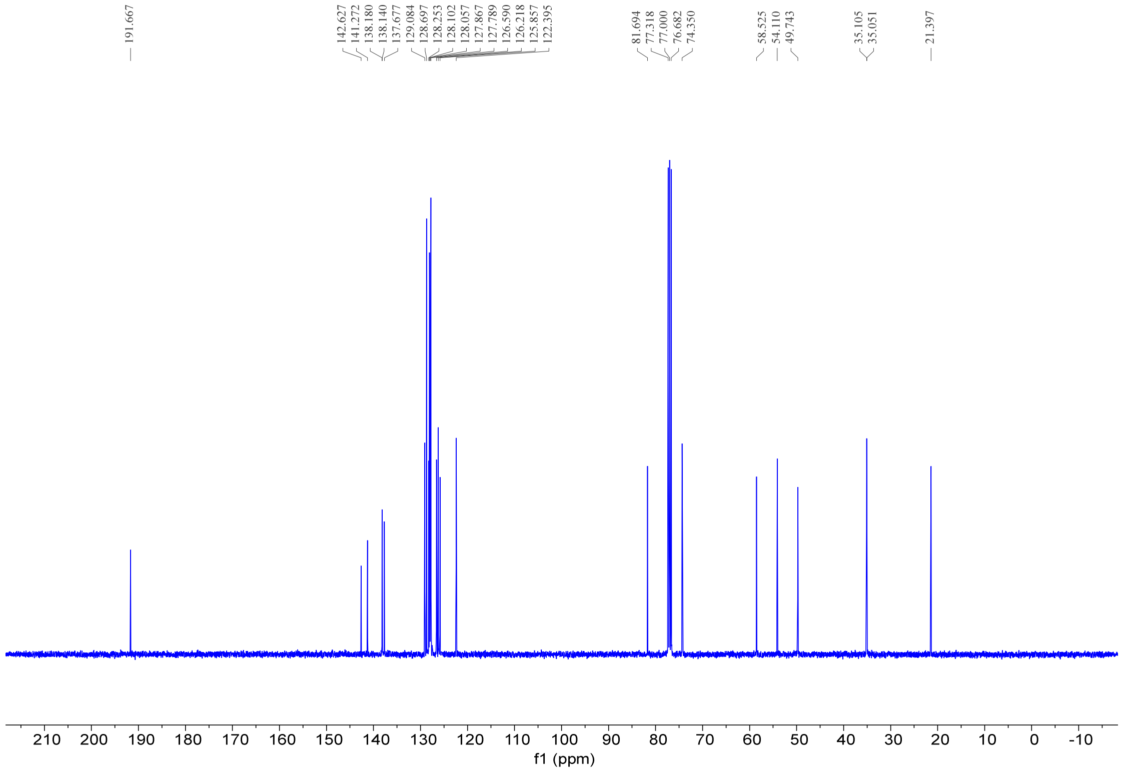

Supplementary Figure 107. ^{13}C NMR (100 MHz, $CDCl_3$) spectrum of compound **3fb**

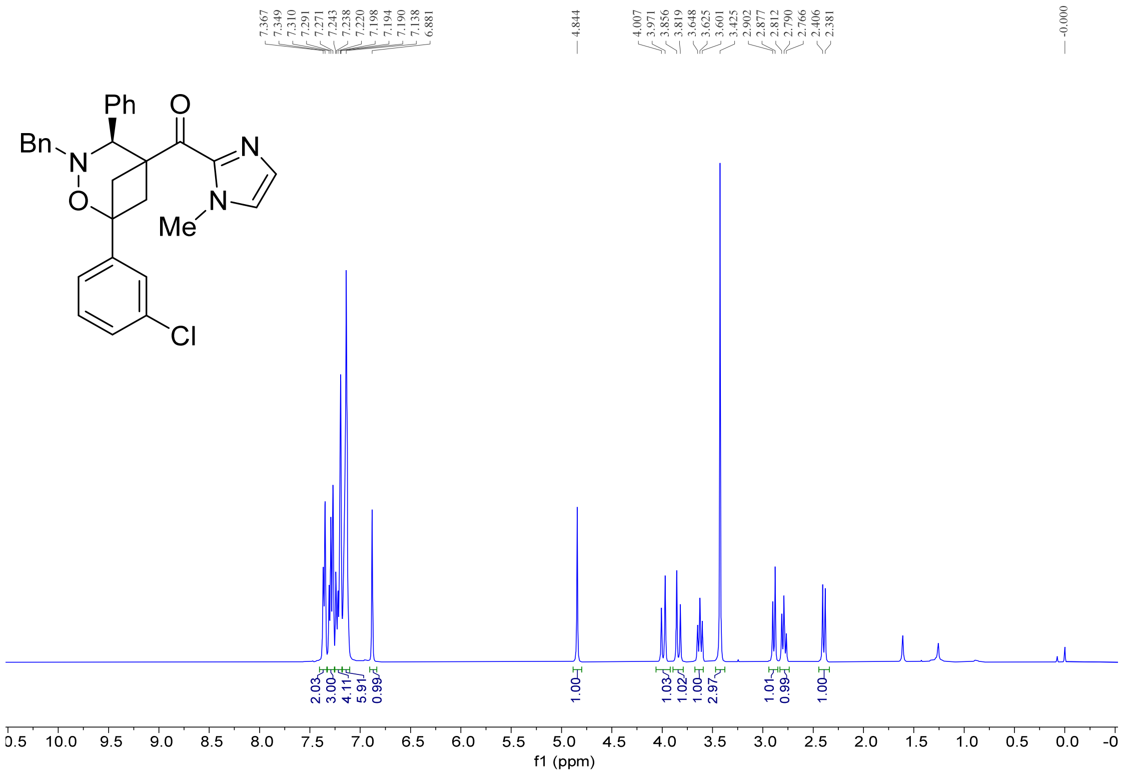

Supplementary Figure 108. ^{1}H NMR (400 MHz, $CDCl_3$) spectrum of compound **3gb**

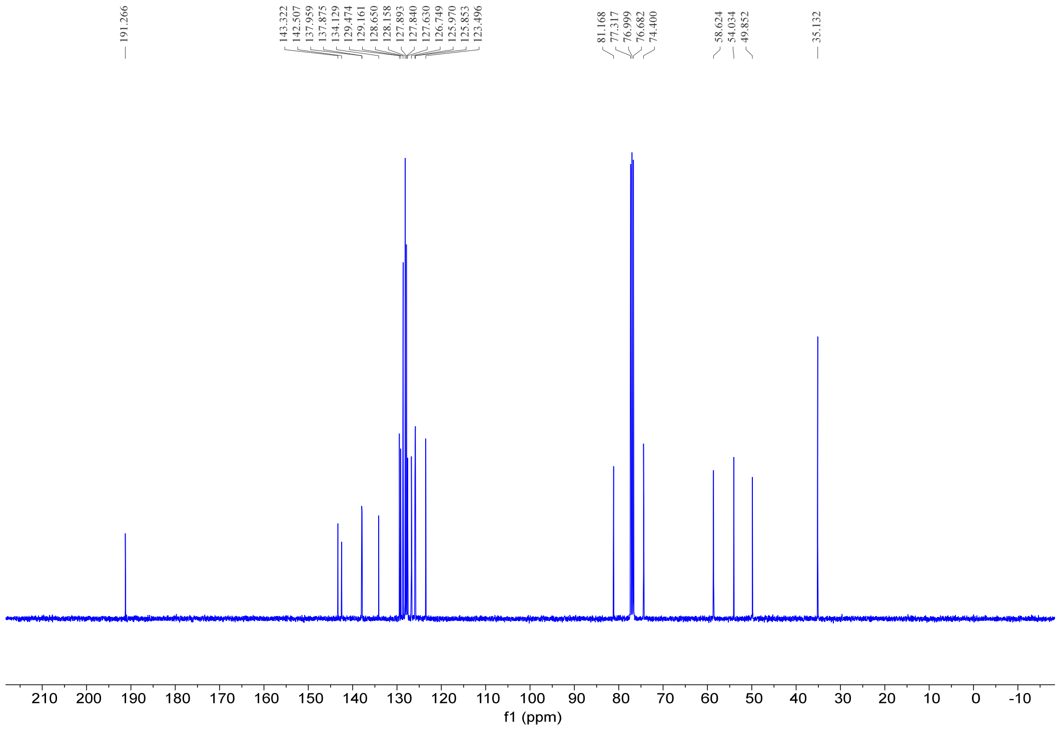

Supplementary Figure 109. ^{13}C NMR (100 MHz, $CDCl_3$) spectrum of compound **3gb**

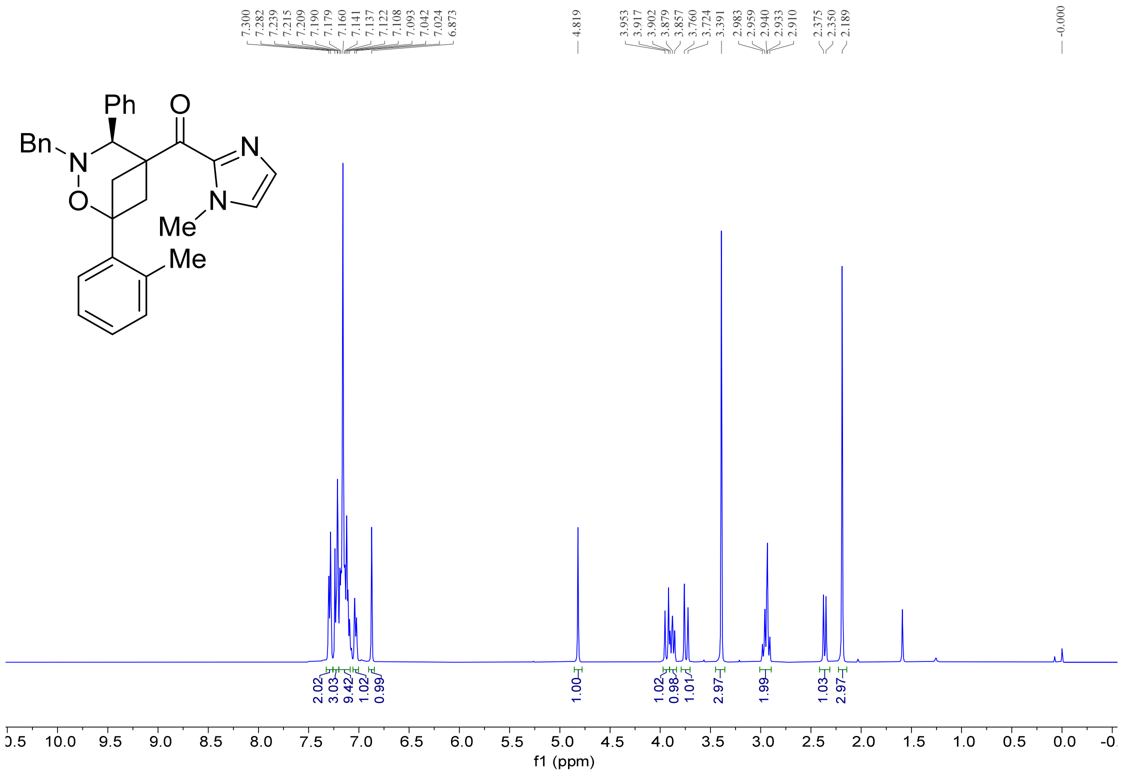

Supplementary Figure 110. ^{1}H NMR (400 MHz, $CDCl_3$) spectrum of compound **3hb**

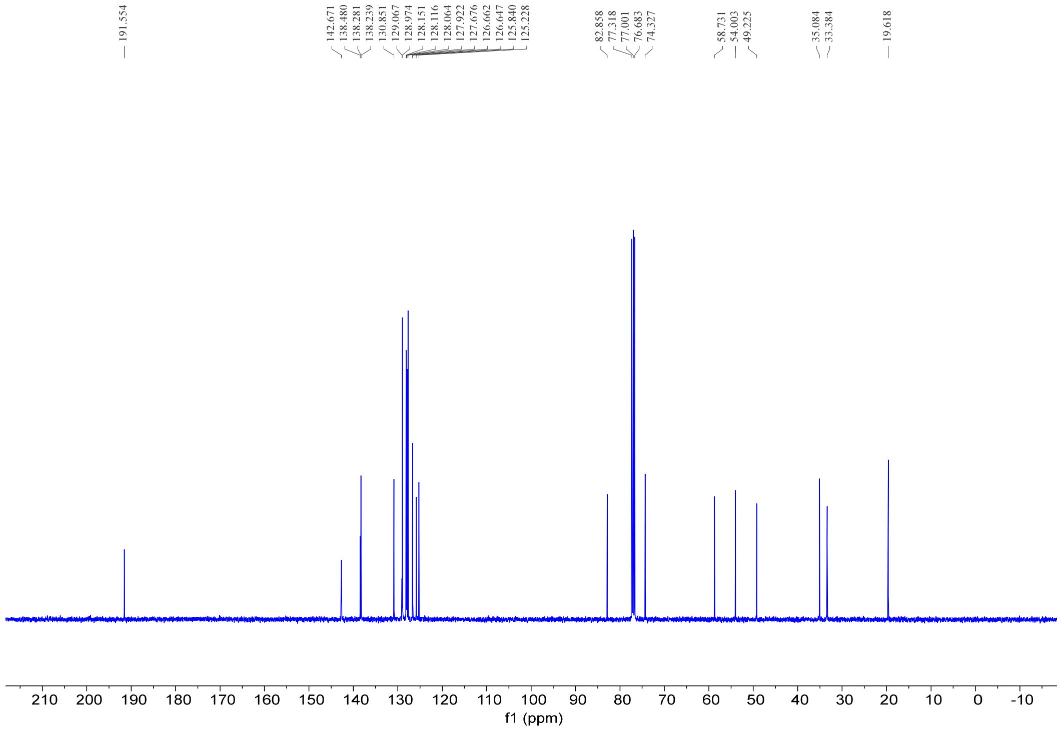

Supplementary Figure 111. ^{13}C NMR (100 MHz, $CDCl_3$) spectrum of compound **3hb**

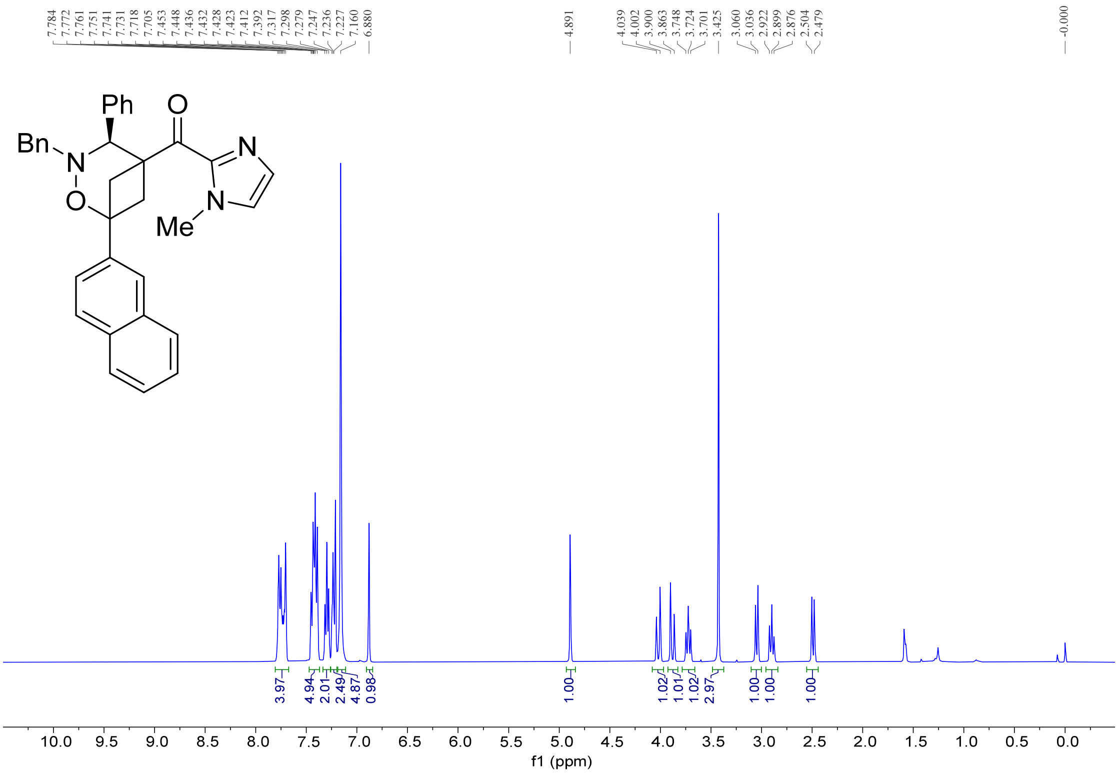

Supplementary Figure 112. ^{1}H NMR (400 MHz, $CDCl_3$) spectrum of compound **3ib**

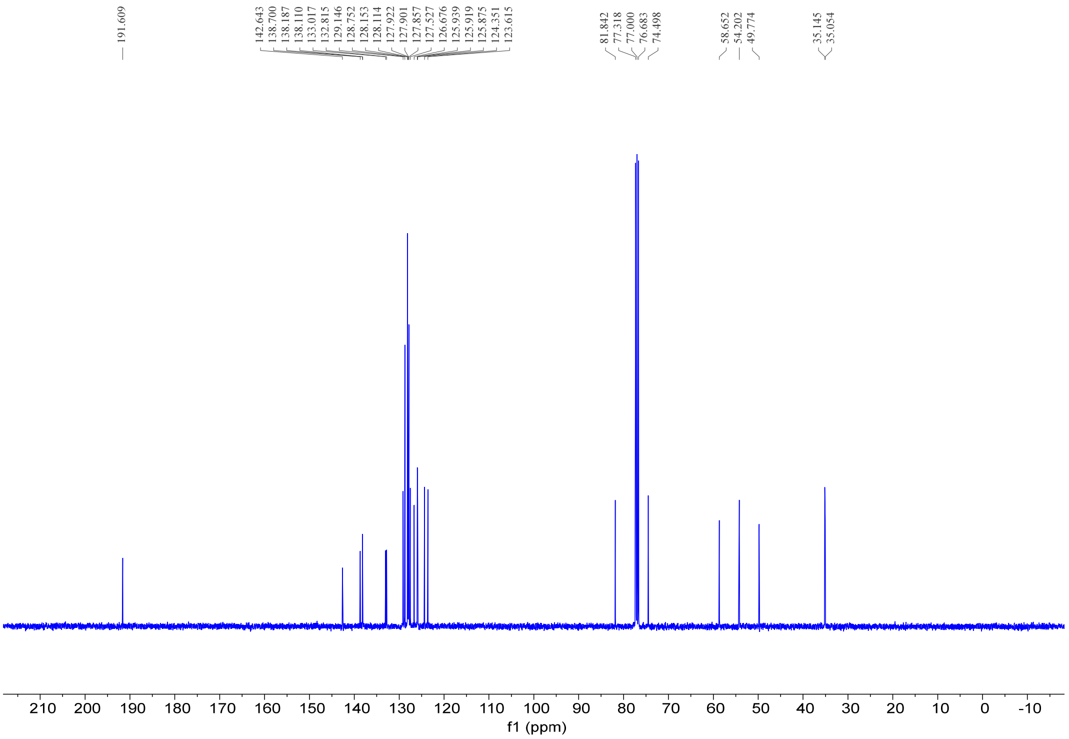

Supplementary Figure 113. ^{13}C NMR (100 MHz, $CDCl_3$) spectrum of compound **3ib**

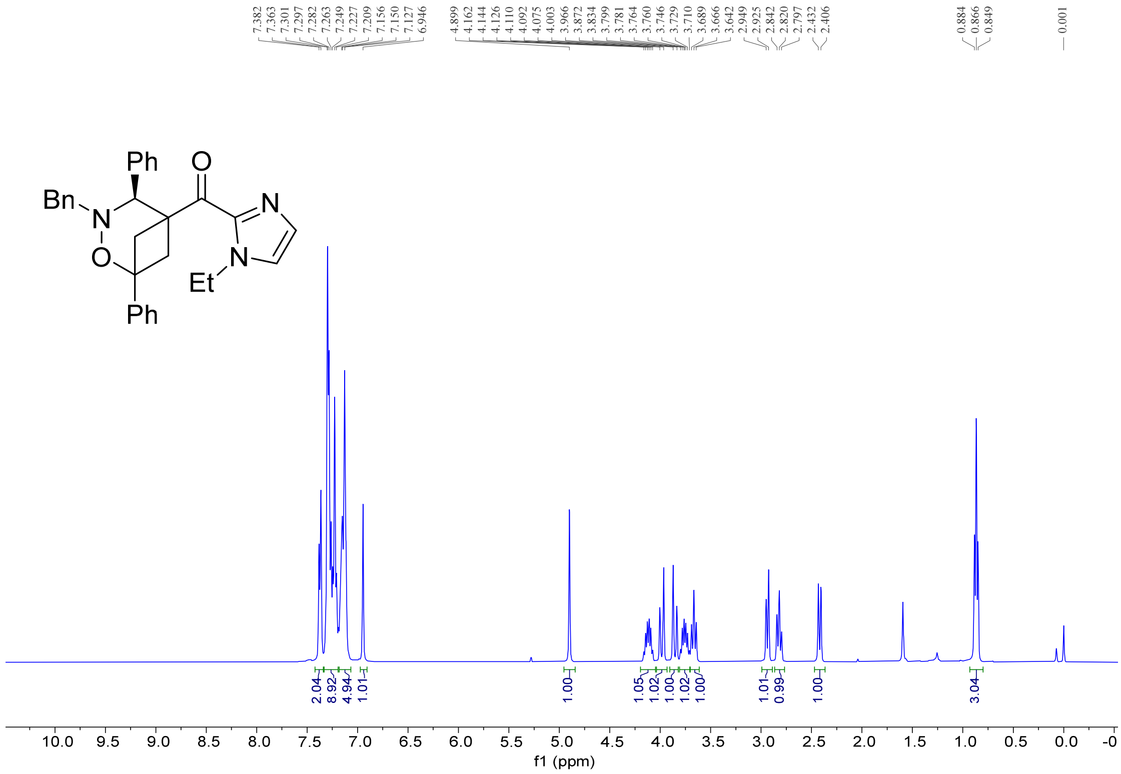

Supplementary Figure 114. ^{1}H NMR (400 MHz, $CDCl_3$) spectrum of compound **3jb**

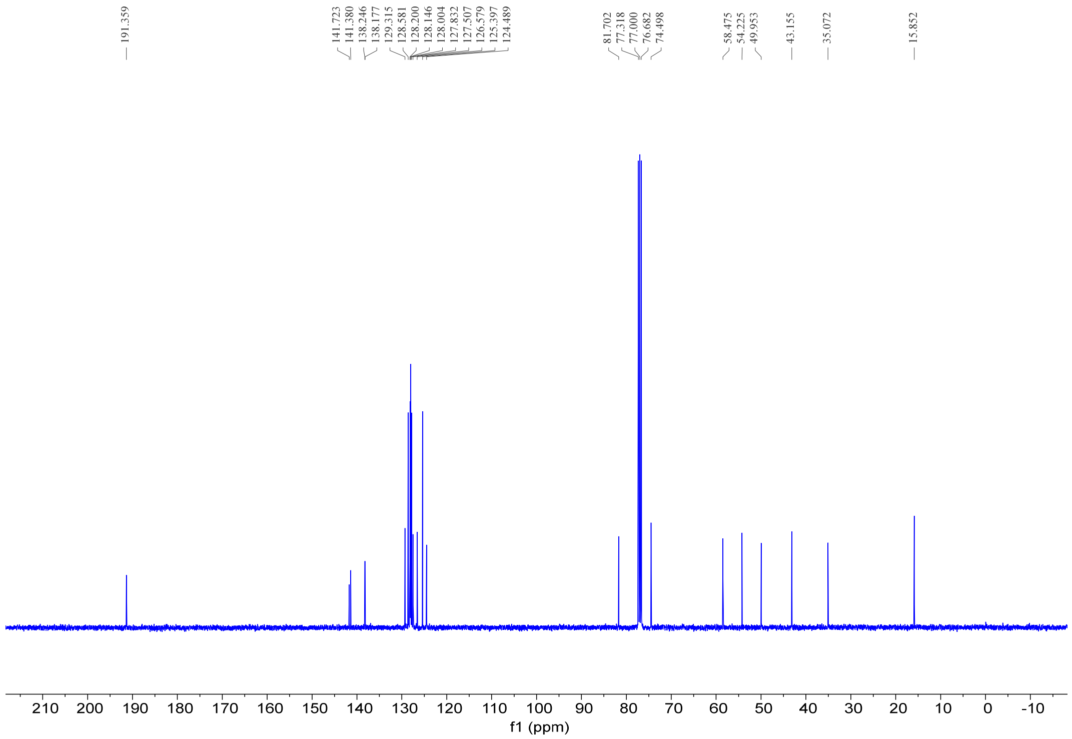

Supplementary Figure 115. ^{13}C NMR (100 MHz, $CDCl_3$) spectrum of compound **3jb**

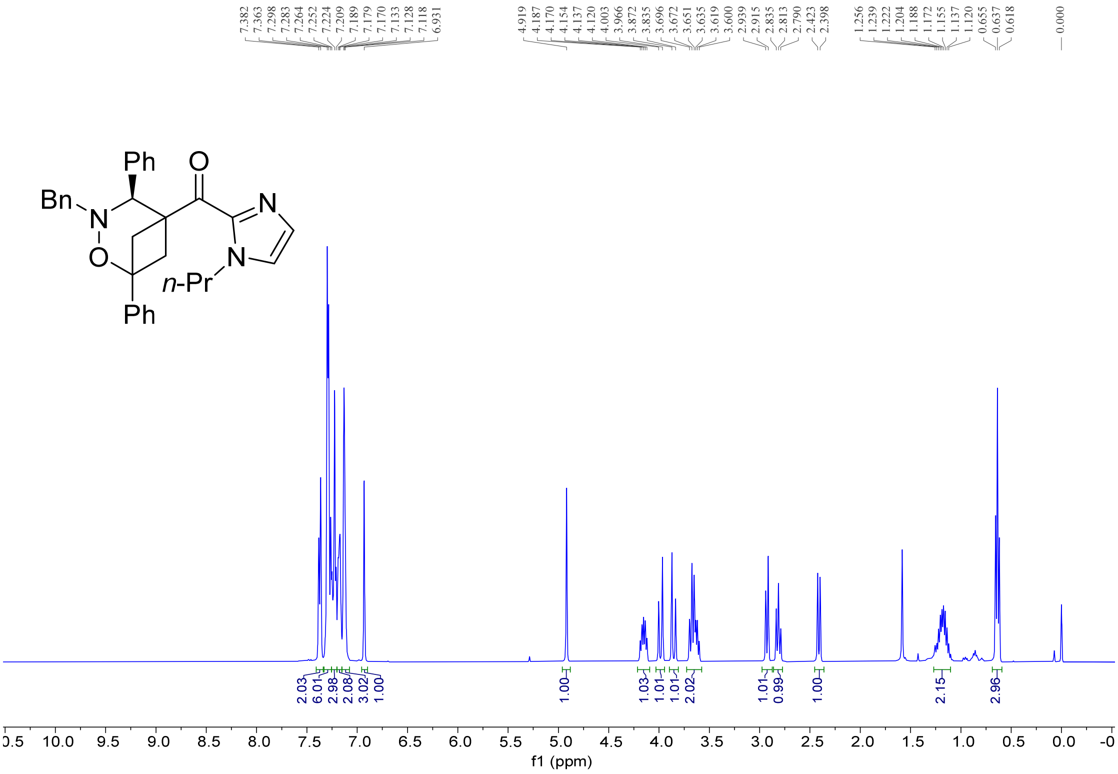

Supplementary Figure 116. ^{1}H NMR (400 MHz, $CDCl_3$) spectrum of compound **3kb**

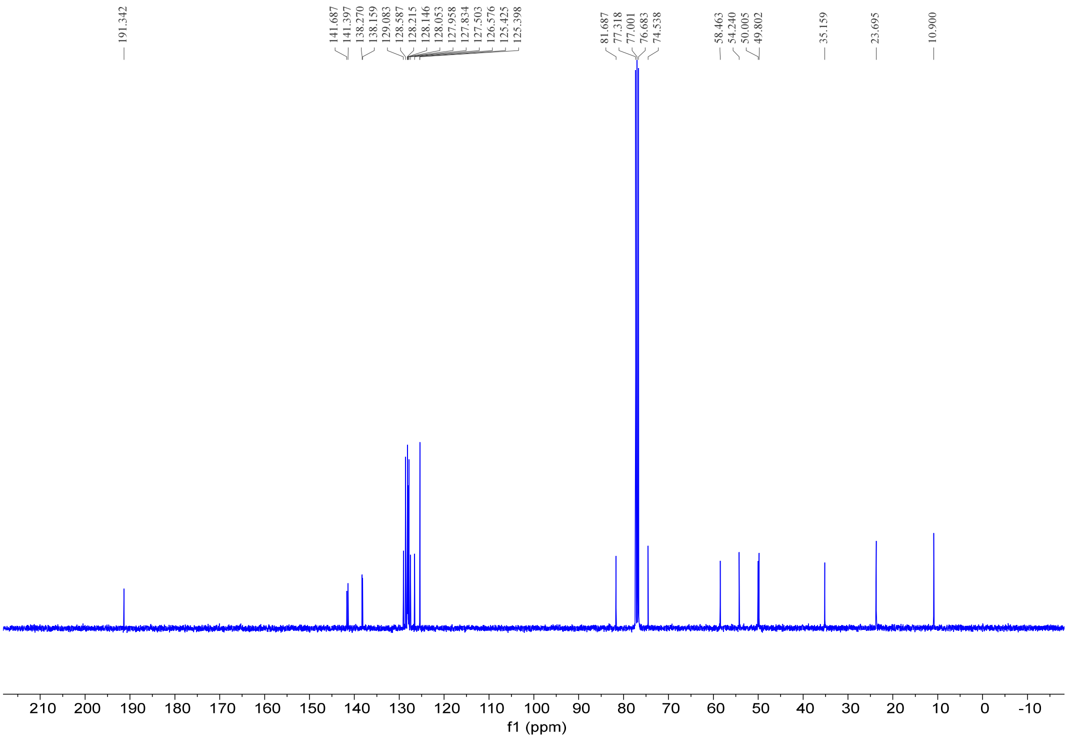

Supplementary Figure 117. ^{13}C NMR (100 MHz, $CDCl_3$) spectrum of compound **3kb**

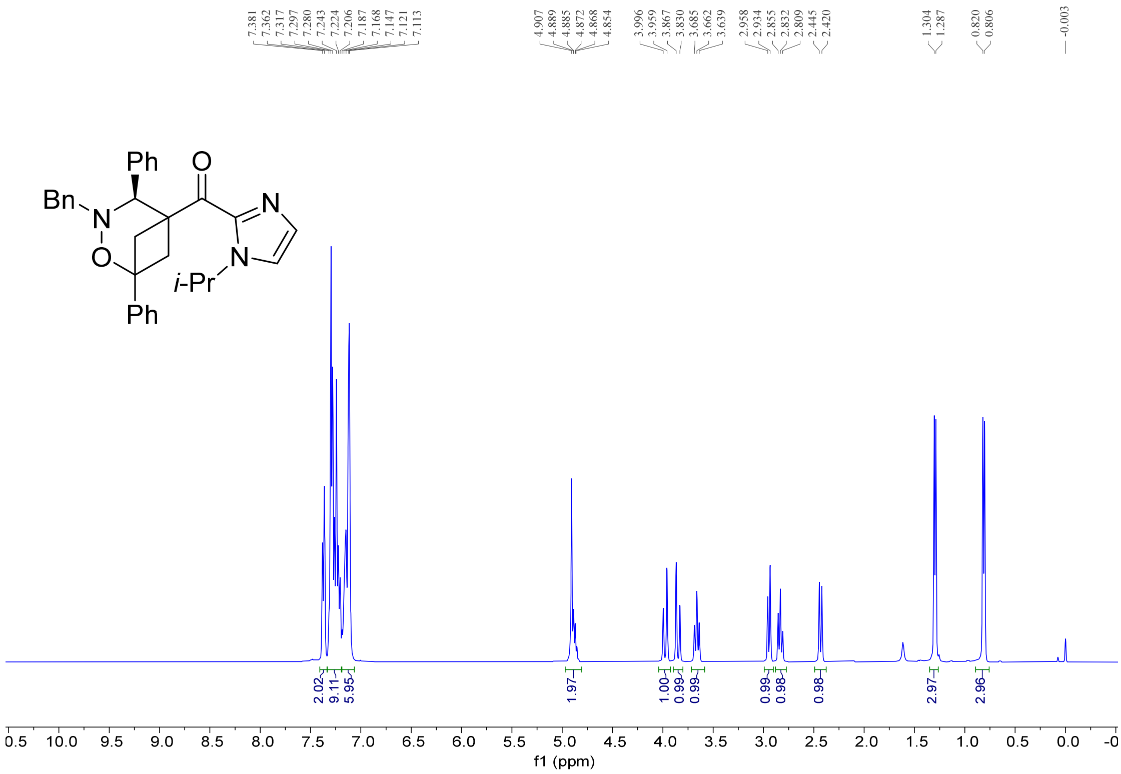

Supplementary Figure 118. ^{1}H NMR (400 MHz, $CDCl_3$) spectrum of compound **3lb**

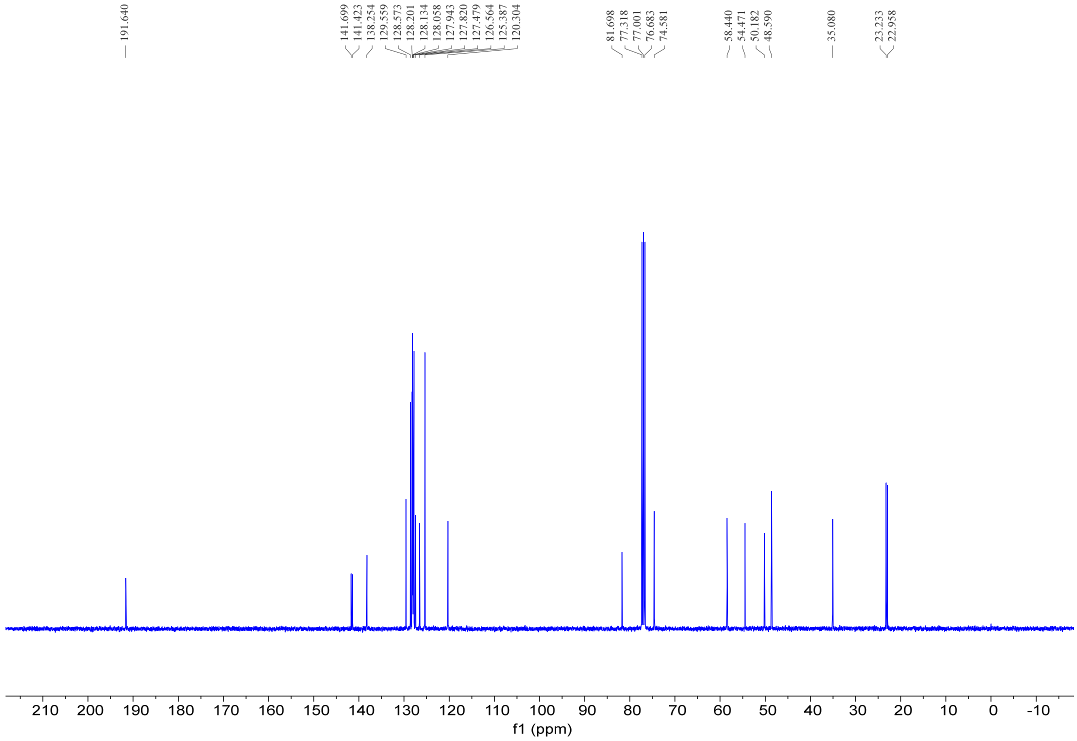

Supplementary Figure 119. ^{13}C NMR (100 MHz, $CDCl_3$) spectrum of compound **3lb**

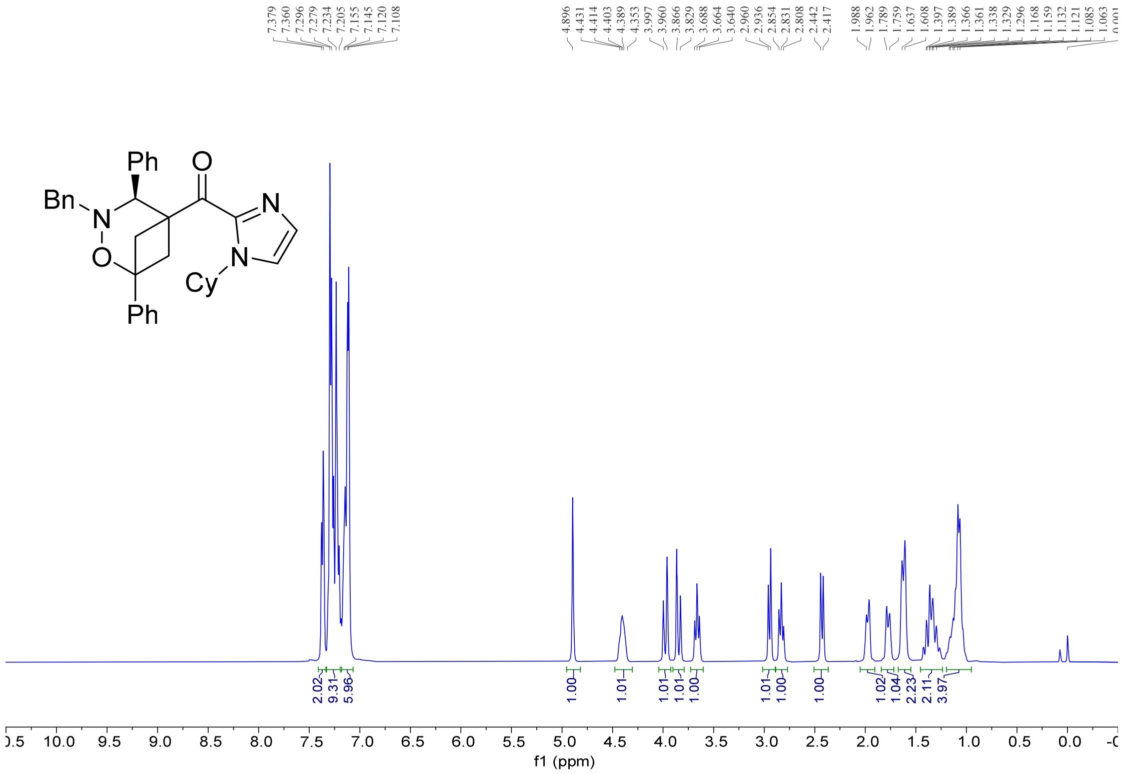

Supplementary Figure 120. ^{1}H NMR (400 MHz, $CDCl_3$) spectrum of compound **3mb**

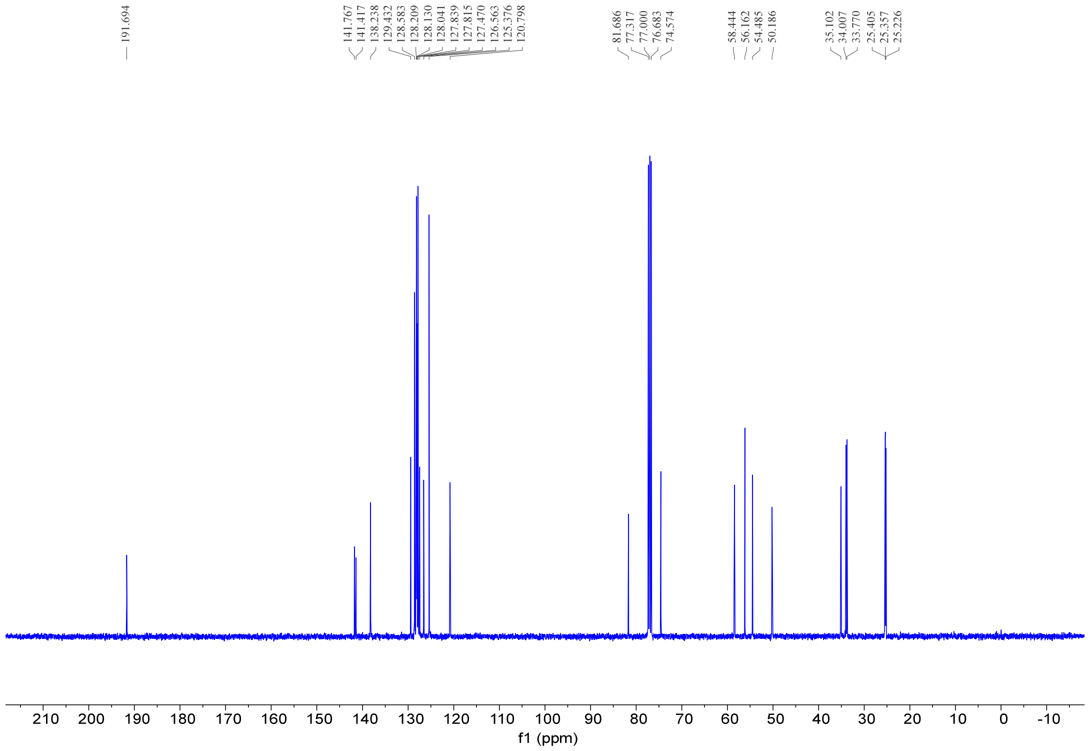

Supplementary Figure 121. ^{13}C NMR (100 MHz, $CDCl_3$) spectrum of compound **3mb**

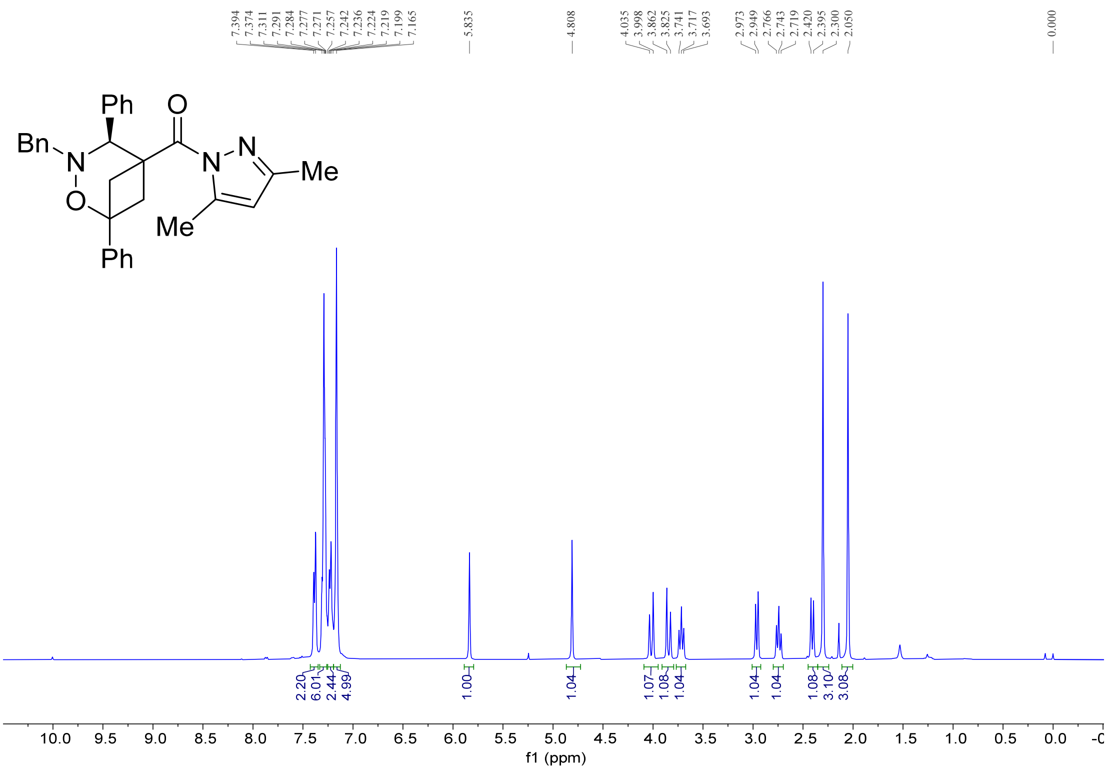

Supplementary Figure 122. ^{1}H NMR (400 MHz, $CDCl_3$) spectrum of compound **3nb**

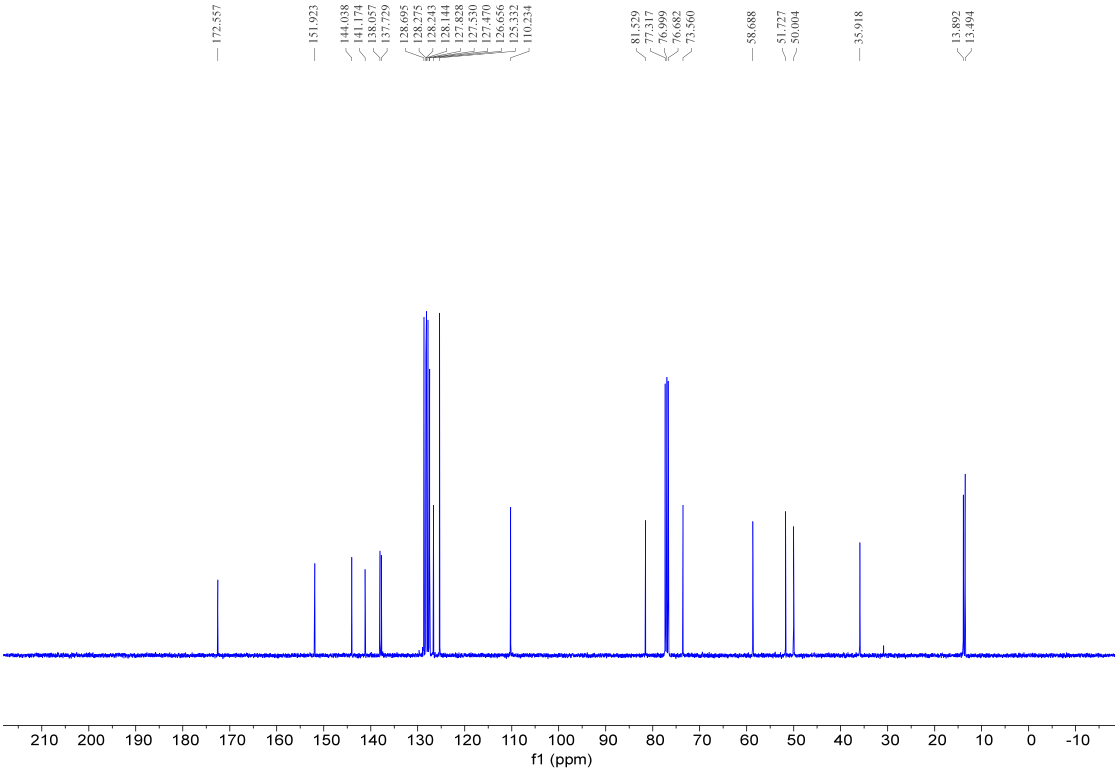

Supplementary Figure 123. ^{13}C NMR (100 MHz, $CDCl_3$) spectrum of compound **3nb**

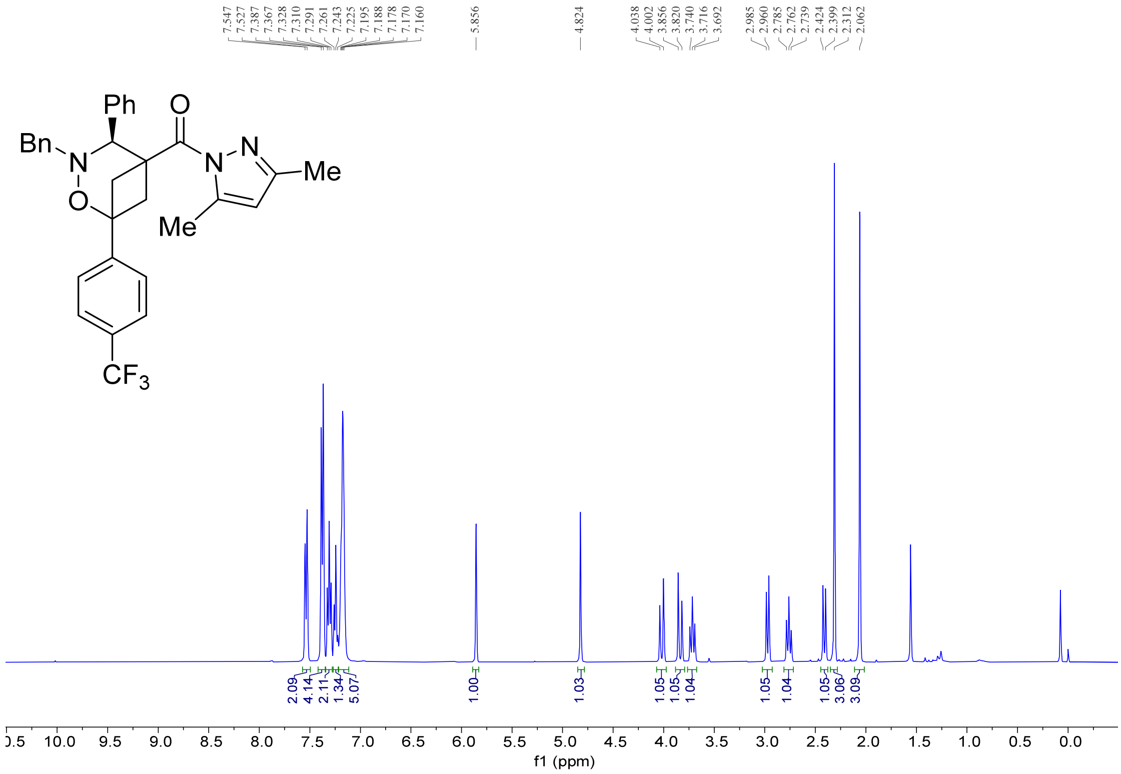

Supplementary Figure 124. ^{1}H NMR (400 MHz, $CDCl_3$) spectrum of compound **3ob**

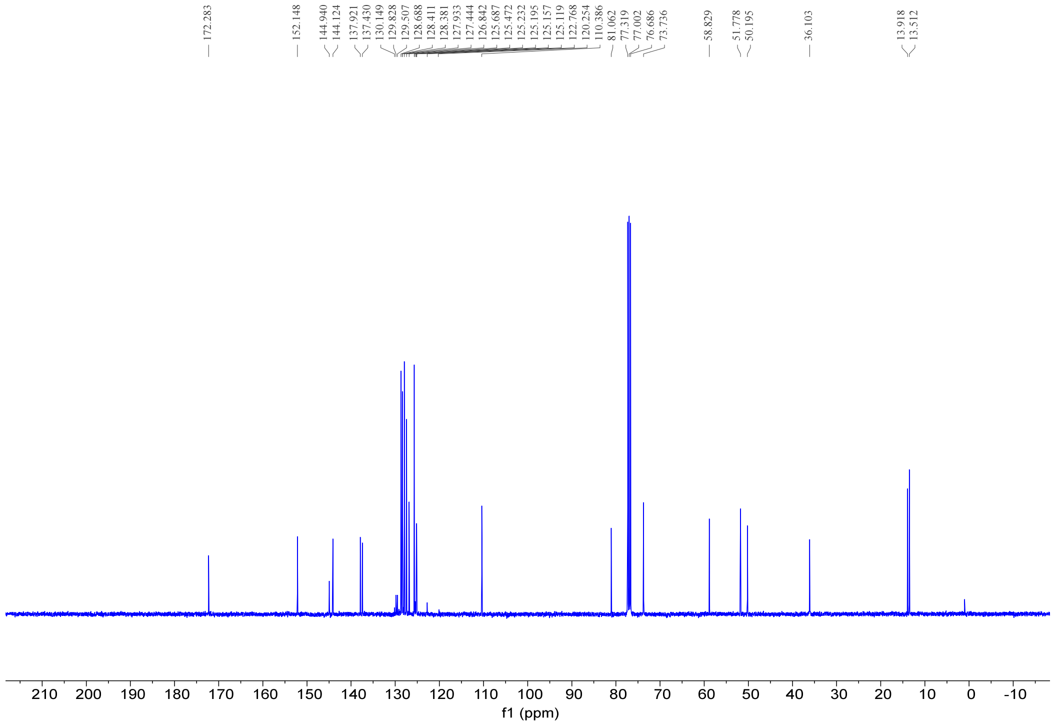

Supplementary Figure 125. ^{13}C NMR (100 MHz, $CDCl_3$) spectrum of compound **3ob**

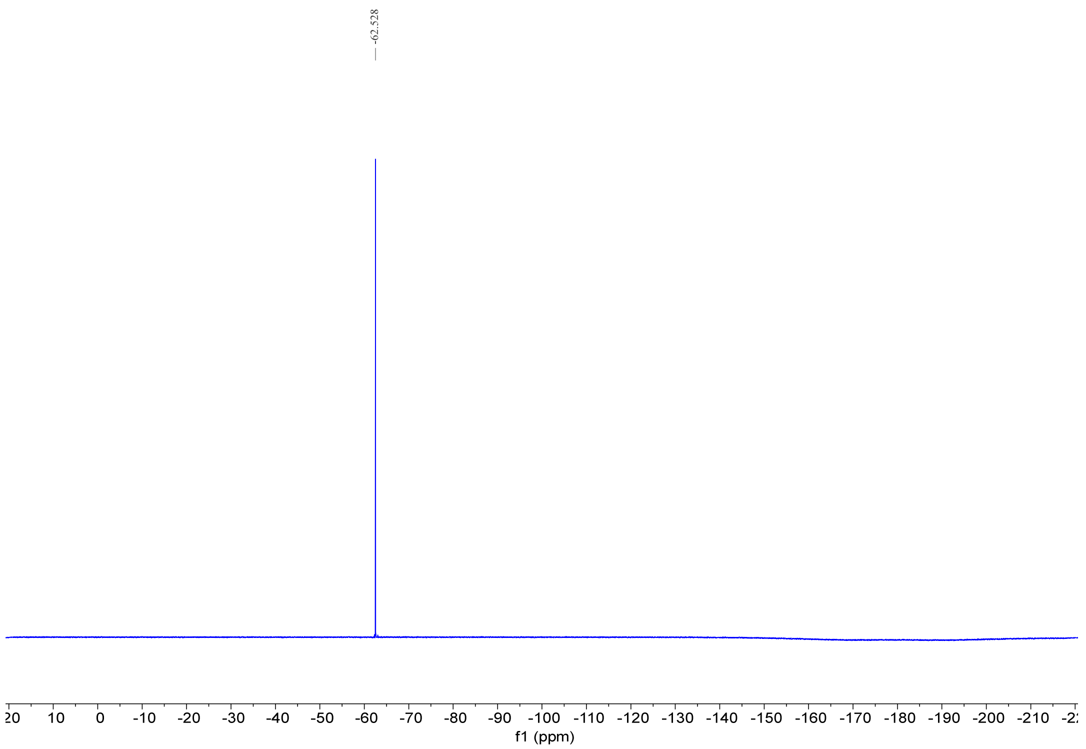

Supplementary Figure 126. ^{19}F NMR (376 MHz, $CDCl_3$) spectrum of compound **3ob**

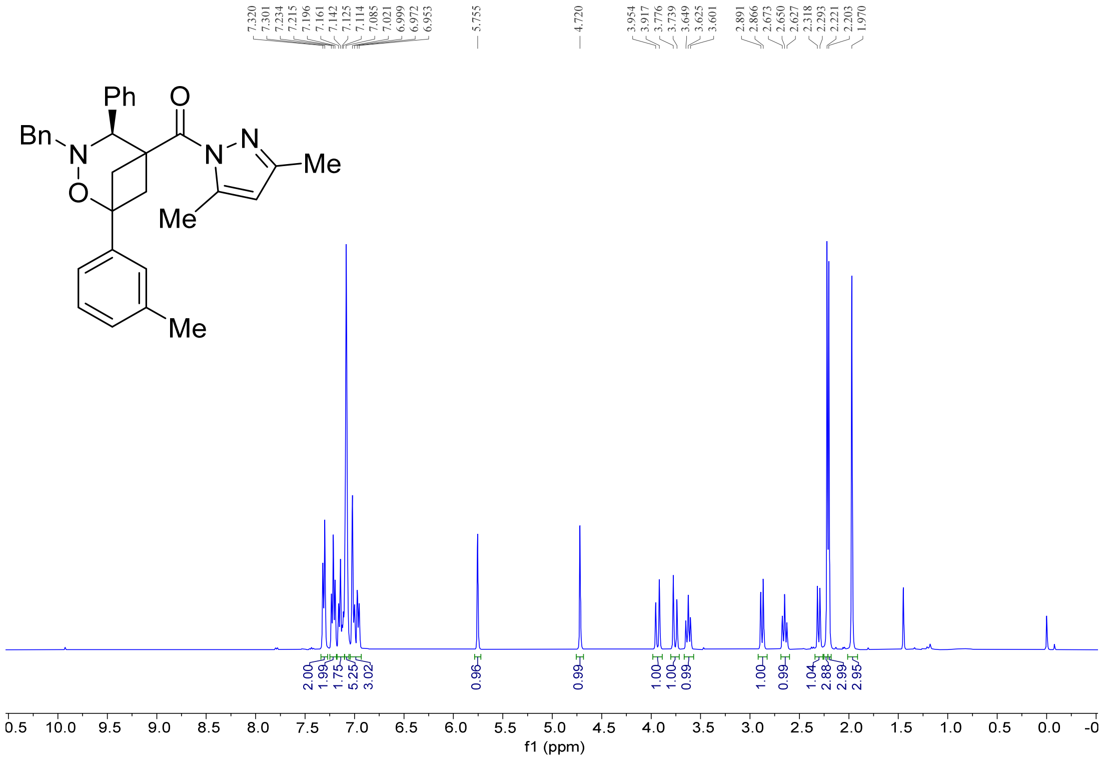

Supplementary Figure 127. ^{1}H NMR (400 MHz, $CDCl_3$) spectrum of compound **3pb**

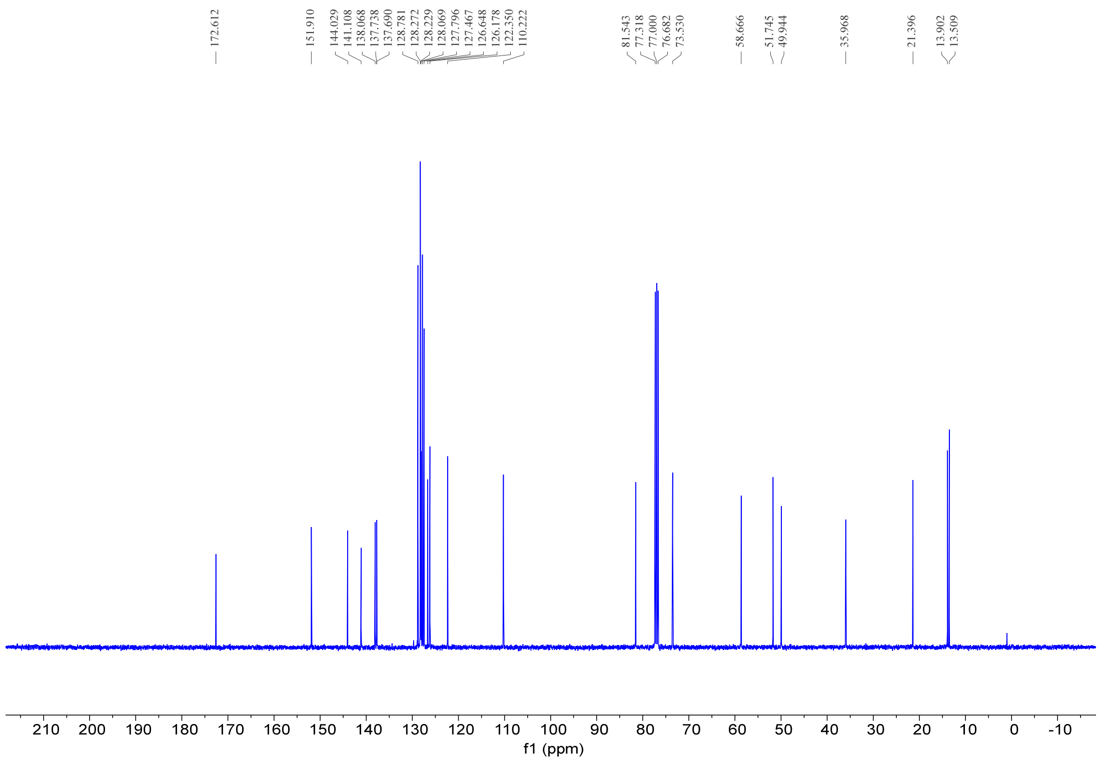

Supplementary Figure 128. ^{13}C NMR (100 MHz, $CDCl_3$) spectrum of compound **3pb**

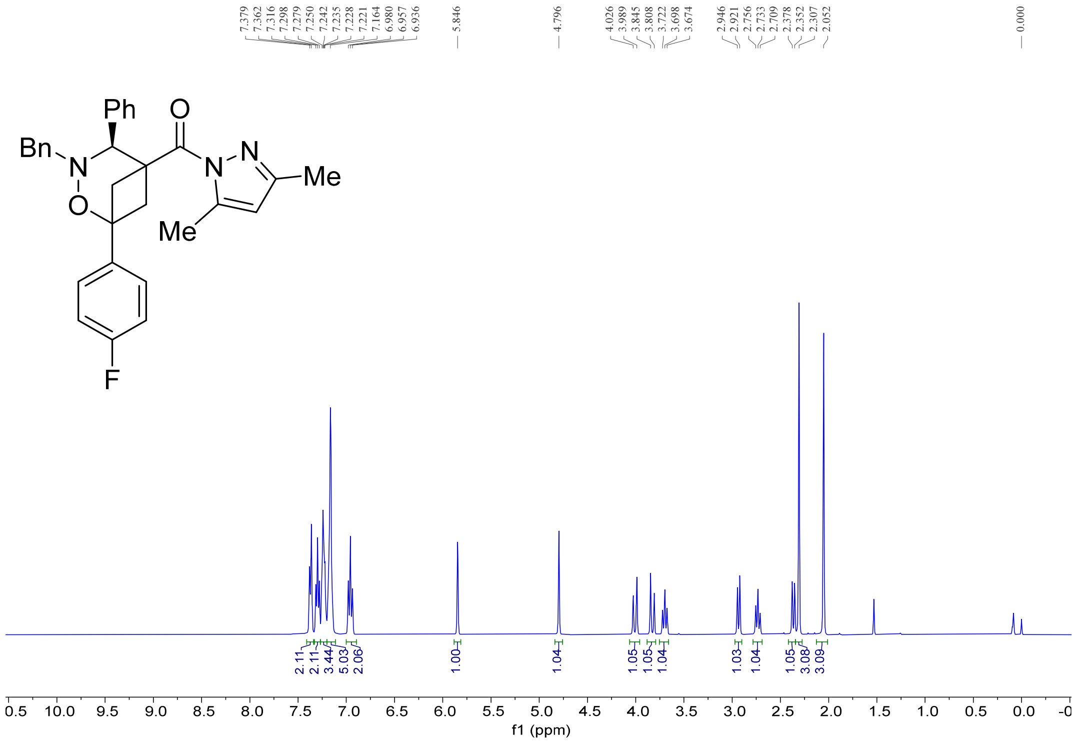

Supplementary Figure 129. ^{1}H NMR (400 MHz, $CDCl_3$) spectrum of compound **3qb**

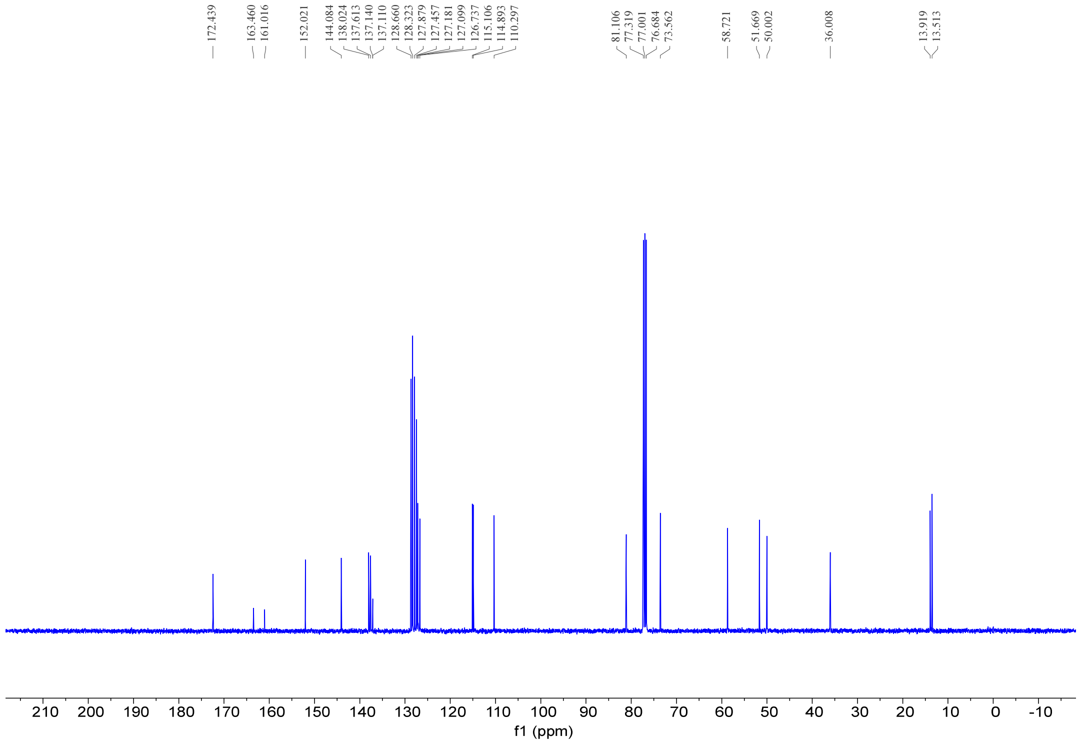

Supplementary Figure 130. ^{13}C NMR (100 MHz, $CDCl_3$) spectrum of compound **3qb**

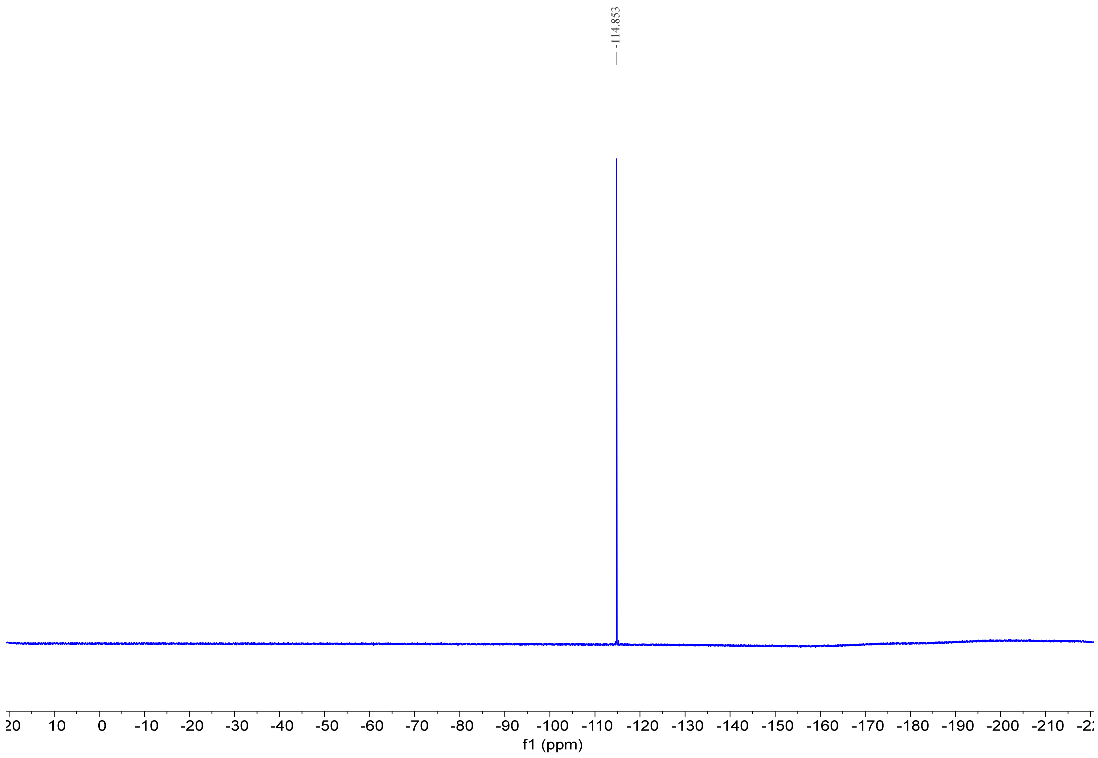

Supplementary Figure 131. ^{19}F NMR (376 MHz, $CDCl_3$) spectrum of compound **3qb**

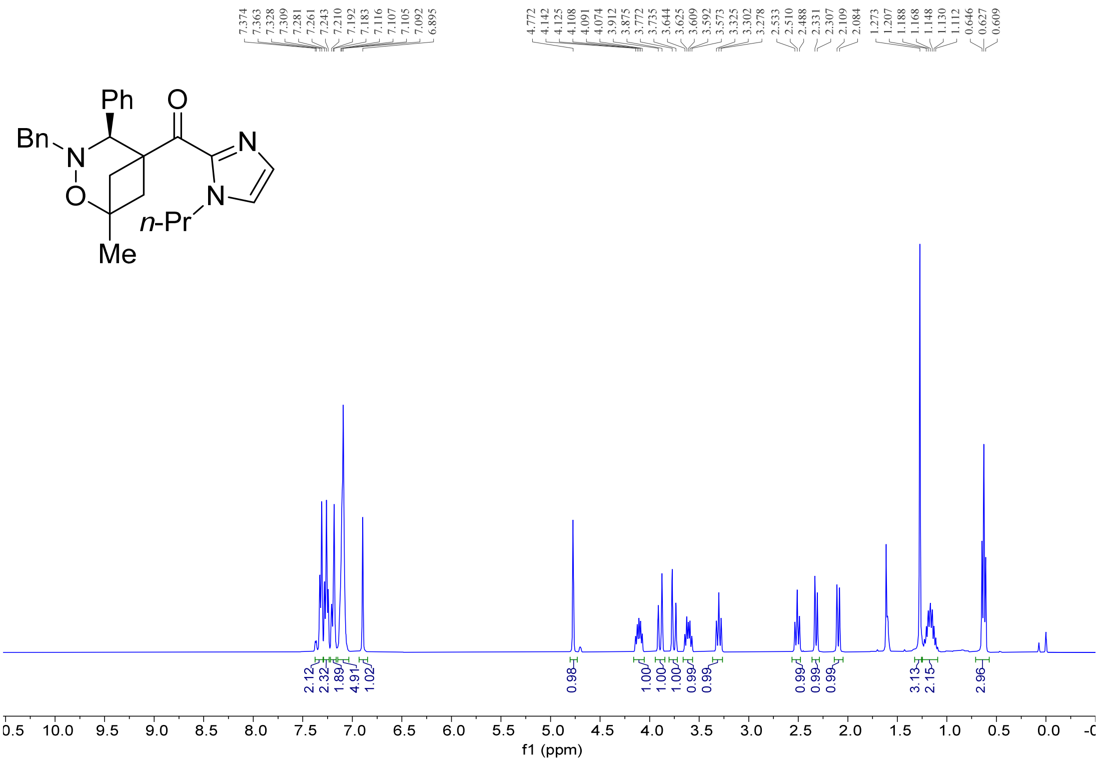

Supplementary Figure 132. ^{1}H NMR (400 MHz, $CDCl_3$) spectrum of compound **3rb**

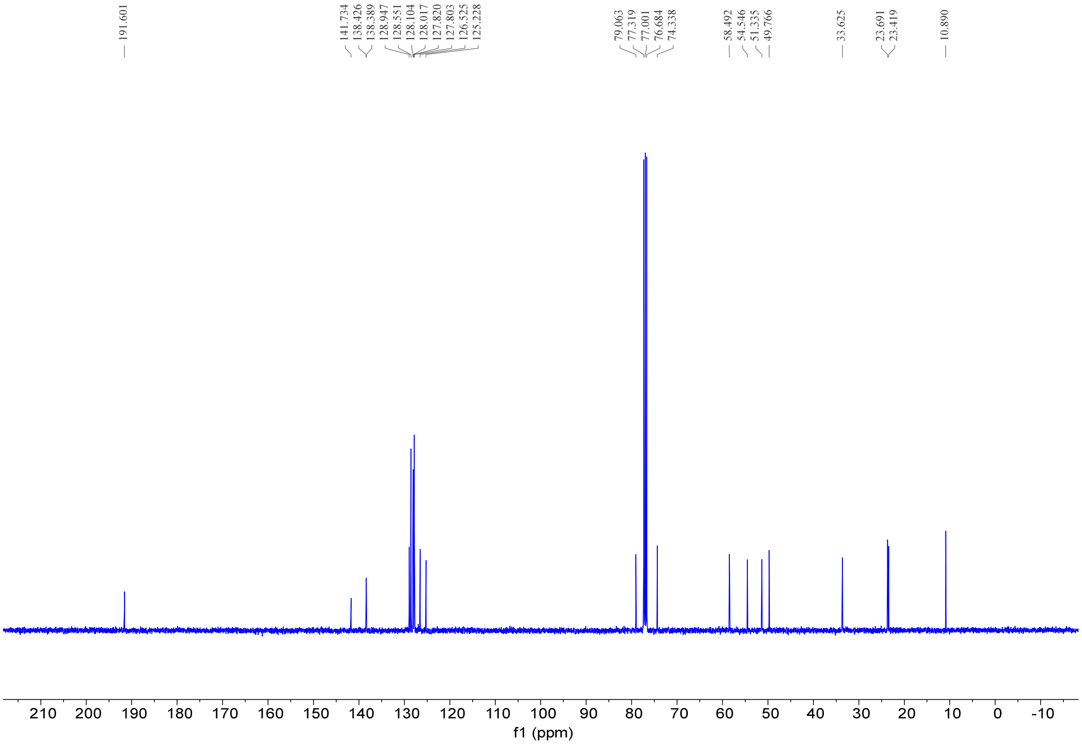

Supplementary Figure 133. ^{13}C NMR (100 MHz, $CDCl_3$) spectrum of compound **3rb**

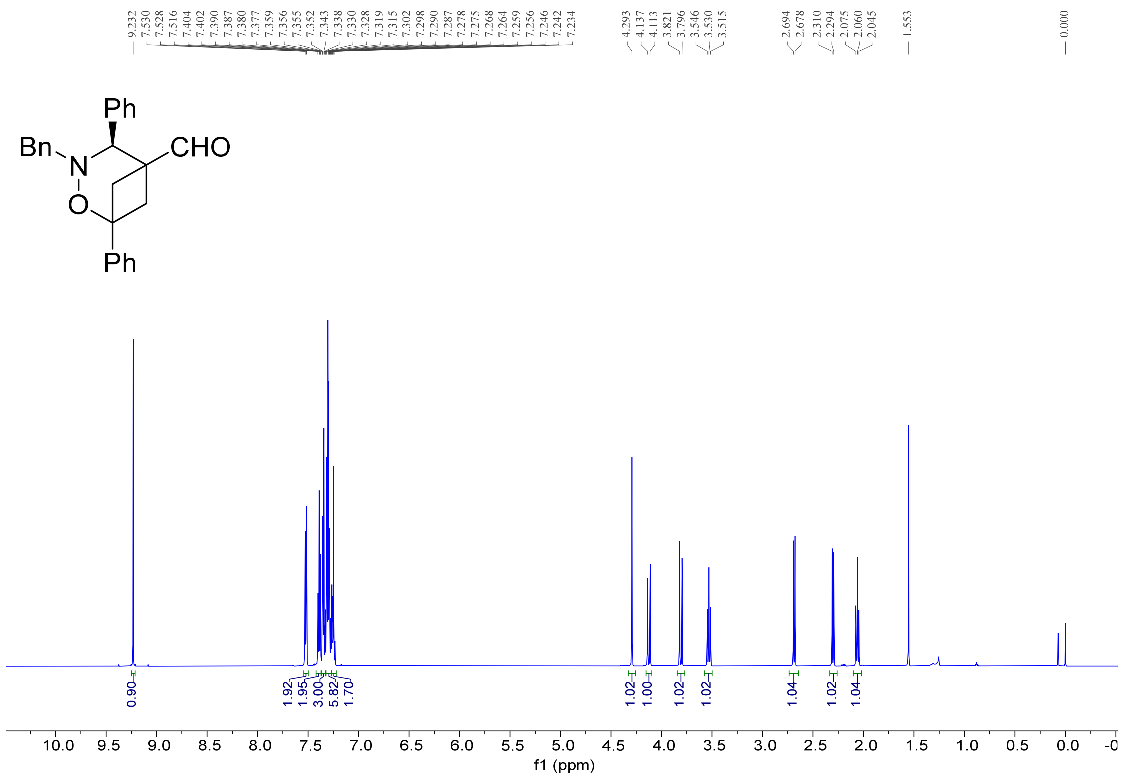

Supplementary Figure 134. ^{1}H NMR (600 MHz, $CDCl_3$) spectrum of compound **4nb**

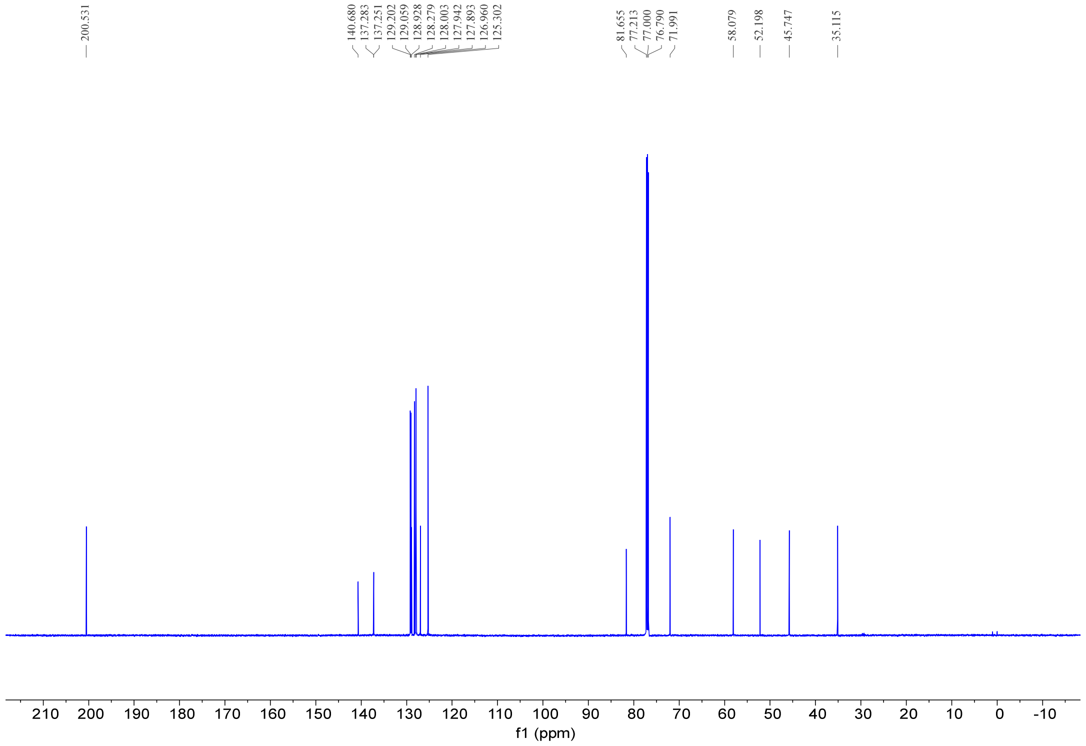

Supplementary Figure 135. ^{13}C NMR (150 MHz, $CDCl_3$) spectrum of compound **4nb**

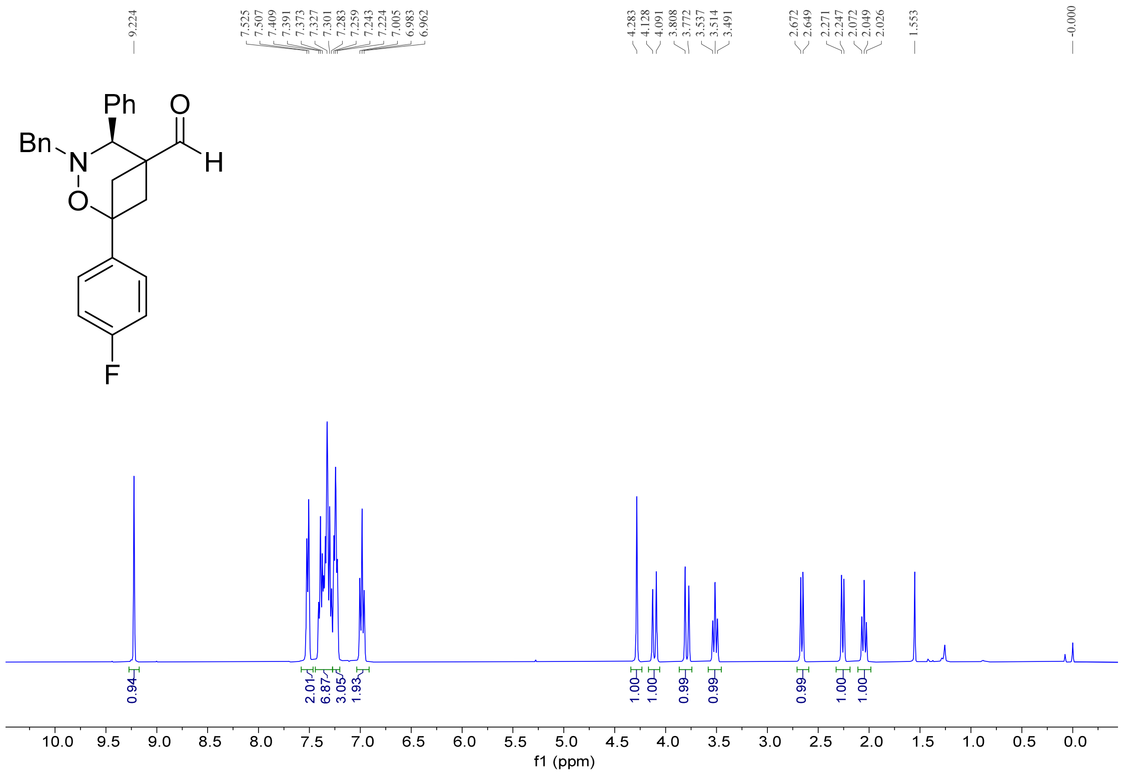

Supplementary Figure 136. ^{1}H NMR (400 MHz, $CDCl_3$) spectrum of compound **4qb**

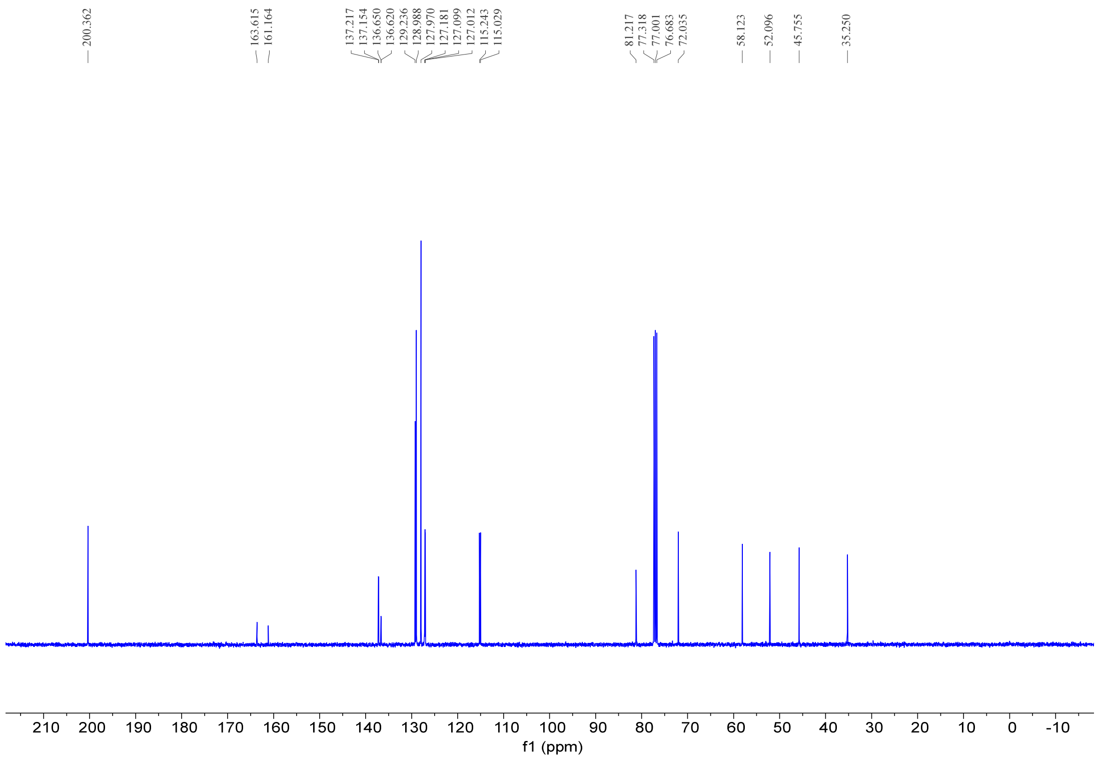

Supplementary Figure 137. ^{13}C NMR (100 MHz, $CDCl_3$) spectrum of compound **4qb**

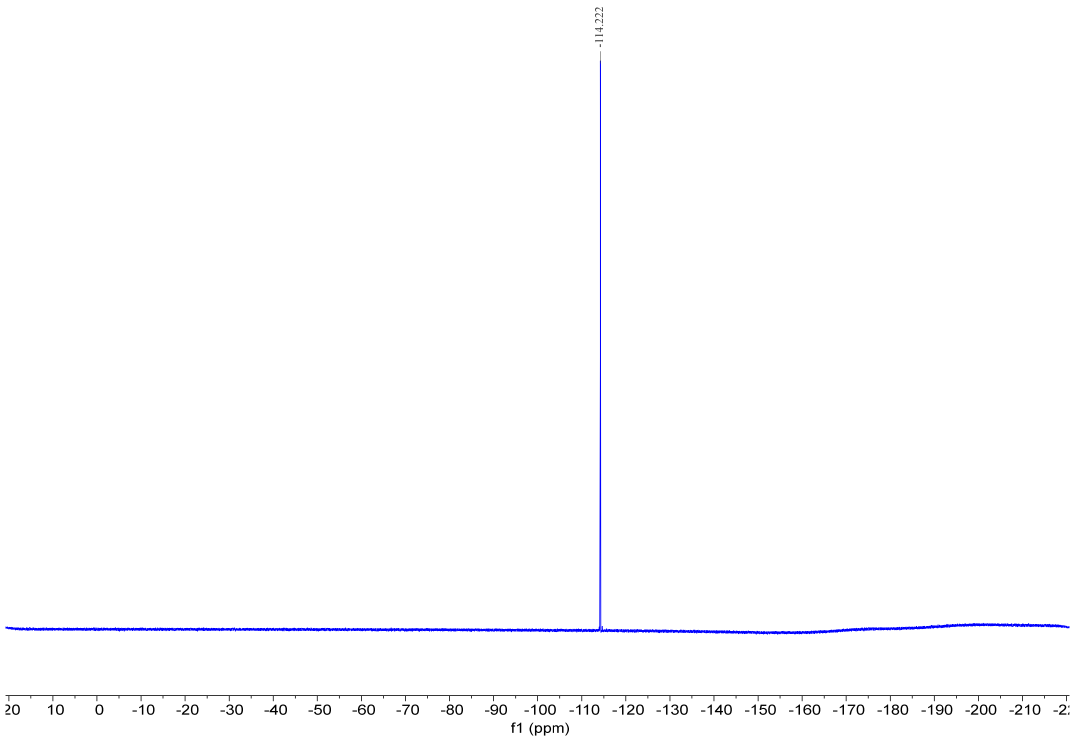

Supplementary Figure 138. ^{19}F NMR (376 MHz, $CDCl_3$) spectrum of compound **4qb**

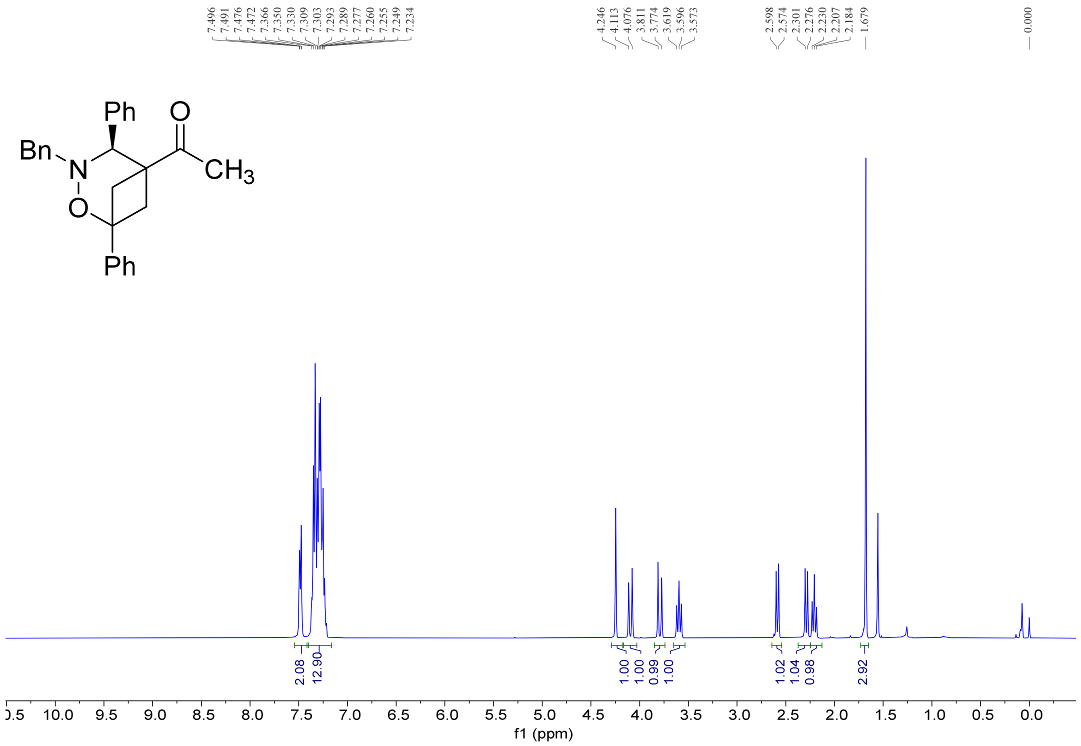

Supplementary Figure 139. ^{1}H NMR (400 MHz, $CDCl_3$) spectrum of compound **5**

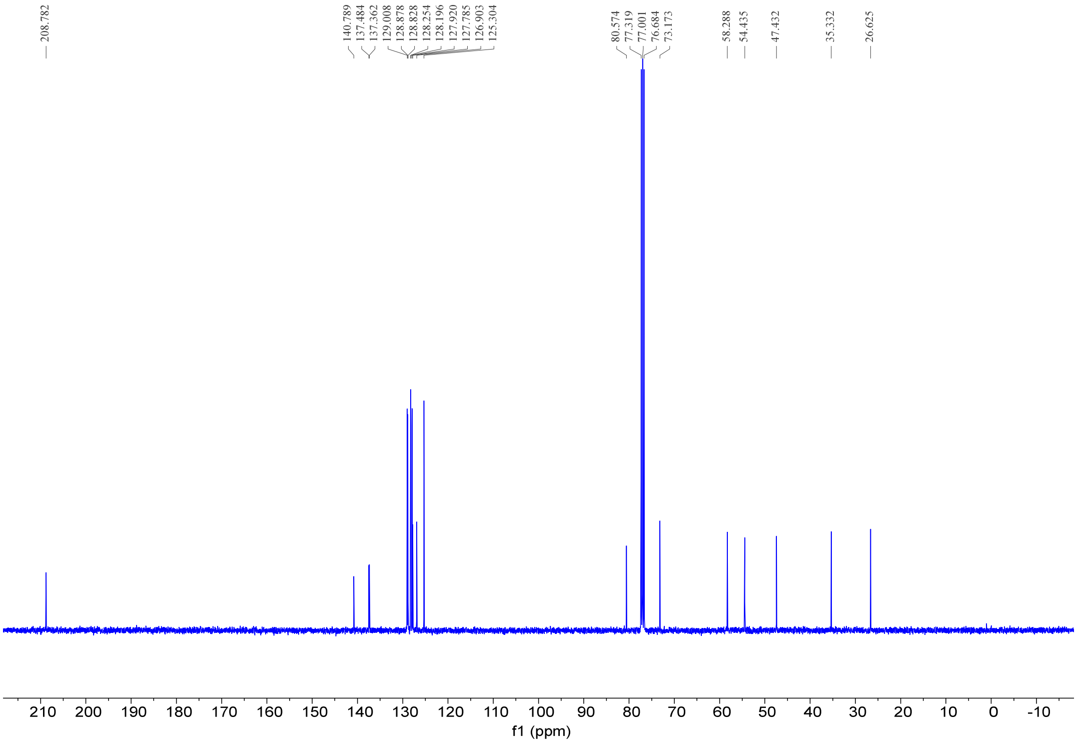

Supplementary Figure 140. ^{13}C NMR (100 MHz, $CDCl_3$) spectrum of compound **5**

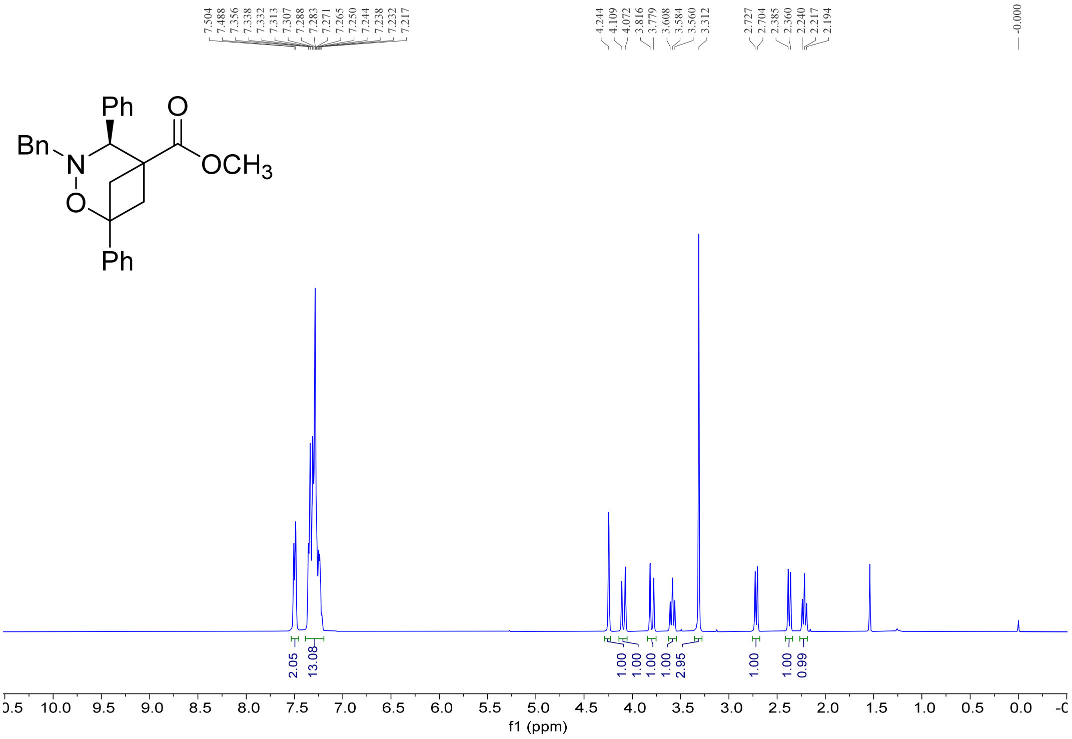

Supplementary Figure 141. ^{1}H NMR (400 MHz, $CDCl_3$) spectrum of compound **6**

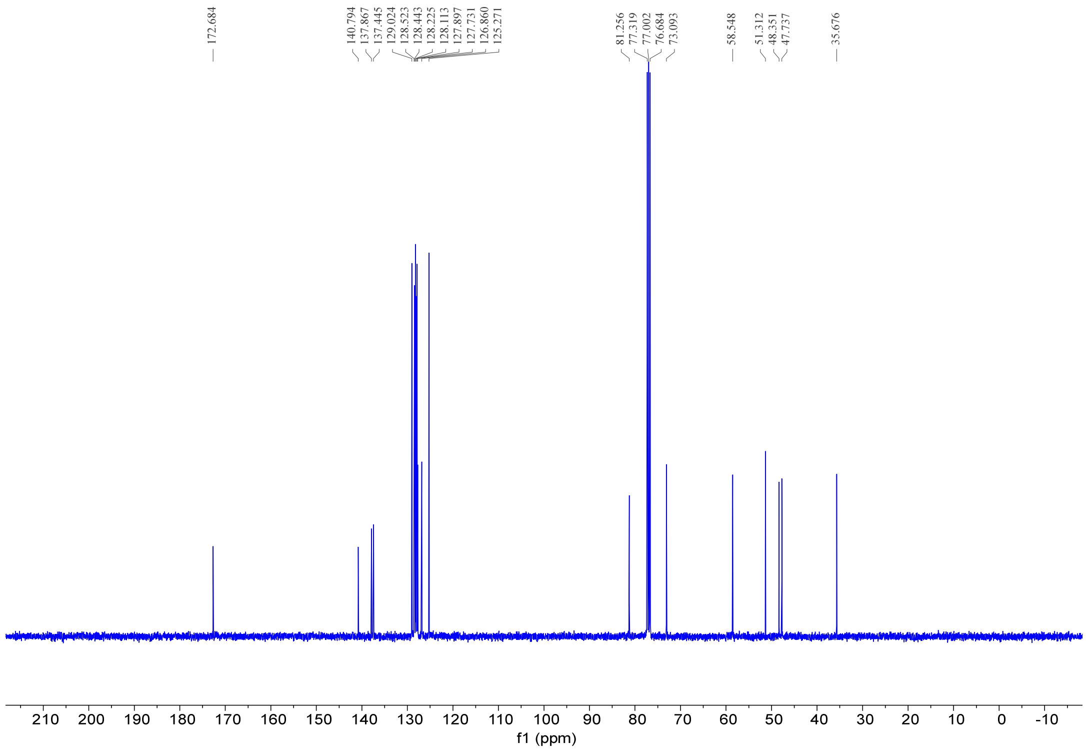

Supplementary Figure 142. ^{13}C NMR (100 MHz, $CDCl_3$) spectrum of compound **6**

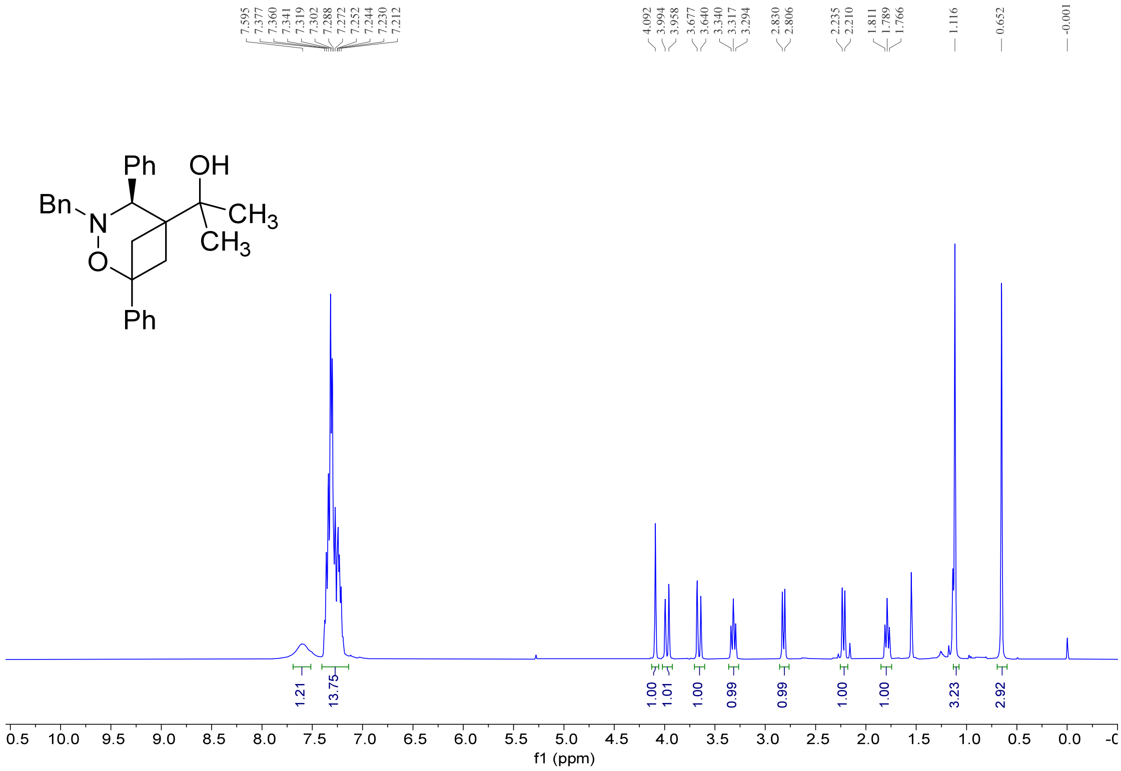

Supplementary Figure 143. ^{1}H NMR (400 MHz, $CDCl_3$) spectrum of compound **7**

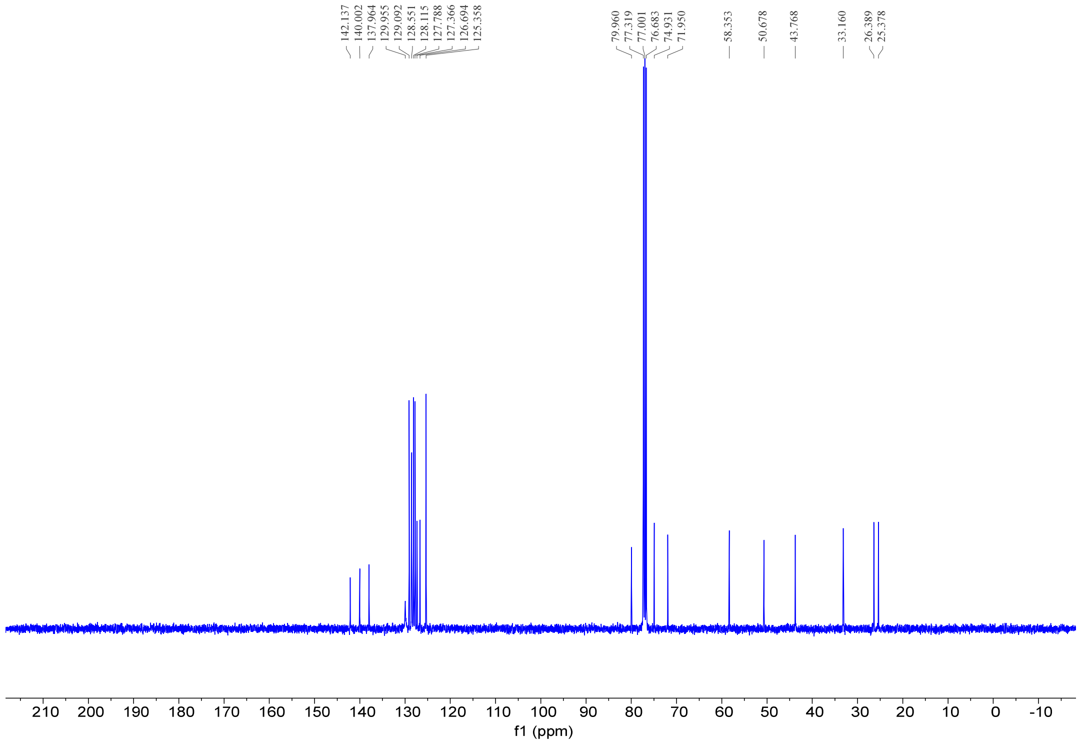

Supplementary Figure 144. ^{13}C NMR (100 MHz, $CDCl_3$) spectrum of compound **7**

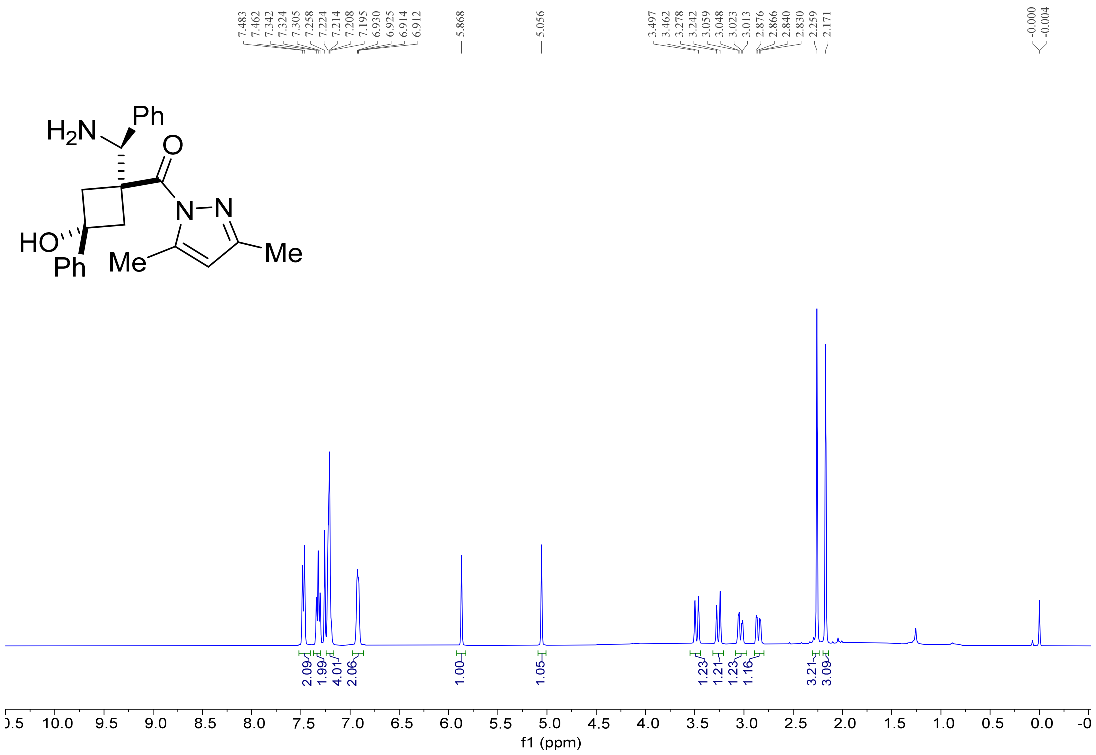

Supplementary Figure 145. ^{1}H NMR (400 MHz, $CDCl_3$) spectrum of compound **8**

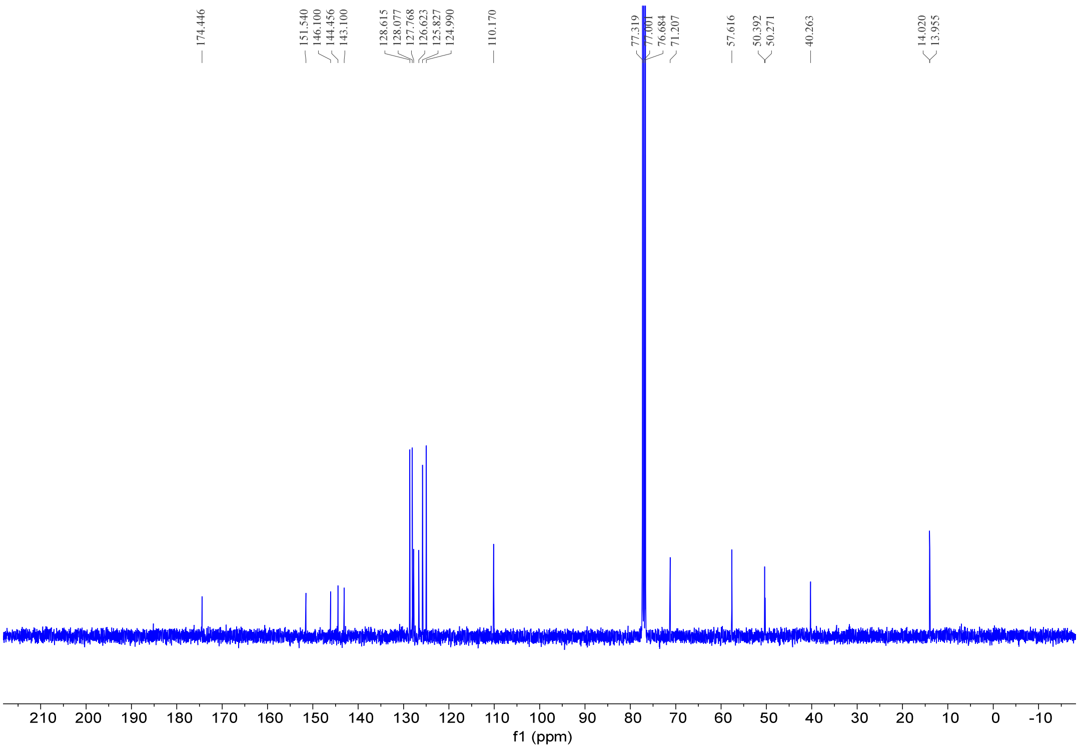

Supplementary Figure 146. ^{13}C NMR (100 MHz, $CDCl_3$) spectrum of compound **8**

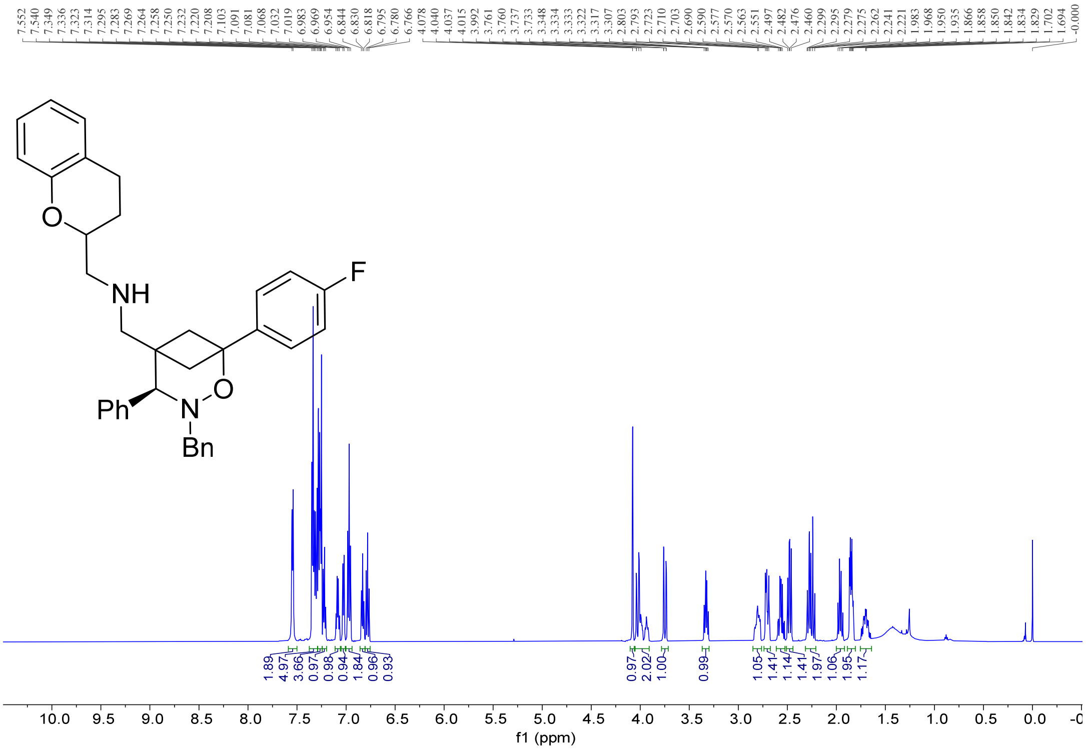

Supplementary Figure 147. ^{1}H NMR (600 MHz, $CDCl_3$) spectrum of compound **9**

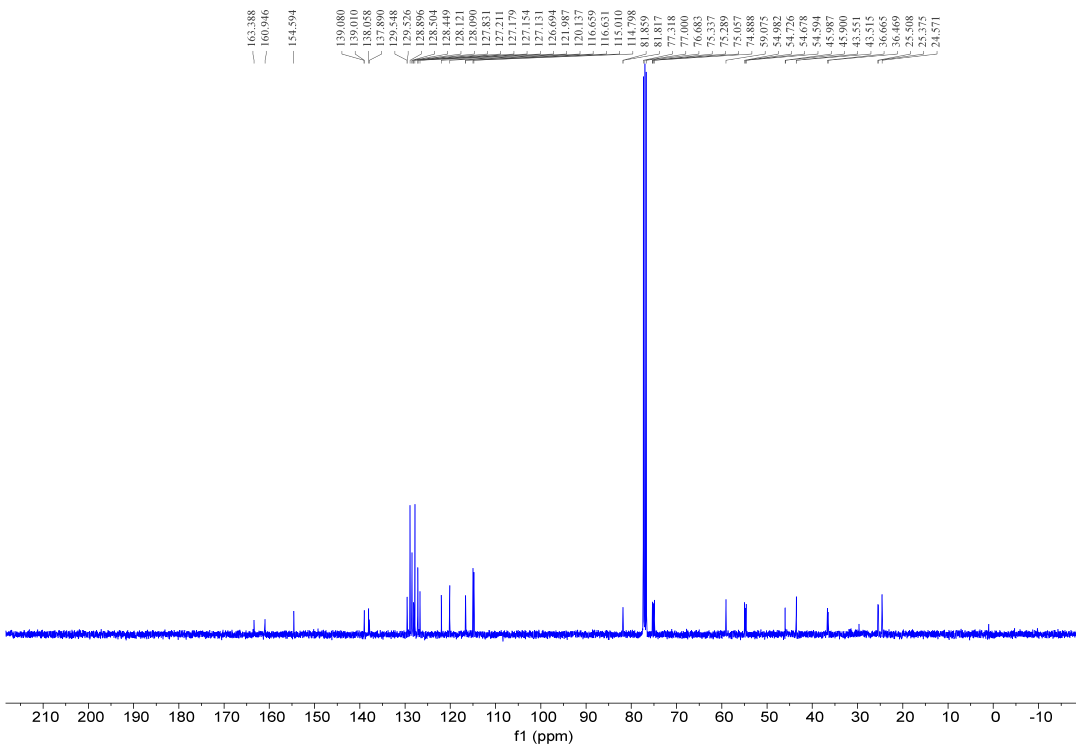

Supplementary Figure 148. ^{13}C NMR (100 MHz, $CDCl_3$) spectrum of compound **9**

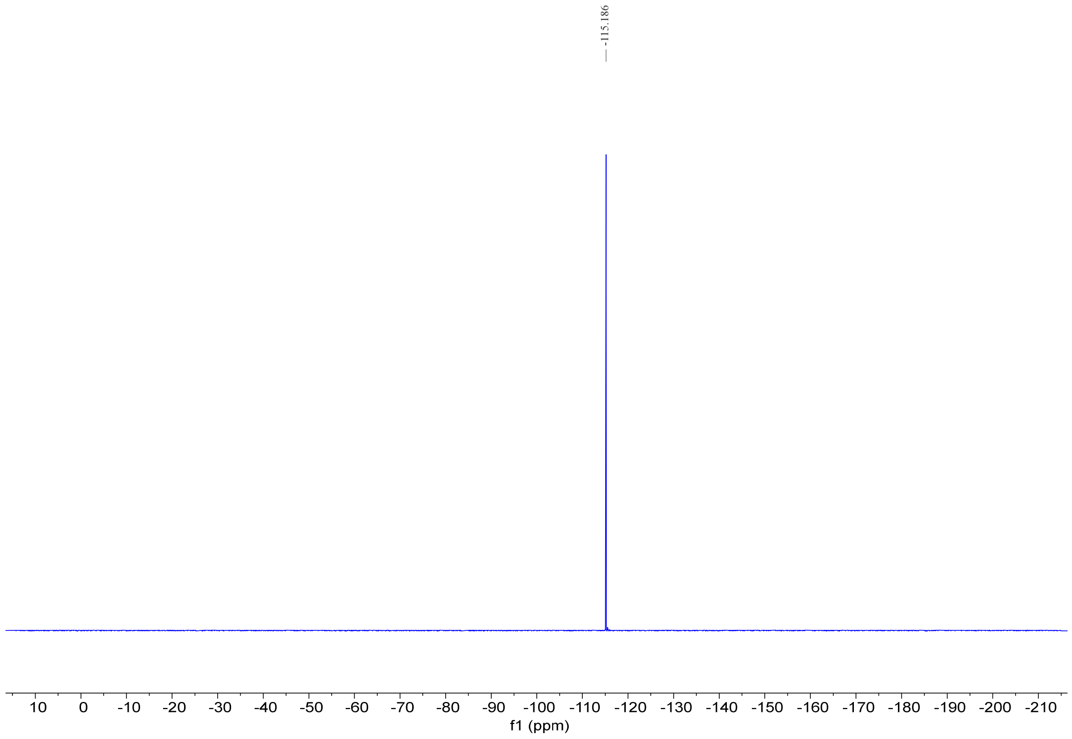

Supplementary Figure 149. ^{19}F NMR (565 MHz, $CDCl_3$) spectrum of compound **9**

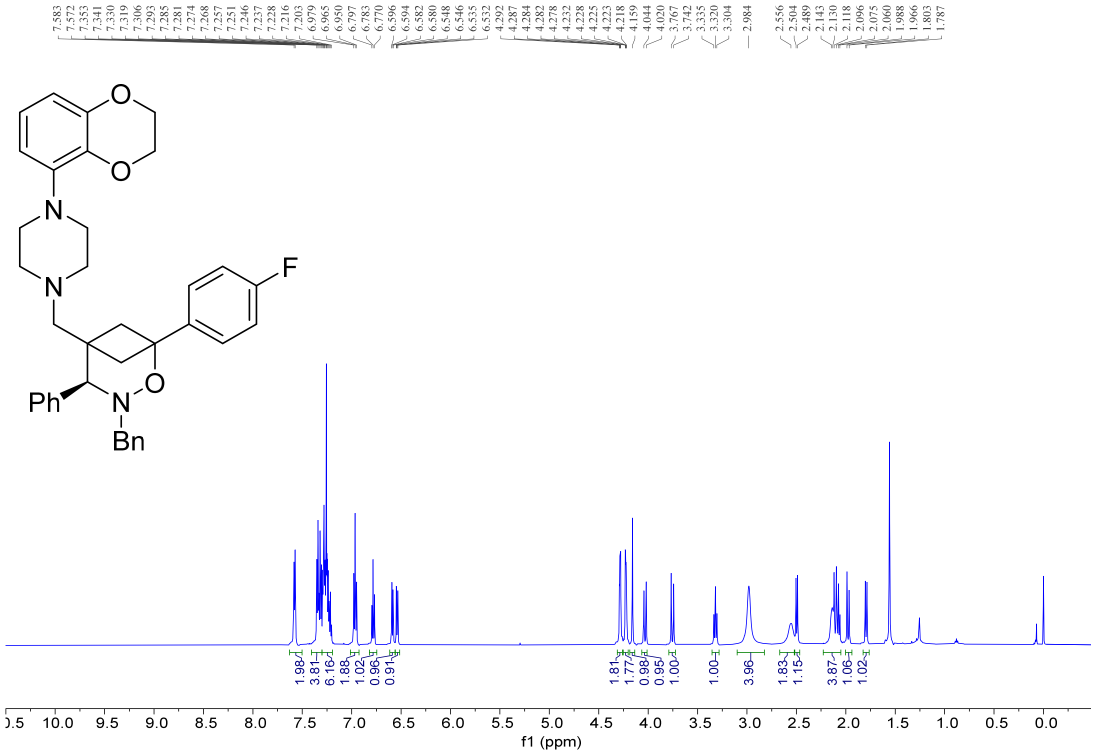

Supplementary Figure 150. ^{1}H NMR (600 MHz, $CDCl_3$) spectrum of compound **10**

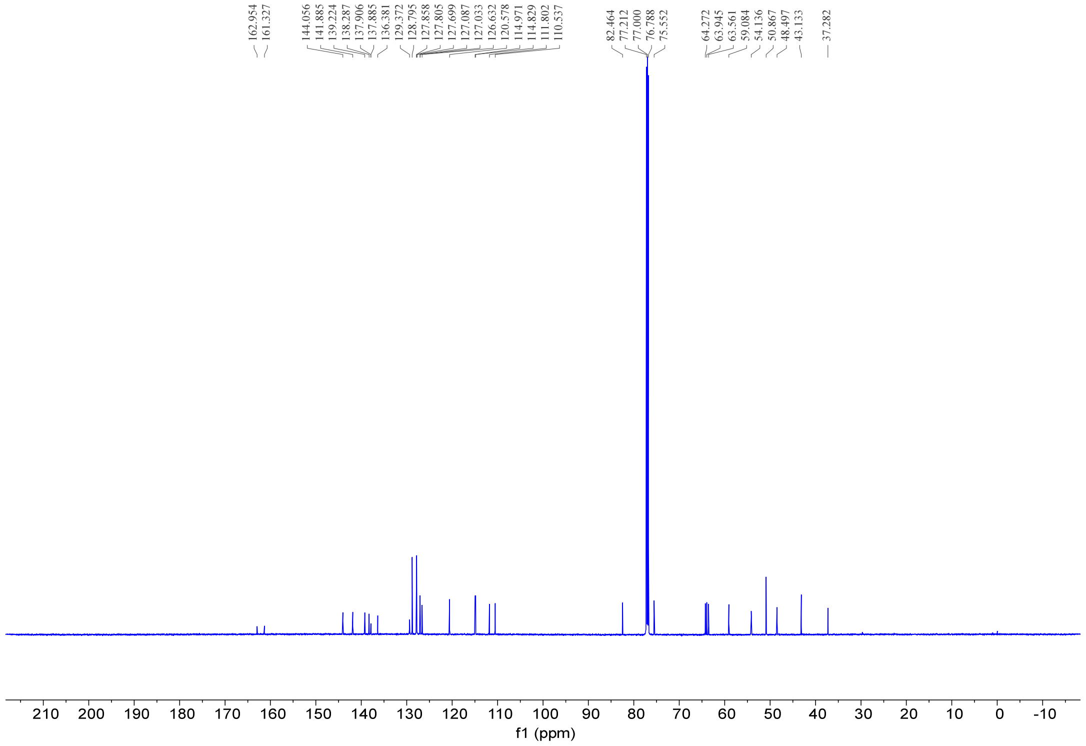

Supplementary Figure 151. ^{13}C NMR (150 MHz, $CDCl_3$) spectrum of compound **10**

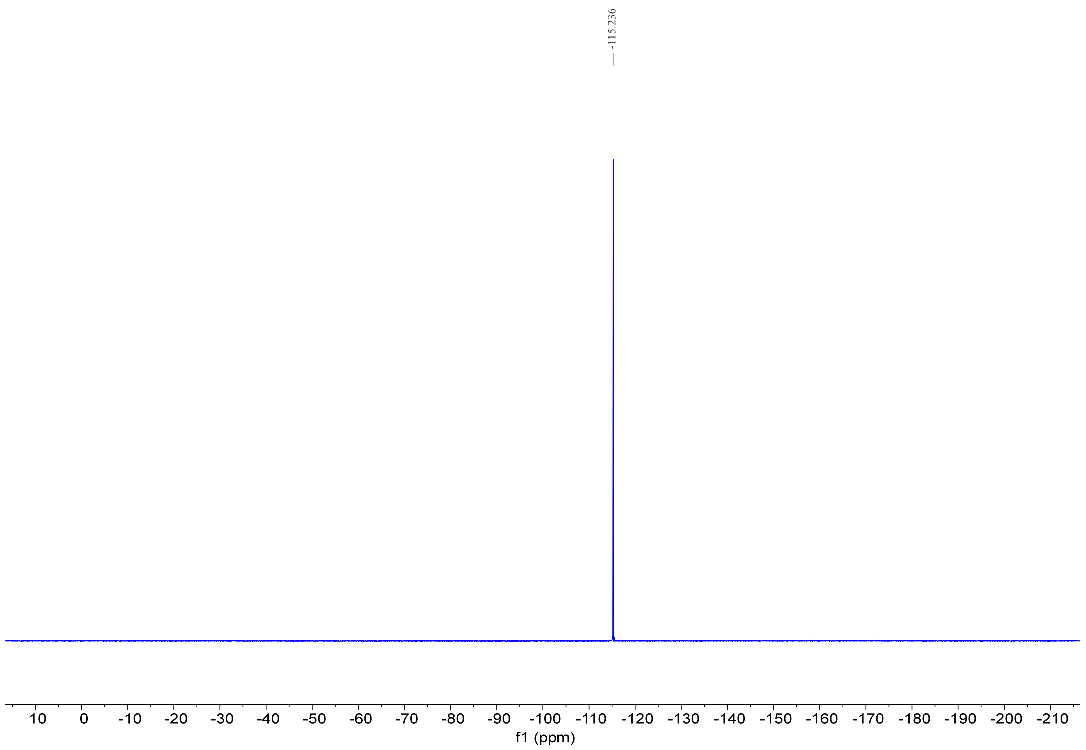

Supplementary Figure 152. ^{19}F NMR (565 MHz, $CDCl_3$) spectrum of compound **10**

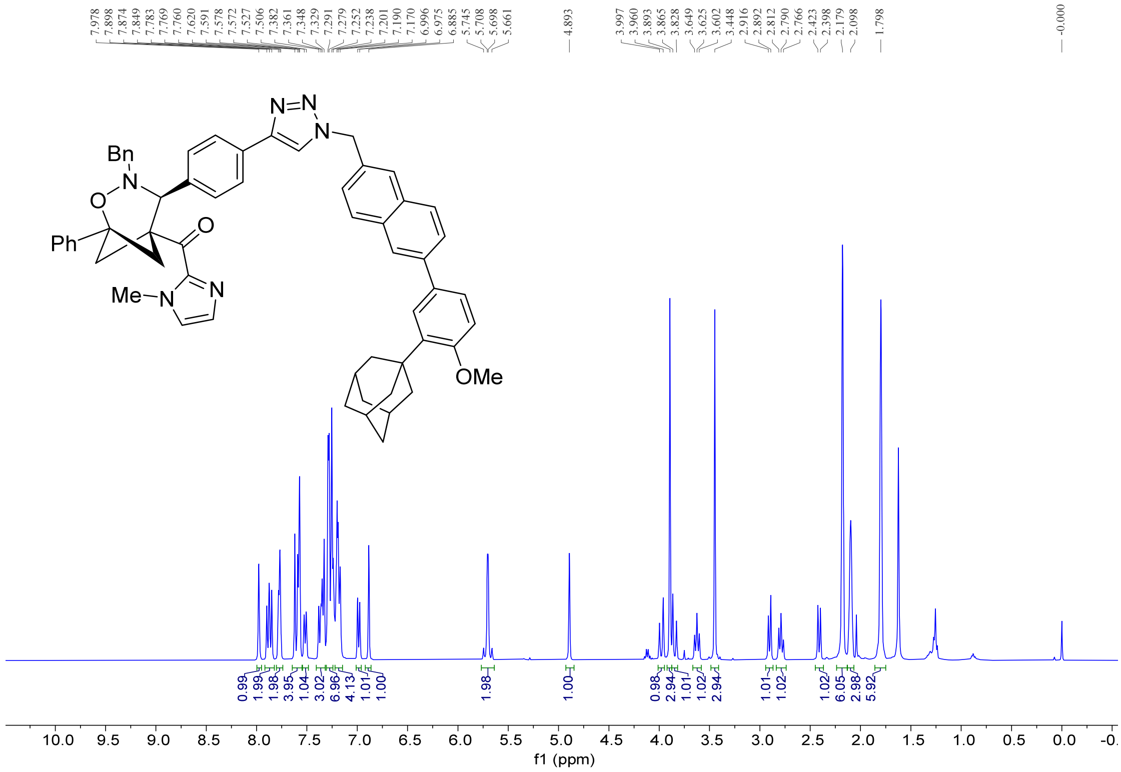

Supplementary Figure 153. ^{1}H NMR (600 MHz, $CDCl_3$) spectrum of compound **11**

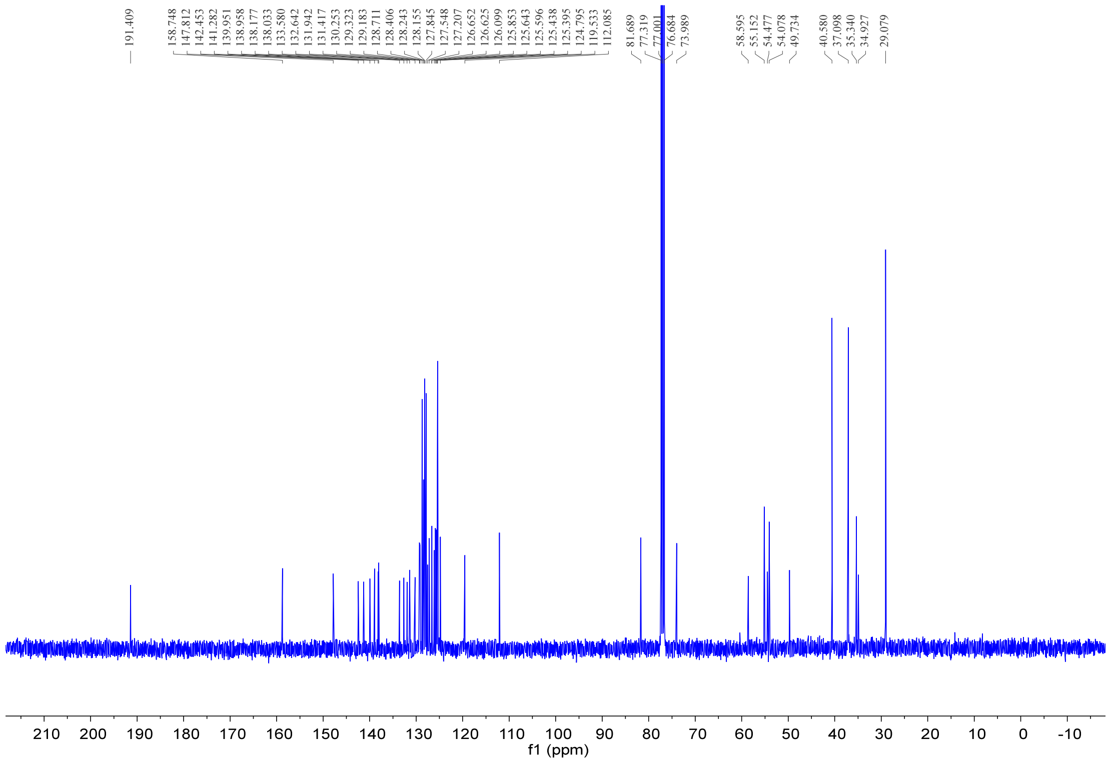

Supplementary Figure 154. ^{13}C NMR (150 MHz, $CDCl_3$) spectrum of compound **11**

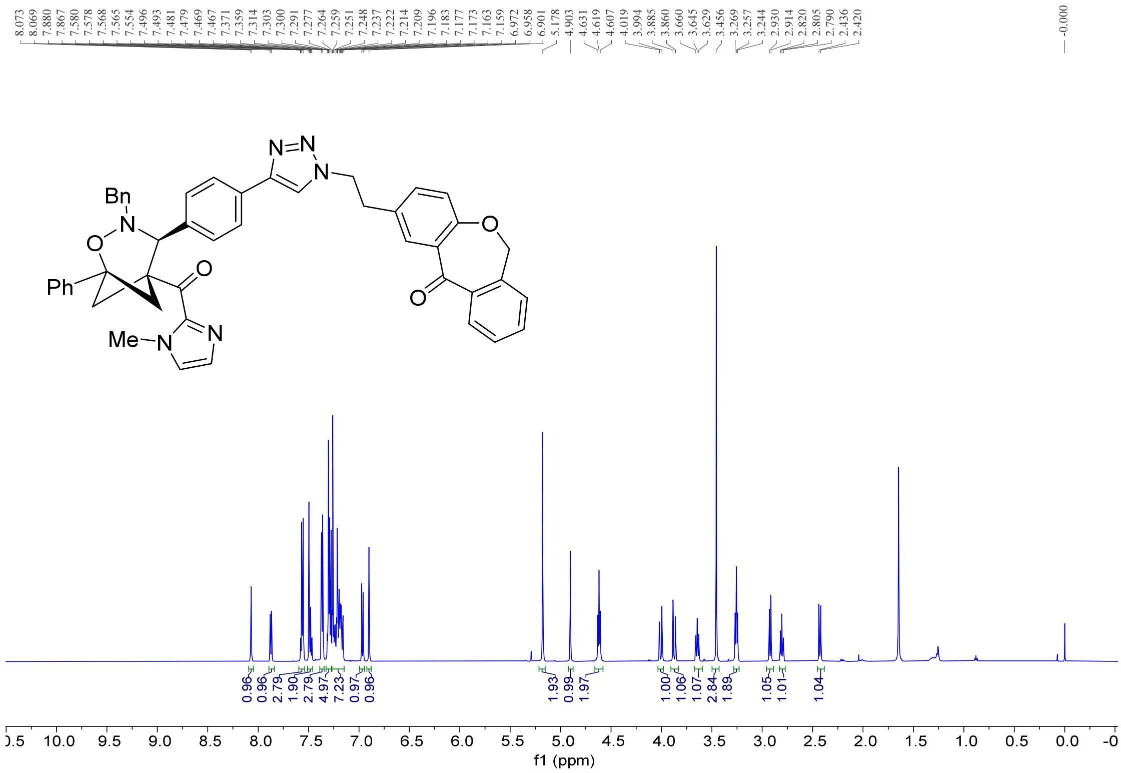

Supplementary Figure 155. ^{1}H NMR (600 MHz, $CDCl_3$) spectrum of compound **12**

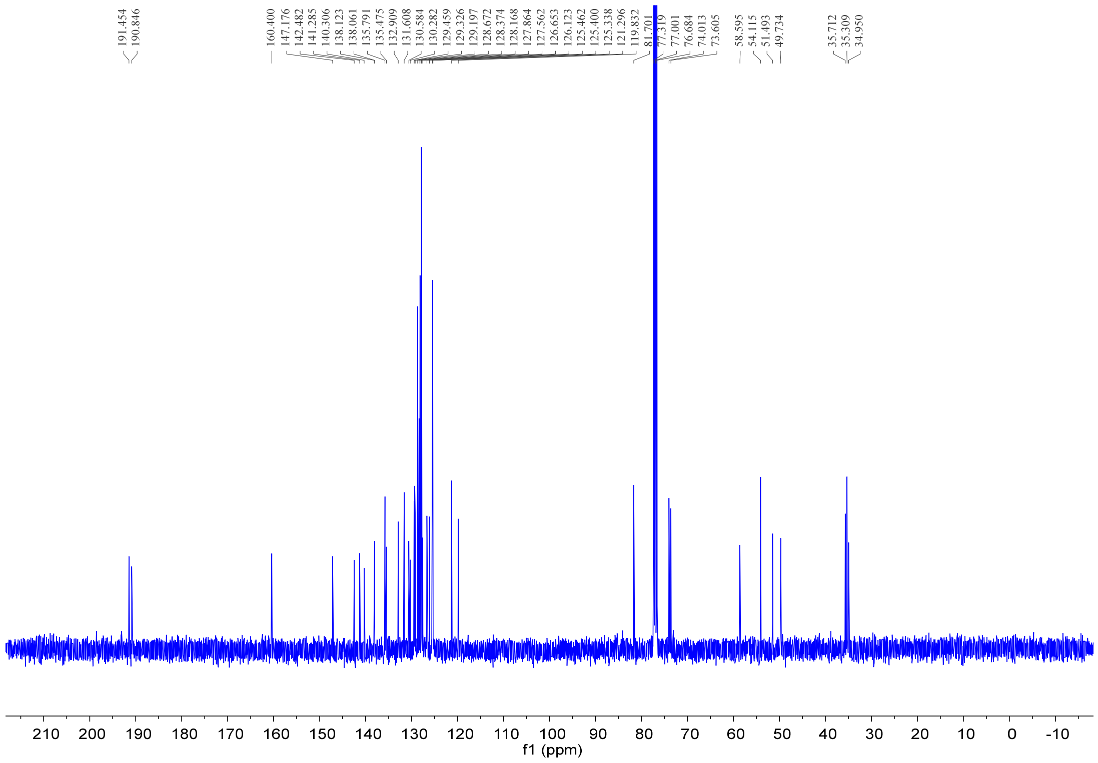

Supplementary Figure 156. ^{13}C NMR (100 MHz, $CDCl_3$) spectrum of compound **12**

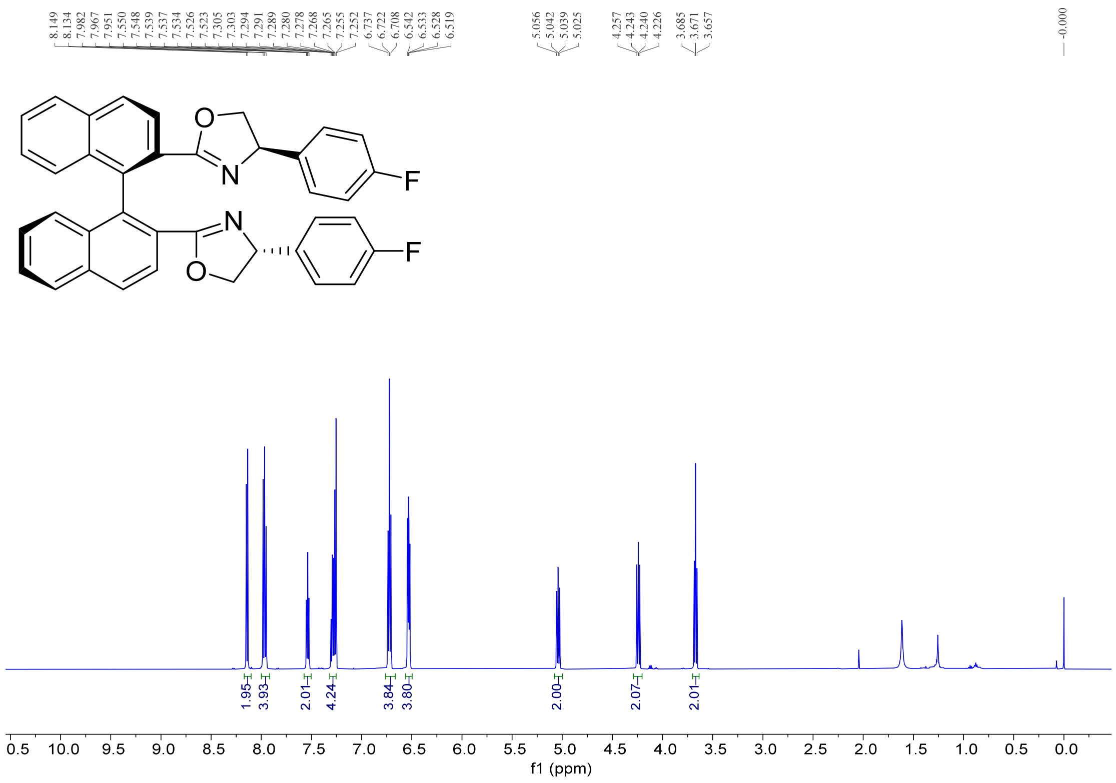

Supplementary Figure 157. ^{1}H NMR (600 MHz, $CDCl_3$) spectrum of compound **L21**

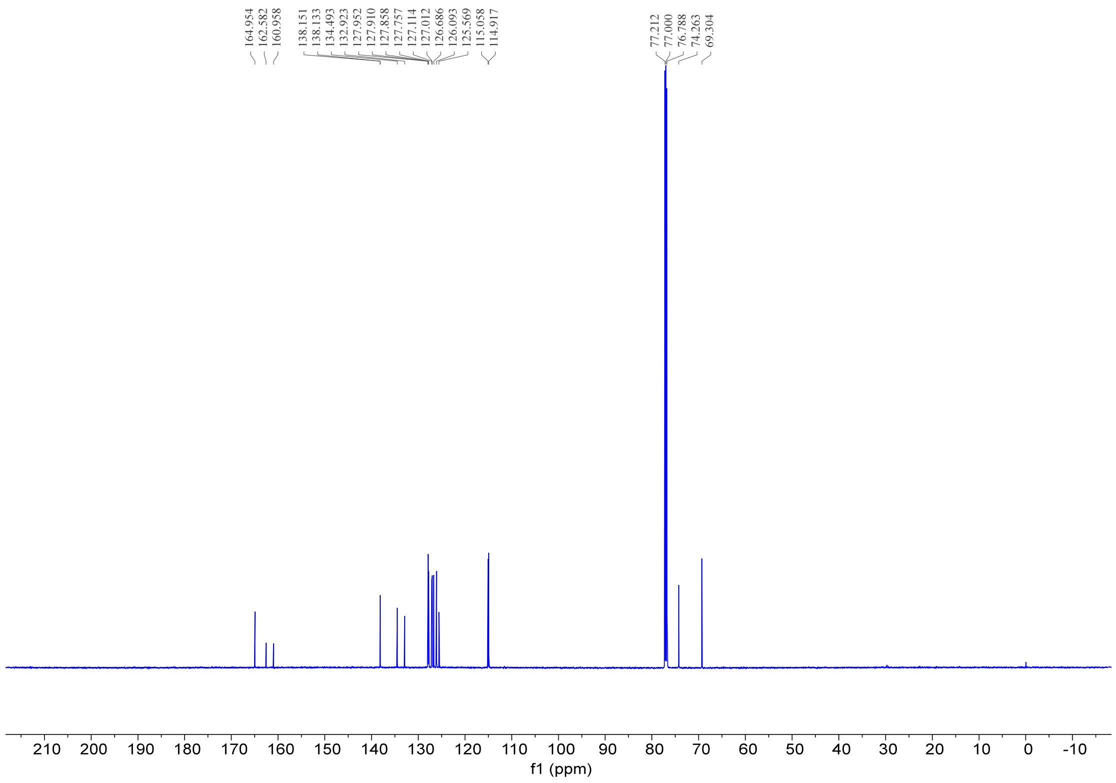

Supplementary Figure 158. ^{13}C NMR (150 MHz, $CDCl_3$) spectrum of compound **L21**

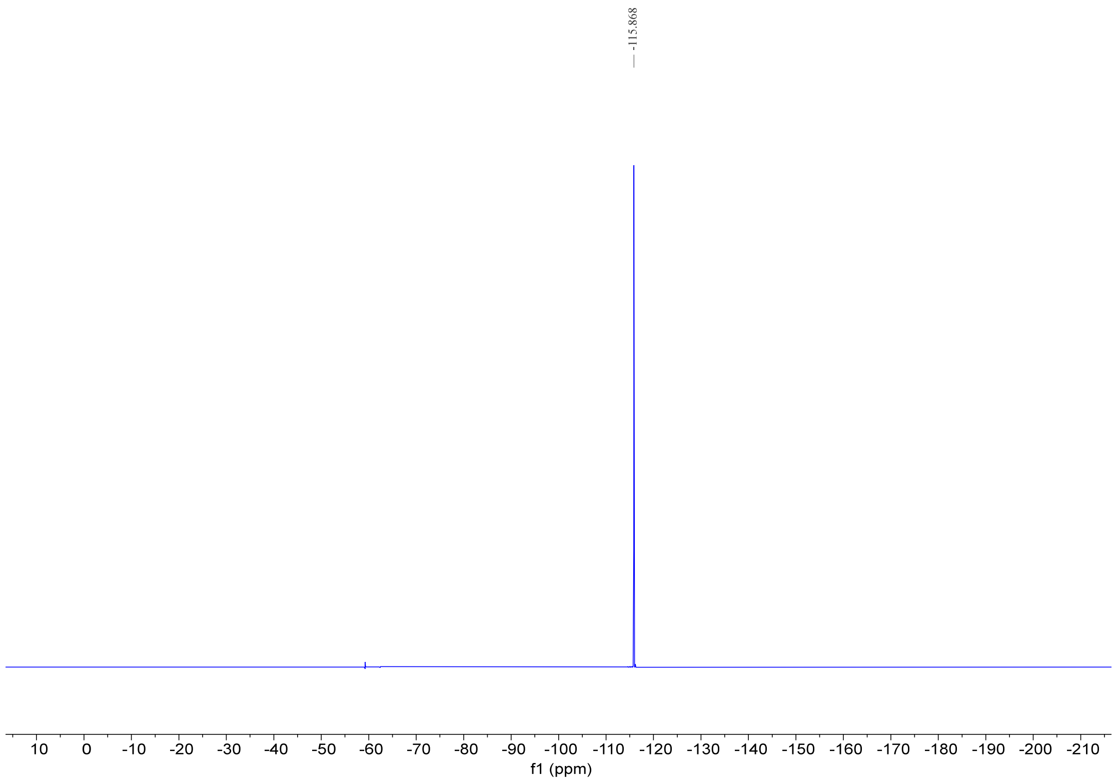

Supplementary Figure 159. ^{19}F NMR (565 MHz, $CDCl_3$) spectrum of compound **L21**

2024-03-19 11:21:33 1 / 1

Analysis Report

SHIMADZU LabSolutions

Sample Information

Sample Name	: WWB-1-52-RAC-ADH-9010-1.0-1
Sample ID	: WWB-1-52-RAC-ADH-9010-1.0-1
Data File	: WWB-1-52-RAC-ADH-9010-1.0-2.lcd
Method File	: 90-10.lcm
Report Format File	: DEFAULT.lsr
Date Acquired	: 2023/10/8 10:12:50
Date Processed	: 2023/10/8 10:32:29

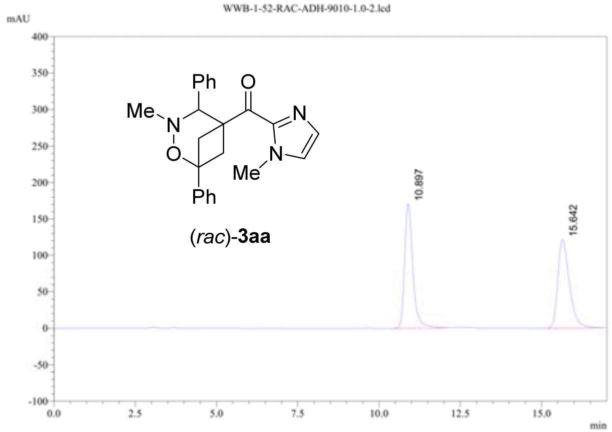

Detector A Channel 1 254nm

Peak#	Ret. Time [min]	Peak Start [min]	Height [mAU]	Area [mAU*min]	Area%
1	10.897	10.392	170784	3015242	50.071
2	15.642	14.983	121756	3006637	49.929
Total			292540	6021879	100.000

2024-03-19 11:21:10 1 / 1

Analysis Report

SHIMADZU LabSolutions

Sample Information

Sample Name	: YXC-2-11-ASY-ADH-9010-1.0-3
Sample ID	: YXC-2-11-ASY-ADH-9010-1.0-3
Data File	: YXC-2-11-ASY-ADH-9010-1.0-4.lcd
Method File	: 90-10.lcm
Report Format File	: DEFAULT.lsr
Date Acquired	: 2024/3/19 11:03:01
Date Processed	: 2024/3/19 11:20:40

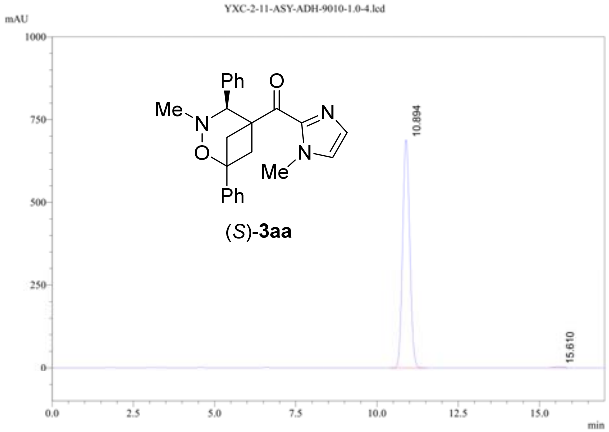

Detector A Channel 1 254nm

Peak#	Ret. Time [min]	Peak Start [min]	Height [mAU]	Area [mAU*min]	Area%
1	10.894	10.375	688858	10553294	99.433
2	15.610	15.333	3333	60216	0.567
Total			692191	10613509	100.000

Supplementary Figure 160. HPLC spectra of (*rac*)-**3aa** and (*S*)-**3aa**

2024-02-29 16:10:16 1 / 1

Analysis Report

SHIMADZU LabSolutions

Sample Information

Sample Name	: WWB-1-189-RAC-ADH-9010-1.0-1
Sample ID	: WWB-1-189-RAC-ADH-9010-1.0-1
Data File	: WWB-1-189-RAC-ADH-9010-1.0-2.lcd
Method File	: 90-10.lcm
Report Format File	: DEFAULT.lsr
Date Acquired	: 2024/2/29 15:43:43
Date Processed	: 2024/2/29 16:07:46

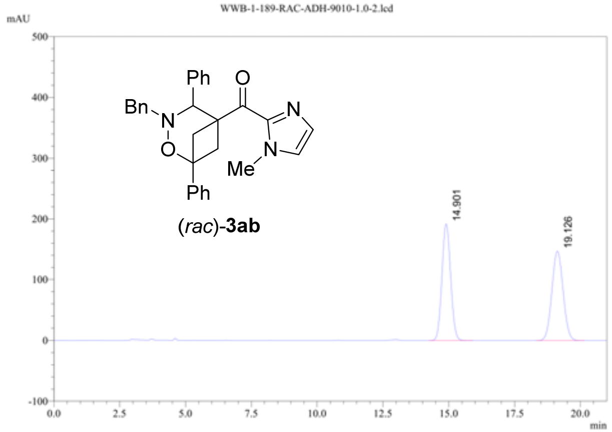

Detector A Channel 1 254nm

Peak#	Ret. Time [min]	Peak Start [min]	Height [mAU]	Area [mAU*min]	Area%
1	14.901	14.250	191912	4411046	50.191
2	19.126	18.325	147012	4377492	49.809
Total			338924	8788538	100.000

2024-02-29 16:09:55 1 / 1

Analysis Report

SHIMADZU LabSolutions

Sample Information

Sample Name	: WWB-1-189-2-ASY-ADH-9010-1.0-1
Sample ID	: WWB-1-189-2-ASY-ADH-9010-1.0-1
Data File	: WWB-1-189-2-ASY-ADH-9010-1.0-2.lcd
Method File	: 90-10.lcm
Report Format File	: DEFAULT.lsr
Date Acquired	: 2024/2/29 15:18:31
Date Processed	: 2024/2/29 16:09:08

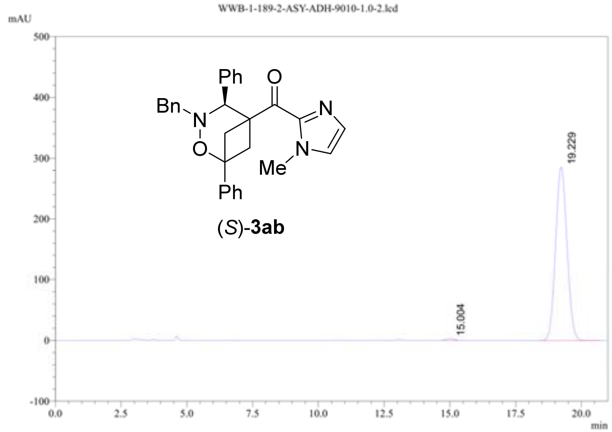

Detector A Channel 1 254nm

Peak#	Ret. Time [min]	Peak Start [min]	Height [mAU]	Area [mAU*min]	Area%
1	15.004	14.650	2999	62987	0.726
2	19.229	18.317	285544	8609355	99.274
Total			288543	8672342	100.000

Supplementary Figure 161. HPLC spectra of (*rac*)-**3ab** and (*S*)-**3ab**

2024-03-25 19:39:22 1 / 1

Analysis Report

SHIMADZU LabSolutions

Sample Information

Sample Name	: WWB-1-133-RAC-ADH-9505-1.0-1
Sample ID	: WWB-1-133-RAC-ADH-9505-1.0-1
Data File	: WWB-1-133-RAC-ADH-9505-1.0-2.lcd
Method File	: 90-10.lcm
Report Format File	: DEFAULT.lsr
Date Acquired	: 2023/12/27 17:12:50
Date Processed	: 2024/3/25 19:29:13

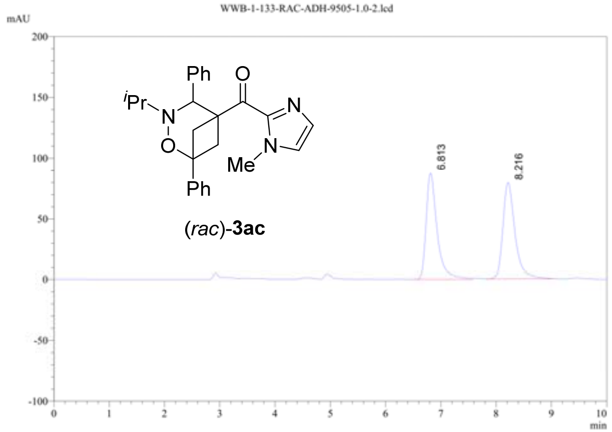

Detector A Channel 1 254nm

Peak#	Ret. Time [min]	Peak Start [min]	Height [mAU]	Area [mAU*min]	Area%
1	6.813	6.492	87700	1215130	50.064
2	8.216	7.817	79577	1212012	49.936
Total			167277	2427142	100.000

2024-03-25 19:40:30 1 / 1

Analysis Report

SHIMADZU LabSolutions

Sample Information

Sample Name	: YXC-2-19-ASY-ADH-9505-1.0-1
Sample ID	: YXC-2-19-ASY-ADH-9505-1.0-1
Data File	: YXC-2-19-ASY-ADH-9505-1.0-2.lcd
Method File	: 90-10.lcm
Report Format File	: DEFAULT.lsr
Date Acquired	: 2024/3/25 19:26:25
Date Processed	: 2024/3/25 19:40:10

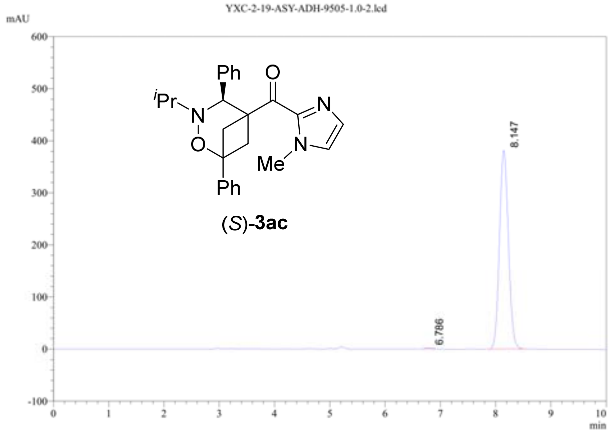

Detector A Channel 1 254nm

Peak#	Ret. Time [min]	Peak Start [min]	Height [mAU]	Area [mAU*min]	Area%
1	6.786	6.683	2030	15116	0.352
2	8.147	7.883	381725	4274411	99.648
Total			383755	4289527	100.000

Supplementary Figure 162. HPLC spectra of (*rac*)-**3ac** and (*S*)-**3ac**

2024-03-19 10:42:32 1 / 1

Analysis Report

SHIMADZU LabSolutions

Sample Information

Sample Name	: YXC-2-10-RAC-ADH-9010-1.0-3
Sample ID	: YXC-2-10-RAC-ADH-9010-1.0-3
Data File	: YXC-2-10-RAC-ADH-9010-1.0-4.lcd
Method File	: 90-10.lcm
Report Format File	: DEFAULT.lsr
Date Acquired	: 2024/3/19 10:08:44
Date Processed	: 2024/3/19 10:38:08

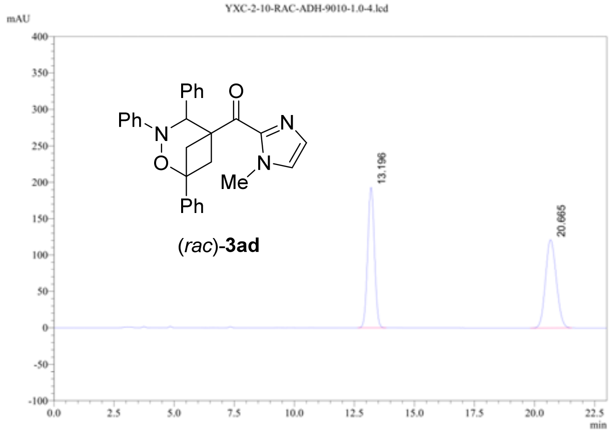

Detector A Channel 1 254nm

Peak#	Ret. Time [min]	Peak Start [min]	Height [mAU]	Area [mAU*min]	Area%
1	13.196	12.642	192865	3787653	49.943
2	20.665	19.825	121229	3796231	50.057
Total			314094	7583884	100.000

2024-03-20 10:08:07 1 / 1

Analysis Report

SHIMADZU LabSolutions

Sample Information

Sample Name	: WWB-1-195-ASY-ADH-9010-1.0-1
Sample ID	: WWB-1-195-ASY-ADH-9010-1.0-1
Data File	: WWB-1-195-ASY-ADH-9010-1.0-2.lcd
Method File	: 90-10.lcm
Report Format File	: DEFAULT.lsr
Date Acquired	: 2024/3/20 9:43:58
Date Processed	: 2024/3/20 10:07:20

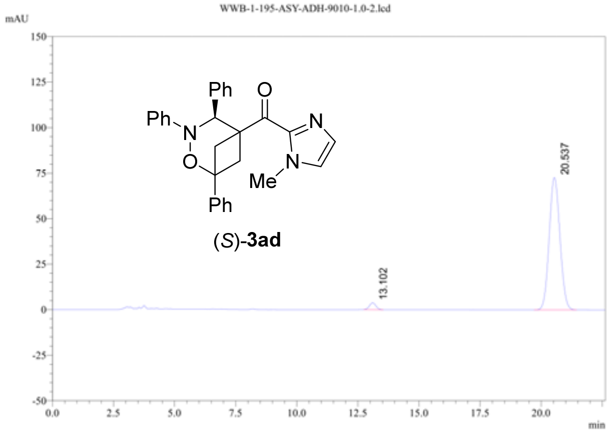

Detector A Channel 1 254nm

Peak#	Ret. Time [min]	Peak Start [min]	Height [mAU]	Area [mAU*min]	Area%
1	13.102	12.733	3707	68303	2.977
2	20.537	19.708	72778	2226030	97.023
Total			76485	2294333	100.000

Supplementary Figure 163. HPLC spectra of (*rac*)-**3ad** and (*S*)-**3ad**

2024-03-14 10:36:44 1 / 1

Analysis Report

SHIMADZU LabSolutions

Sample Information

Sample Name	: YXC-1-195-RAC-ADH-8515-1.0-1
Sample ID	: YXC-1-195-RAC-ADH-8515-1.0-1
Data File	: YXC-1-195-RAC-ADH-8515-1.0-2.lcd
Method File	: 90-10.lcm
Report Format File	: DEFAULT.lsr
Date Acquired	: 2024/3/14 10:20:15
Date Processed	: 2024/3/14 10:34:04

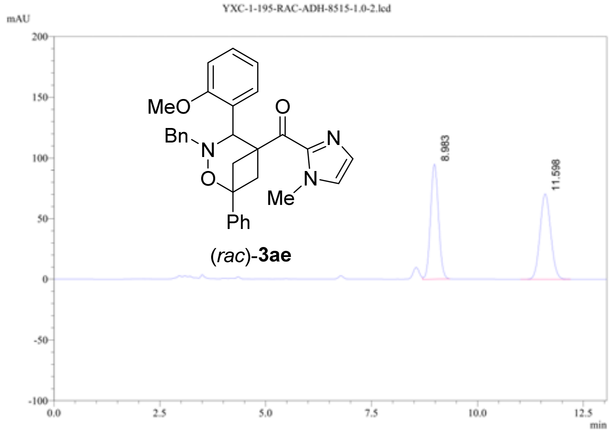

Detector A Channel 1 254nm

Peak#	Ret. Time [min]	Peak Start [min]	Height [mAU]	Area [mAU*min]	Area%
1	8.983	8.700	95065	1247877	50.084
2	11.598	11.017	70505	1243670	49.916
Total			165570	2491547	100.000

2024-03-14 10:51:49 1 / 1

Analysis Report

SHIMADZU LabSolutions

Sample Information

Sample Name	: YXC-1-195-ASY-ADH-8515-1.0-1
Sample ID	: YXC-1-195-ASY-ADH-8515-1.0-1
Data File	: YXC-1-195-ASY-ADH-8515-1.0-2.lcd
Method File	: 90-10.lcm
Report Format File	: DEFAULT.lsr
Date Acquired	: 2024/3/14 10:34:59
Date Processed	: 2024/3/14 10:51:26

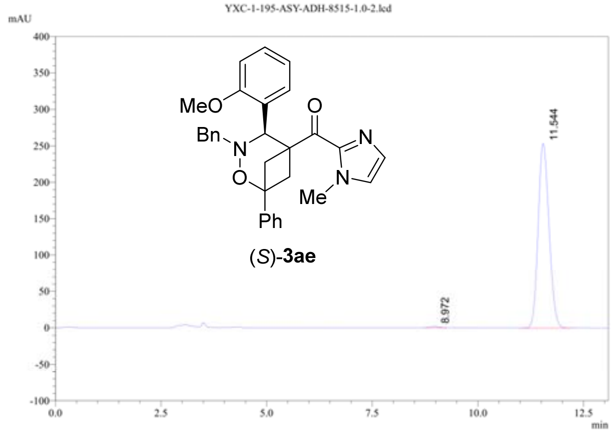

Detector A Channel 1 254nm

Peak#	Ret. Time [min]	Peak Start [min]	Height [mAU]	Area [mAU*min]	Area%
1	8.972	8.717	2025	26632	0.584
2	11.544	10.975	253743	4537244	99.416
Total			255767	4563875	100.000

Supplementary Figure 164. HPLC spectra of (*rac*)-**3ae** and (*S*)-**3ae**

2024-03-14 17:27:47 1 / 1

Analysis Report

SHIMADZU LabSolutions

Sample Information

Sample Name	: YXC-1-196-RAC-ADH-8515-0.8-1
Sample ID	: YXC-1-196-RAC-ADH-8515-0.8-1
Data File	: YXC-1-196-RAC-ADH-8515-0.8-2.lcd
Method File	: 90-10.lcm
Report Format File	: DEFAULT.lsr
Date Acquired	: 2024/3/14 16:55:47
Date Processed	: 2024/3/14 17:14:21

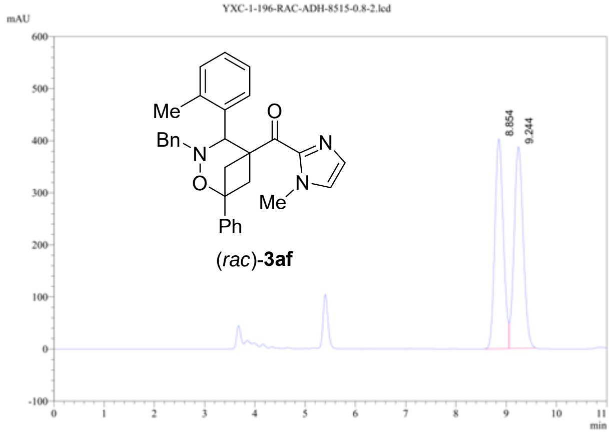

Detector A Channel 1 254nm

Peak#	Ret. Time [min]	Peak Start [min]	Height [mAU]	Area [mAU*min]	Area%
1	8.854	8.558	402694	4768315	49.462
2	9.244	9.058	387074	4872079	50.538
Total			789768	9640394	100.000

2024-03-14 17:27:58 1 / 1

Analysis Report

SHIMADZU LabSolutions

Sample Information

Sample Name	: YXC-1-196-ASY-ADH-8515-0.8-1
Sample ID	: YXC-1-196-ASY-ADH-8515-0.8-1
Data File	: YXC-1-196-ASY-ADH-8515-0.8-2.lcd
Method File	: 90-10.lcm
Report Format File	: DEFAULT.lsr
Date Acquired	: 2024/3/14 17:15:02
Date Processed	: 2024/3/14 17:27:17

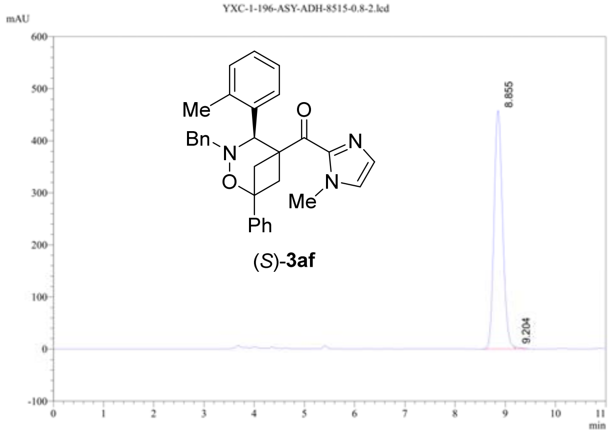

Detector A Channel 1 254nm

Peak#	Ret. Time [min]	Peak Start [min]	Height [mAU]	Area [mAU*min]	Area%
1	8.855	8.525	457725	5447201	99.500
2	9.204	9.192	4137	27348	0.500
Total			461861	5474549	100.000

Supplementary Figure 165. HPLC spectra of (*rac*)-**3af** and (*S*)-**3af**

2024-03-14 16:30:29 1 / 1

Analysis Report

SHIMADZU LabSolutions

Sample Information

Sample Name	: YXC-1-192-RAC-ADH-8515-0.8-1
Sample ID	: YXC-1-192-RAC-ADH-8515-0.8-1
Data File	: YXC-1-192-RAC-ADH-8515-0.8-2.lcd
Method File	: 90-10.lcm
Report Format File	: DEFAULT.lsr
Date Acquired	: 2024/3/14 16:03:24
Date Processed	: 2024/3/14 16:27:32

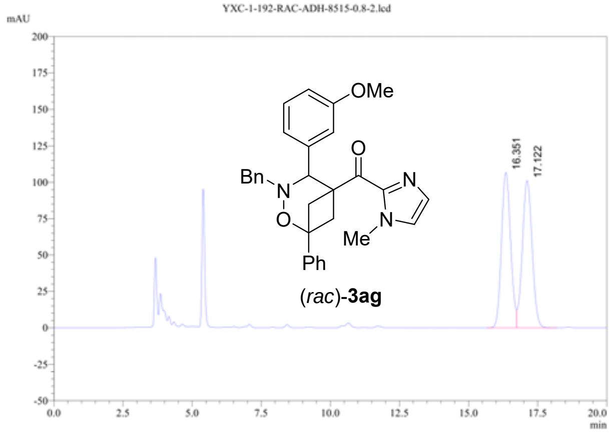

Detector A Channel 1 254nm

Peak#	Ret. Time [min]	Peak Start [min]	Height [mAU]	Area [mAU*min]	Area%
1	16.351	15.658	106713	2456846	49.695
2	17.122	16.742	101089	2486953	50.305
Total			207802	4943799	100.000

2024-04-16 15:48:02 1 / 1

Analysis Report

SHIMADZU LabSolutions

Sample Information

Sample Name	: YXC-1-192-ASY-ADH-8515-0.8-1
Sample ID	: YXC-1-192-ASY-ADH-8515-0.8-1
Data File	: YXC-1-192-ASY-ADH-8515-0.8-2.lcd
Method File	: 90-10.lcm
Report Format File	: DEFAULT.lsr
Date Acquired	: 2024/3/14 16:28:31
Date Processed	: 2024/3/14 16:54:40

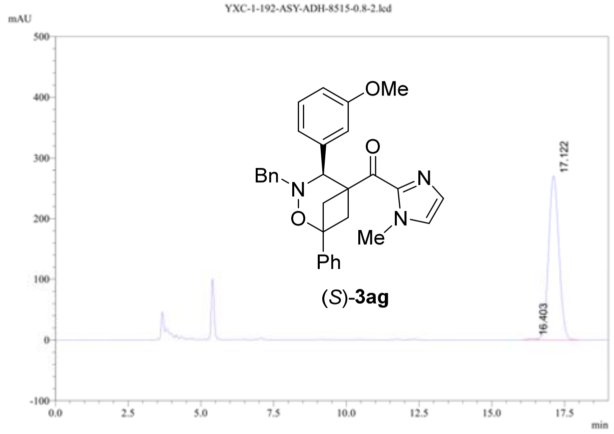

Detector A Channel 1 254nm

Peak#	Ret. Time [min]	Peak Start [min]	Height [mAU]	Area [mAU*min]	Area%
1	16.403	15.958	2524	50867	0.757
2	17.122	16.567	270544	6665730	99.243
Total			273067	6716597	100.000

Supplementary Figure 166. HPLC spectra of (*rac*)-**3ag** and (*S*)-**3ag**

2024-03-16 09:52:18 1 / 1

Analysis Report

SHIMADZU LabSolutions

Sample Information

Sample Name	: YXC-1-191-RAC-IC-9010-0.8-1
Sample ID	: YXC-1-191-RAC-IC-9010-0.8-1
Data File	: YXC-1-191-RAC-IC-9010-0.8-2.lcd
Method File	: 90-10.lcm
Report Format File	: DEFAULT.lsr
Date Acquired	: 2024/3/16 9:14:57
Date Processed	: 2024/3/16 9:51:46

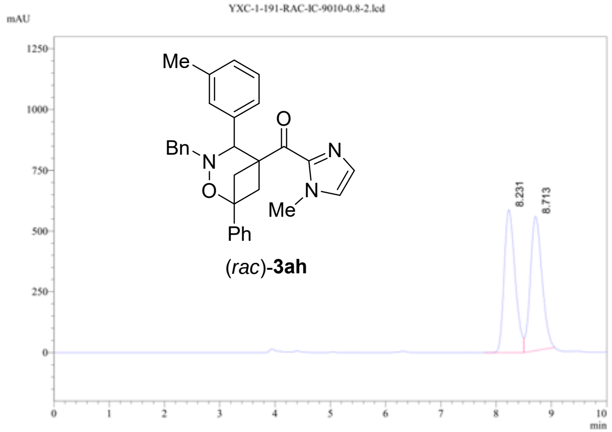

Detector A Channel 1 254nm

Peak#	Ret. Time [min]	Peak Start [min]	Height [mAU]	Area [mAU*min]	Area%
1	8.231	7.783	588033	8088358	49.951
2	8.713	8.500	551916	8104251	50.049
Total			1139950	16192609	100.000

2024-03-16 09:50:25 1 / 1

Analysis Report

SHIMADZU LabSolutions

Sample Information

Sample Name	: YXC-1-191-ASY-IC-9010-0.8-1
Sample ID	: YXC-1-191-ASY-IC-9010-0.8-1
Data File	: YXC-1-191-ASY-IC-9010-0.8-2.lcd
Method File	: 90-10.lcm
Report Format File	: DEFAULT.lsr
Date Acquired	: 2024/3/16 9:32:22
Date Processed	: 2024/3/16 9:47:16

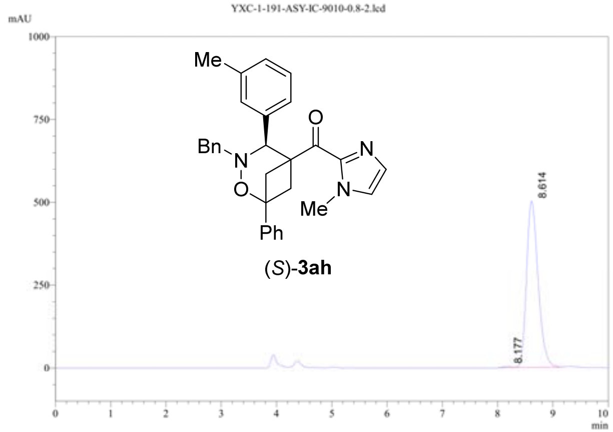

Detector A Channel 1 254nm

Peak#	Ret. Time [min]	Peak Start [min]	Height [mAU]	Area [mAU*min]	Area%
1	8.177	8.008	3983	43181	0.591
2	8.614	8.325	501617	7265326	99.409
Total			505599	7308507	100.000

Supplementary Figure 167. HPLC spectra of (*rac*)-**3ah** and (*S*)-**3ah**

2024-03-25 20:50:28 1 / 1

Analysis Report

SHIMADZU LabSolutions

Sample Information

Sample Name	: YXC-2-15-RAC-ADH-8515-1.0-1
Sample ID	: YXC-2-15-RAC-ADH-8515-1.0-1
Data File	: YXC-2-15-RAC-ADH-8515-1.0-2.lcd
Method File	: 90-10.lcm
Report Format File	: DEFAULT.lsr
Date Acquired	: 2024/3/25 20:01:59
Date Processed	: 2024/3/25 20:28:09

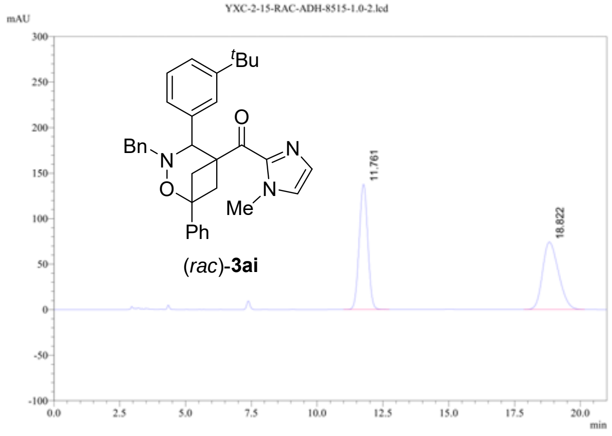

Detector A Channel 1 254nm

Peak#	Ret. Time [min]	Peak Start [min]	Height [mAU]	Area [mAU*min]	Area%
1	11.761	11.000	137571	3114842	49.981
2	18.822	17.850	74020	3117169	50.019
Total			211591	6232010	100.000

2024-03-25 20:51:37 1 / 1

Analysis Report

SHIMADZU LabSolutions

Sample Information

Sample Name	: YXC-2-15-ASY-ADH-8515-1.0-1
Sample ID	: YXC-2-15-ASY-ADH-8515-1.0-1
Data File	: YXC-2-15-ASY-ADH-8515-1.0-2.lcd
Method File	: 90-10.lcm
Report Format File	: DEFAULT.lsr
Date Acquired	: 2024/3/25 20:29:08
Date Processed	: 2024/3/25 20:51:10

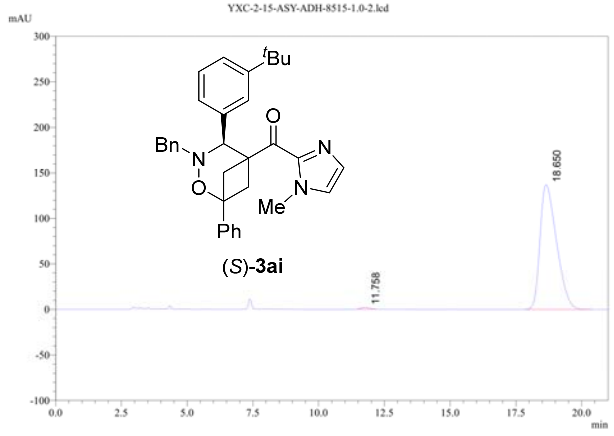

Detector A Channel 1 254nm

Peak#	Ret. Time [min]	Peak Start [min]	Height [mAU]	Area [mAU*min]	Area%
1	11.758	11.433	1993	41221	0.683
2	18.650	17.842	137169	5990055	99.317
Total			139162	6031276	100.000

Supplementary Figure 168. HPLC spectra of (*rac*)-**3ai** and (*S*)-**3ai**

2024-03-14 11:06:32 1 / 1

Analysis Report

SHIMADZU LabSolutions

Sample Information

Sample Name	: YXC-1-197-RAC-ADH-8515-1.0-1
Sample ID	: YXC-1-197-RAC-ADH-8515-1.0-1
Data File	: YXC-1-197-RAC-ADH-8515-1.0-2.lcd
Method File	: 90-10.lcm
Report Format File	: DEFAULT.lsr
Date Acquired	: 2024/3/14 10:03:12
Date Processed	: 2024/3/14 10:19:28

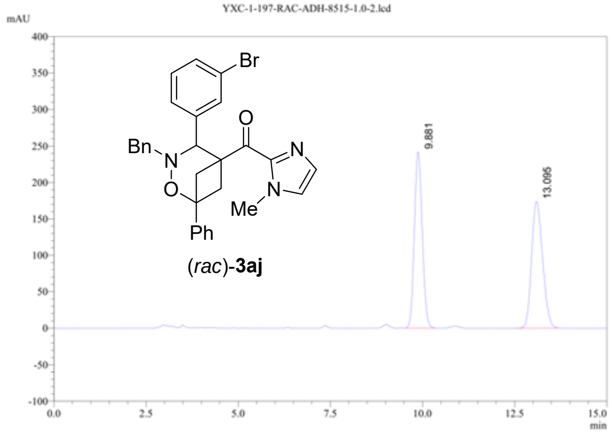

Detector A Channel 1 254nm

Peak#	Ret. Time [min]	Peak Start [min]	Height [mAU]	Area [mAU*min]	Area%
1	9.881	9.467	241857	3589956	49.921
2	13.095	12.525	173776	3601361	50.079
Total			415634	7191317	100.000

2024-03-14 11:06:52 1 / 1

Analysis Report

SHIMADZU LabSolutions

Sample Information

Sample Name	: YXC-1-197-ASY-ADH-8515-1.0-1
Sample ID	: YXC-1-197-ASY-ADH-8515-1.0-1
Data File	: YXC-1-197-ASY-ADH-8515-1.0-2.lcd
Method File	: 90-10.lcm
Report Format File	: DEFAULT.lsr
Date Acquired	: 2024/3/14 10:48:59
Date Processed	: 2024/3/14 11:05:58

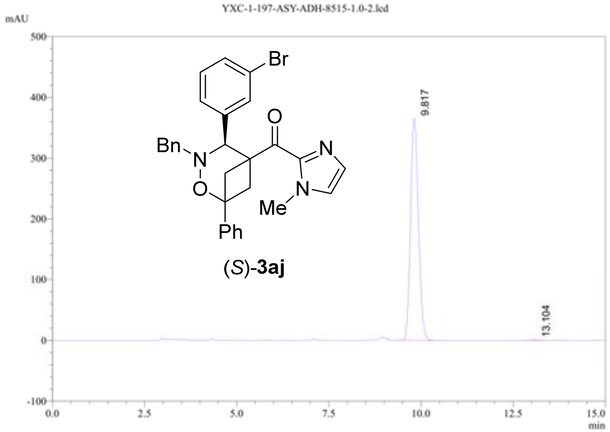

Detector A Channel 1 254nm

Peak#	Ret. Time [min]	Peak Start [min]	Height [mAU]	Area [mAU*min]	Area%
1	9.817	9.425	364823	5429670	99.812
2	13.104	12.867	671	10221	0.188
Total			365494	5439891	100.000

Supplementary Figure 169. HPLC spectra of (*rac*)-**3aj** and (*S*)-**3aj**

2024-03-13 19:11:35 1 / 1

Analysis Report

SHIMADZU LabSolutions

Sample Information

Sample Name	: YXC-1-193-RAC-ADH-8515-1.0-1
Sample ID	: YXC-1-193-RAC-ADH-8515-1.0-1
Data File	: YXC-1-193-RAC-ADH-8515-1.0-2.lcd
Method File	: 90-10.lcm
Report Format File	: DEFAULT.lsr
Date Acquired	: 2024/3/13 18:47:54
Date Processed	: 2024/3/13 19:08:11

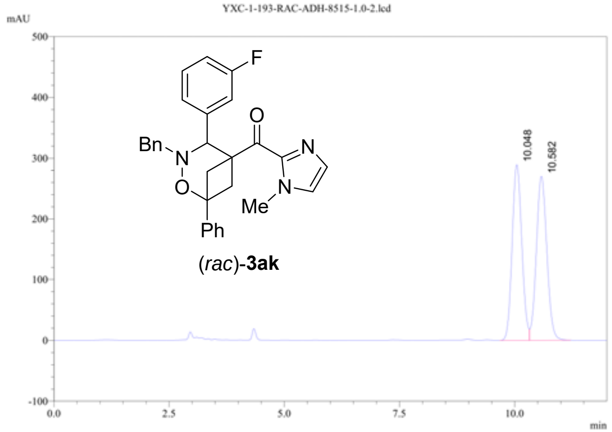

Detector A Channel 1 254nm

Peak#	Ret. Time [min]	Peak Start [min]	Height [mAU]	Area [mAU*min]	Area%
1	10.048	9.675	289500	4227893	49.724
2	10.582	10.325	270082	4274824	50.276
Total			559582	8502717	100.000

2024-03-13 19:25:30 1 / 1

Analysis Report

SHIMADZU LabSolutions

Sample Information

Sample Name	: YXC-1-193-ASY-ADH-8515-1.0-1
Sample ID	: YXC-1-193-ASY-ADH-8515-1.0-1
Data File	: YXC-1-193-ASY-ADH-8515-1.0-2.lcd
Method File	: 90-10.lcm
Report Format File	: DEFAULT.lsr
Date Acquired	: 2024/3/13 19:09:17
Date Processed	: 2024/3/13 19:22:48

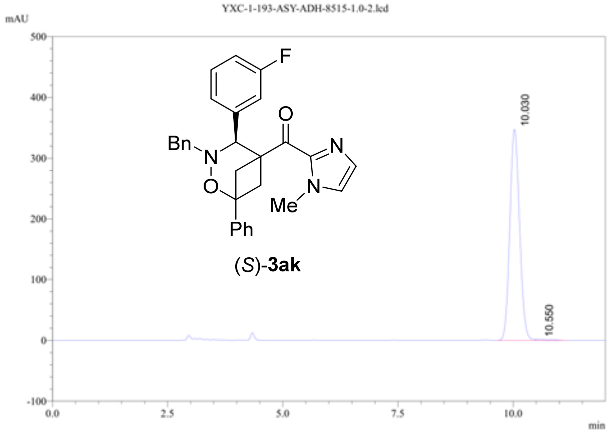

Detector A Channel 1 254nm

Peak#	Ret. Time [min]	Peak Start [min]	Height [mAU]	Area [mAU*min]	Area%
1	10.030	9.650	347617	5106310	99.327
2	10.550	10.492	1602	34591	0.673
Total			349219	5140901	100.000

Supplementary Figure 170. HPLC spectra of (*rac*)-**3ak** and (*S*)-**3ak**

2024-03-08 14:53:15 1 / 1

Analysis Report

SHIMADZU LabSolutions

Sample Information

Sample Name	: YXC-1-182-RAC-ADH-8515-1.0-1
Sample ID	: YXC-1-182-RAC-ADH-8515-1.0-1
Data File	: YXC-1-182-RAC-ADH-8515-1.0-2.lcd
Method File	: 90-10.lcm
Report Format File	: DEFAULT.lsr
Date Acquired	: 2024/3/8 14:29:32
Date Processed	: 2024/3/8 14:50:37

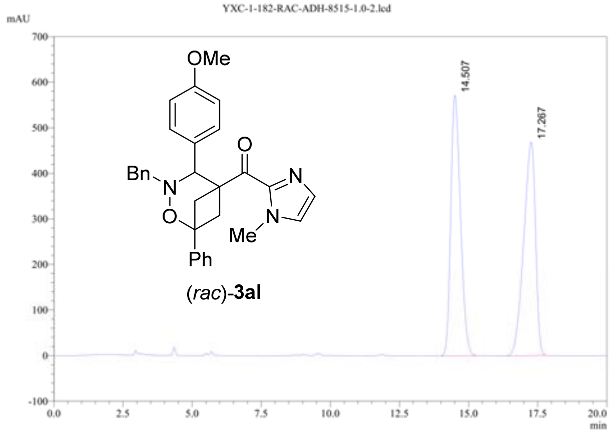

Detector A Channel 1 254nm

Peak#	Ret. Time [min]	Peak Start [min]	Height [mAU]	Area [mAU*min]	Area%
1	14.507	13.975	571643	13895029	50.013
2	17.267	16.292	468627	13887924	49.987
Total			1040270	27782953	100.000

2024-03-08 15:14:25 1 / 1

Analysis Report

SHIMADZU LabSolutions

Sample Information

Sample Name	: YXC-1-182-ASY-ADH-8515-1.0-1
Sample ID	: YXC-1-182-ASY-ADH-8515-1.0-1
Data File	: YXC-1-182-ASY-ADH-8515-1.0-2.lcd
Method File	: 90-10.lcm
Report Format File	: DEFAULT.lsr
Date Acquired	: 2024/3/8 14:51:28
Date Processed	: 2024/3/8 15:14:05

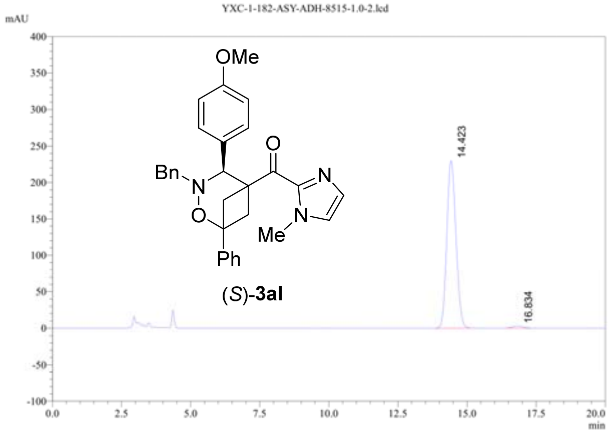

Detector A Channel 1 254nm

Peak#	Ret. Time [min]	Peak Start [min]	Height [mAU]	Area [mAU*min]	Area%
1	14.423	13.833	230016	5278181	98.628
2	16.834	16.433	2972	73427	1.372
Total			232988	5351608	100.000

Supplementary Figure 171. HPLC spectra of (*rac*)-**3al** and (*S*)-**3al**

2024-03-08 15:40:34 1 / 1

Analysis Report

SHIMADZU LabSolutions

Sample Information

Sample Name	: YXC-1-183-RAC-ADH-8515-1.0-1
Sample ID	: YXC-1-183-RAC-ADH-8515-1.0-1
Data File	: YXC-1-183-RAC-ADH-8515-1.0-2.lcd
Method File	: 90-10.lcm
Report Format File	: DEFAULT.lsr
Date Acquired	: 2024/3/8 15:16:00
Date Processed	: 2024/3/8 15:37:43

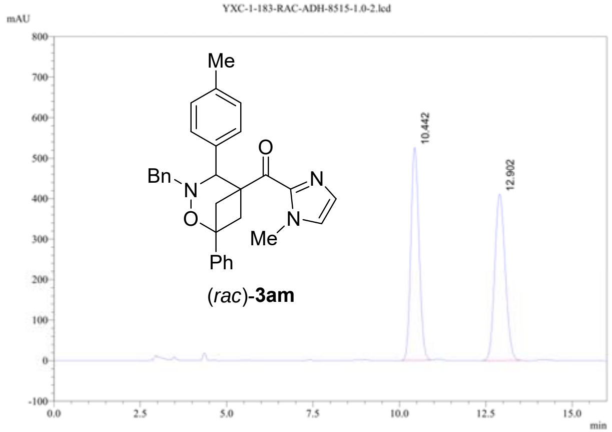

Detector A Channel 1 254nm

Peak#	Ret. Time [min]	Peak Start [min]	Height [mAU]	Area [mAU*min]	Area%
1	10.442	10.058	525678	8628731	49.861
2	12.902	12.392	411102	8676742	50.139
Total			936779	17305473	100.000

2024-03-08 16:28:10 1 / 1

Analysis Report

SHIMADZU LabSolutions

Sample Information

Sample Name	: YXC-1-183-ASY-ADH-8515-1.0-1
Sample ID	: YXC-1-183-ASY-ADH-8515-1.0-1
Data File	: YXC-1-183-ASY-ADH-8515-1.0-2.lcd
Method File	: 90-10.lcm
Report Format File	: DEFAULT.lsr
Date Acquired	: 2024/3/8 15:38:36
Date Processed	: 2024/3/8 16:01:53

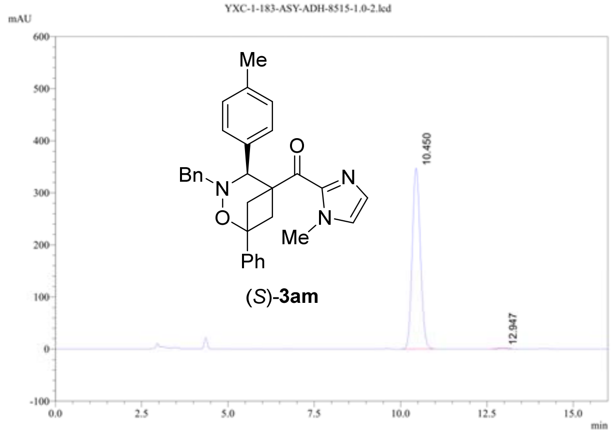

Detector A Channel 1 254nm

Peak#	Ret. Time [min]	Peak Start [min]	Height [mAU]	Area [mAU*min]	Area%
1	10.450	10.017	347759	5679335	99.159
2	12.947	12.592	2471	48178	0.841
Total			350230	5727513	100.000

Supplementary Figure 172. HPLC spectra of (*rac*)-**3am** and (*S*)-**3am**

2024-03-08 10:21:28 1 / 1

Analysis Report

SHIMADZU
LabSolutions

Sample Information

Sample Name	: YXC-1-187-RAC-ADH-8515-1.0-1
Sample ID	: YXC-1-187-RAC-ADH-8515-1.0-1
Data File	: YXC-1-187-RAC-ADH-8515-1.0-2.lcd
Method File	: 90-10.lcm
Report Format File	: DEFAULT.lsr
Date Acquired	: 2024/3/8 9:52:51
Date Processed	: 2024/3/8 10:18:33

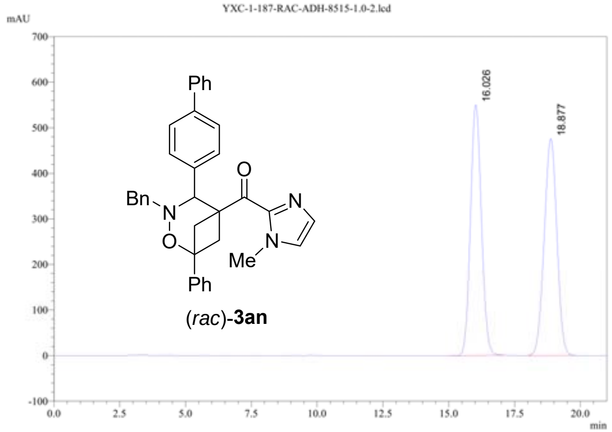

Detector A Channel 1 254nm

Peak#	Ret. Time [min]	Peak Start [min]	Height [mAU]	Area [mAU*min]	Area%
1	16.026	15.067	549967	15891895	49.912
2	18.877	17.925	475898	15947992	50.088
Total			1025865	31839887	100.000

2024-03-08 10:41:45 1 / 1

Analysis Report

SHIMADZU
LabSolutions

Sample Information

Sample Name	: YXC-1-187-ASY-ADH-8515-1.0-1
Sample ID	: YXC-1-187-ASY-ADH-8515-1.0-1
Data File	: YXC-1-187-ASY-ADH-8515-1.0-2.lcd
Method File	: 90-10.lcm
Report Format File	: DEFAULT.lsr
Date Acquired	: 2024/3/8 10:19:31
Date Processed	: 2024/3/8 10:41:07

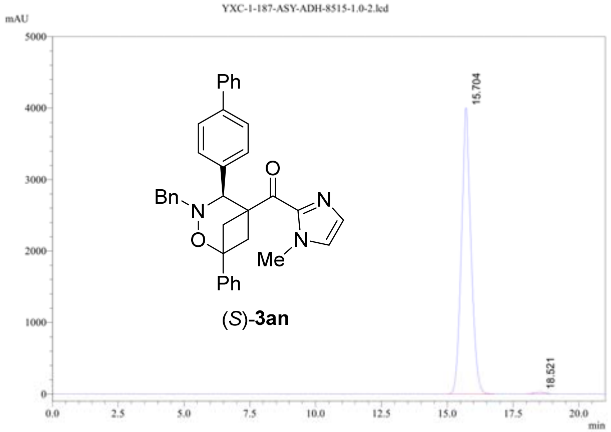

Detector A Channel 1 254nm

Peak#	Ret. Time [min]	Peak Start [min]	Height [mAU]	Area [mAU*min]	Area%
1	15.704	15.000	3998580	100714192	99.353
2	18.521	18.050	23121	655880	0.647
Total			4021701	101370072	100.000

Supplementary Figure 173. HPLC spectra of (*rac*)-**3an** and (*S*)-**3an**

2024-03-06 21:42:33 1 / 1

Analysis Report

SHIMADZU LabSolutions

	Sample Information
Sample Name	: YXC-1-186-RAC-ADH-8515-1.0-2
Sample ID	: YXC-1-186-RAC-ADH-8515-1.0-2
Data File	: YXC-1-186-RAC-ADH-8515-1.0-3.lcd
Method File	: 90-10.lcm
Report Format File	: DEFAULT.lsr
Date Acquired	: 2024/3/6 21:07:47
Date Processed	: 2024/3/6 21:42:20

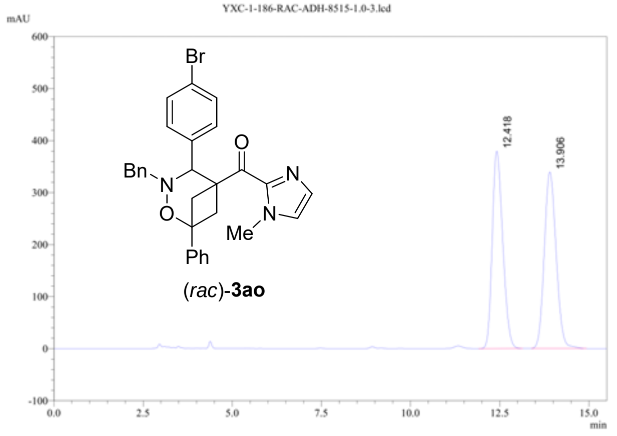

Detector A Channel 1 254nm

Peak#	Ret. Time [min]	Peak Start [min]	Height [mAU]	Area [mAU*min]	Area%
1	12.418	11.908	379652	7774863	49.728
2	13.906	13.383	339545	7859883	50.272
Total			719197	15634746	100.000

2024-03-06 21:43:45 1 / 1

Analysis Report

SHIMADZU LabSolutions

	Sample Information
Sample Name	: YXC-1-186-ASY-ADH-8515-1.0-2
Sample ID	: YXC-1-186-ASY-ADH-8515-1.0-2
Data File	: YXC-1-186-ASY-ADH-8515-1.0-3.lcd
Method File	: 90-10.lcm
Report Format File	: DEFAULT.lsr
Date Acquired	: 2024/3/6 21:24:51
Date Processed	: 2024/3/6 21:43:26

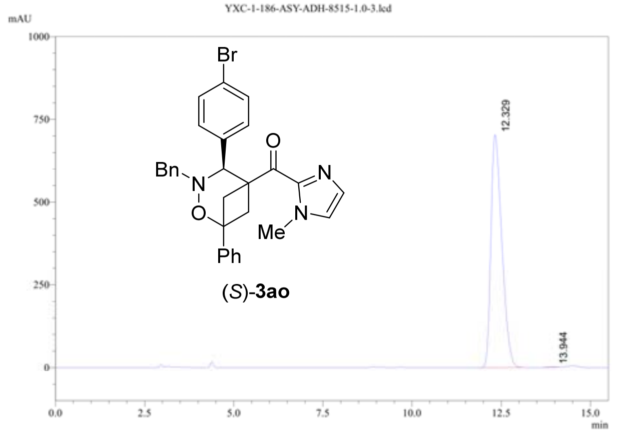

Detector A Channel 1 254nm

Peak#	Ret. Time [min]	Peak Start [min]	Height [mAU]	Area [mAU*min]	Area%
1	12.329	11.925	702846	14945422	99.813
2	13.944	13.650	1687	28046	0.187
Total			704533	14973468	100.000

Supplementary Figure 174. HPLC spectra of (*rac*)-**3ao** and (*S*)-**3ao**

2024-03-06 21:10:49 1 / 1

Analysis Report

SHIMADZU LabSolutions

Sample Information

Sample Name	: YXC-1-184-RAC-ADH-8515-1.0-2
Sample ID	: YXC-1-184-RAC-ADH-8515-1.0-2
Data File	: YXC-1-184-RAC-ADH-8515-1.0-3.lcd
Method File	: 90-10.lcm
Report Format File	: DEFAULT.lsr
Date Acquired	: 2024/3/6 20:09:52
Date Processed	: 2024/3/6 20:33:38

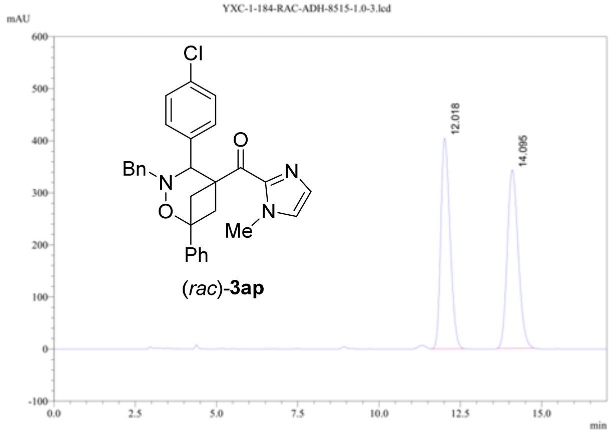

Detector A Channel 1 254nm

Peak#	Ret. Time [min]	Peak Start [min]	Height [mAU]	Area [mAU*min]	Area%
1	12.018	11.600	404519	7964018	49.828
2	14.095	13.633	342160	8019079	50.172
Total			746679	15983097	100.000

2024-03-06 21:09:52 1 / 1

Analysis Report

SHIMADZU LabSolutions

Sample Information

Sample Name	: YXC-1-184-ASY-ADH-8515-1.0-2
Sample ID	: YXC-1-184-ASY-ADH-8515-1.0-2
Data File	: YXC-1-184-ASY-ADH-8515-1.0-4.lcd
Method File	: 90-10.lcm
Report Format File	: DEFAULT.lsr
Date Acquired	: 2024/3/6 20:48:58
Date Processed	: 2024/3/6 21:09:31

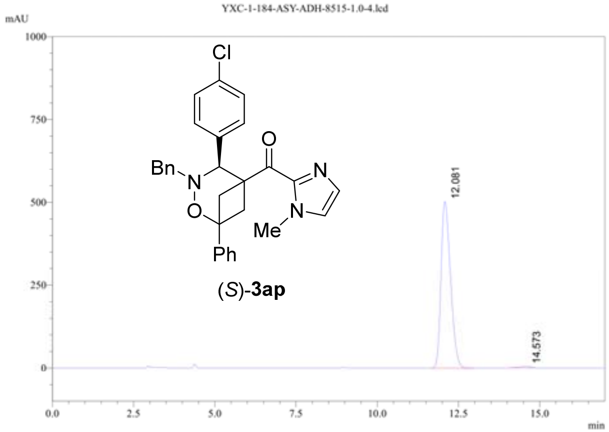

Detector A Channel 1 254nm

Peak#	Ret. Time [min]	Peak Start [min]	Height [mAU]	Area [mAU*min]	Area%
1	12.081	11.658	502613	10151025	98.904
2	14.573	14.042	4749	112448	1.096
Total			507362	10263474	100.000

Supplementary Figure 175. HPLC spectra of (*rac*)-**3ap** and (*S*)-**3ap**

2024-03-25 21:14:15 1 / 1

Analysis Report

SHIMADZU LabSolutions

Sample Information

Sample Name	: YXC-2-16-RAC-ADH-8515-1.0-1
Sample ID	: YXC-2-16-RAC-ADH-8515-1.0-1
Data File	: YXC-2-16-RAC-ADH-8515-1.0-2.lcd
Method File	: 90-10.lcm
Report Format File	: DEFAULT.lsr
Date Acquired	: 2024/3/25 20:52:58
Date Processed	: 2024/3/25 21:11:38

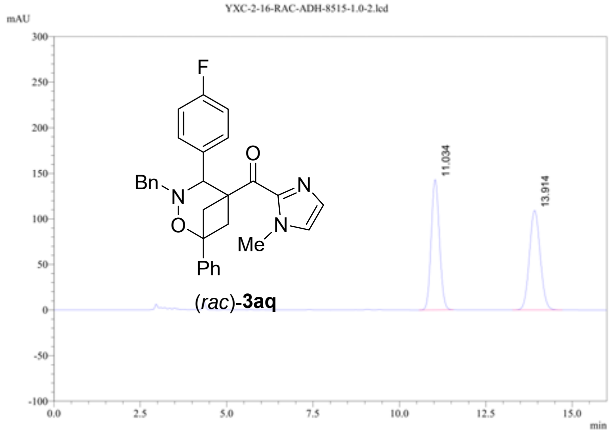

Detector A Channel 1 254nm

Peak#	Ret. Time [min]	Peak Start [min]	Height [mAU]	Area [mAU*min]	Area%
1	11.034	10.567	143088	2384230	50.038
2	13.914	13.275	109323	2380601	49.962
Total			252411	4764831	100.000

2024-03-25 21:44:53 1 / 1

Analysis Report

SHIMADZU LabSolutions

Sample Information

Sample Name	: YXC-2-16-ASY-ADH-8515-1.0-1
Sample ID	: YXC-2-16-ASY-ADH-8515-1.0-1
Data File	: YXC-2-16-ASY-ADH-8515-1.0-2.lcd
Method File	: 90-10.lcm
Report Format File	: DEFAULT.lsr
Date Acquired	: 2024/3/25 21:12:44
Date Processed	: 2024/3/25 21:44:32

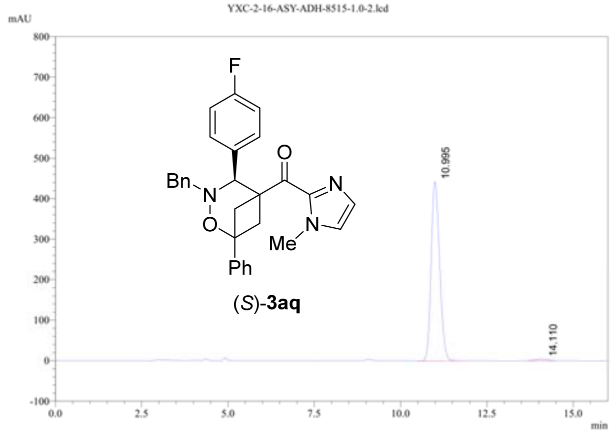

Detector A Channel 1 254nm

Peak#	Ret. Time [min]	Peak Start [min]	Height [mAU]	Area [mAU*min]	Area%
1	10.995	10.492	441646	7498265	98.775
2	14.110	13.650	3852	92967	1.225
Total			445498	7591233	100.000

Supplementary Figure 176. HPLC spectra of (*rac*)-**3aq** and (*S*)-**3aq**

2024-03-08 16:28:49 1 / 1

Analysis Report

SHIMADZU LabSolutions

Sample Information

Sample Name	: YXC-1-185-RAC-ADH-8515-1.0-1
Sample ID	: YXC-1-185-RAC-ADH-8515-1.0-1
Data File	: YXC-1-185-RAC-ADH-8515-1.0-2.lcd
Method File	: 90-10.lcm
Report Format File	: DEFAULT.lsr
Date Acquired	: 2024/3/8 16:02:59
Date Processed	: 2024/3/8 16:25:40

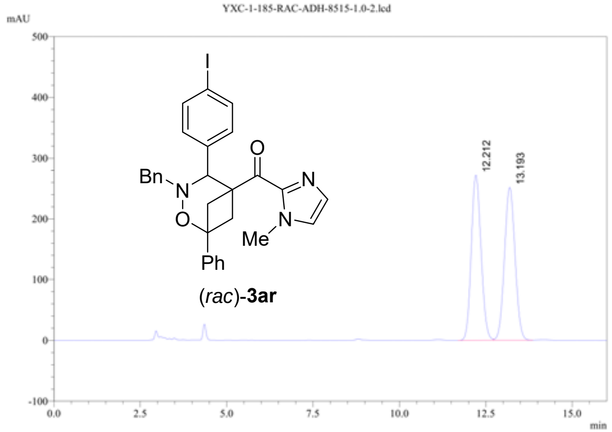

Detector A Channel 1 254nm

Peak#	Ret. Time [min]	Peak Start [min]	Height [mAU]	Area [mAU*min]	Area%
1	12.212	11.692	272013	5350557	49.985
2	13.193	12.725	251800	5353826	50.015
Total			523814	10704383	100.000

2024-03-08 16:43:39 1 / 1

Analysis Report

SHIMADZU LabSolutions

Sample Information

Sample Name	: YXC-1-185-ASY-ADH-8515-1.0-1
Sample ID	: YXC-1-185-ASY-ADH-8515-1.0-1
Data File	: YXC-1-185-ASY-ADH-8515-1.0-2.lcd
Method File	: 90-10.lcm
Report Format File	: DEFAULT.lsr
Date Acquired	: 2024/3/8 16:26:41
Date Processed	: 2024/3/8 16:43:18

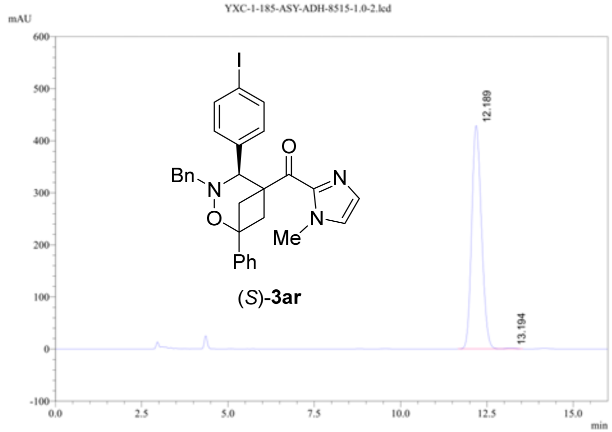

Detector A Channel 1 254nm

Peak#	Ret. Time [min]	Peak Start [min]	Height [mAU]	Area [mAU*min]	Area%
1	12.189	11.658	429140	8481412	99.524
2	13.194	12.892	1959	40571	0.476
Total			431098	8521983	100.000

Supplementary Figure 177. HPLC spectra of (*rac*)-**3ar** and (*S*)-**3ar**

2024-03-16 10:37:42 1 / 1

Analysis Report

SHIMADZU
LabSolutions

Sample Information

Sample Name	: YXC-1-194-RAC-IC-9505-0.8-1
Sample ID	: YXC-1-194-RAC-IC-9505-0.8-1
Data File	: YXC-1-194-RAC-IC-9505-0.8-2.lcd
Method File	: 90-10.lcm
Report Format File	: DEFAULT.lsr
Date Acquired	: 2024/3/16 10:17:55
Date Processed	: 2024/3/16 10:35:31

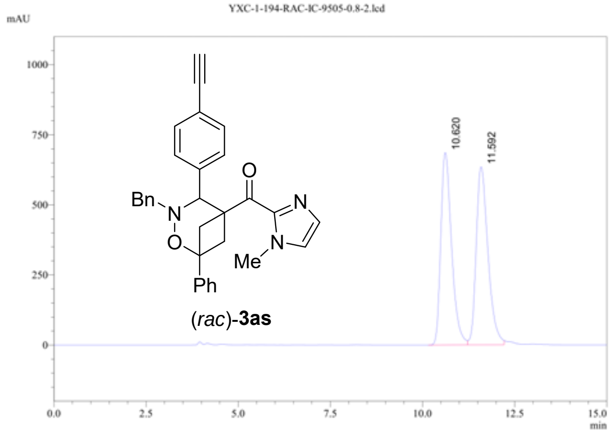

Detector A Channel 1 254nm

Peak#	Ret. Time [min]	Peak Start [min]	Height [mAU]	Area [mAU*min]	Area%
1	10.620	10.158	686376	13940064	49.657
2	11.592	11.225	635207	14132783	50.343
Total			1321583	28072847	100.000

2024-03-16 11:20:11 1 / 1

Analysis Report

SHIMADZU
LabSolutions

Sample Information

Sample Name	: YXC-1-194-ASY-IC-9505-0.8-1
Sample ID	: YXC-1-194-ASY-IC-9505-0.8-1
Data File	: YXC-1-194-ASY-IC-9505-0.8-3.lcd
Method File	: 90-10.lcm
Report Format File	: DEFAULT.lsr
Date Acquired	: 2024/3/16 10:54:53
Date Processed	: 2024/3/16 11:19:44

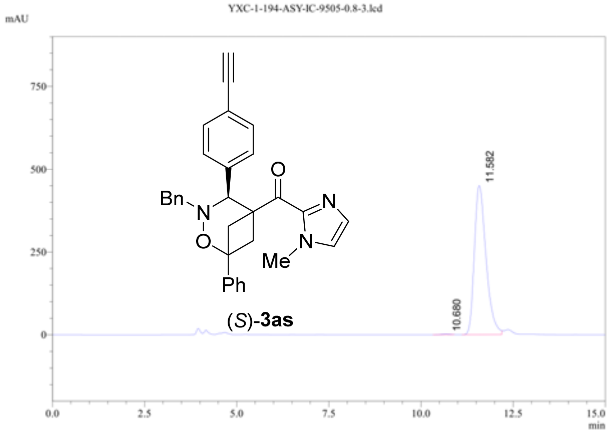

Detector A Channel 1 254nm

Peak#	Ret. Time [min]	Peak Start [min]	Height [mAU]	Area [mAU*min]	Area%
1	10.680	10.325	1758	29106	0.293
2	11.582	11.108	449978	9896263	99.707
Total			451736	9925369	100.000

Supplementary Figure 178. HPLC spectra of (*rac*)-**3as** and (*S*)-**3as**

2024-03-14 15:47:51 1 / 1

Analysis Report

SHIMADZU LabSolutions

Sample Information

Sample Name	: YXC-1-199-RAC-ADH-8515-1.0-1
Sample ID	: YXC-1-199-RAC-ADH-8515-1.0-1
Data File	: YXC-1-199-RAC-ADH-8515-1.0-2.lcd
Method File	: 90-10.lcm
Report Format File	: DEFAULT.lsr
Date Acquired	: 2024/3/14 14:59:45
Date Processed	: 2024/3/14 15:23:40

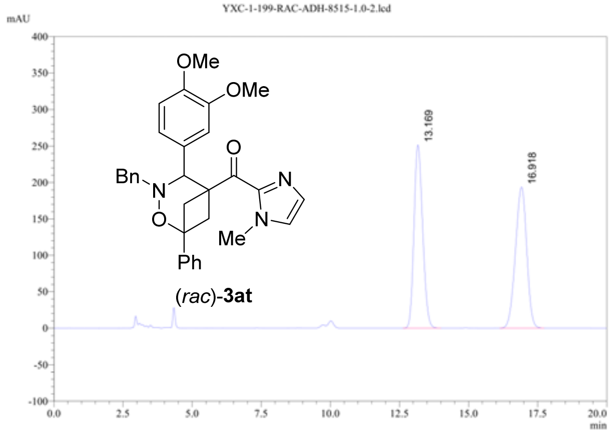

Detector A Channel 1 254nm

Peak#	Ret. Time [min]	Peak Start [min]	Height [mAU]	Area [mAU*min]	Area%
1	13.169	12.625	251515	5488511	50.000
2	16.918	16.133	193566	5488567	50.000
Total			445081	10977078	100.000

2024-03-14 15:48:15 1 / 1

Analysis Report

SHIMADZU LabSolutions

Sample Information

Sample Name	: YXC-1-199-ASY-ADH-8515-1.0-1
Sample ID	: YXC-1-199-ASY-ADH-8515-1.0-1
Data File	: YXC-1-199-ASY-ADH-8515-1.0-2.lcd
Method File	: 90-10.lcm
Report Format File	: DEFAULT.lsr
Date Acquired	: 2024/3/14 15:24:40
Date Processed	: 2024/3/14 15:47:07

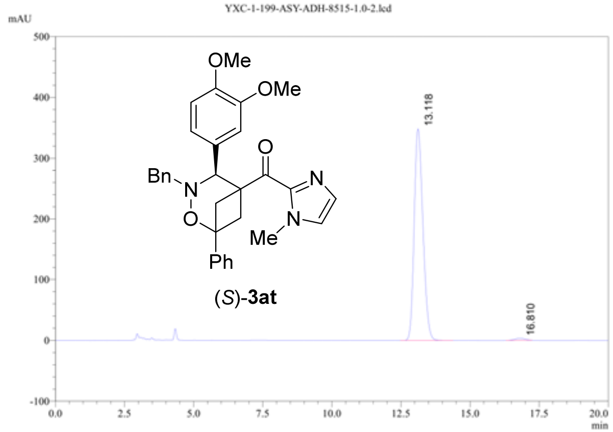

Detector A Channel 1 254nm

Peak#	Ret. Time [min]	Peak Start [min]	Height [mAU]	Area [mAU*min]	Area%
1	13.118	12.475	348438	7667272	98.875
2	16.810	16.300	3319	87214	1.125
Total			351757	7754486	100.000

Supplementary Figure 179. HPLC spectra of (*rac*)-**3at** and (*S*)-**3at**

2024-03-19 15:51:02 1 / 1

Analysis Report

SHIMADZU LabSolutions

	Sample Information
Sample Name	: YXC-2-14-RAC-ADH-9010-1.0-1
Sample ID	: YXC-2-14-RAC-ADH-9010-1.0-1
Data File	: YXC-2-14-RAC-ADH-9010-1.0-2.lcd
Method File	: 90-10.lcm
Report Format File	: DEFAULT.lsr
Date Acquired	: 2024/3/19 15:34:27
Date Processed	: 2024/3/19 15:48:45

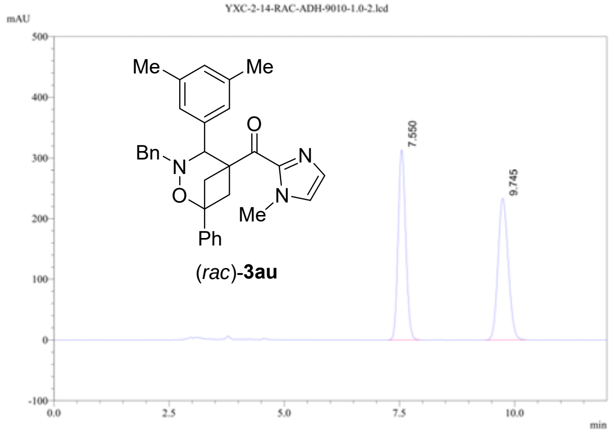

Detector A Channel 1 254nm

Peak#	Ret. Time [min]	Peak Start [min]	Height [mAU]	Area [mAU*min]	Area%
1	7.550	7.250	313314	3584898	49.891
2	9.745	9.358	233160	3600534	50.109
Total			546474	7185432	100.000

2024-03-19 16:02:06 1 / 1

Analysis Report

SHIMADZU LabSolutions

	Sample Information
Sample Name	: YXC-2-14-ASY-ADH-9010-1.0-1
Sample ID	: YXC-2-14-ASY-ADH-9010-1.0-1
Data File	: YXC-2-14-ASY-ADH-9010-1.0-2.lcd
Method File	: 90-10.lcm
Report Format File	: DEFAULT.lsr
Date Acquired	: 2024/3/19 15:49:41
Date Processed	: 2024/3/19 16:01:26

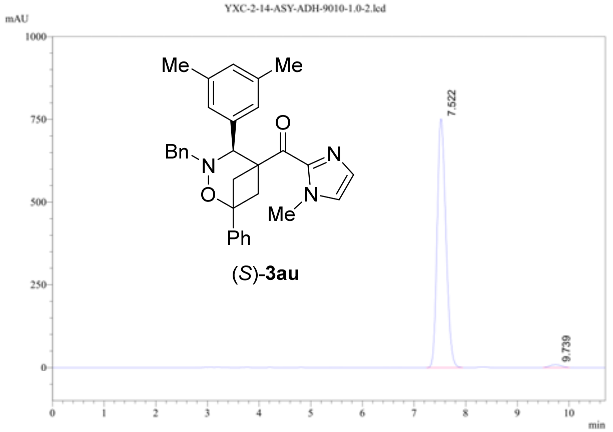

Detector A Channel 1 254nm

Peak#	Ret. Time [min]	Peak Start [min]	Height [mAU]	Area [mAU*min]	Area%
1	7.522	7.242	751519	8826986	98.507
2	9.739	9.492	8528	133821	1.493
Total			760047	8960807	100.000

Supplementary Figure 180. HPLC spectra of (*rac*)-**3au** and (*S*)-**3au**

2024-03-08 11:05:09 1 / 1

Analysis Report

SHIMADZU
LabSolutions

Sample Information

Sample Name	: YXC-1-188-RAC-ADH-8515-1.0-1
Sample ID	: YXC-1-188-RAC-ADH-8515-1.0-1
Data File	: YXC-1-188-RAC-ADH-8515-1.0-2.lcd
Method File	: 90-10.lcm
Report Format File	: DEFAULT.lsr
Date Acquired	: 2024/3/8 10:43:19
Date Processed	: 2024/3/8 11:04:40

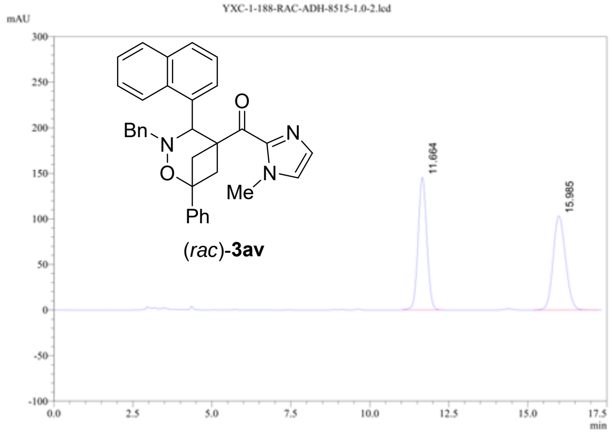

Detector A Channel 1 254nm

Peak#	Ret. Time [min]	Peak Start [min]	Height [mAU]	Area [mAU*min]	Area%
1	11.664	11.008	145567	2802690	49.799
2	15.985	15.183	103408	2825323	50.201
Total			248975	5628013	100.000

2024-03-08 11:20:13 1 / 1

Analysis Report

SHIMADZU
LabSolutions

Sample Information

Sample Name	: YXC-1-188-ASY-ADH-8515-1.0-1
Sample ID	: YXC-1-188-ASY-ADH-8515-1.0-1
Data File	: YXC-1-188-ASY-ADH-8515-1.0-2.lcd
Method File	: 90-10.lcm
Report Format File	: DEFAULT.lsr
Date Acquired	: 2024/3/8 11:01:39
Date Processed	: 2024/3/8 11:19:59

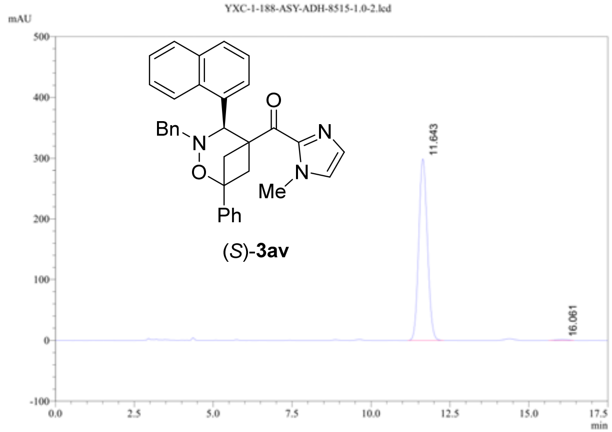

Detector A Channel 1 254nm

Peak#	Ret. Time [min]	Peak Start [min]	Height [mAU]	Area [mAU*min]	Area%
1	11.643	11.158	299057	5705082	99.497
2	16.061	15.625	1163	28852	0.503
Total			300220	5733934	100.000

Supplementary Figure 181. HPLC spectra of (*rac*)-**3av** and (*S*)-**3av**

2024-03-15 22:03:09 1 / 1

Analysis Report

SHIMADZU LabSolutions

Sample Information

Sample Name : YXC-1-190-RAC-IC-9010-0.8-1
Sample ID : YXC-1-190-RAC-IC-9010-0.8-1
Data File : YXC-1-190-RAC-IC-9010-0.8-3.lcd
Method File : 90-10.lcm
Report Format File : DEFAULT.lsr
Date Acquired : 2024/3/15 21:50:51
Date Processed : 2024/3/15 22:02:50

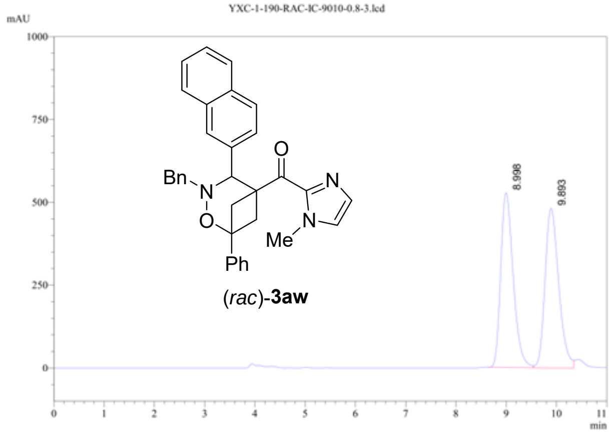

Detector A Channel 1 254nm

Peak#	Ret. Time [min]	Peak Start [min]	Height [mAU]	Area [mAU*min]	Area%
1	8.998	8.642	527180	9050652	49.864
2	9.893	9.542	481278	9099950	50.136
Total			1008459	18150601	100.000

2024-03-15 21:52:58 1 / 1

Analysis Report

SHIMADZU LabSolutions

Sample Information

Sample Name : YXC-1-190-ASY-IC-9010-0.8-1
Sample ID : YXC-1-190-ASY-IC-9010-0.8-1
Data File : YXC-1-190-ASY-IC-9010-0.8-2.lcd
Method File : 90-10.lcm
Report Format File : DEFAULT.lsr
Date Acquired : 2024/3/15 21:36:17
Date Processed : 2024/3/15 21:52:27

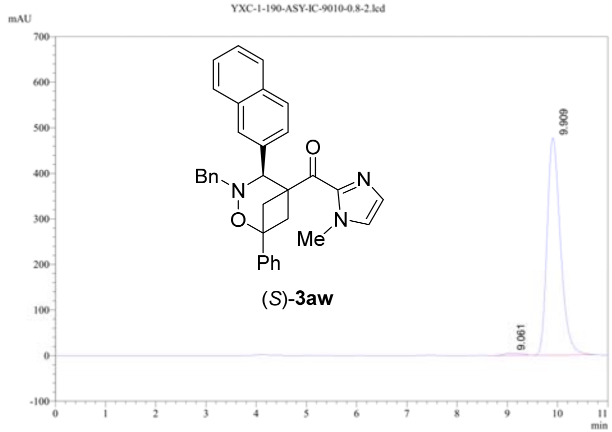

Detector A Channel 1 254nm

Peak#	Ret. Time [min]	Peak Start [min]	Height [mAU]	Area [mAU*min]	Area%
1	9.061	8.742	4897	101582	1.140
2	9.909	9.517	476848	8811454	98.860
Total			481745	8913036	100.000

Supplementary Figure 182. HPLC spectra of (*rac*)-**3aw** and (*S*)-**3aw**

2024-03-28 10:09:52 1 / 1

Analysis Report

SHIMADZU LabSolutions

Sample Information

Sample Name	: WWB-1-200-RAC-ADH-8515-1.0-2
Sample ID	: WWB-1-200-RAC-ADH-8515-1.0-2
Data File	: WWB-1-200-RAC-ADH-8515-1.0-3.lcd
Method File	: 90-10.lcm
Report Format File	: DEFAULT.lsr
Date Acquired	: 2024/3/28 9:33:38
Date Processed	: 2024/3/28 10:09:34

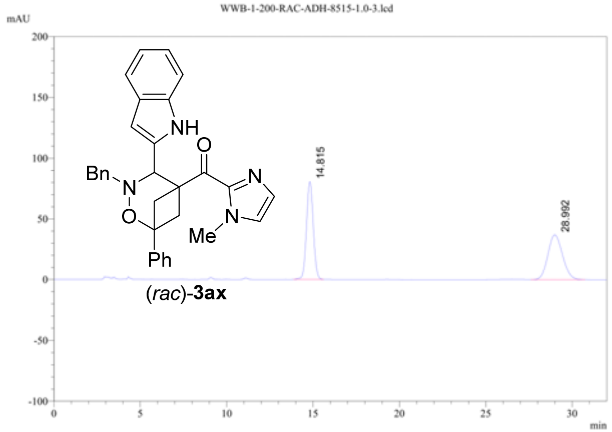

Detector A Channel 1 254nm

Peak#	Ret. Time [min]	Peak Start [min]	Height [mAU]	Area [mAU*min]	Area%
1	14.815	13.858	80685	2202970	50.342
2	28.992	27.608	36996	2173023	49.658
Total			117681	4375993	100.000

2024-03-28 10:42:08 1 / 1

Analysis Report

SHIMADZU LabSolutions

Sample Information

Sample Name	: WWB-1-200-ASY-ADH-8515-1.0-2
Sample ID	: WWB-1-200-ASY-ADH-8515-1.0-2
Data File	: WWB-1-200-ASY-ADH-8515-1.0-3.lcd
Method File	: 90-10.lcm
Report Format File	: DEFAULT.lsr
Date Acquired	: 2024/3/28 10:08:00
Date Processed	: 2024/3/28 10:41:28

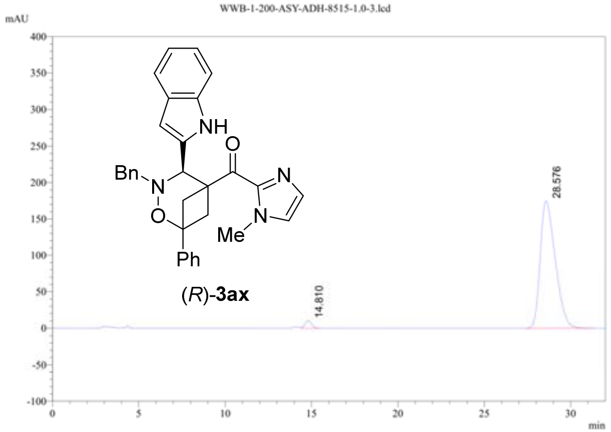

Detector A Channel 1 254nm

Peak#	Ret. Time [min]	Peak Start [min]	Height [mAU]	Area [mAU*min]	Area%
1	14.810	14.417	10410	277619	2.559
2	28.576	27.200	174527	10573168	97.441
Total			184938	10850787	100.000

Supplementary Figure 183. HPLC spectra of (*rac*)-**3ax** and (*R*)-**3ax**

2024-03-27 21:55:49 1 / 1

Analysis Report

SHIMADZU LabSolutions

Sample Information

Sample Name	: WWB-1-198-RAC-ADH-8515-1.0-2
Sample ID	: WWB-1-198-RAC-ADH-8515-1.0-2
Data File	: WWB-1-198-RAC-ADH-8515-1.0-3.lcd
Method File	: 90-10.lcm
Report Format File	: DEFAULT.lsr
Date Acquired	: 2024/3/27 21:11:59
Date Processed	: 2024/3/27 21:33:35

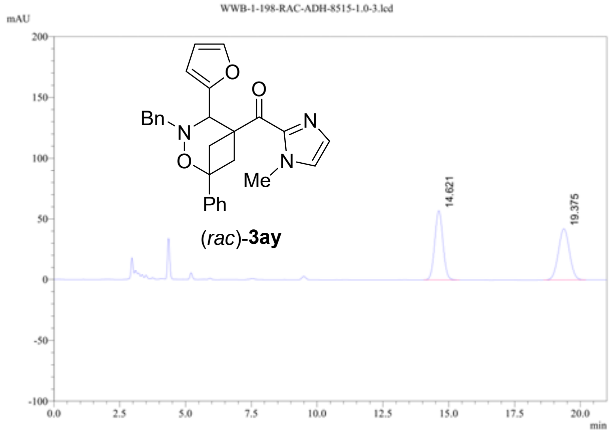

Detector A Channel 1 254nm

Peak#	Ret. Time [min]	Peak Start [min]	Height [mAU]	Area [mAU*min]	Area%
1	14.621	14.050	57087	1235613	50.064
2	19.375	18.600	42211	1232456	49.936
Total			99298	2468069	100.000

2024-03-27 21:56:43 1 / 1

Analysis Report

SHIMADZU LabSolutions

Sample Information

Sample Name	: WWB-1-198-ASY-ADH-8515-1.0-2
Sample ID	: WWB-1-198-ASY-ADH-8515-1.0-2
Data File	: WWB-1-198-ASY-ADH-8515-1.0-3.lcd
Method File	: 90-10.lcm
Report Format File	: DEFAULT.lsr
Date Acquired	: 2024/3/27 21:34:28
Date Processed	: 2024/3/27 21:56:26

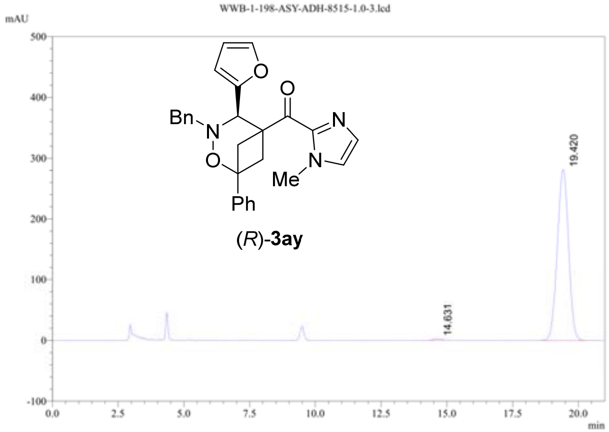

Detector A Channel 1 254nm

Peak#	Ret. Time [min]	Peak Start [min]	Height [mAU]	Area [mAU*min]	Area%
1	14.631	14.342	2359	43221	0.515
2	19.420	18.567	281642	8352376	99.485
Total			284001	8395597	100.000

Supplementary Figure 184. HPLC spectra of (*rac*)-**3ay** and (*R*)-**3ay**

2024-03-28 09:15:31 1 / 1

Analysis Report

SHIMADZU LabSolutions

Sample Information

Sample Name	: WWB-1-199-RAC-ADH-8515-1.0-2
Sample ID	: WWB-1-199-RAC-ADH-8515-1.0-2
Data File	: WWB-1-199-RAC-ADH-8515-1.0-3.lcd
Method File	: 90-10.lcm
Report Format File	: DEFAULT.lsr
Date Acquired	: 2024/3/28 8:54:41
Date Processed	: 2024/3/28 9:11:44

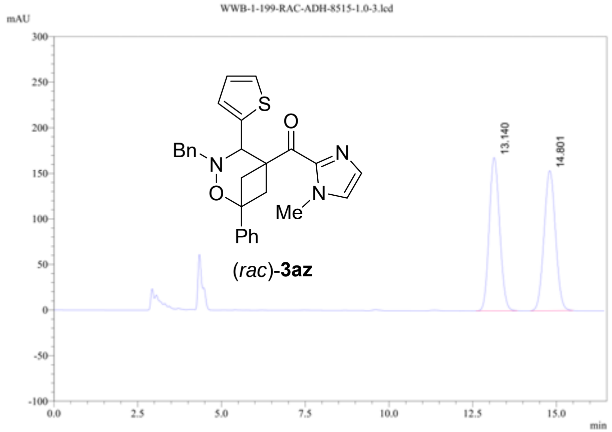

Detector A Channel 1 254nm

Peak#	Ret. Time [min]	Peak Start [min]	Height [mAU]	Area [mAU*min]	Area%
1	13.140	12.600	168497	3655273	49.941
2	14.801	14.225	153936	3663928	50.059
Total			322434	7319201	100.000

2024-03-28 09:32:26 1 / 1

Analysis Report

SHIMADZU LabSolutions

Sample Information

Sample Name	: WWB-1-199-ASY-ADH-8515-1.0-2
Sample ID	: WWB-1-199-ASY-ADH-8515-1.0-2
Data File	: WWB-1-199-ASY-ADH-8515-1.0-3.lcd
Method File	: 90-10.lcm
Report Format File	: DEFAULT.lsr
Date Acquired	: 2024/3/28 9:12:49
Date Processed	: 2024/3/28 9:32:00

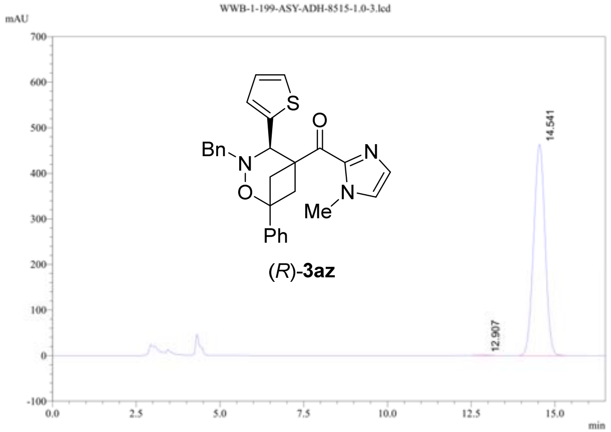

Detector A Channel 1 254nm

Peak#	Ret. Time [min]	Peak Start [min]	Height [mAU]	Area [mAU*min]	Area%
1	12.907	12.533	799	15858	0.145
2	14.541	13.842	463538	10925145	99.855
Total			464337	10941004	100.000

Supplementary Figure 185. HPLC spectra of (*rac*)-**3az** and (*R*)-**3az**

2024-04-24 10:27:16 1 / 1

Analysis Report

SHIMADZU LabSolutions

Sample Information

Sample Name	: WWB-2-1-RAC-ADH-8515-1.0-2
Sample ID	: WWB-2-1-RAC-ADH-8515-1.0-2
Data File	: WWB-2-1-RAC-ADH-8515-1.0-3.lcd
Method File	: 90-10.lcm
Report Format File	: DEFAULT.lsr
Date Acquired	: 2024/3/28 10:43:10
Date Processed	: 2024/3/28 11:02:12

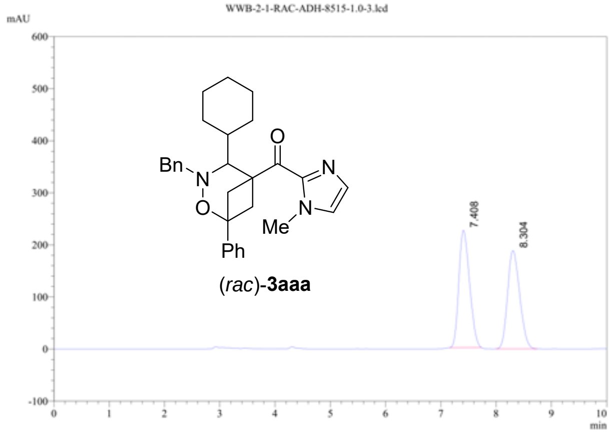

(*rac*)-**3aaa**

Detector A Channel 1 254nm

Peak#	Ret. Time [min]	Peak Start [min]	Height [mAU]	Area [mAU*min]	Area%
1	7.408	7.158	225273	3025053	51.303
2	8.304	7.992	188617	2871408	48.697
Total			413890	5896462	100.000

2024-04-24 10:27:30 1 / 1

Analysis Report

SHIMADZU LabSolutions

Sample Information

Sample Name	: WWB-2-1-ASY-ADH-8515-1.0-2
Sample ID	: WWB-2-1-ASY-ADH-8515-1.0-2
Data File	: WWB-2-1-ASY-ADH-8515-1.0-3.lcd
Method File	: 90-10.lcm
Report Format File	: DEFAULT.lsr
Date Acquired	: 2024/3/28 11:03:03
Date Processed	: 2024/3/28 11:14:58

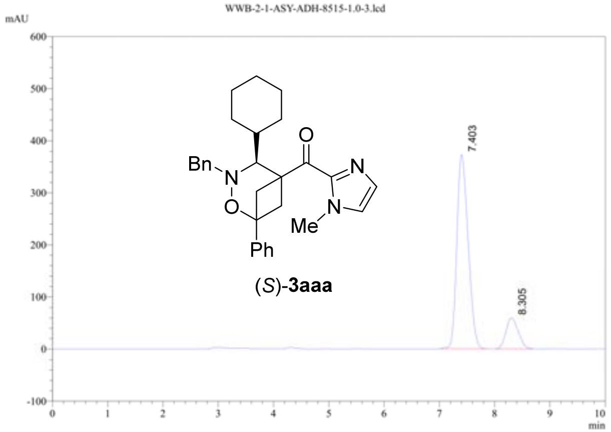

(*S*)-**3aaa**

Detector A Channel 1 254nm

Peak#	Ret. Time [min]	Peak Start [min]	Height [mAU]	Area [mAU*min]	Area%
1	7.403	7.008	373312	5284383	85.203
2	8.305	8.017	59607	917696	14.797
Total			432919	6202079	100.000

Supplementary Figure 186. HPLC spectra of (*rac*)-**3aaa** and (*S*)-**3aaa**

2024-03-19 15:15:15 1 / 1

Analysis Report

SHIMADZU LabSolutions

Sample Information

Sample Name	: YXC-2-12-RAC-ADH-9010-1.0-1
Sample ID	: YXC-2-12-RAC-ADH-9010-1.0-1
Data File	: YXC-2-12-RAC-ADH-9010-1.0-2.lcd
Method File	: 90-10.lcm
Report Format File	: DEFAULT.lsr
Date Acquired	: 2024/3/19 14:51:47
Date Processed	: 2024/3/19 15:15:01

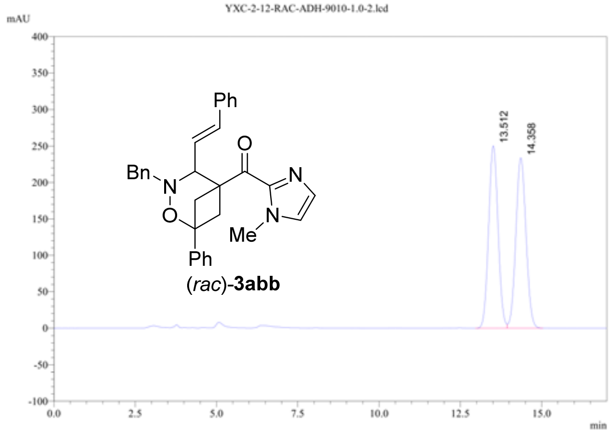

Detector A Channel 1 254nm

Peak#	Ret. Time [min]	Peak Start [min]	Height [mAU]	Area [mAU*min]	Area%
1	13.512	12.958	250510	5061689	49.893
2	14.358	13.942	233945	5083312	50.107
Total			484455	10145000	100.000

2024-03-19 15:36:53 1 / 1

Analysis Report

SHIMADZU LabSolutions

Sample Information

Sample Name	: YXC-2-12-ASY-ADH-9010-1.0-1
Sample ID	: YXC-2-12-ASY-ADH-9010-1.0-1
Data File	: YXC-2-12-ASY-ADH-9010-1.0-2.lcd
Method File	: 90-10.lcm
Report Format File	: DEFAULT.lsr
Date Acquired	: 2024/3/19 15:13:48
Date Processed	: 2024/3/19 15:32:18

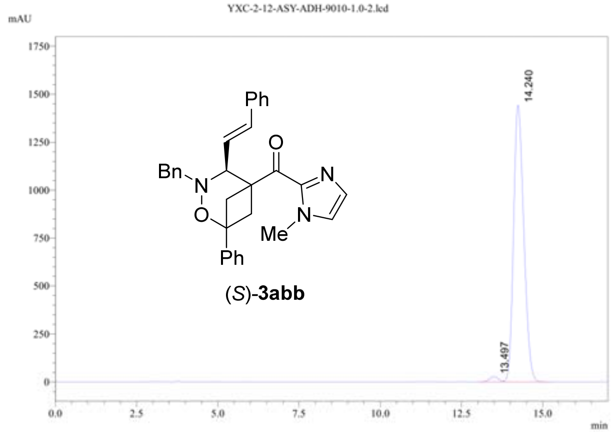

Detector A Channel 1 254nm

Peak#	Ret. Time [min]	Peak Start [min]	Height [mAU]	Area [mAU*min]	Area%
1	13.497	13.017	27984	552578	1.716
2	14.240	13.792	1442364	31645346	98.284
Total			1470348	32197924	100.000

Supplementary Figure 187. HPLC spectra of (*rac*)-**3abb** and (*S*)-**3abb**

2024-03-20 18:43:49 1 / 1

Analysis Report

SHIMADZU LabSolutions

Sample Information

Sample Name	: YXC-2-1-RAC-ADH-8515-1.0-1
Sample ID	: YXC-2-1-RAC-ADH-8515-1.0-1
Data File	: YXC-2-1-RAC-ADH-8515-1.0-2.lcd
Method File	: 90-10.lcm
Report Format File	: DEFAULT.lsr
Date Acquired	: 2024/3/20 14:31:36
Date Processed	: 2024/3/20 14:57:27

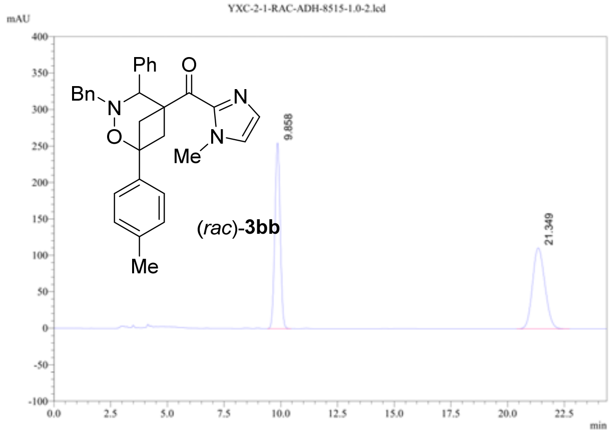

Detector A Channel 1 254nm

Peak#	Ret. Time [min]	Peak Start [min]	Height [mAU]	Area [mAU*min]	Area%
1	9.858	9.408	254896	4205405	50.024
2	21.349	20.400	110903	4201438	49.976
Total			365799	8406843	100.000

2024-03-20 18:44:18 1 / 1

Analysis Report

SHIMADZU LabSolutions

Sample Information

Sample Name	: YXC-2-1-ASY-ADH-8515-1.0-2
Sample ID	: YXC-2-1-ASY-ADH-8515-1.0-2
Data File	: YXC-2-1-ASY-ADH-8515-1.0-3.lcd
Method File	: 90-10.lcm
Report Format File	: DEFAULT.lsr
Date Acquired	: 2024/3/20 17:50:04
Date Processed	: 2024/3/20 18:14:48

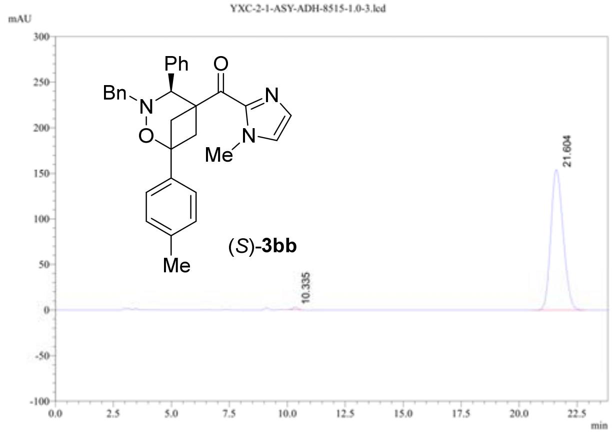

Detector A Channel 1 254nm

Peak#	Ret. Time [min]	Peak Start [min]	Height [mAU]	Area [mAU*min]	Area%
1	10.335	10.058	2695	38841	0.677
2	21.604	20.625	154136	5697163	99.323
Total			156831	5736004	100.000

Supplementary Figure 188. HPLC spectra of (*rac*)-**3bb** and (*S*)-**3bb**

2024-03-18 20:50:39 1 / 1

Analysis Report

SHIMADZU LabSolutions

Sample Information

Sample Name	: YXC-2-2-RAC-ADH-8515-1.0-2
Sample ID	: YXC-2-2-RAC-ADH-8515-1.0-2
Data File	: YXC-2-2-RAC-ADH-8515-1.0-3.lcd
Method File	: 90-10.lcm
Report Format File	: DEFAULT.lsr
Date Acquired	: 2024/3/18 20:07:47
Date Processed	: 2024/3/18 20:48:05

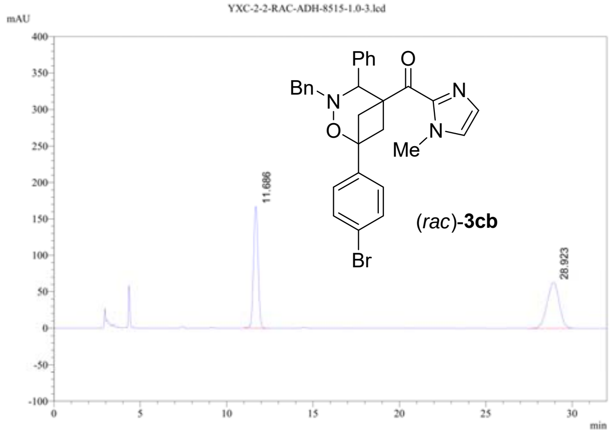

Detector A Channel 1 254nm

Peak#	Ret. Time [min]	Peak Start [min]	Height [mAU]	Area [mAU*min]	Area%
1	11.686	11.000	167007	3040222	50.060
2	28.923	27.583	62902	3032949	49.940
Total			229909	6073171	100.000

2024-03-18 21:20:40 1 / 1

Analysis Report

SHIMADZU LabSolutions

Sample Information

Sample Name	: YXC-2-2-ASY-ADH-8515-1.0-2
Sample ID	: YXC-2-2-ASY-ADH-8515-1.0-2
Data File	: YXC-2-2-ASY-ADH-8515-1.0-3.lcd
Method File	: 90-10.lcm
Report Format File	: DEFAULT.lsr
Date Acquired	: 2024/3/18 20:48:56
Date Processed	: 2024/3/18 21:20:20

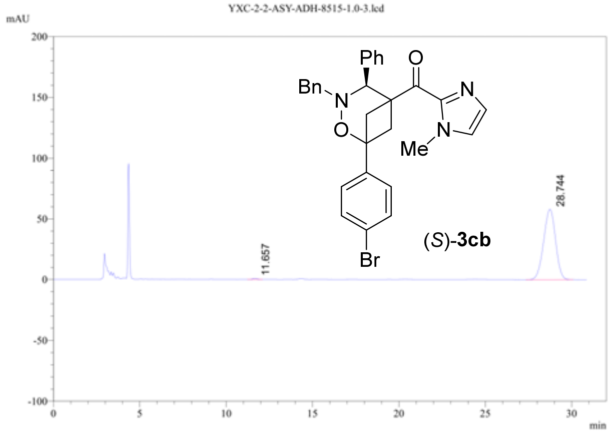

Detector A Channel 1 254nm

Peak#	Ret. Time [min]	Peak Start [min]	Height [mAU]	Area [mAU*min]	Area%
1	11.657	11.217	863	16314	0.582
2	28.744	27.350	58084	2785904	99.418
Total			58947	2802218	100.000

Supplementary Figure 189. HPLC spectra of (*rac*)-**3cb** and (*S*)-**3cb**

2024-03-30 17:07:08 1 / 1

Analysis Report

SHIMADZU LabSolutions

Sample Information

Sample Name	: WWB-2-13-RAC-ADH-8515-1.0-1
Sample ID	: WWB-2-13-RAC-ADH-8515-1.0-1
Data File	: WWB-2-13-RAC-ADH-8515-1.0-2.lcd
Method File	: 90-10.lcm
Report Format File	: DEFAULT.lsr
Date Acquired	: 2024/3/30 16:40:54
Date Processed	: 2024/3/30 17:06:36

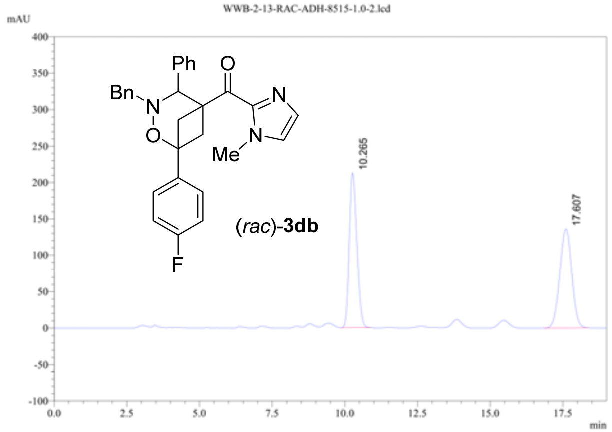

Detector A Channel 1 254nm

Peak#	Ret. Time [min]	Peak Start [min]	Height [mAU]	Area [mAU*min]	Area%
1	10.265	9.867	212656	3870818	49.855
2	17.607	16.850	136000	3893389	50.145
Total			348656	7764207	100.000

2024-03-30 17:22:24 1 / 1

Analysis Report

SHIMADZU LabSolutions

Sample Information

Sample Name	: WWB-2-13-ASY-ADH-8515-1.0-1
Sample ID	: WWB-2-13-ASY-ADH-8515-1.0-1
Data File	: WWB-2-13-ASY-ADH-8515-1.0-2.lcd
Method File	: 90-10.lcm
Report Format File	: DEFAULT.lsr
Date Acquired	: 2024/3/30 17:01:25
Date Processed	: 2024/3/30 17:21:44

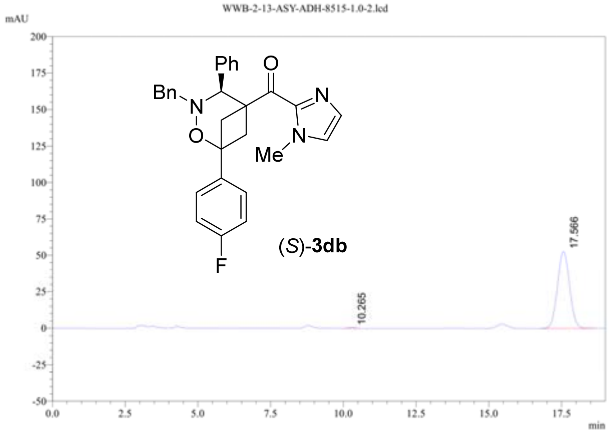

Detector A Channel 1 254nm

Peak#	Ret. Time [min]	Peak Start [min]	Height [mAU]	Area [mAU*min]	Area%
1	10.265	10.033	508	8307	0.545
2	17.566	16.792	52666	1516699	99.455
Total			53175	1525005	100.000

Supplementary Figure 190. HPLC spectra of (*rac*)-**3db** and (*S*)-**3db**

2024-05-07 16:10:29 1 / 1

Analysis Report

SHIMADZU
LabSolutions

Sample Information

Sample Name	: WWB-2-57-RAC-ADH-8515-1.0-1
Sample ID	: WWB-2-57-RAC-ADH-8515-1.0-1
Data File	: WWB-2-57-RAC-ADH-8515-1.0-2.lcd
Method File	: 98-02.lcm
Report Format File	: DEFAULT.lsr
Date Acquired	: 2024/5/7 14:54:53
Date Processed	: 2024/5/7 16:09:54

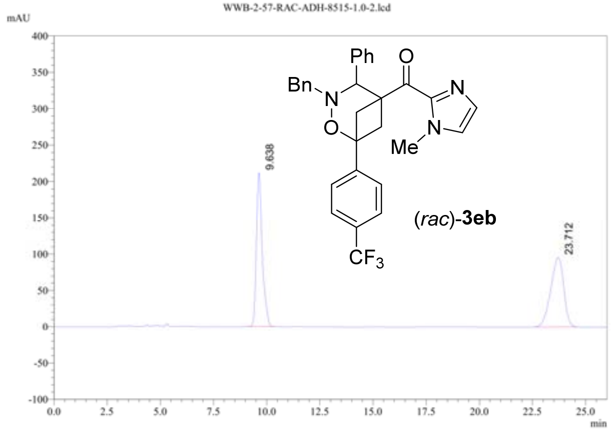

Detector A Channel 1 254nm

Peak#	Ret. Time [min]	Peak Start [min]	Height [mAU]	Area [mAU*min]	Area%
1	9.638	9.067	211492	4045334	49.963
2	23.712	22.583	95526	4051254	50.037
Total			307019	8096588	100.000

2024-05-07 16:10:43 1 / 1

Analysis Report

SHIMADZU
LabSolutions

Sample Information

Sample Name	: WWB-2-57-ASY-ADH-8515-1.0-1
Sample ID	: WWB-2-57-ASY-ADH-8515-1.0-1
Data File	: WWB-2-57-ASY-ADH-8515-1.0-2.lcd
Method File	: 98-02.lcm
Report Format File	: DEFAULT.lsr
Date Acquired	: 2024/5/7 15:40:44
Date Processed	: 2024/5/7 16:09:31

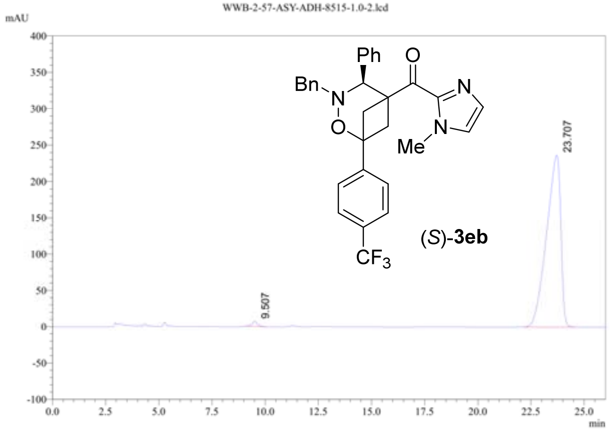

Detector A Channel 1 254nm

Peak#	Ret. Time [min]	Peak Start [min]	Height [mAU]	Area [mAU*min]	Area%
1	9.507	9.050	6882	133805	1.203
2	23.707	22.108	236479	10990996	98.797
Total			243361	11124800	100.000

Supplementary Figure 191. HPLC spectra of (*rac*)-**3eb** and (*S*)-**3eb**

2024-04-02 15:21:37 1 / 1

Analysis Report

SHIMADZU LabSolutions

Sample Information

Sample Name	: WWB-2-14-RAC-ADH-9010-1.0-1
Sample ID	: WWB-2-14-RAC-ADH-9010-1.0-1
Data File	: WWB-2-14-RAC-ADH-9010-1.0-4.lcd
Method File	: 90-10.lcm
Report Format File	: DEFAULT.lsr
Date Acquired	: 2024/4/2 15:11:08
Date Processed	: 2024/4/2 15:21:00

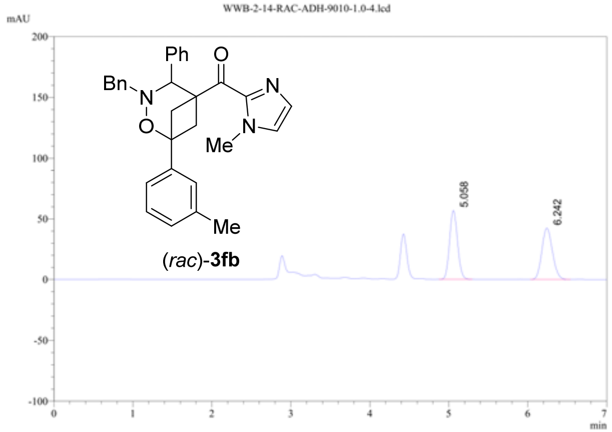

Detector A Channel 1 254nm

Peak#	Ret. Time [min]	Peak Start [min]	Height [mAU]	Area [mAU*min]	Area%
1	5.058	4.875	56902	399463	50.169
2	6.242	6.033	42483	396773	49.831
Total			99385	796236	100.000

2024-04-02 15:27:43 1 / 1

Analysis Report

SHIMADZU LabSolutions

Sample Information

Sample Name	: WWB-2-14-ASY-ADH-9010-1.0-1
Sample ID	: WWB-2-14-ASY-ADH-9010-1.0-1
Data File	: WWB-2-14-ASY-ADH-9010-1.0-2.lcd
Method File	: 90-10.lcm
Report Format File	: DEFAULT.lsr
Date Acquired	: 2024/4/2 15:19:13
Date Processed	: 2024/4/2 15:27:34

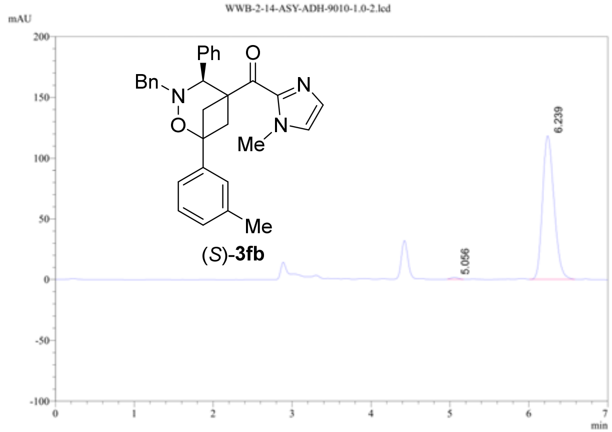

Detector A Channel 1 254nm

Peak#	Ret. Time [min]	Peak Start [min]	Height [mAU]	Area [mAU*min]	Area%
1	5.056	4.967	1444	8995	0.742
2	6.239	6.008	118292	1203188	99.258
Total			119736	1212183	100.000

Supplementary Figure 192. HPLC spectra of (*rac*)-**3fb** and (*S*)-**3fb**

2024-03-20 18:45:08 1 / 1

Analysis Report

SHIMADZU LabSolutions

Sample Information

Sample Name	: YXC-2-7-RAC-ADH-8515-1.0-1
Sample ID	: YXC-2-7-RAC-ADH-8515-1.0-1
Data File	: YXC-2-7-RAC-ADH-8515-1.0-2.lcd
Method File	: 90-10.lcm
Report Format File	: DEFAULT.lsr
Date Acquired	: 2024/3/20 14:56:49
Date Processed	: 2024/3/20 15:17:10

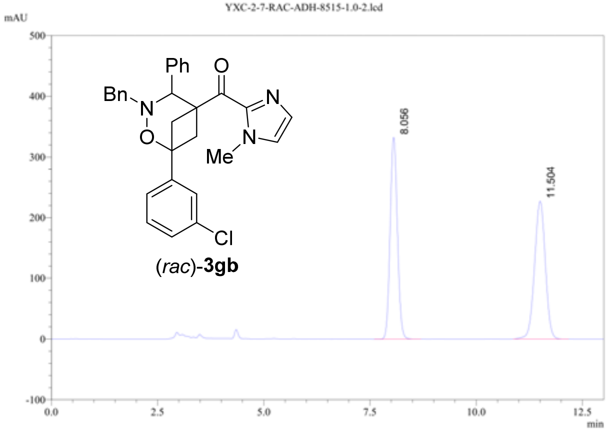

Detector A Channel 1 254nm

Peak#	Ret. Time [min]	Peak Start [min]	Height [mAU]	Area [mAU*min]	Area%
1	8.056	7.600	332645	3943502	49.607
2	11.504	10.867	227063	4006009	50.393
Total			559708	7949511	100.000

2024-03-20 18:59:40 1 / 1

Analysis Report

SHIMADZU LabSolutions

Sample Information

Sample Name	: YXC-2-7-ASY-ADH-8515-1.0-2
Sample ID	: YXC-2-7-ASY-ADH-8515-1.0-2
Data File	: YXC-2-7-ASY-ADH-8515-1.0-3.lcd
Method File	: 90-10.lcm
Report Format File	: DEFAULT.lsr
Date Acquired	: 2024/3/20 18:41:20
Date Processed	: 2024/3/20 18:59:31

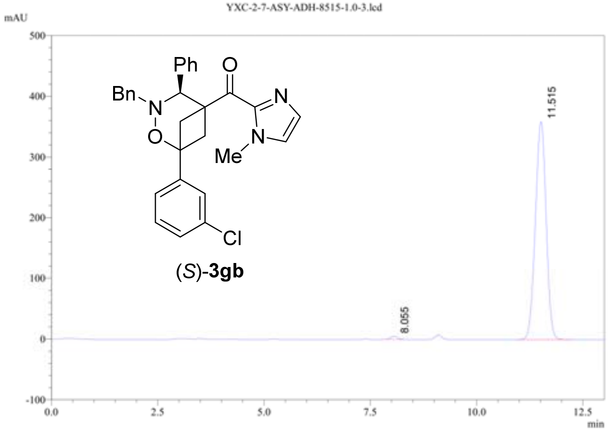

Detector A Channel 1 254nm

Peak#	Ret. Time [min]	Peak Start [min]	Height [mAU]	Area [mAU*min]	Area%
1	8.055	7.867	5434	61607	0.964
2	11.515	10.942	359239	6328886	99.036
Total			364673	6390493	100.000

Supplementary Figure 193. HPLC spectra of (*rac*)-**3gb** and (*S*)-**3gb**

2024-03-18 21:45:14 1 / 1

Analysis Report

SHIMADZU LabSolutions

Sample Information

Sample Name	: YXC-2-5-RAC-ADH-8515-1.0-2
Sample ID	: YXC-2-5-RAC-ADH-8515-1.0-2
Data File	: YXC-2-5-RAC-ADH-8515-1.0-3.lcd
Method File	: 90-10.lcm
Report Format File	: DEFAULT.lsr
Date Acquired	: 2024/3/18 21:22:14
Date Processed	: 2024/3/18 21:44:49

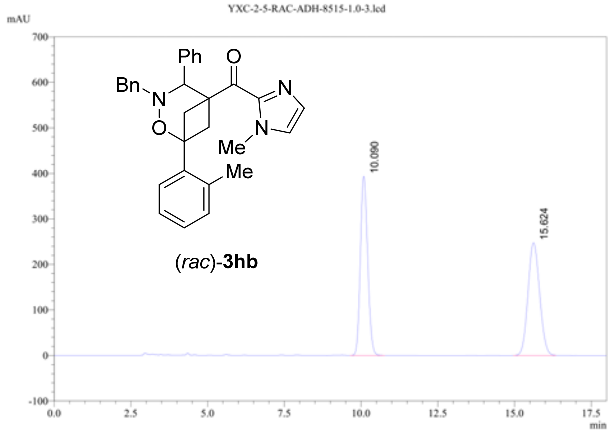

Detector A Channel 1 254nm

Peak#	Ret. Time [min]	Peak Start [min]	Height [mAU]	Area [mAU*min]	Area%
1	10.090	9.642	394073	6504813	49.974
2	15.624	14.958	247342	6511606	50.026
Total			641415	13016419	100.000

2024-03-18 22:02:41 1 / 1

Analysis Report

SHIMADZU LabSolutions

Sample Information

Sample Name	: YXC-2-5-ASY-ADH-8515-1.0-2
Sample ID	: YXC-2-5-ASY-ADH-8515-1.0-2
Data File	: YXC-2-5-ASY-ADH-8515-1.0-3.lcd
Method File	: 90-10.lcm
Report Format File	: DEFAULT.lsr
Date Acquired	: 2024/3/18 21:43:29
Date Processed	: 2024/3/18 22:02:21

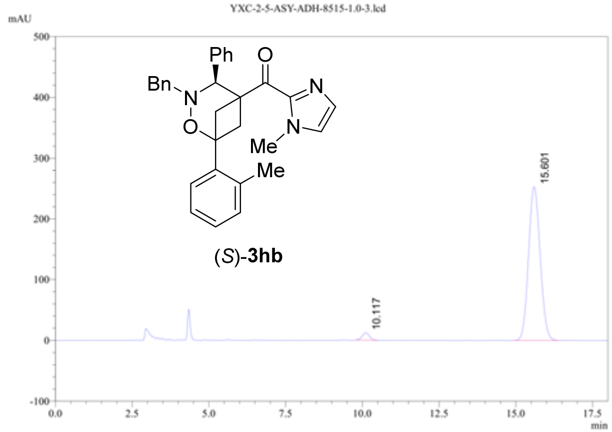

Detector A Channel 1 254nm

Peak#	Ret. Time [min]	Peak Start [min]	Height [mAU]	Area [mAU*min]	Area%
1	10.117	9.783	12243	198668	2.894
2	15.601	14.900	253099	6665021	97.106
Total			265342	6863688	100.000

Supplementary Figure 194. HPLC spectra of (*rac*)-**3hb** and (*S*)-**3hb**

2024-03-16 17:09:23 1 / 1

Analysis Report

SHIMADZU LabSolutions

Sample Information

Sample Name	: YXC-2-3-RAC-ADH-8515-1.0-1
Sample ID	: YXC-2-3-RAC-ADH-8515-1.0-1
Data File	: YXC-2-3-RAC-ADH-8515-1.0-2.lcd
Method File	: 90-10.lcm
Report Format File	: DEFAULT.lsr
Date Acquired	: 2024/3/16 16:35:06
Date Processed	: 2024/3/16 17:09:05

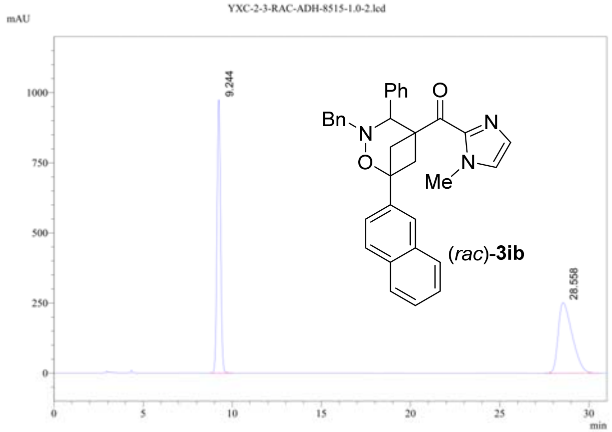

Detector A Channel 1 254nm

Peak#	Ret. Time [min]	Peak Start [min]	Height [mAU]	Area [mAU*min]	Area%
1	9.244	8.725	975047	13563347	49.721
2	28.558	27.508	251205	13715627	50.279
Total			1226252	27278973	100.000

2024-03-16 17:40:24 1 / 1

Analysis Report

SHIMADZU LabSolutions

Sample Information

Sample Name	: YXC-2-3-ASY-ADH-8515-1.0-1
Sample ID	: YXC-2-3-ASY-ADH-8515-1.0-1
Data File	: YXC-2-3-ASY-ADH-8515-1.0-2.lcd
Method File	: 90-10.lcm
Report Format File	: DEFAULT.lsr
Date Acquired	: 2024/3/16 17:07:13
Date Processed	: 2024/3/16 17:40:04

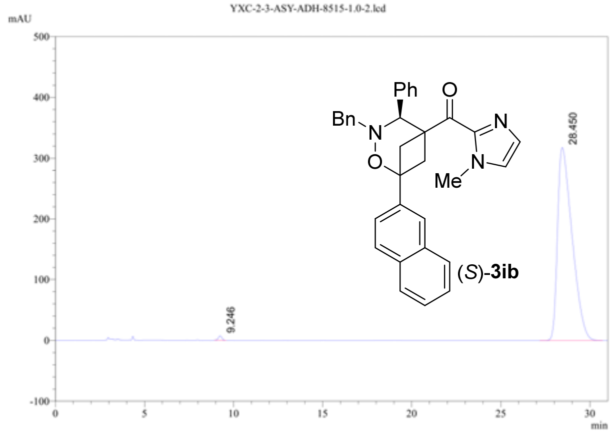

Detector A Channel 1 254nm

Peak#	Ret. Time [min]	Peak Start [min]	Height [mAU]	Area [mAU*min]	Area%
1	9.246	8.925	7328	102588	0.565
2	28.450	27.183	317939	18042693	99.435
Total			325266	18145281	100.000

Supplementary Figure 195. HPLC spectra of (*rac*)-**3ib** and (*S*)-**3ib**

2024-02-28 14:19:44 1 / 1

Analysis Report

SHIMADZU
LabSolutions

Sample Information

Sample Name	: WWB-1-184-RAC-ADH-8515-1.0-1
Sample ID	: WWB-1-184-RAC-ADH-8515-1.0-1
Data File	: WWB-1-184-RAC-ADH-8515-1.0-2.lcd
Method File	: 90-10.lcm
Report Format File	: DEFAULT.lsr
Date Acquired	: 2024/2/28 10:36:28
Date Processed	: 2024/2/28 10:58:30

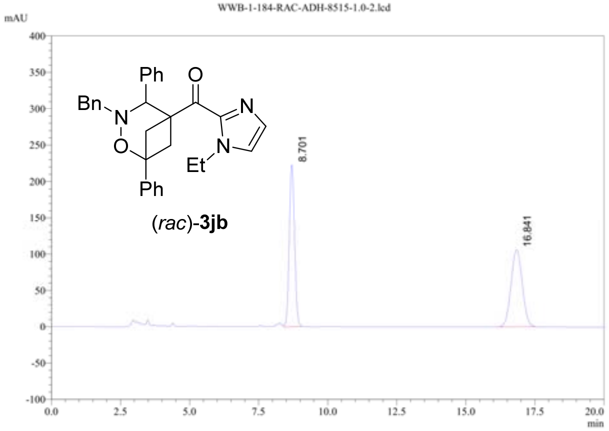

Detector A Channel 1 254nm

Peak#	Ret. Time [min]	Peak Start [min]	Height [mAU]	Area [mAU*min]	Area%
1	8.701	8.408	222841	2987634	49.968
2	16.841	16.150	106146	2991499	50.032
Total			328987	5979134	100.000

2024-03-06 17:27:55 1 / 1

Analysis Report

SHIMADZU
LabSolutions

Sample Information

Sample Name	: YXC-1-179-ASY-ADH-8515-1.0-2
Sample ID	: YXC-1-179-ASY-ADH-8515-1.0-2
Data File	: YXC-1-179-ASY-ADH-8515-1.0-3.lcd
Method File	: 90-10.lcm
Report Format File	: DEFAULT.lsr
Date Acquired	: 2024/3/6 17:07:40
Date Processed	: 2024/3/6 17:27:06

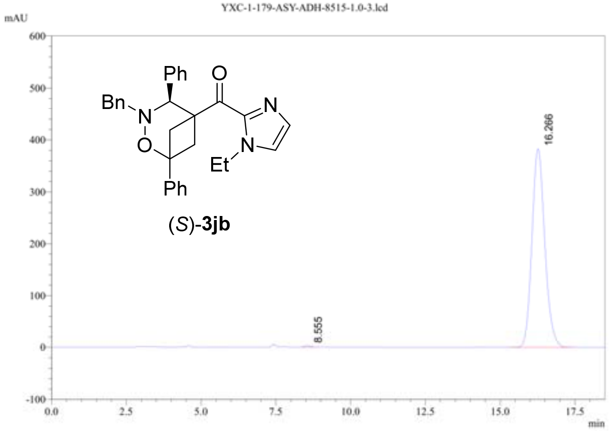

Detector A Channel 1 254nm

Peak#	Ret. Time [min]	Peak Start [min]	Height [mAU]	Area [mAU*min]	Area%
1	8.555	8.333	3044	39418	0.343
2	16.266	15.367	382984	11453630	99.657
Total			386027	11493048	100.000

Supplementary Figure 196. HPLC spectra of (*rac*)-**3jb** and (*S*)-**3jb**

2024-02-28 15:55:12 1 / 1

Analysis Report

SHIMADZU LabSolutions

Sample Information

Sample Name	: WWB-1-185-RAC-ADH-8515-1.0-1
Sample ID	: WWB-1-185-RAC-ADH-8515-1.0-1
Data File	: WWB-1-185-RAC-ADH-8515-1.0-4.lcd
Method File	: 90-10.lcm
Report Format File	: DEFAULT.lsr
Date Acquired	: 2024/2/28 15:31:55
Date Processed	: 2024/2/28 15:51:40

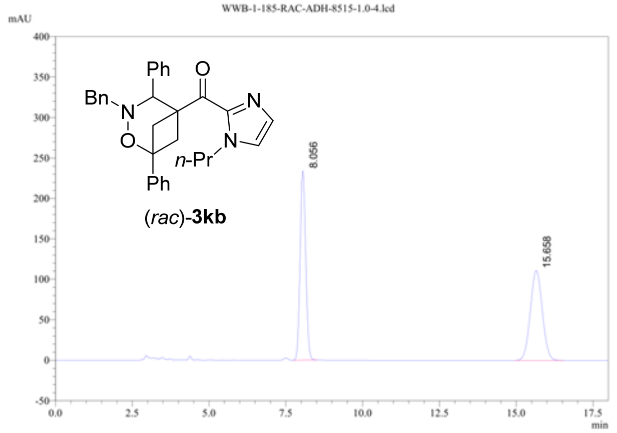

Detector A Channel 1 254nm

Peak#	Ret. Time [min]	Peak Start [min]	Height [mAU]	Area [mAU*min]	Area%
1	8.056	7.733	233959	2972618	49.765
2	15.658	14.950	110535	3000685	50.235
Total			344493	5973302	100.000

2024-03-06 19:07:58 1 / 1

Analysis Report

SHIMADZU LabSolutions

Sample Information

Sample Name	: YXC-1-180-1-ASY-ADH-8515-1.0-2
Sample ID	: YXC-1-180-1-ASY-ADH-8515-1.0-2
Data File	: YXC-1-180-1-ASY-ADH-8515-1.0-3.lcd
Method File	: 90-10.lcm
Report Format File	: DEFAULT.lsr
Date Acquired	: 2024/3/6 18:48:59
Date Processed	: 2024/3/6 19:07:23

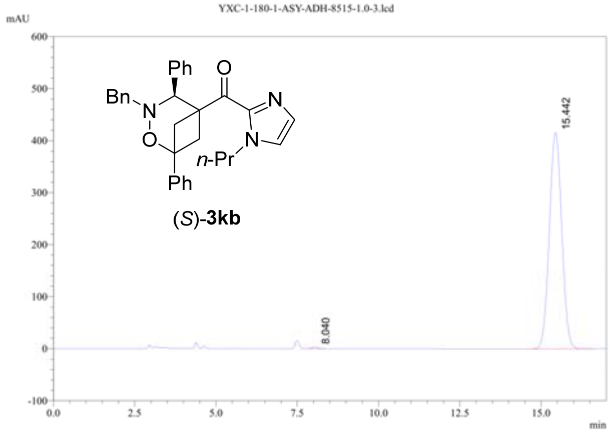

Detector A Channel 1 254nm

Peak#	Ret. Time [min]	Peak Start [min]	Height [mAU]	Area [mAU*min]	Area%
1	8.040	7.808	3758	45534	0.412
2	15.442	14.575	415868	10996378	99.588
Total			419626	11041912	100.000

Supplementary Figure 197. HPLC spectra of (*rac*)-**3kb** and (*S*)-**3kb**

2024-02-28 16:52:30 1 / 1

Analysis Report

SHIMADZU
LabSolutions

Sample Information

Sample Name	: WWB-1-186-RAC-ADH-8515-1.0-1
Sample ID	: WWB-1-186-RAC-ADH-8515-1.0-1
Data File	: WWB-1-186-RAC-ADH-8515-1.0-2.lcd
Method File	: 90-10.lcm
Report Format File	: DEFAULT.lsr
Date Acquired	: 2024/2/28 15:52:28
Date Processed	: 2024/2/28 16:18:53

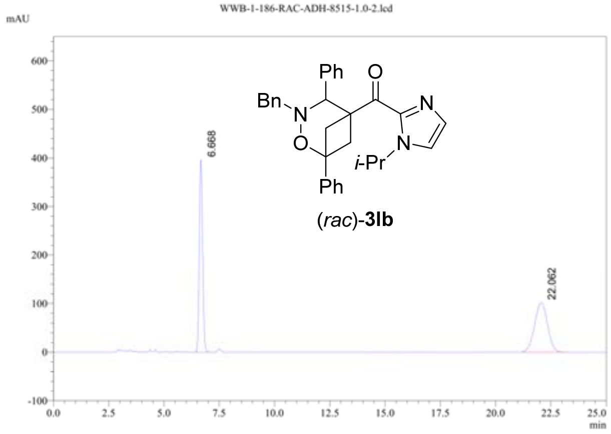

Detector A Channel 1 254nm

Peak#	Ret. Time [min]	Peak Start [min]	Height [mAU]	Area [mAU*min]	Area%
1	6.668	6.375	396383	4130734	49.990
2	22.062	21.175	101756	4132374	50.010
Total			498140	8263108	100.000

2024-03-06 19:34:26 1 / 1

Analysis Report

SHIMADZU
LabSolutions

Sample Information

Sample Name	: YXC-1-180-2-ASY-ADH-8515-1.0-2
Sample ID	: YXC-1-180-2-ASY-ADH-8515-1.0-2
Data File	: YXC-1-180-2-ASY-ADH-8515-1.0-3.lcd
Method File	: 90-10.lcm
Report Format File	: DEFAULT.lsr
Date Acquired	: 2024/3/6 19:09:37
Date Processed	: 2024/3/6 19:33:49

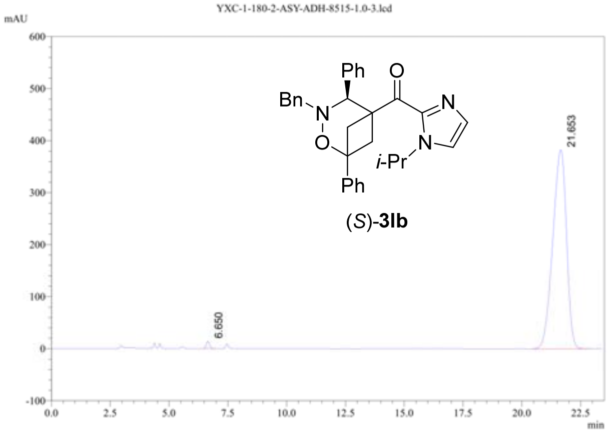

Detector A Channel 1 254nm

Peak#	Ret. Time [min]	Peak Start [min]	Height [mAU]	Area [mAU*min]	Area%
1	6.650	6.450	14209	144911	0.927
2	21.653	20.400	382571	15483960	99.073
Total			396780	15628871	100.000

Supplementary Figure 198. HPLC spectra of (*rac*)-**3lb** and (*S*)-**3lb**

2024-02-28 18:30:18 1 / 1

Analysis Report

SHIMADZU LabSolutions

Sample Information

Sample Name	: WWB-1-188-RAC-ADH-8515-1.0-1
Sample ID	: WWB-1-188-RAC-ADH-8515-1.0-1
Data File	: WWB-1-188-RAC-ADH-8515-1.0-2.lcd
Method File	: 90-10.lcm
Report Format File	: DEFAULT.lsr
Date Acquired	: 2024/2/28 16:50:47
Date Processed	: 2024/2/28 17:20:32

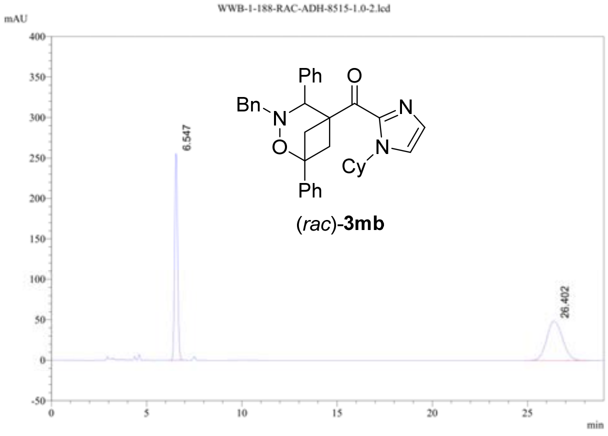

Detector A Channel 1 254nm

Peak#	Ret. Time [min]	Peak Start [min]	Height [mAU]	Area [mAU*min]	Area%
1	6.547	6.183	255439	2746555	49.817
2	26.402	25.142	48448	2766750	50.183
Total			303887	5513306	100.000

2024-03-06 20:08:11 1 / 1

Analysis Report

SHIMADZU LabSolutions

Sample Information

Sample Name	: YXC-1-181-ASY-ADH-8515-1.0-2
Sample ID	: YXC-1-181-ASY-ADH-8515-1.0-2
Data File	: YXC-1-181-ASY-ADH-8515-1.0-3.lcd
Method File	: 90-10.lcm
Report Format File	: DEFAULT.lsr
Date Acquired	: 2024/3/6 19:36:14
Date Processed	: 2024/3/6 20:07:42

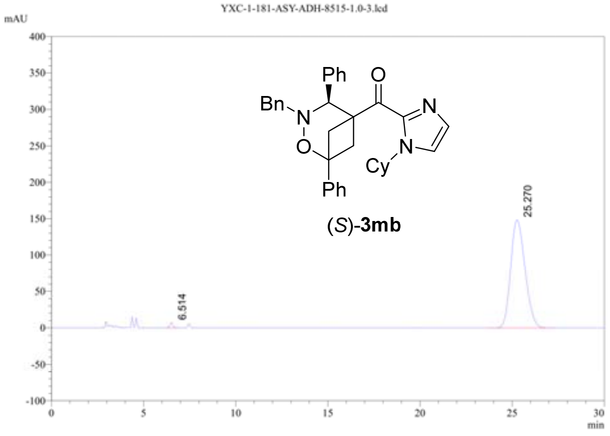

Detector A Channel 1 254nm

Peak#	Ret. Time [min]	Peak Start [min]	Height [mAU]	Area [mAU*min]	Area%
1	6.514	6.225	7080	72969	0.903
2	25.270	23.758	148442	8011080	99.097
Total			155522	8084050	100.000

Supplementary Figure 199. HPLC spectra of (*rac*)-**3mb** and (*S*)-**3mb**

2024-03-25 19:12:50 1 / 1

Analysis Report

SHIMADZU LabSolutions

Sample Information

Sample Name	: WWB-1-131-RAC-ADH-9802-1.0-1
Sample ID	: WWB-1-131-RAC-ADH-9802-1.0-1
Data File	: WWB-1-131-RAC-ADH-9802-1.0-2.lcd
Method File	: 90-10.lcm
Report Format File	: DEFAULT.lsr
Date Acquired	: 2023/12/26 15:47:41
Date Processed	: 2024/3/25 19:12:05

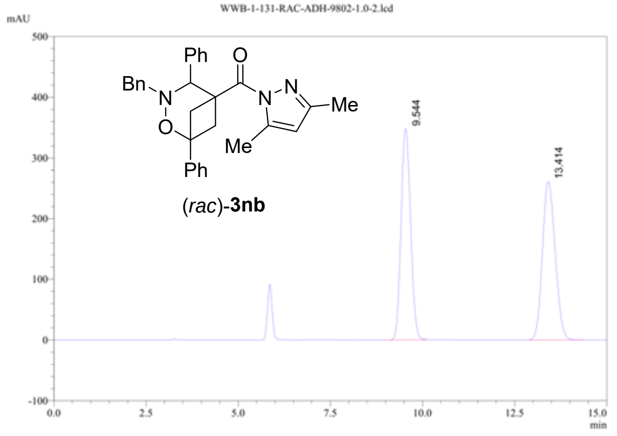

Detector A Channel 1 254nm

Peak#	Ret. Time [min]	Peak Start [min]	Height [mAU]	Area [mAU*min]	Area%
1	9.544	8.967	348171	6236119	49.771
2	13.414	12.850	260692	6293387	50.229
Total			608863	12529506	100.000

2024-03-25 19:14:13 1 / 1

Analysis Report

SHIMADZU LabSolutions

Sample Information

Sample Name	: YXC-2-9-ASY-ADH-9802-1.0-2
Sample ID	: YXC-2-9-ASY-ADH-9802-1.0-2
Data File	: YXC-2-9-ASY-ADH-9802-1.0-3.lcd
Method File	: 90-10.lcm
Report Format File	: DEFAULT.lsr
Date Acquired	: 2024/3/25 18:57:57
Date Processed	: 2024/3/25 19:13:42

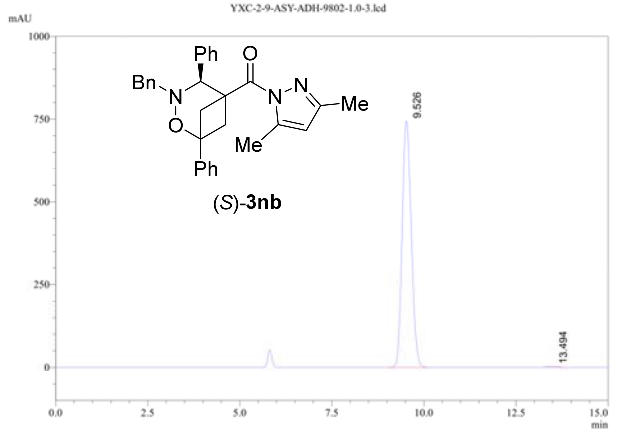

Detector A Channel 1 254nm

Peak#	Ret. Time [min]	Peak Start [min]	Height [mAU]	Area [mAU*min]	Area%
1	9.526	9.050	743690	12798511	99.604
2	13.494	13.250	2817	50840	0.396
Total			746507	12849351	100.000

Supplementary Figure 200. HPLC spectra of (*rac*)-**3nb** and (*S*)-**3nb**

2024-04-18 16:23:17 1 / 1

Analysis Report

SHIMADZU LabSolutions

Sample Information

Sample Name	: WWB-2-33-RAC-ODH-9802-0.8-2
Sample ID	: WWB-2-33-RAC-ODH-9802-0.8-2
Data File	: WWB-2-33-RAC-ODH-9802-0.8-3.lcd
Method File	: 98-02.lcm
Report Format File	: DEFAULT.lsr
Date Acquired	: 2024/4/18 15:59:14
Date Processed	: 2024/4/18 16:13:27

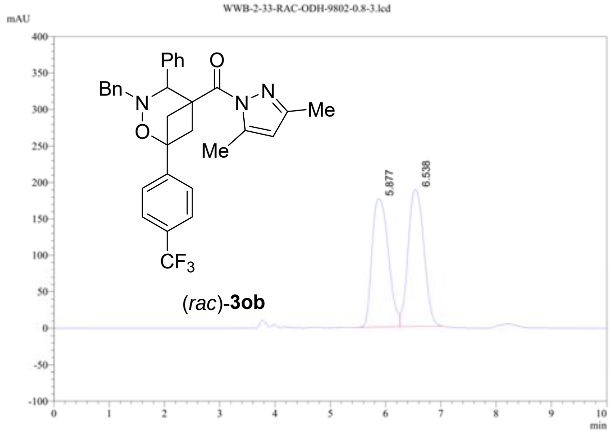

Detector A Channel 1 254nm

Peak#	Ret. Time [min]	Peak Start [min]	Height [mAU]	Area [mAU*min]	Area%
1	5.877	5.492	176209	3620315	49.375
2	6.538	6.258	188121	3711981	50.625
Total			364330	7332295	100.000

2024-04-18 16:23:34 1 / 1

Analysis Report

SHIMADZU LabSolutions

Sample Information

Sample Name	: WWB-2-33-ASY-ODH-9802-0.8-2
Sample ID	: WWB-2-33-ASY-ODH-9802-0.8-2
Data File	: WWB-2-33-ASY-ODH-9802-0.8-3.lcd
Method File	: 98-02.lcm
Report Format File	: DEFAULT.lsr
Date Acquired	: 2024/4/18 16:11:51
Date Processed	: 2024/4/18 16:22:32

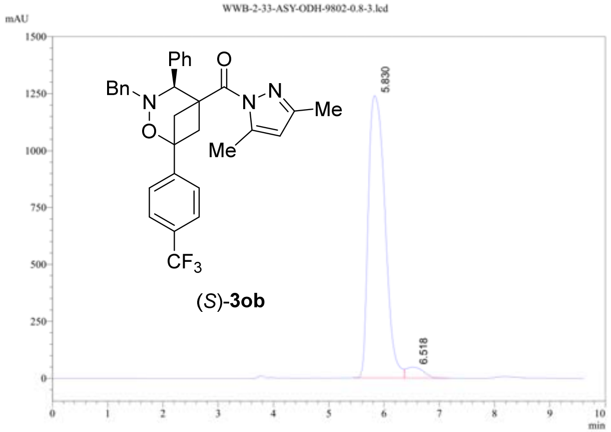

Detector A Channel 1 254nm

Peak#	Ret. Time [min]	Peak Start [min]	Height [mAU]	Area [mAU*min]	Area%
1	5.830	5.433	1238764	25986508	96.127
2	6.518	6.367	47924	1047103	3.873
Total			1286688	27033610	100.000

Supplementary Figure 201. HPLC spectra of (*rac*)-**3ob** and (*S*)-**3ob**

2024-04-22 11:20:21 1 / 1

Analysis Report

SHIMADZU LabSolutions

Sample Information

Sample Name	: WWB-2-32-RAC-ADH-9802-1.0-1
Sample ID	: WWB-2-32-RAC-ADH-9802-1.0-1
Data File	: WWB-2-32-RAC-ADH-9802-1.0-4.lcd
Method File	: 98-02.lcm
Report Format File	: DEFAULT.lsr
Date Acquired	: 2024/4/22 11:06:23
Date Processed	: 2024/4/22 11:17:22

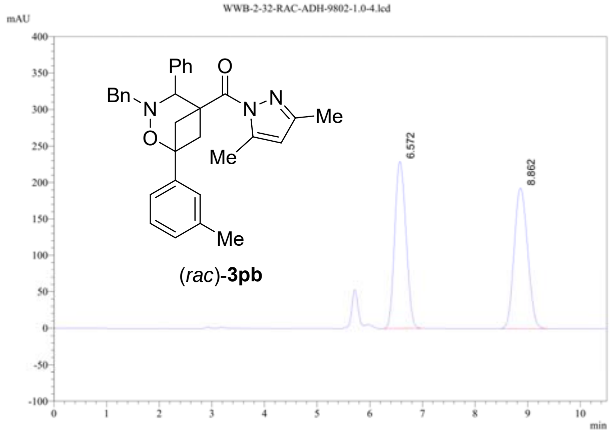

Detector A Channel 1 254nm

Peak#	Ret. Time [min]	Peak Start [min]	Height [mAU]	Area [mAU*min]	Area%
1	6.572	6.267	229194	3403634	49.882
2	8.862	8.475	192796	3419714	50.118
Total			421990	6823348	100.000

2024-04-22 11:30:14 1 / 1

Analysis Report

SHIMADZU LabSolutions

Sample Information

Sample Name	: WWB-2-32-ASY-ADH-9802-1.0-1
Sample ID	: WWB-2-32-ASY-ADH-9802-1.0-1
Data File	: WWB-2-32-ASY-ADH-9802-1.0-2.lcd
Method File	: 98-02.lcm
Report Format File	: DEFAULT.lsr
Date Acquired	: 2024/4/22 11:18:27
Date Processed	: 2024/4/22 11:29:45

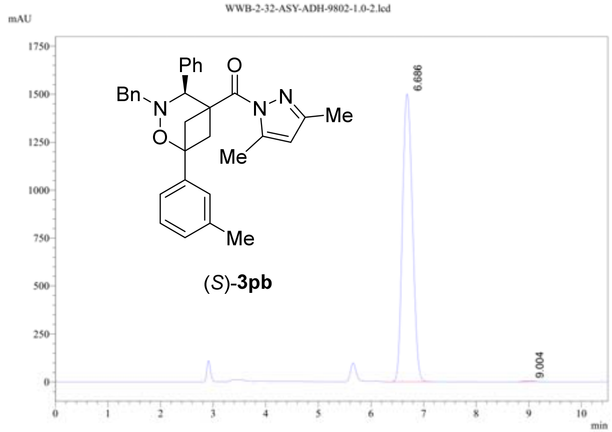

Detector A Channel 1 254nm

Peak#	Ret. Time [min]	Peak Start [min]	Height [mAU]	Area [mAU*min]	Area%
1	6.686	6.342	1500103	19823799	99.753
2	9.004	8.825	4223	49039	0.247
Total			1504326	19872838	100.000

Supplementary Figure 202. HPLC spectra of (*rac*)-**3pb** and (*S*)-**3pb**

2024-05-10 11:05:17 1 / 1

Analysis Report

SHIMADZU LabSolutions

Sample Information

Sample Name	: WWB-2-63-RAC-ADH-9901-1.0-1
Sample ID	: WWB-2-63-RAC-ADH-9901-1.0-1
Data File	: WWB-2-63-RAC-ADH-9901-1.0-2.lcd
Method File	: 98-02.lcm
Report Format File	: DEFAULT.lsr
Date Acquired	: 2024/5/10 10:02:02
Date Processed	: 2024/5/10 10:22:29

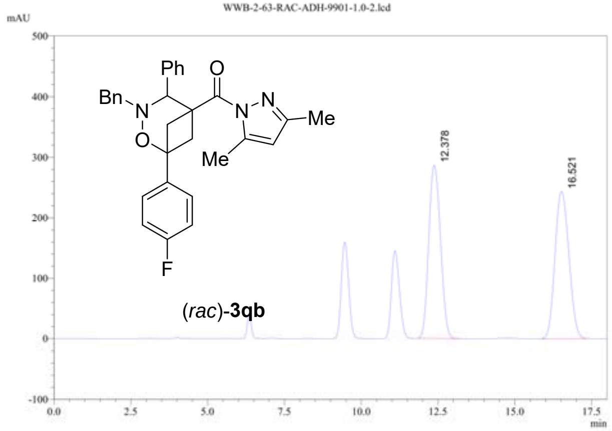

Detector A Channel 1 254nm

Peak#	Ret. Time [min]	Peak Start [min]	Height [mAU]	Area [mAU*min]	Area%
1	12.378	11.858	286561	7636129	49.858
2	16.521	15.858	243812	7679759	50.142
Total			530373	15315888	100.000

2024-05-10 11:05:35 1 / 1

Analysis Report

SHIMADZU LabSolutions

Sample Information

Sample Name	: WWB-2-63-ASY-ADH-9901-1.0-1
Sample ID	: WWB-2-63-ASY-ADH-9901-1.0-1
Data File	: WWB-2-63-ASY-ADH-9901-1.0-2.lcd
Method File	: 98-02.lcm
Report Format File	: DEFAULT.lsr
Date Acquired	: 2024/5/10 10:21:15
Date Processed	: 2024/5/10 10:40:06

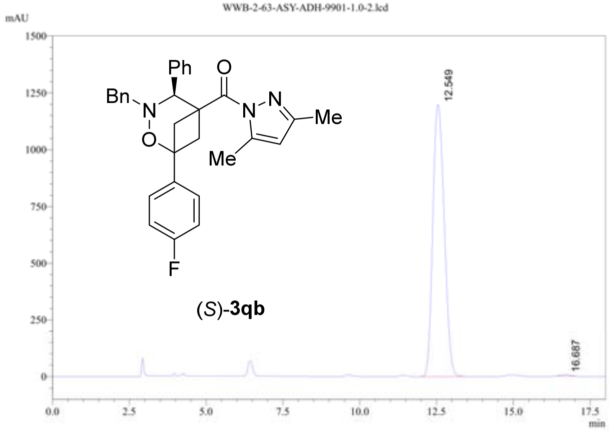

Detector A Channel 1 254nm

Peak#	Ret. Time [min]	Peak Start [min]	Height [mAU]	Area [mAU*min]	Area%
1	12.549	12.000	1198428	30917643	99.585
2	16.687	16.417	5994	128861	0.415
Total			1204422	31046505	100.000

Supplementary Figure 203. HPLC spectra of (*rac*)-**3qb** and (*S*)-**3qb**

2024-04-29 20:15:20 1 / 1

Analysis Report

SHIMADZU LabSolutions

Sample Information

Sample Name	: WWB-2-53-RAC-ADH-9802-1.0-1
Sample ID	: WWB-2-53-RAC-ADH-9802-1.0-1
Data File	: WWB-2-53-RAC-ADH-9802-1.0-3.lcd
Method File	: 98-02.lcm
Report Format File	: DEFAULT.lsr
Date Acquired	: 2024/4/29 19:51:46
Date Processed	: 2024/4/29 20:02:25

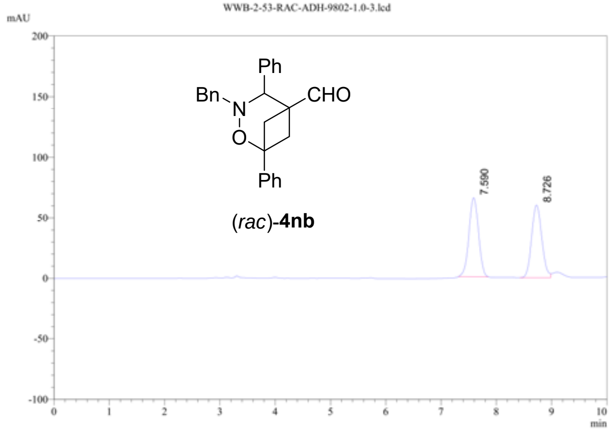

Detector A Channel 1 254nm

Peak#	Ret. Time [min]	Peak Start [min]	Height [mAU]	Area [mAU*min]	Area%
1	7.590	7.317	65377	786229	50.550
2	8.726	8.425	60167	769121	49.450
Total			125543	1555351	100.000

2024-04-29 20:15:41 1 / 1

Analysis Report

SHIMADZU LabSolutions

Sample Information

Sample Name	: WWB-2-53-ASY-ADH-9802-1.0-1
Sample ID	: WWB-2-53-ASY-ADH-9802-1.0-1
Data File	: WWB-2-53-ASY-ADH-9802-1.0-2.lcd
Method File	: 98-02.lcm
Report Format File	: DEFAULT.lsr
Date Acquired	: 2024/4/29 20:03:15
Date Processed	: 2024/4/29 20:14:23

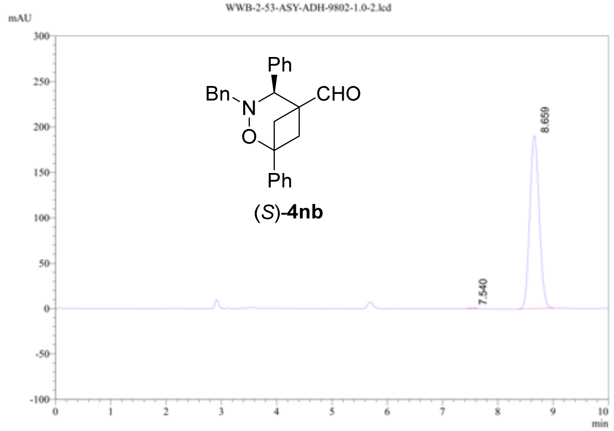

Detector A Channel 1 254nm

Peak#	Ret. Time [min]	Peak Start [min]	Height [mAU]	Area [mAU*min]	Area%
1	7.540	7.417	922	7383	0.326
2	8.659	8.367	190471	2260587	99.674
Total			191393	2267970	100.000

Supplementary Figure 204. HPLC spectra of (*rac*)-**4nb** and (*S*)-**4nb**

2024-04-02 15:21:37 1 / 1

Analysis Report

SHIMADZU LabSolutions

Sample Information

Sample Name	: WWB-2-14-RAC-ADH-9010-1.0-1
Sample ID	: WWB-2-14-RAC-ADH-9010-1.0-1
Data File	: WWB-2-14-RAC-ADH-9010-1.0-4.lcd
Method File	: 90-10.lcm
Report Format File	: DEFAULT.lsr
Date Acquired	: 2024/4/2 15:11:08
Date Processed	: 2024/4/2 15:21:00

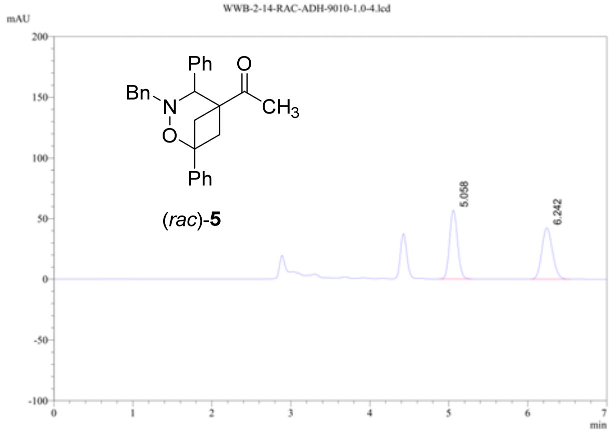

Detector A Channel 1 254nm

Peak#	Ret. Time [min]	Peak Start [min]	Height [mAU]	Area [mAU*min]	Area%
1	5.058	4.875	56902	399463	50.169
2	6.242	6.033	42483	396773	49.831
Total			99385	796236	100.000

2024-04-02 15:27:43 1 / 1

Analysis Report

SHIMADZU LabSolutions

Sample Information

Sample Name	: WWB-2-14-ASY-ADH-9010-1.0-1
Sample ID	: WWB-2-14-ASY-ADH-9010-1.0-1
Data File	: WWB-2-14-ASY-ADH-9010-1.0-2.lcd
Method File	: 90-10.lcm
Report Format File	: DEFAULT.lsr
Date Acquired	: 2024/4/2 15:19:13
Date Processed	: 2024/4/2 15:27:34

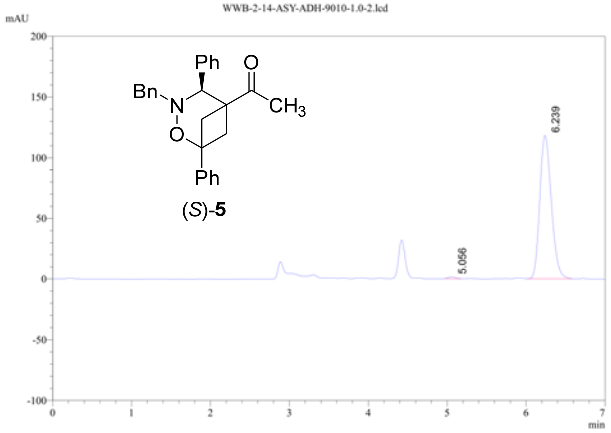

Detector A Channel 1 254nm

Peak#	Ret. Time [min]	Peak Start [min]	Height [mAU]	Area [mAU*min]	Area%
1	5.056	4.967	1444	8995	0.742
2	6.239	6.008	118292	1203188	99.258
Total			119736	1212183	100.000

Supplementary Figure 205. HPLC spectra of (*rac*)-**5** and (*S*)-**5**

2024-03-29 21:59:57 1 / 1

Analysis Report

SHIMADZU LabSolutions

Sample Information

Sample Name	: WWB-2-10-RAC-ADH-9802-1.0-2
Sample ID	: WWB-2-10-RAC-ADH-9802-1.0-2
Data File	: WWB-2-10-RAC-ADH-9802-1.0-3.lcd
Method File	: 90-10.lcm
Report Format File	: DEFAULT.lsr
Date Acquired	: 2024/3/29 21:45:03
Date Processed	: 2024/3/29 21:57:24

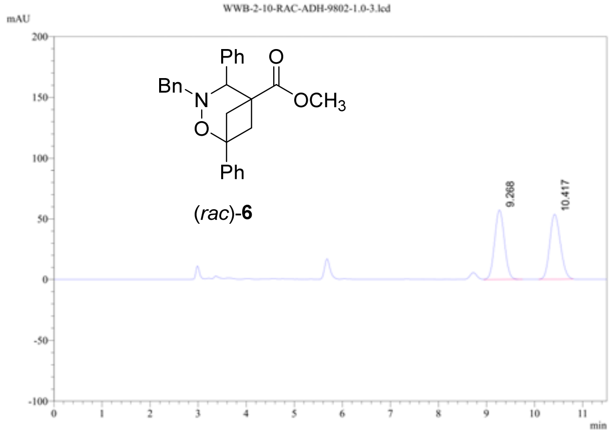

Detector A Channel 1 254nm

Peak#	Ret. Time [min]	Peak Start [min]	Height [mAU]	Area [mAU*min]	Area%
1	9.268	8.942	57347	816263	49.802
2	10.417	10.083	53482	822765	50.198
Total			110829	1639027	100.000

2024-03-29 22:12:52 1 / 1

Analysis Report

SHIMADZU LabSolutions

Sample Information

Sample Name	: WWB-2-10-ASY-ADH-9802-1.0-2
Sample ID	: WWB-2-10-ASY-ADH-9802-1.0-2
Data File	: WWB-2-10-ASY-ADH-9802-1.0-3.lcd
Method File	: 90-10.lcm
Report Format File	: DEFAULT.lsr
Date Acquired	: 2024/3/29 21:58:30
Date Processed	: 2024/3/29 22:12:40

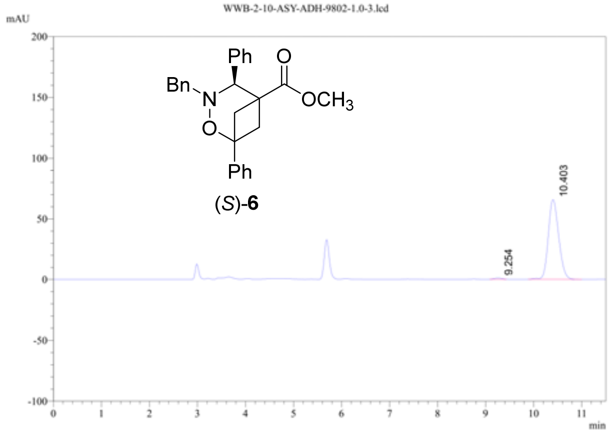

Detector A Channel 1 254nm

Peak#	Ret. Time [min]	Peak Start [min]	Height [mAU]	Area [mAU*min]	Area%
1	9.254	9.083	968	10815	1.079
2	10.403	9.883	65743	991145	98.921
Total			66711	1001960	100.000

Supplementary Figure 206. HPLC spectra of (*rac*)-**6** and (*S*)-**6**

2024-03-29 21:34:54 1 / 1

Analysis Report

SHIMADZU LabSolutions

Sample Information

Sample Name	: WWB-2-11-RAC-ADH-9505-0.8-1
Sample ID	: WWB-2-11-RAC-ADH-9505-0.8-1
Data File	: WWB-2-11-RAC-ADH-9505-0.8-2.lcd
Method File	: 90-10.lcm
Report Format File	: DEFAULT.lsr
Date Acquired	: 2024/3/29 20:58:42
Date Processed	: 2024/3/29 21:17:27

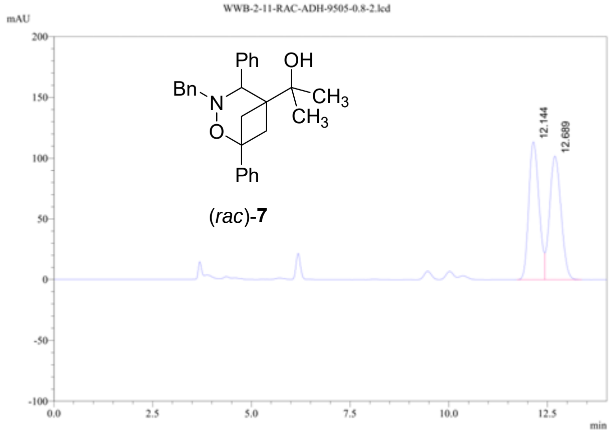

Detector A Channel 1 254nm

Peak#	Ret. Time [min]	Peak Start [min]	Height [mAU]	Area [mAU*min]	Area%
1	12.144	11.725	113722	2059234	50.018
2	12.689	12.433	101936	2057761	49.982
Total			215658	4116995	100.000

2024-03-29 21:35:14 1 / 1

Analysis Report

SHIMADZU LabSolutions

Sample Information

Sample Name	: WWB-2-11-ASY-ADH-9505-0.8-1
Sample ID	: WWB-2-11-ASY-ADH-9505-0.8-1
Data File	: WWB-2-11-ASY-ADH-9505-0.8-2.lcd
Method File	: 90-10.lcm
Report Format File	: DEFAULT.lsr
Date Acquired	: 2024/3/29 21:18:20
Date Processed	: 2024/3/29 21:34:01

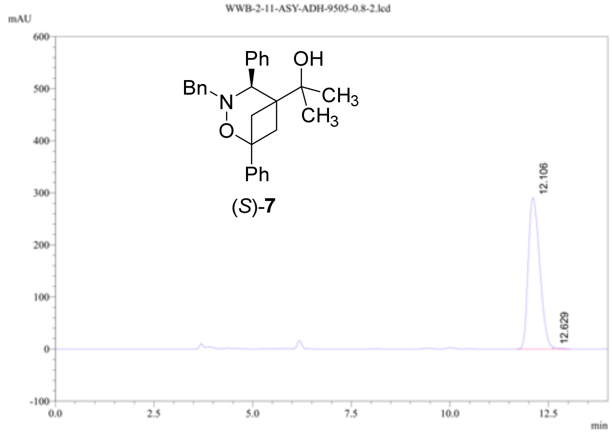

Detector A Channel 1 254nm

Peak#	Ret. Time [min]	Peak Start [min]	Height [mAU]	Area [mAU*min]	Area%
1	12.106	11.692	290471	5863090	99.452
2	12.629	12.617	3282	32312	0.548
Total			293753	5895402	100.000

Supplementary Figure 207. HPLC spectra of (*rac*)-**7** and (*S*)-**7**

2024-04-16 15:46:20 1 / 1

Analysis Report

SHIMADZU LabSolutions

Sample Information

Sample Name	: WWB-2-19-RAC-ADH-9802-1.0-1
Sample ID	: WWB-2-19-RAC-ADH-9802-1.0-1
Data File	: WWB-2-19-RAC-ADH-9802-1.0-3.lcd
Method File	: 98-02.lcm
Report Format File	: DEFAULT.lsr
Date Acquired	: 2024/4/5 15:23:39
Date Processed	: 2024/4/5 15:36:36

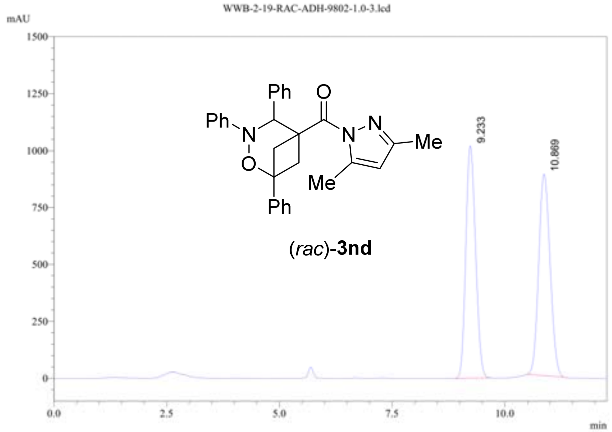

Detector A Channel 1 254nm

Peak#	Ret. Time [min]	Peak Start [min]	Height [mAU]	Area [mAU*min]	Area%
1	9.233	8.858	1020268	15369146	49.775
2	10.869	10.500	884611	15507991	50.225
Total			1904879	30877137	100.000

2024-04-16 15:46:36 1 / 1

Analysis Report

SHIMADZU LabSolutions

Sample Information

Sample Name	: WWB-2-19-ASY-ADH-9802-1.0-1
Sample ID	: WWB-2-19-ASY-ADH-9802-1.0-1
Data File	: WWB-2-19-ASY-ADH-9802-1.0-2.lcd
Method File	: 98-02.lcm
Report Format File	: DEFAULT.lsr
Date Acquired	: 2024/4/5 15:37:15
Date Processed	: 2024/4/5 15:51:25

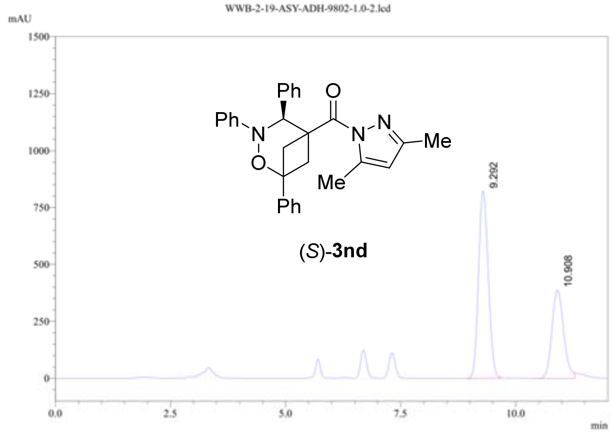

Detector A Channel 1 254nm

Peak#	Ret. Time [min]	Peak Start [min]	Height [mAU]	Area [mAU*min]	Area%
1	9.292	8.883	822236	12066117	63.749
2	10.908	10.433	386980	6861395	36.251
Total			1209215	18927512	100.000

Supplementary Figure 208. HPLC spectra of (*rac*)-**3nd** and (*S*)-**3nd**

2024-04-17 16:45:58 1 / 1

Analysis Report

Sample Information

Sample Name	: WWB-2-29-RAC-ADH-9010-1.0-1
Sample ID	: WWB-2-29-RAC-ADH-9010-1.0-1
Data File	: WWB-2-29-RAC-ADH-9010-1.0-3.lcd
Method File	: 98-02.lcm
Report Format File	: DEFAULT.lsr
Date Acquired	: 2024/4/17 15:56:32
Date Processed	: 2024/4/17 16:17:54

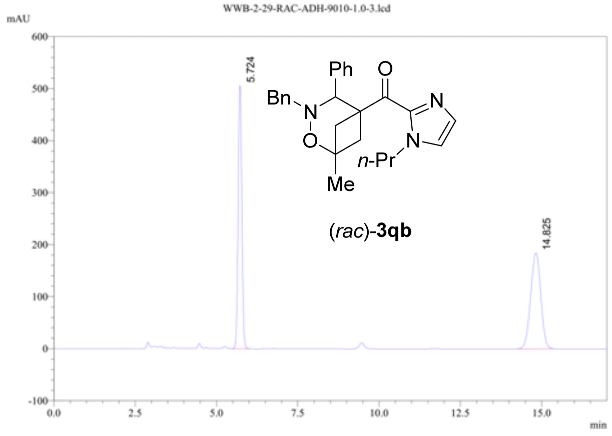

Detector A Channel 1 254nm

Peak#	Ret. Time [min]	Peak Start [min]	Height [mAU]	Area [mAU*min]	Area%
1	5.724	5.467	505887	3939366	49.829
2	14.825	14.250	184001	3966392	50.171
Total			689888	7905758	100.000

2024-04-17 16:46:24 1 / 1

Analysis Report

Sample Information

Sample Name	: WWB-2-29-ASY-ADH-9010-1.0-1
Sample ID	: WWB-2-29-ASY-ADH-9010-1.0-1
Data File	: WWB-2-29-ASY-ADH-9010-1.0-2.lcd
Method File	: 98-02.lcm
Report Format File	: DEFAULT.lsr
Date Acquired	: 2024/4/17 16:18:51
Date Processed	: 2024/4/17 16:43:57

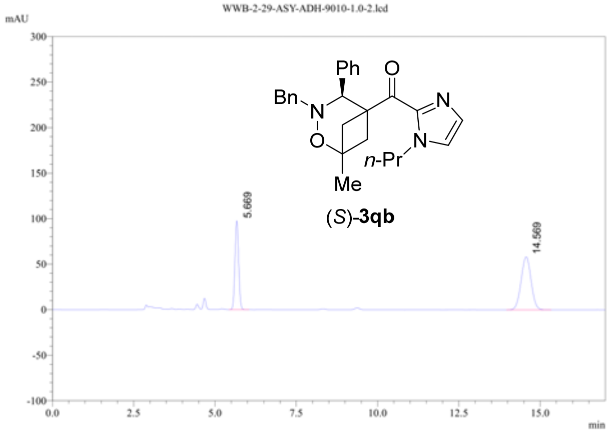

Detector A Channel 1 254nm

Peak#	Ret. Time [min]	Peak Start [min]	Height [mAU]	Area [mAU*min]	Area%
1	5.669	5.425	97440	765456	38.152
2	14.569	13.958	58199	1240886	61.848
Total			155639	2006343	100.000

Supplementary Figure 209. HPLC spectra of (*rac*)-**3qb** and (*S*)-**3qb**

5. Supplementary Computational Details

In order to understand the origin of enantioselectivity, density functional theory (DFT) calculations were carried out using the Gaussian 09 software package.[13-16] The spin-quartet state was calculated for the cobalt(II) complexes because of the high-spin state with lower energy. The stationary structures were optimized using PBE0 method and combined basis set. That is, SDD for Co atom, and 6-31G(d) basis set for all the other atoms.[17] Truhlar and coworkers' SMD solvation model was employed to consider the solvent effect of Dichloromethane.[18] The geometry optimizations were performed without symmetry constraints, and the nature of the extrema was checked by analytical frequency calculations. The intrinsic reaction coordinate (IRC)[19] pathways have been traced to verify two desired minima connected by the transition states. The independent gradient model based on Hirshfeld partition (IGMH) analysis was conducted with Multiwfn and VMD.[20-22] The 3-D images of the calculated structures were prepared using CYLview.[23]

Note: source data are provided with this paper

6. Supplementary References

1. Dhake, K., Woelk, K. J., Becica, J., Un, A., Jenny, S. E. & Leitch, D. C., Beyond Bioisosteres: Divergent Synthesis of Azabicyclohexanes and Cyclobutenyl Amines from Bicyclobutanes. *Angew. Chem. Int. Ed.* **61**, e202204719 (2022).

2. Livingstone, K. et al. Skeletal Ring Contractions via I(I)/I(III) Catalysis: Stereoselective Synthesis of cis-α,α-Difluorocyclopropanes. *ACS Catal.* **12**, 14507-14516 (2022).

3. Guo, R. et al. Strain-Release [2π+2σ] Cycloadditions for the Synthesis of Bicyclo[2.1.1]hexanes Initiated by Energy Transfer. *J. Am. Chem. Soc.* **144**, 7988-7994 (2022).

4. Sharland, J. C. & Davies, H. M. L., One-Pot Synthesis of Difluorobicyclo[1.1.1]pentanes from α-Allyldiazoacetates. *Org. Lett.* **25**, 5214-5219 (2023).

5. Poulsen, P. H., Vergura, S., Monleon, A., Jørgensen, D. K. B. & Jørgensen, K. A. Controlling Asymmetric Remote and Cascade 1,3-Dipolar Cycloaddition Reactions by Organocatalysis. *J. Am. Chem. Soc.* **138**, 6412-6415 (2016).

6. Matassini, C., Parmeggiani, C., Cardona, F. & Goti, A., Oxidation of N,N-Disubstituted Hydroxylamines to Nitrones with Hypervalent Iodine Reagents. *Org. Lett*. **17**, 4082-4085 (2015).

7. Chen, X., Cheng, Z., Guo, J. & Lu, Z., Asymmetric remote C-H borylation of internal alkenes via alkene isomerization. *Nat. Commun*. **9**, 3939 (2018).

8. Wang, X.-B. et al. Rational Design of Chiral Tridentate Ligands: Bifunctional Cobalt(II) Complex/Hydrogen Bond for Enantioselective Michael Reactions. *Org. Lett.* **24**, 3861-3866 (2022).

9. Wang, H.-X. et al. Design of C_1-symmetric tridentate ligands for enantioselective dearomative [3 + 2] annulation of indoles with aminocyclopropanes. *Nat. Commun.* **14**, 2270 (2023).

10. Wang, X.-Y. et al. Cobalt-Catalyzed Asymmetric Dearomative [3 + 2] Annulation of Quinolines, Isoquinolines, and Pyridines. *ACS Catal.* **13**, 11528−11540 (2023).

11. Zhang, J., Su, J.-Y., Zheng, H., Li, H. & Deng, W.-P., $Eu(OTf)_3$-Catalyzed Formal Dipolar [4π+2σ] Cycloaddition of Bicyclo-[1.1.0]butanes with Nitrones: Access to Polysubstituted 2-Oxa-3-azabicyclo[3.1.1]heptanes. *Angew. Chem. Int. Ed.* **63**, e202318476 (2024).

12. Liang, Y., Paulus, F., Daniliuc, C. G. & Glorius, F. Catalytic Formal [2π+2σ] Cycloaddition of Aldehydes with Bicyclobutanes: Expedient Access to

Polysubstituted 2-Oxabicyclo[2.1.1]hexanes. *Angew. Chem. Int. Ed.* **62**, e202305043 (2023).

13. Becke, A. D., Density-functional thermochemistry. III. The role of exact exchange. *J. Chem. Phys.* **98**, 5648 (1993).

14. Stephens, P. J., Devlin, F. J., Chabalowski, C. F. & Frisch, M. J., Ab Initio Calculation of Vibrational Absorption and Circular Dichroism Spectra Using Density Functional Force Fields. *J. Phys. Chem.* **98**, 11623 (1994).

15. Lee, C., Yang, W. & Parr, R. G., Development of the Colle-Salvetti correlation-energy formula into a functional of the electron density. *Phys. Rev. B* **37**, 785 (1998).

16. Frisch, M. J. et al. Gaussian 09, Revision A.02; Gaussian, Inc.: Wallingford, CT, 2009.

17. Hay, P. J. & Wadt, W. R., Ab initio effective core potentials for molecular calculations. Potentials for the transition metal atoms Sc to Hg. *J. Chem. Phys.* **82,** 270 (1985).

18. Marenich, A. V., Cramer,C. J. & Truhlar, D. G., Universal Solvation Model Based on Solute Electron Density and on a Continuum Model of the Solvent Defined by the Bulk Dielectric Constant and Atomic Surface Tensions. *J. Phys. Chem. B* **113**, 6378 (2009).

19. Fukui, K., Formulation of the reaction coordinate. *J. Phys. Chem.* **74**, 4161-4163 (1970).

20. Johnson, E. R. et al. Revealing Noncovalent Interactions. *J. Am. Chem. Soc.* **132**, 6498-6506 (2010).

21. Lu, T. & Chen, F., Multiwfn: A multifunctional wavefunction analyzer. *J. Comput. Chem.* **33**, 580-592 (2012).

22. Humphrey, W., Dalke, A. & Schulten, K., VMD: Visual molecular dynamics. *J. Mol. Graphics.* **14**, 33-38 (1996).

23. Legault, C. Y. C., 1.0b; Universitéde Sherbrooke: Québec, Montreal, Canada; 2009, http://www.cylview.org.
